# Do GPS collars and coded neckbands tell the same story about year-round movements in geese?

**DOI:** 10.1186/s40462-025-00620-y

**Published:** 2026-01-12

**Authors:** Mariëlle L. van Toor, Christen H. Fleming, Niklas Liljebäck, Johan Månsson, Jonas Waldenström, Johan Elmberg

**Affiliations:** 1 Centre for Ecology and Evolution in Microbial Model Systems, Linnaeus University, Kalmar, Sweden; 2 Department of Biology, University of Central Florida, Orlando, FL USA; 3 Smithsonian Conservation Biology Institute, Front Royal, VA USA; 4 Grimsö Wildlife Research Station, Department of Ecology, Swedish University of Agricultural Sciences, Riddarhyttan, Sweden; 5 Department of Environmental Science, Kristianstad University, Kristianstad, Sweden

**Keywords:** Animal telemetry, Greylag goose *Anser anser*, *Branta*, Auto-correlated kernel density estimators, Capture mark recapture/resighting, Comparative study, Seasonal migration

## Abstract

**Background:**

GPS telemetry has become the norm for the tracking of large-bodied bird species, whereas management and conservation of populations often rely on low-tech methods such as capture-mark-resighting (CMR). Direct evaluations of the comparability of the respective outcome from these methods remain rare despite being crucial for comparative studies and management decisions. Here, we investigated whether GPS tracking and CMR lead to same conclusions about seasonal migration and year-round space use. We chose greylag geese (*Anser anser*) as a study species, for which a long record of both coded neckband reports and GPS tracking are available, and whose management relies on CMR data.

**Methods:**

Our data set was comprised of neckband reports and GPS tracks collected for birds from five capture sites in Sweden (*n* = 665 neckband birds; *n* = 156 GPS collar birds). We evaluated the similarity of movement metrics and year-round space use derived from continuous-time movement models and auto-correlated kernel density estimators. We further quantified overlap of spatial range estimates between tracking methods for the breeding period and the wintering season. We approximated spatial observation bias by contrasting range estimates estimated with and without the use of a debiasing algorithm.

**Results:**

We found that estimates of space use derived from CMR and GPS tracking were in general agreement: average year-round space use for most individuals was similar even if means among tracking methods differed among all individuals per method (CMR: $5.07\times10^5 \mathrm{km}^2$; GPS: $1.76\times10^5 \mathrm{km}^2$), and mean overlap of range estimates for summer and winter did not differ depending on whether the comparison was with the same, or differing tracking methods. Movement metrics differed considerably between methods whenever the CMR data captured behaviour at a different temporal scale than GPS (position & velocity autocorrelation), and else in agreement with GPS tracking (periodicity).

**Conclusion:**

Our study suggests that the historical and current use of coded neckband data for greylag goose management decisions is appropriate regarding space use of migratory greylag geese in Europe. Understanding whether the existing reporter network can capture changes to the migratory behaviour of greylag geese including short-stopping of migration will however require additional in-depth analyses.

**Supplementary information:**

The online version contains supplementary material available at 10.1186/s40462-025-00620-y.

## Background

Mapping routes and timing of seasonal migration is essential for sustainable management of bird populations, whether a species is an important game bird managed for harvesting [1], of key conservation concern [2], or due to conflicts with agriculture or other human interests [3, 4]. While historically migration routes were investigated using a combination of approaches including direct observations [5], large-scale bird banding efforts [6], analysis of observation reports [7], or approaches such as stable isotope analysis of feather samples [8] and population genetics [9, 10], telemetric approaches have become the increasingly dominant method of choice [11–13], not least due to the unprecedented volume of data they can provide.

All remote telemetric methods have in common that they generate location histories for tagged individuals, often without any need for human intervention or observation post-tagging during the data collection stage. Especially data produced by GPS devices have negligible variation regarding the success of location attempts and the quality of observations in most habitats (but see [14, 15] ), leading to comparatively minor spatial biases in the collected data, though the sampling interval may vary with charge level. Today it is possible to acquire long-term GPS data or even life-time trajectories of individuals in high spatial and temporal resolution, either remotely via data transmission to satellites or GSM networks, or by retrieving logged data when recapturing individuals [16–18]. Data collected in this way have the potential to illustrate both small-scale movements and large-scale migratory processes in detail, including mechanistic flight patterns, but also ontogenetic changes in movement as well as diel rhythm and habitat use e.g., during stopovers or on wintering grounds [18].

GPS telemetry contrasts with traditional capture-mark-recapture/resight (CMR) approaches for mapping movements, such as bird banding with markers that can either be identified in the field (e.g. wing tags, colour rings, neckbands) or require recapture of the individual for identification (e.g. tarsal rings). These methods produce fewer observations per individual, and in many cases just where a bird was banded and subsequently recovered, in most cases after death. In other words, every location registered for an individual relies on human observation, with no knowledge about what happened between observation events. Hence, the probability of such observations depends on the density of dedicated reporters, the detectability of marked birds, and possible differences in loss of markers between groups [7, 19, 20]. Depending on the study design and area, data collected via CMR approaches can suffer from spatial and temporal biases because of the distribution of observers and variation in their effort [21], and can differ e.g. with weekday or holiday periods. However, CMR may also provide advantages over remote telemetry approaches, and over GPS telemetry in particular. For instance, marking with bands or other identifiers is less costly than deployment of telemetry devices. Consequently, CMR-based studies often include a much larger sample of individuals, hence the conclusions about spatial and temporal patterns of migration tend to be more representative of the population. Furthermore, the impact of the marking method on an individual animals’ behaviour and movements also needs to be considered when choosing a method for tracking birds. Bands and similar markers, including colour rings and neckbands, are considered to impose lower physiological restrictions and overall impact than telemetry devices [22]. GPS tags are comparatively large and heavy, although their impact (on survival, reproductive success, behaviour, etc.) varies with weight/size, species group, tag design, and attachment method [22, 23]. If GPS devices affect migration patterns, e.g. the timing of migration events [24], comparative studies will in many cases be dependent on historical CMR data for standardisation of other methods. This is especially important in long-distance migrants with predominantly flapping flight, as large devices have been shown to delay migratory decisions, slow down migration, and affect survival [22, 24–27].

Thus, when designing a study for mapping a species’ annual and migratory movements, a balance must be struck that considers the research goals, the sample sizes (i.e. the number of independent individuals) required to achieve those goals, ethical considerations, data characteristics, and other constraints (effort, monetary limitations, biases). However, this is under the assumption that both remote telemetry and CMR approaches result in equivalent answers to the research question, at least within the margin of error of the method used. How well this assumption holds up is much less clear than the benefits and counterarguments for either approach. Most studies are based on a single tracking method, thus lacking a direct comparison (but see [28]). Understanding whether different methods tell the same story of a species’ year-round movements is however pivotal, not least for methodological considerations but also how to maximise conservation and management gain [29, 30].

One promising group of species for such comparisons are geese. These birds are large herbivores that have been subject to intensified management efforts in Europe and North America in recent decades [4, 31]. Some species are critically endangered and managed mainly from a conservation perspective (e.g. lesser white-fronted goose *Anser erythropus*, [32]), whereas other species have increased to unprecedented abundance (e.g. [33]). The latter species include greylag goose (*Anser anser*) and barnacle goose (*Branta leucopsis*), which are actively managed in part of their range to reduce conflict with agriculture as well as degradation of natural habitats due to over-grazing [4, 30, 34–37]. Coded neckbands (without GPS) have been used for more than 70 years to map migration patterns and to delineate management units (flyways; sub-populations) of rare as well as super-abundant geese [38–40]. Data bases for geese with coded neckbands contain millions of re-sightings, which are mainly the result of volunteer and citizen-science efforts [41]. These data have been used extensively to devise ambitious management strategies and prioritize efforts, calling for an evaluation of the quality of the former. At this point, it is crucial for management and conservation to establish how well neckband observations correspond to conclusions drawn from GPS data, and vice versa. In this sense geese can also serve as models for other taxa of migratory birds in which a combination of CMR and remote animal telemetry is available, either from GPS devices or other methods.

Here, we assessed whether location data generated by different tracking methods can lead to similar conclusions about annual spatio-temporal patterns, including migration, using either GPS tracking or reported sightings of greylag geese with individually coded neckbands. Over a period of six years (2017–2022), we captured a total of 887 greylag geese at five breeding locations along a latitudinal gradient in Sweden and deployed either GPS collars (*n* = 156) or neckbands with a unique identifying code (*n* = 665). Specifically, we addressed the following questions: i) do models based on GPS collar data and neckband observations produce the same outcome with regard to movement metrics?; ii) are estimated annual migratory ranges similar for GPS collar and neckband birds, and how congruent are range estimates, per capture site and tracking method?; iii) do potential spatial biases affect range estimates for neckband birds?

## Methods

### Overview of study species, capture sites, and data collection

Five capture sites covering a longitudinal gradient from 55^∘^ to 61^∘^ N in Sweden were used: Svedala (hereafter S1; 55^∘^ 33’ N, 13^∘^ 14’ E), Kristianstad (hereafter S2; 56^∘^ 5’ N, 14^∘^ 21’ E), Örebro (hereafter C1; 59^∘^ 10’ N, 15^∘^ 23’ E), Nyköping (hereafter C2; 58^∘^ 58’ N, 17^∘^ 9’ E), and Hudiksvall (hereafter N; 61^∘^ 43’ N, 17^∘^ 6’ E). Breeding and moulting greylag geese were caught while foraging in fields, pastures, or lawns near water during the month of June in 2017–2022 (see [42] for more details about capture sites and methods). In addition to tarsal steel bands, geese were fitted with either a neckband with an individual 3-digit code (hereafter “neckband”) or a solar-powered GPS/GSM tracking device integrated into a neck collar (hereafter “GPS collar”).

Neckbands were engraved with a 3-digit code which can easily be read by observers in the field. Observers across Europe - often including citizen scientists, researchers, and birdwatchers - opportunistically report their sightings of geese with neckbands to online databases. In the case of greylag geese in Sweden, sightings are reported to the database of European Colour-Ring Birding (http://www.cr-birding.org, hereafter CR Birding; prior to 2025, sightings were reported to geese.org, [41]). The observers note the code and colour of the neckband, location, date, and behaviour, and often supplement their sightings with photographs. New reports are managed on a regular basis, and suspicious observations are deleted if a verification cannot be obtained from the reporter. Despite this process, the dataset might contain erroneous reports.

GPS collars had an engraved 3-digit code similar to those in neckbands, albeit in smaller print and on one side only. Throughout the study period, we used three different models of GPS collars, specifically the OrniTrack OT-N35 (weight: 40 g, inner diameter: 35 mm) and OT-N44 (weight: 50 g, inner diameter: 44 mm), both produced by Ornitela (Ornitela UAB, Vilnius, Lithuania) and devices produced by Made-by-Theo (Theo Gerrits, weight: 47.9 g; inner diameter: 46 mm). The weight of GPS collars corresponded on average to 1.6% of goose body mass, and in no case exceeded 2.5%.

Geese were aged as juvenile or adult based on plumage and sexed by examining the shape of genitals by cloacal inspection. At capture sites C1, S1, and S2, adult birds were randomly selected for being fitted with either a neckband or a GPS collar. Most juveniles were fitted with a neckband. At capture sites N and C2, adult females were randomly selected for either a neckband or a GPS collar, whereas males were only fitted with a neckband. All catching and handling were done according to permits from the Animal Ethics Committee of Central Sweden (permit numbers 5.8.18–03584/2017 and 5.8.18–07375/2021).

Data from the GPS collars were accessed via Movebank (made-by-theo tags) or directly from the database of the manufacturer (Ornitela tags). All data consisted of georeferenced timestamps of observations, identifiers for the tags and the individual goose on which it was deployed, as well as additional sensory information and the number of GPS satellites contributing to the recorded location. Observations of neckband birds were accessed from geese.org (now located on http://www.cr-birding.org) and consisted of, among others, records of location, date, time, and neckband code. Data for each individual were associated with the capture site and date, sex, and age at capture.

### Data preparation

As the capture of birds was untargeted it was possible for several individuals of the same family to be caught and subsequently tagged. As individuals within goose families often move and migrate together until the next breeding season [43, 44], they cannot be considered as independent. To alleviate possible biases arising from family groups, we censored all data of juveniles using April 01 of an individual’s second spring as a cut-off date, a time when previous behavioural studies have shown that offspring have become independent from their parents [43, 45]. We further identified likely mating pairs using personal field observations, remarks noted by observers, and GPS data to identify birds moving together. For each identified pair, we retained data from the individual with the longer tracking duration and higher number of observations, and censored its respective partner individual. This resulted in the censoring of 22 GPS collar birds (S1: *n* = 1; C1: *n* = 9; C2: *n* = 4; N: *n* = 6), and eight neckband birds (C1: *n* = 1; C2: *n* = 2; N: *n* = 5).

Neckband reports were further filtered to exclude erroneous observations and potential conflicts with other marking schemes (e.g., duplicated codes) as follows: We retained only a single observation per bird and day, thus excluding 153 observations. We further made a rough estimate of ground speed (geodesic distance between subsequent observations divided by time between observations). No individual was found to have exceeded on average 3.65 m/s (corresponding to 315 km/day), well below observations of migrating greylag geese [46]. One individual, O91, was once falsely reported as 091. As the first digit was always a letter, we were confident that this report referred to individual O91, and we retained the observation in the data set. After the preprocessing, the data set contained 6,963 observations for 496 neckband birds (range: 1–92 observations per bird; median: 11), with 461 birds having been reported at least twice. Reporting statistics per site can be found in Table S1 in Appendix A. GPS collars were also reported, but only in very few cases: in total, GPS collar birds yielded only 20 neckband observations of four birds. We thus decided to exclude these observations from the data prior to the analyses.

We also applied some pre-processing steps to the GPS data. First, we removed any scheduled locations during which the tags failed to connect to any GPS satellites. We further applied a speed filter to remove erroneous locations. We used a relatively lenient speed filter of 40 m/s, which was based on airspeed distributions of migratory greylag geese: we used the mean + 3 standard deviations reported by [46] (i.e. 24.91 m/s), and assumed a maximum wind support of 15 m/s. While thinning the data was not strictly necessary for the approach we used to compare neckband observations and GPS tracking, we found that fitting models at the full temporal resolution was not computationally feasible. We thinned the data, retaining the location closest to solar noon on each day, resulting in a time interval between observations of approximately 24 hours (see Appendix B). After the preprocessing, 86,634 observations from 119 GPS collar birds remained in the data set (range: 5–1,986 observations per bird; median: 606).

**Fig. 1:**
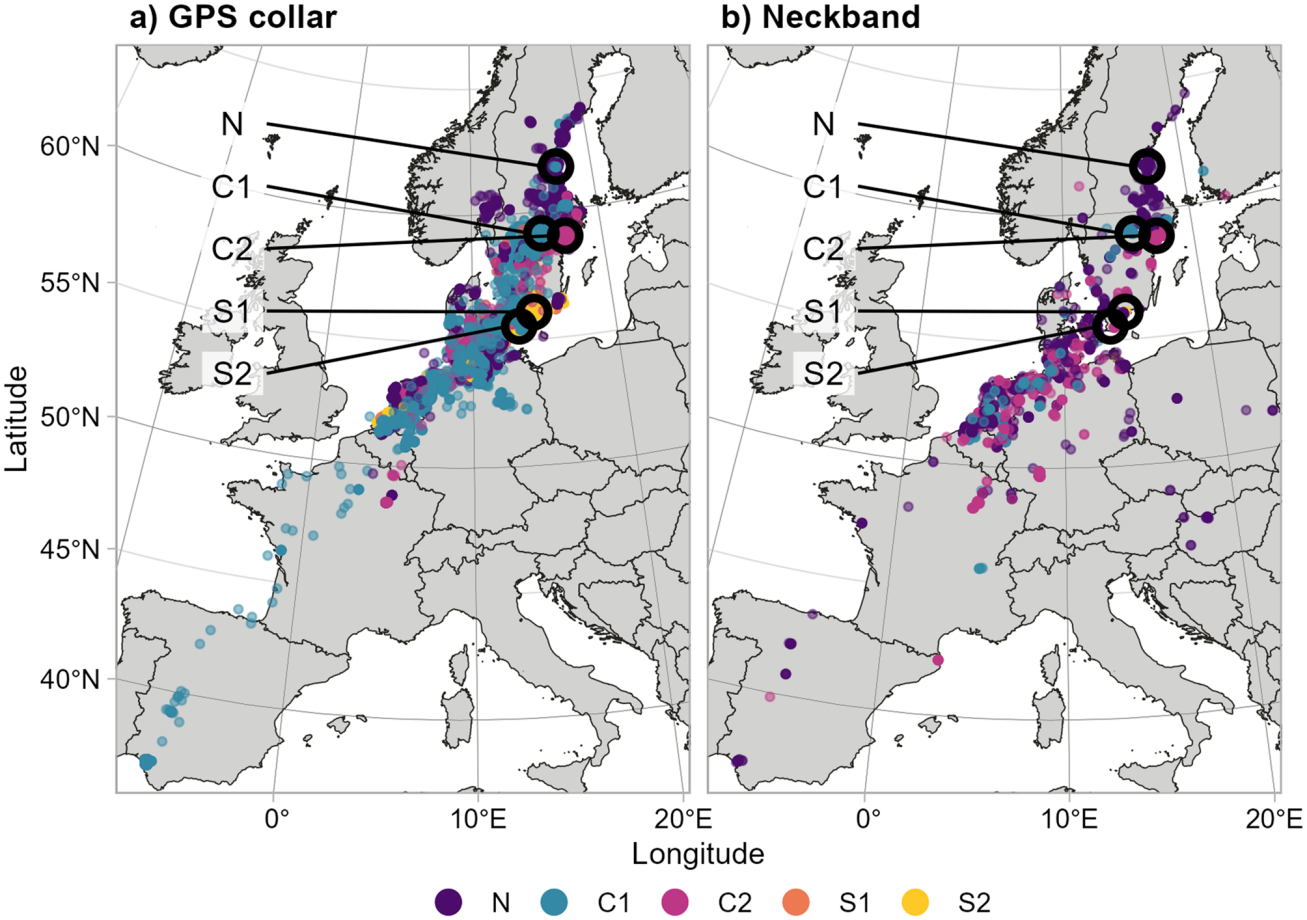
Overview of the location data. GPS tracking data are shown in the left-hand panel (**a**); the right-hand panel (**b**) shows observations of neckband birds reported to CR Birding. Open circles indicate the five capture sites, and location data are coloured to indicate capture site (N: North; C1, C2: Central; S1, S2: South). The map is shown in an oblique Lambert azimuthal equal area projection

The resulting samples sizes, with the number of tracking days and observations, are listed in Table 1, and the tracking data for either method are shown in Fig. 1.

**Table 1:** Overview over sample sizes

	GPS collar birds		Neckband birds	
	Sample size	Tracking days	Sample size	Tracking days
Capture site	**total** (m/f/u)	total (mean per bird)	**total** (m/f/u)	total (mean per bird)
Hudiksvall (N)	8 (0/ 8/ 0)	8,215 (1027)	240 (146/ 90/ 4)	7,853 (15)
Örebro (C1)	45 (25/ 19/ 1)	36,330 (807)	90 (42/ 41/ 7)	687 (6)
Nyköping (C2)	11 (2/ 9/ 0)	10,107 (919)	152 (92/ 58/ 2)	2,962 (18)
Svedala (S1)	31 (16/ 14/ 1)	14,029 (453)	8 (4/ 4/ 0)	47 (3)
Kristianstad (S2)	24 (12/ 12/ 0)	17,953 (748)	6 (2/ 4/ 0)	49 (2)
**Total**	119 (55/ 62/ 2)	86,634 (728)	496 (286/197/ 13)	7,853 (14)

The table shows the number of birds for each capture site and the available tracking days separately for both tracking methods. Tracking days refers to the number of days for which any observation (GPS fix for GPS collar birds, neckband report for Neckband birds) was available for a given individual. Note that the numbers shown here include only those individuals retained for the analyses; i.e. censored individuals (see *Data preparation*) are not shown here. Sample sizes are shown both as total and broken down by sex (m: male; f: female, u: unknown)

### Continuous-time movement models

We chose to address our study questions using a framework based on continuous-time movement models (hereafter ctmm, [47]). This approach is based on fitting movement models to the autocorrelation structure of the data [48, 49], and can estimate range distributions sensu Alston et al. [50] via auto-correlated kernel density estimators [51, 52]. This approach has several advantages for comparing movement and space use including: a) it does not require data collected at regular intervals and takes into account position error; b) it is scale-insensitive to sampling frequency and position error as long as the sampling interval and position error are smaller than the movement behaviour of interest [53]; c) seasonal migration can be taken into account via the implementation of non-stationary, periodic mean locations [54], and d) it provides robust methods for estimating averages at the population level [55]. As part of this study, we also introduce an update to the ctmm-package including a new method for the calculation of meta-analytic population means for models with non-stationary mean locations (included in version 1.2.1 of the ctmm-package, [56]). Our update to population-level averaging is ideally suited for estimating population-level averages, or in this case averages for each capture site, for seasonal migratory movements [55].

We implemented continuous-time movement models in two ways. First, we applied ctmms to data covering the full annual cycle to estimate movement parameters reflecting large-scale movement. We implemented these models with periodic means [54, 57] to account for shifts of individuals’ positions resulting from seasonal migration. We computed semi-variograms (bin width: 24 hours) for each individual, and averaged them per capture site and tracking method. We visually inspected the semi-variograms to determine whether semi-variance approximated an upper limit indicating range resident behaviour (see [47, 49] ). For GPS collar birds, we found that the semi-variograms based on the full annual cycle displayed range resident behaviour with a periodicity of approximately one year for all capture sites (see Fig. S1 in Appendix A). This was expected for the birds from the three northern-most locations, but not necessarily for the birds from the southernmost sites (S1, S2), since some of the latter remain resident year-round [42, 58]. For neckband birds, the semi-variograms were similar to those derived from GPS collar birds in the case of birds from capture sites N, C1, and C2 (Fig. S1). For sites S1 and S2, however, there were very few individuals with multiple observations (S1: *n* = 5, with 2–5 observations per bird; S2: *n* = 4, with 2–4 observations per bird), and the resulting semi-variograms did not fit the expected visual pattern (Fig. S1). Based on these observations, we decided to exclude neckband birds from S1 and S2 from the subsequent analyses. For all other birds, we used the model selection functionality included in the ctmm R-package [47] to fit range-resident continuous-time movement models with a non-stationary mean (period length: one year) to each individual separately. The model selection iterates over several alternative models, including an Ornstein-Uhlenbeck (OU, [48, 49]) model which includes a parameter for the auto-correlation of position (*τ*_*pos*_), and an Ornstein-Uhlenbeck-Foraging (OUF, [48, 49]) model which includes parameters for the auto-correlation of velocity (*τ*_*v*_) in addition to *τ*_*pos*_. A null model lacking any auto-correlation parameters (independent and identically distributed IID, [47]) is also included. The model selection process uses AICc for the autocorrelation model and root mean square predictive error for the trend model to rank alternative models and also fits and compares models with different harmonics of the specified period length. We retained the highest-ranking model for each individual.

We further applied ctmms to data covering just part of the annual cycle (summer: May – July; winter: November – January). These models were implemented using stationary mean positions, and were intended for investigating the effect of reporting bias. With the exception of the above differences, we followed the same procedure for fitting sesaonal models as described above.

### Comparing model and range distribution estimates between tracking methods

We considered two groups of metrics derived from the year-round data to compare inferences drawn from GPS tracking data and neckband observations, respectively. First, we decided which models would contribute to the mean models and range distributions per tracking method and capture site. For some individuals, IID models, which are defined by a single parameter (range area) and assume infinite speed, were indicated as the best model. Since IID models do not reflect the actual behaviour of birds well and their selection indicates that movement cannot be resolved, and because these models were selected only for individuals with few data points (range: 4–32 observations for neckband birds; 5–17 observations for GPS collar birds), we decided to exclude all individuals for which an IID model was selected from the subsequent analyses. Models could be successfully fitted to 329 (out of 461 birds with > 1 observation) neckband individuals, out of which 226 could meaningfully reflect actual goose movement (i.e., non-IID models). We were able to fit ctmms to 114 (out of 119 birds) GPS collar birds, out of which three had IID models as the highest-ranking model. The final number of individuals considered for the analyses were thus 226 neckband birds (per capture site: S1: *n* = 0; S2: *n* = 0; C1: *n* = 19; C2: *n* = 91; N: *n* = 116), and 111 GPS collar birds (per capture site: 1: *n* = 26; S2: *n* = 23; C1: *n* = 43; C2: *n* = 11; N: *n* = 8).

Secondly, we estimated site-level averages for the remaining models per capture site and tracking method. We chose three metrics: i) position autocorrelation *τ*_*pos*_, which can be interpreted as the time that it takes an individual to cross its entire year-round range; ii) velocity autocorrelation *τ*_*v*_, which indicates the time period over which movement behaviour persists; and iii) periodicity, which estimates the degree to which the mean position of the individual can be explained by the position on the period length (here: 1 year). We estimated for each of these parameters the mean and 50, 75, and 95% confidence intervals (CI) around the mean per capture site and tracking method where possible. As exact parametrisations of models could differ among individuals, sample sizes for averaging varied with movement parameter. Only individuals with finite estimates and an effective sample size for the mean ctmm process ≥ 1 contributed to mean estimates. Finally, we derived space use estimates for individuals using autocorrelated kernel density estimators (aKDE), which estimates space use under the assumption that the movement of individuals continues indefinitely based on the movement process estimated using the respective ctmm for that individual. We accounted for potential sampling biases with a weight optimisation algorithm which minimises the mean integrated square error of the aKDE [59]. We then applied the averaging algorithm for aKDEs to derive estimates for the mean area of 95% utilisation distributions (UD) for each tracking method and capture site. We inspected the effective sample size of individual aKDEs, which here refers to the number of independent observations available for estimating space use (i.e., the equivalent number of independent observations that obtain an estimate with the same quality). We retained all individuals from the N, C1, and C2 capture sites for which the area estimate had an effective sample size > 1 (neckband birds: *n* = 190; GPS collar birds: *n* = 60).

### Spatial congruence between range estimates derived from GPS collar data and neckband observations

We investigated the degree of overlap of range estimates derived from birds tracked with either GPS or neckbands in addition to comparing the overall area estimates of year-round distribution ranges between tracking methods. It was not feasible to compute site-level range estimates (see [55]), likely due to a low sample size of entire range crossings by individuals. Instead, we decided to calculate overlap between UDs of all possible pairs of birds tagged in the same capture site. We computed overlap using the Bhattacharyya coefficient [60] between year-round aKDEs for pairs of birds where one individual was tracked with GPS collar and the other with a coded neckband to determine average agreement between the tracking methods. To provide a point of reference, we also computed overlap for pairs of birds where either both individuals were tracked with neckbands, or both with GPS collars. The Bhattacharyya coefficient here measures the intersection area between the 2D autocorrelated kernel density estimates, and ranges from 0 (no overlap) to 1 (complete congruence). Only overlap estimates with an effective sample size ≥ 1 were considered.

### Effect of sampling bias on range estimates

We investigated whether range estimates were affected by sampling bias differently depending on tracking method, using the GPS data as a reference. While we could not investigate this directly due to a lack of data on spatial reporting bias, we made use of the debiasing algorithm that is included in the ctmm-package [59]. This algorithm weights the contribution of observations to the space use estimate based on their density in the auto-correlation structure of the data. The algorithm should minimise the effect of sampling biases such as those inherent in the neckband observation data, on the resulting space use estimates. Here, we computed two sets of range estimate for every capture site and tracking method: one with the debiasing algorithm (hereafter debiased), and one for which we deliberately excluded the debiasing algorithm (hereafter non-debiased), the result of which should thus inherit location biases from the data. As we expected that effects of bias might differ between the breeding season and the remaining year, we applied this analysis to seasonally restricted rather than to year-round data. Seasons were here defined as “summer”, which included May, June, and July, and “winter”, comprised of November, December, and January. We subsequently computed overlap between these pairs of range estimates using the Bhattacharyya coefficient, and averaged overlap estimates for each season, tracking method, and capture site.

### Effect of sample size on estimates at capture site level

Finally, we considered sample size (i.e. number of tracked individuals) as a factor when comparing coded neckbands and GPS collars, with a particular focus on the number of individuals required to approximate the global means for each method and capture site. Here, we used a cross-validation (leave-out-k) approach when computing model and UD averages. For each tracking method *m* and capture site *p* with sample size of individuals $N_{m,p}$, we randomly sampled $k \in 2,...,(N_{m,p} -1)$ individuals. We estimated the mean Gaussian area based on ctmms for the individuals included in *k*, and determined the deviation, in %, from the global area estimated including all $N_{m,p}$ individuals as $dev_k=(a_k - a_{global} )/a_{global} \times 100$, where *a*_*k*_ indicates the area estimate for a sample of *k* birds, and *a*_*global*_ indicating the area estimate when including all birds tracked from capture site *p* with method *m*. We repeated this process 100 times for each *k*, capture site *p*, and tracking method *m*.

Since both under- and over-estimation can lead to misinformed management, we determined the smallest *k* for which the median of the absolute deviation ($|dev_k|$) would fall below 10%, both per capture site and method, and among all capture sites per method.

## Results

### Comparison of movement metrics and year-round space use

#### Position autocorrelation

**Fig. 2:**
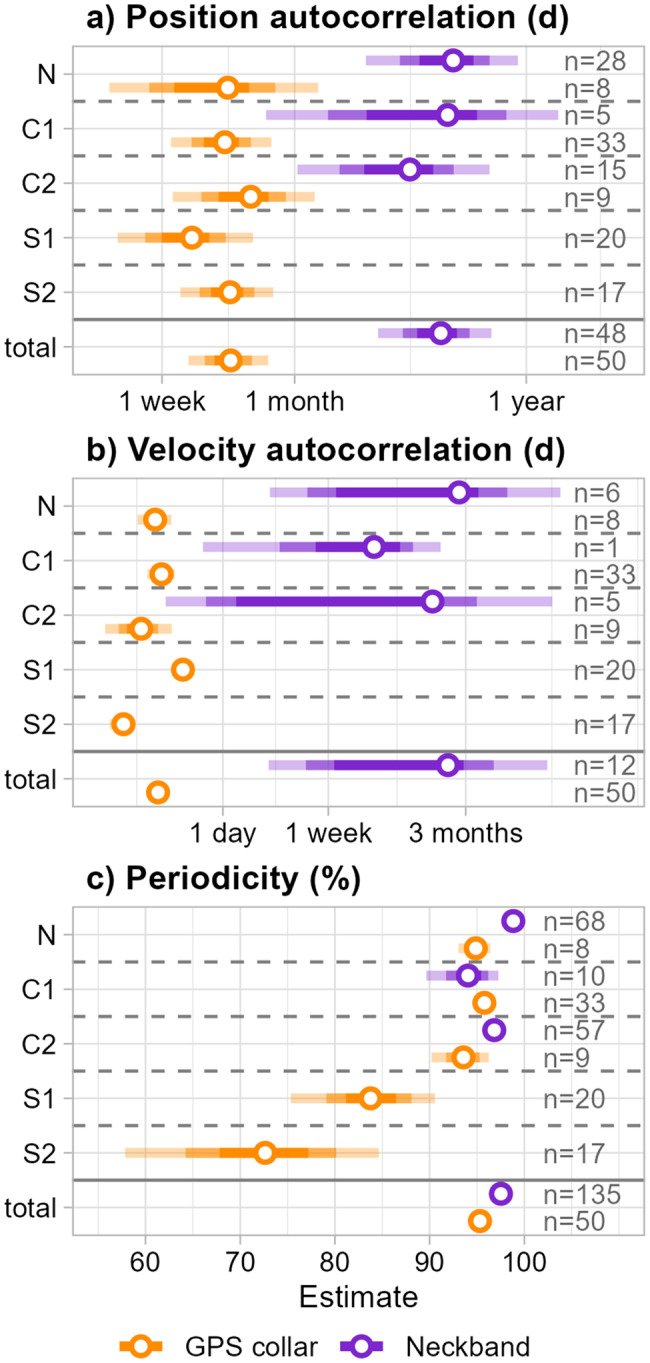
Mean model estimates from the meta-analysis. (**a**) Position autocorrelation (range crossing time, *τ*_*pos*_) (**b**) velocity autocorrelation (behavioural persistence, *τ*_*v*_), and (**c**) periodicity (amount of variation that can be explained by periodic patterns of space use). The plot shows estimates derived from GPS collars (orange) and neckbands (purple) side by side for each capture site. Open circles denote mean estimate, and the 50%, 75%, and 95% CIs are shown as lines with decreasing intensity of shade. Total estimates are based on fully migratory capture sites only (N, C1, C2), and sample sizes are indicated in the figure

Overall, the estimates for position autocorrelation tended to be larger for neckband data than for GPS collar data (Fig. 2a), with position autocorrelation for GPS collar birds measured in weeks, whereas the order of magnitude was months for neckband birds. Across all sites, the mean estimate for *τ*_*pos*_ was 4.89 months, or 144.56 days (95% CI: 73.11–250.13 days) for neckband data, and 14.72 days (95% CI: 9.32–22.2 days) for GPS collar data. This was reflected in the mean estimates per site and method, with mean estimates ranging from 9.69 days (S1) to 18.34 days (C2) for GPS collar data and 103 days (C2) to 165 days (N) for neckband data (see 2a).

#### Velocity autocorrelation

There were considerable discrepancies in mean estimates for *τ*_*v*_ between neckband and GPS collar data (Fig. 2b). Whereas the estimates for GPS collar data were generally on the scale of days, ranging from 0.16 to 0.48 days between capture sites (global mean: 0.30 days, 95% CI: 0.25 - 0.37 days), the estimates for neckband data birds were on the scale of months, ranging from 16.3 days to 78.38 days (global mean: 63.8 days, 95% CI: 2.33 - 398.98 days).

#### Periodicity

The periodicity estimate, indicating the degree to which the mean position of the individual can be explained by the position on the period length, was close to 100% for all of the capture sites with migratory birds, irrespective of tracking method (Fig. 2c). The mean estimate for periodicity in the partially migratory (S1, S2) sites was lowest, with 72.64% and 83.77% for GPS collar birds from S1 and S2, respectively.

In contrast, the periodicity for GPS collar birds from capture sites with migratory birds (C1, C2, N) ranged from 93.57 to 95.78% (global mean: 95.32%, 95% CI: 94.53 - 96.05%); for neckband birds, the mean estimates ranged from 94.05 to 98.84% (global mean: 97.53%, 95% CI: 96.95–98.05%; see also Fig. 2c). Note that these estimates are based on the birds for which the model selection supported at least the 1st harmonic of the suggested period length (1 year), and thus mostly includes individuals tracked for longer than a year. The sample sizes for mean periodicity are thus lower than the overall sample sizes, specifically: S1 - 26 GPS collar birds; S2 - 23 GPS collar birds; C2 - 11 GPS collar birds and 80 neckband birds; C1 - 43 GPS collar birds and 19 neckband birds; N − 8 GPS collar birds and 107 neckband birds.

#### Area of year-round utilisation distributions

**Fig. 3:**
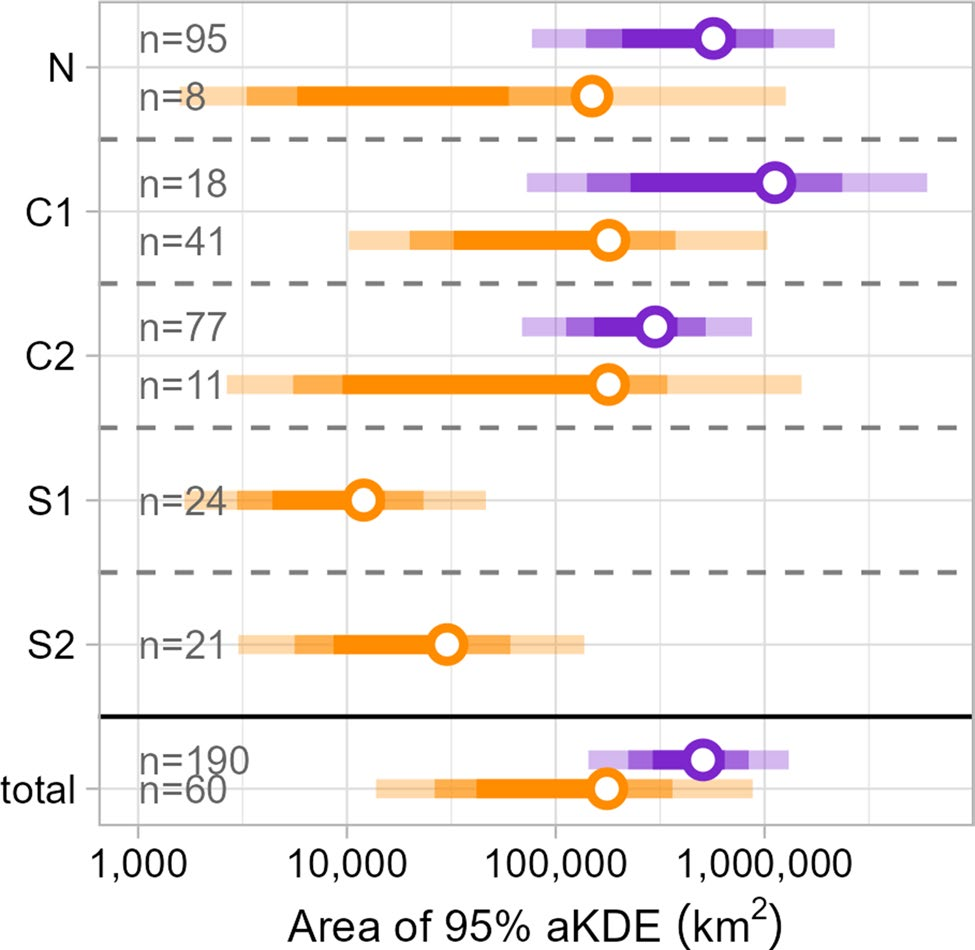
Estimates for the area of capture site-level home ranges shown are the mean (open circles) and 50, 75, and 95% CIs (bars in decreasing opacity) derived from annual aKDEs averaged across all birds per capture site and tracking method. Estimates for GPS collar birds are shown in orange, estimates for neckband birds in purple. Total means are estimated from all individuals from N, C1, and C2 capture sites (see Fig. S2 in the Appendix for individual-level estimates). Sample sizes are indicated in the figure. Note that the x-axis has been log-transformed for increased legibility

Estimates for the area of model-derived migratory ranges (aKDE, full annual cycle) tended to be lower for GPS collar birds than for neckband birds (see Fig. 3), with mean estimates for GPS collar birds of $1.49\times10^5 \mathrm{km}^2$ (95%CI: $0.01\times10^5$ – $12.6\times10^5 \mathrm{km}^2$) for N (*n* = 8), $1.79\times10^5 \mathrm{km}^2$ (95%CI: $0.03\times10^5$ – $15.04\times10^5 \mathrm{km}^2$) for C1 (*n* = 41), and 1.$79\times10^5 \mathrm{km}^2$ (95%CI: $0.1\times10^5$ – $10.3\times10^5 \mathrm{km}^2$) for C2 (*n* = 11). While the overlap of CIs was substantial (see Fig. 3), neckband birds tended to have larger ranges on average, with $5.69\times10^5 \mathrm{km}^2$ (95%CI: $0.76\times10^5$ – $21.681\times10^5 \mathrm{km}^2$) for N (*n* = 95), $11.22\times10^5 \mathrm{km}^2$ (95%CI: $0.73\times10^5$ – $60.13\times10^5 \mathrm{km}^2$) for C1 (*n* = 18), and $2.99\times10^5 \mathrm{km}^2$ (95%CI: $0.69\times10^5$ – $8.71\times10^5 \mathrm{km}^2$) for C2 (*n* = 77). The total averages across birds of all migratory capture sites (N, C1, and C2) differed similarly, with a larger average of $5.07\times10^5 \mathrm{km}^2$ (95%CI: 1.43 – $13.06\times10^5 \mathrm{km}^2$) for neckband birds, and $1.76\times10^5 \mathrm{km}^2$ (95%CI: 0.14 – $8.78\times10^5 \mathrm{km}^2$) for GPS collar birds. Out of all mean estimates for individual neckband birds, 60.4% of them were within the 95% CI for the total average of GPS collar birds, whereas 50% of mean area estimates for GPS collar birds were within the 95% CI of the total average for neckband birds (see Fig. S2).

The remaining two sites, S1 and S2, were estimated to have considerably smaller range sizes due to many of the individuals being resident year-round. Here, only results for the GPS collars are available, with $0.12\times10^5 \mathrm{km}^2$ (95%CI: $0.02\times10^5$ – $0.46\times10^5 \mathrm{km}^2$) for S1 (*n* = 24) and $0.30\times10^5 \mathrm{km}^2$ (95%CI: $0.03\times10^5$ – $1.37\times10^5 \mathrm{km}^2$) for S2 (*n* = 21).

### Congruence of spatial distributions during summer and winter

**Fig. 4:**
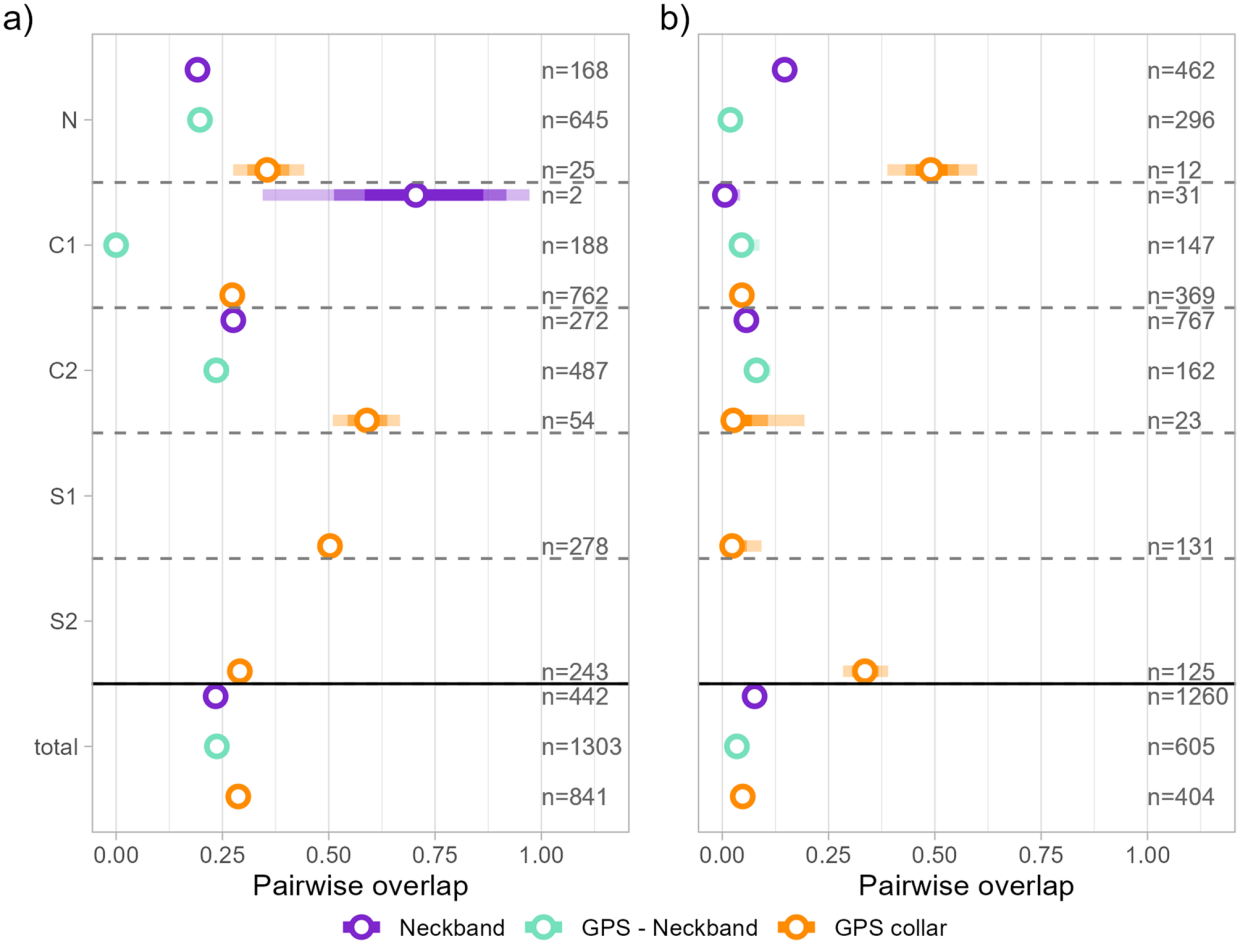
Congruence of home range estimates during a) summer and b) winter. Shown are estimates of overlap, using the Bhattacharyya coefficient, between utilisation distributions for unique pairs of birds that were either tracked using neckbands (purple), GPS collars (orange), and pairs of birds tracked by neckband and by GPS collar (green). Shown are the mean estimates (open circles) and 50, 75, and 95% CIs (bars in decreasing opacity). Total estimates are based on migratory capture sites only (N, C1, C2). Sample sizes, here referring to unique pairs of birds, are indicated in the figure

When estimating pairwise overlap between seasonal range estimates of birds, we found that the overlap between ranges estimated tended to be higher during the summer than during the winter months (see Fig. 4). For capture sites N and C1, the estimated overlap between range pairs was lower for neckband birds than for GPS collar birds (Fig. 4). Pairs of birds tracked using different methods tended to have overlap that was similar to the levels of pairs of birds tracked using the same method, with the exceptions including capture site C1 during the summer months, for which only few neckband birds were available (*n* = 7), and C2 during the same season, with overlap between GPS collar birds substantially higher than for other comparisons. When considering all pairwise overlaps per season and method for the three northernmost capture sites N, C1 and C2 this picture persisted: during the summer months (Fig. 4a), comparison between GPS collar birds had an average overlap of 0.29 (95% CI: 0.27–0.31), distribution ranges of neckband birds had an average overlap of 0.23 (95% CI: 0.22–0.25), and the overlap between tracking methods was intermediate with 0.24 (95% CI: 0.22–0.25). During winter, overlap was considerably lower across all comparisons (Fig. 4). Pairs of GPS collar birds had an average overlap of 0.05 (95% CI: 0.03–0.07). For neckband birds, the average was 0.08 (95% CI: 0.07–0.08), and the overlap between tracking methods was 0.03 (95% CI: 0.02–0.05; Fig. 4b).

### Estimating effect of location biases on range estimates

**Fig. 5:**
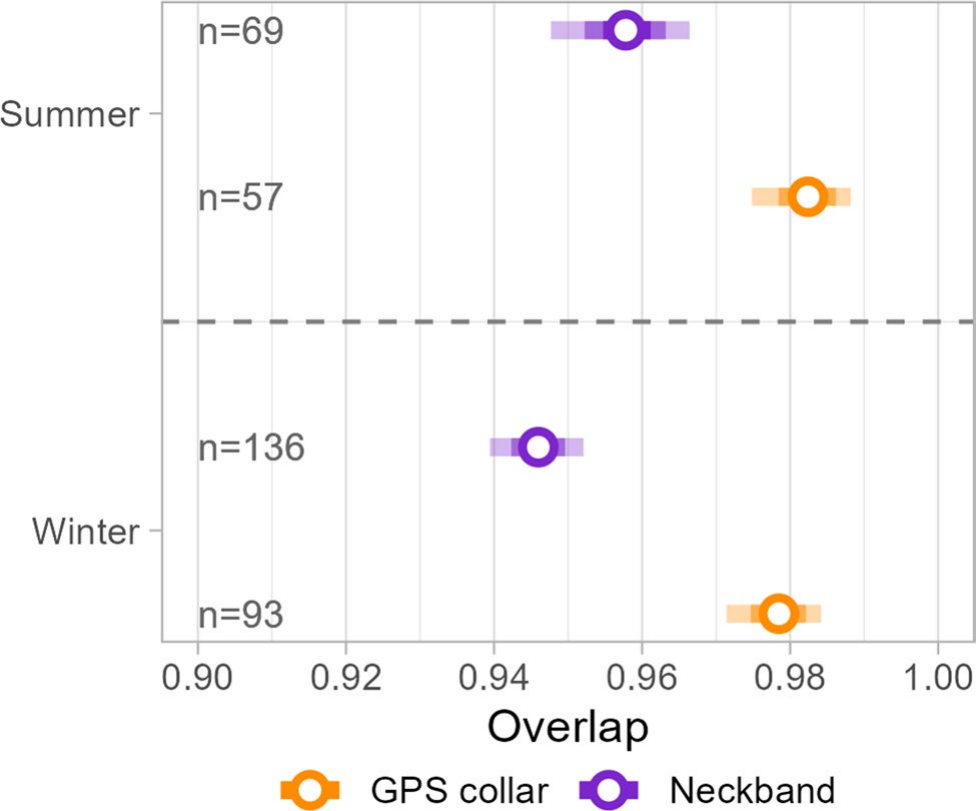
Overlap between debiased and non-debiased UD estimates. overlap between aKDEs computed for the same individual and time-period, with and without use of the debiasing algorithm. Shown are the estimated mean overlap between debiased and non-debiased aKDEs for summer and winter for all birds from the migratory populations, with mean estimates represented by open circles and the respective 50, 75, and 95% CIs indicated by bars of decreasing opacity. Sample sizes are indicated in the figure

We found that the overlap between debiased and non-debiased space use estimates of the same individual was, albeit high for both tracking methods (see Fig. 5), consistently higher for GPS collar birds (summer: mean 0.98, 95% CI: 0.97–0.99; winter: mean 0.98, 95% CI: 0.97–0.98) than in neckband birds (summer: mean 0.96, 95% CI: 0.95–0.97; winter: mean 0.95, 95% CI: 0.94–0.95).

### Effect of sample size on estimates at capture site level

**Fig. 6:**
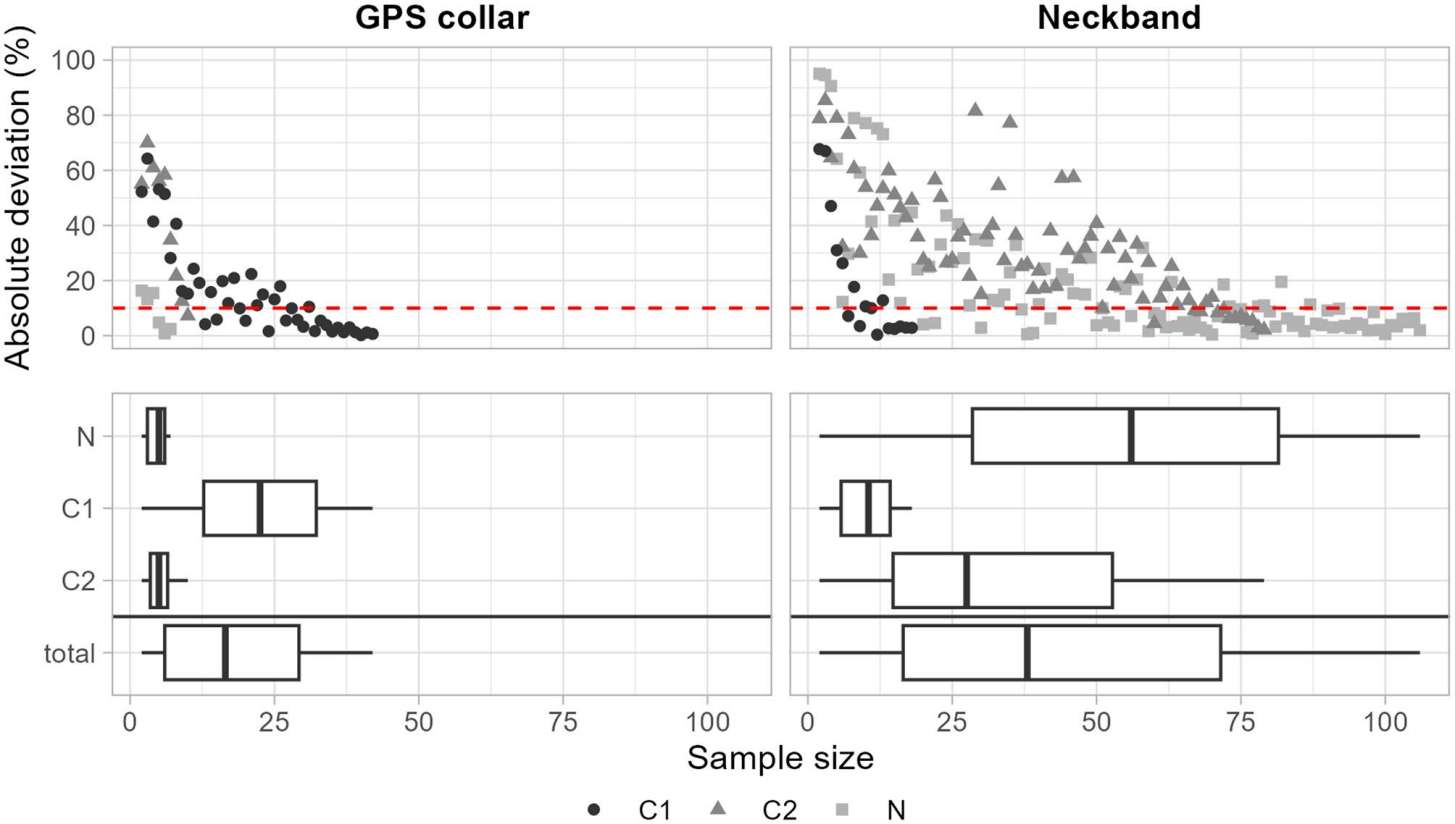
Effect of sample size on mean area estimates. The top row plots show the absolute deviation $|dev_k|$ (mean from k-fold cross-validation) from the total mean estimate for GPS collar birds (left) and neckband birds (right). The red dashed line indicates an absolute deviation of 10%. The bottom row shows for which sample sizes the absolute deviation $|dev_k|$ was equal to, or less than 10%. Boxes show the 25%, 50%, and 75% quartiles, and whiskers indicate 1.5 times the interquartile range

The cross-validation for the mean area estimates from model meta-analysis suggested that even with small numbers of individuals contributing to the meta-analysis, the estimated mean quickly approximates the global mean derived from all neckband and GPS collar birds from each site (see Fig. 6 and S3). For GPS collar birds, a sample size of 17 birds for determining average space use was sufficient for the median of deviation from the global mean $|dev_k|$ to drop below 10% (N: 5 birds; C1: 23 birds; C2: 5 birds). For neckband birds, the required sample size to achieve the same deviation from the global mean was higher, with 37 birds needed when considering all capture sites (N: 55 birds; C1: 11 birds; C2: 27.5 birds). Overall, we found the number of birds needed to approach the global mean to be higher for groups for which the overall sample size was higher.

## Discussion

Our study reveals that despite large differences in sample size and temporal resolution, the analysis of tracks of migratory greylag geese collected using coded neckbands and GPS collars resulted in estimates of year-round space use that were similar irrespective of method. While overall, the mean estimate of space use for neckband birds was larger than for GPS collar birds, most individual estimates for both methods were within the 95% CI of the global GPS collar mean (see Fig. S2). The predominantly resident birds captured at S1 and S2 and marked with neckbands were not sufficiently reported to allow for them to be included in a comparison between GPS collar and neckband birds. Despite this local gap in reporting, neckband observers reporting to CR Birding were able to capture the year-round movements of the migratory study population. While biases in the location data were slightly higher for birds tracked with neckbands than with GPS collars, this did not negatively affect the possibility of estimating the spatial distribution of migratory greylag geese.

The continuous-time movement process as derived from regular GPS data could however not be replicated from the neckband data, which might lead one to conclude that coded neckbands and GPS collars clearly offer completely different interpretations of goose movement behaviour: Average crossing time for full annual ranges, as indicated by position autocorrelation *τ*_*pos*_, was consistently lower for GPS collar birds, with an average of about two weeks, as opposed to neckband birds, for which mean estimates ranged from three to six months. This latter estimate would severely overestimate the amount of time that is required for geese to complete their migration. While this discrepancy between methods does not necessarily affect any other conclusions drawn from the data, sightings of birds with coded neckband included in this study suggest that neckband data lack sufficient temporal resolution to reliably estimate migration speed. Upon inspection of periodograms derived from GPS collar data, we found that periodicity likely occurs at different time scales, with a strong case for including daily as well as annual periodicity in a ctmm. Not taking this into account likely contributed to the differences of temporal scale, which was estimated for *τ*_*pos*_, and might even detract from the ability to explain the mean position to the same degree as for neckband birds. Similarly, behavioural persistence, as approximated by velocity autocorrelation *τ*_*v*_, highlighted the difference in resolution of data generated by the two tracking methods: invariably, mean estimates were < 24 hours for GPS collar birds from all capture sites, whereas the overall average for neckband birds was about 64 days. Additionally, only very few neckband birds had sufficiently many observations to support the estimation of this parameter during the model selection process at all (4%), whereas nearly 50% of selected models for GPS collar birds included both position and velocity autocorrelation. This does not necessarily mean that the available estimates of *τ*_*v*_ for neckband birds are irrelevant, but they appear to measure movement behaviour at a very different temporal scale than *τ*_*v*_ estimated for the GPS collar birds. Whereas the timescale for the former suggests that *τ*_*v*_ measured changes in life-history stages for neckband birds, the higher sampling frequency for GPS collar birds supported the estimation of behavioural changes at a daily scale. Both are important, but their usefulness depends on the purpose of the study. Hypothetically, reducing the temporal resolution of the GPS data further might provide behavioural changes on a scale more similar to those of neckband birds, while the original resolution would allow for an even finer scale. It was surprising that this was the case even though we evaluated semi-variance only at daily intervals, and consequently temporal scale of the behaviour of interest should be considered prior to applying ctmms to data. The only model parameter that appeared not to be influenced by sampling frequency was periodicity, interpreted as the amount of variation in the mean position of birds that can be explained by the annual cycle. Here, all migratory study sites irrespective of tracking method had consistently high estimates of around 95%, suggesting that regularity and consistency of migratory behaviour can be estimated equally well using either tracking method. Thus, our study suggests that movement metrics derived from the data other than space use differed considerably, likely due to a mismatch in temporal resolution, and only the general regularity of seasonal migration was captured equally by either method.

While conclusions about the continuous-time movement process as estimated by the ctmms clearly depended on sampling frequency, this was not the case for the spatial range distributions derived from the models and tracking data. While uncertainty around mean estimates was high for estimates at capture site level for neckband birds, possibly due to low effective sample sizes for aKDES derived from small numbers of observations, the total means considering all birds captured at C1, C2, and N were on the same order of magnitude for both tracking methods, and confidence intervals for the global mean area for capture sites N, C1, and C2 overlapped to a large degree. Overall, estimated mean area at the capture site level was higher for neckband birds by approximately a factor three. However, when considering the individual level, we found that the majority of area estimates for neckband birds (60.4%) were within the 95% confidence interval of the global GPS collar mean, but only 17.1% of these estimates were within the 95% confidence interval of the global neckband mean (see Fig. S2). While this might appear counter-intuitive, we think this discrepancy in global means is likely a consequence of stringency when selecting individuals for computing global estimates. The threshold we used for including area estimates in computing population-levels means was low (effective sample size > 1), and a more stringent selection of UDs with a greater underlying degree of freedom indeed decreases the global mean for neckband birds to be more in line with GPS collar birds: when using a threshold of effective sample size > 10, the global estimate for neckband birds would be in line with that of GPS collar birds (mean $1.52 \mathrm{km}^2$, 95% CI: 0.34 – $1.93 \mathrm{km}^2$, based on *n* = 49 birds). Consequently, we conclude that overall, range estimates derived from neckband and GPS collar birds behaved as if drawn from the same statistical population. This was also indicated by our investigation of the spatial congruence of range estimates. Here, we found that even though overlap between neckband birds tended to be somewhat higher than in GPS collar birds, pairwise distance derived from GPS collar – neckband pairings tended to be intermediate, and especially in winter estimates were very similar irrespective of the comparison made.

Finally, we evaluated the effect of potential sampling biases. We had no information about reporter effort or density, discouraging any analyses investigating the direct effects of spatial reporting bias on range estimates. Instead, we approached reporting bias using a coarser comparison between the summer months, when birds were on the breeding grounds in Sweden, with the winter months, during which birds were more widely distributed throughout Europe, and used the optional debiasing algorithm in the aKDE computation as a proxy. We further relied on the data collected via GPS collars to provide a baseline measurement of discrepancy between range estimates estimated either with or without the debiasing algorithm. Using the GPS collar results as a baseline assumes that the collection of GPS data is indeed unbiased during both the summer and winter months. Solar-powered GPS collars as used in this study tend to perform better under conditions with more daylight hours, suggesting that our baseline values might violate that assumption of observations being equallly likely during summer and winter. However, we found no difference comparing range estimate pairs for GPS collar birds during either summer or winter, and we thus proceeded by interpreting the results for neckband birds relative to the estimates for GPS collar birds. Overlap between aKDEs that have been, and have not been debiased, was lower for neckband birds than for GPS collar birds and it was marginally lower during winter as compared to the summer months. Consequently, our results suggest there is a greater bias in data collected using coded neckbands, and that for the studied capture sites, this bias tended to be larger during the winter months when birds are spread over a larger area than during the breeding season. Average overlap did however exceed a value of 0.9 irrespective of capture site and season for neckband birds, suggesting that at least for the capture sites with migratory individuals, any effects of reporting biases in the study area do not dramatically alter estimated population-level ranges.

### Conclusions

Both GPS collars and coded neckbands generate location histories for known individuals from which we can deduce aspects about the area, timetable, and habitat an individual occupies, as well as how it moves within this area. For a (partially) migratory species like greylag goose, knowledge derived from location histories may include migratory flyways and their connectivity, speed of migration, site fidelity to locations used over the annual cycle, timing of life history stages, and more. The processes that generate these data are, however, very different, and each come with their own set of confounding factors: CMR methods rely on human effort, e.g. observer density and effort for coded neckbands, and are thus frequently biased temporally and/or spatially (for citizen science, e.g. [61]); and data are often sparse and irregular for an individual bird. In contrast, GPS devices, while having decreased exponentially in cost per collected location over the last decades, can still be prohibitively expensive to deploy, often leading to relatively smaller numbers of individuals being marked compared to CMR methods. Furthermore, while GPS devices are becoming increasingly lighter, this does not necessarily lead to lower relative loads being deployed on individuals [62]. Effects of tags and their attachment methods on survival and behaviour of birds are still not fully understood; studies on geese increasingly point towards reduced survival, increased divorce rate, and delays in migration [25, 62–66]. What is less clear currently is whether the adverse effects of tags might also lead to changes in space use, though an increase in energy expenditure induced by added weight and drag (e.g., [67, 68]) might affect migratory decision-making. While we cannot entirely exclude the possibility that potential biases of both methods converge in such a way that any true differences from the methods cancel out, we consider this an unlikely event.

In summary, our results indicate that for greylag geese originating from the five studied Swedish capture sites [37, 40], both neckband resightings and GPS tracking provide a mutually consistent picture of the spatial distribution of birds during both summer and winter months. This pattern is similar to what has been demonstrated for Bewick’s swans (*Cygnus columbianus bewickii*) wintering in Northwestern Europe [69]. While the data did not include a sufficiently large sample of migratory movements per individual to confirm spatial congruence throughout the annual cycle, we expect that this would indeed be the likely outcome given the near-equivalent estimates for distribution ranges between the two methods overall, at least at the level of the capture site. However, this comparability between methods is likely conditional on study area, particularly reporting effort, and has not been tested outside of the areas used by the geese breeding at our capture sites. In areas lacking a culture of reporting neckband observations, remote tracking through GPS collars or similar methods would be essential. Any inferences about the movement process rather than the areas occupied throughout the annual cycle, however, are likely to result in discrepancies between methods. While equivalence can likely be achieved by coarsening the GPS collar data to match average temporal resolution of the neckband data, the differences in temporal resolution in this study led to models estimating behaviour at different temporal scales. Given the low number of reported sightings of neckband birds from capture sites S1 and S2 and our resulting inability to estimate movement variables and distribution ranges, we must conclude that our overall outcome and conclusion are conditional on birds being migratory. Birds from S1 and S2 overall had a lower tendency to migrate, with 26 out 31 GPS collar birds from S1, and 14 out of 24 GPS collar birds from S2 never observed $> 150\,\mathrm{km}$ from their capture site. With geese in the southern parts of Sweden increasingly showing signs of short-stopping their migrations [70, 71], particularly during mild winters, changes in migration habits of Swedish geese are expected to happen. Our results suggest that, if tendency to report neckband birds in the southern parts of Sweden were to increase, these changes can be monitored and mapped using either method. Looking beyond the limitations of our study system, our study shows that leveraging the strengths of both tracking methods - long-term data availability and often large sample sizes in the case of CMR and telemetry methods with low temporal resolution, and the high level of detail of modern GPS-tracking - using approaches such as ctmm can open up possibilities for investigating large scale movements, including variation both within and among populations, and changes in migratory behaviour over long time periods.

## Electronic supplementary material

Below is the link to the electronic supplementary material.

Supplementary Material 1

Supplementary Material 2

## Data Availability

The data that are necessary for replicating the analyses, including neckband observations, GPS tracking data, and capture information about individuals are available on zenodo under 10.5281/zenodo.17817498. Annotated R-code used to conduct the analyses for this study can be found in Supplementary Material 2.
