# Supplementary material for: Do GPS collars and coded neckbands tell the same story about year-round movements in geese?
Source: Mov Ecol. 2026 Jan 12;14:3. doi: 10.1186/s40462-025-00620-y (PMC12829013; doi:10.1186/s40462-025-00620-y)
Supplement: Supplementary file 1 — Supplementary Material 1 [file 40462_2025_620_MOESM1_ESM.pdf]

Do GPS collars and coded neckbands tell the same story about year-round movements in geese? - Appendix A

Mariëlle L. van Toor[1], Christen H. Fleming[2,3], Niklas Liljebäck[4], Johan Månsson[4], Jonas Waldenström[1], Johan Elmberg[5]

[1]Centre for Ecology and Evolution in Microbial Model Systems, Linnaeus University, Kalmar, Sweden.
[2]Department of Biology, University of Central Florida, Orlando, Florida, USA.
[3]Smithsonian Conservation Biology Institute, Front Royal, Virginia, USA.
[4]Grimsö Wildlife Research Station, Department of Ecology, Swedish University of Agricultural Sciences, Riddarhyttan, Sweden.
[5]Department of Environmental Science, Kristianstad University, Kristianstad, Sweden.

Contributing authors: marielle.vantoor@lnu.se; Christen.Fleming@ucf.edu; niklas.liljeback@jagareforbundet.se; johan.mansson@slu.se; jonas.waldenstrom@lnu.se; johan.elmberg@hkr.se;

Additional Figures

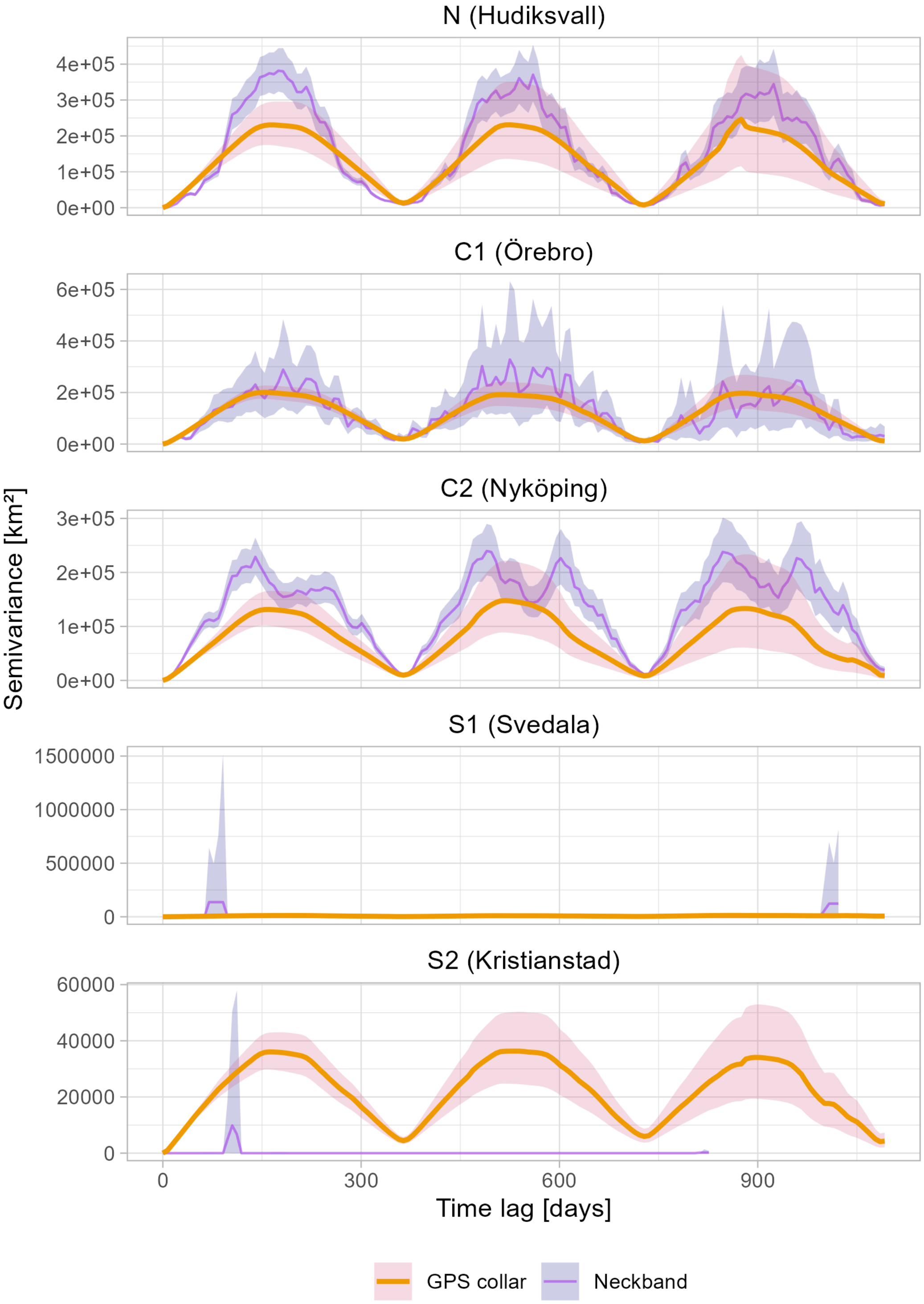

Fig. S1 Mean semivariograms for each capture site and method. Shown are the mean (solid lines) and 95% confidence intervals (shaded areas). Variograms were computed with a timelag of seven days for this figure for legibility.

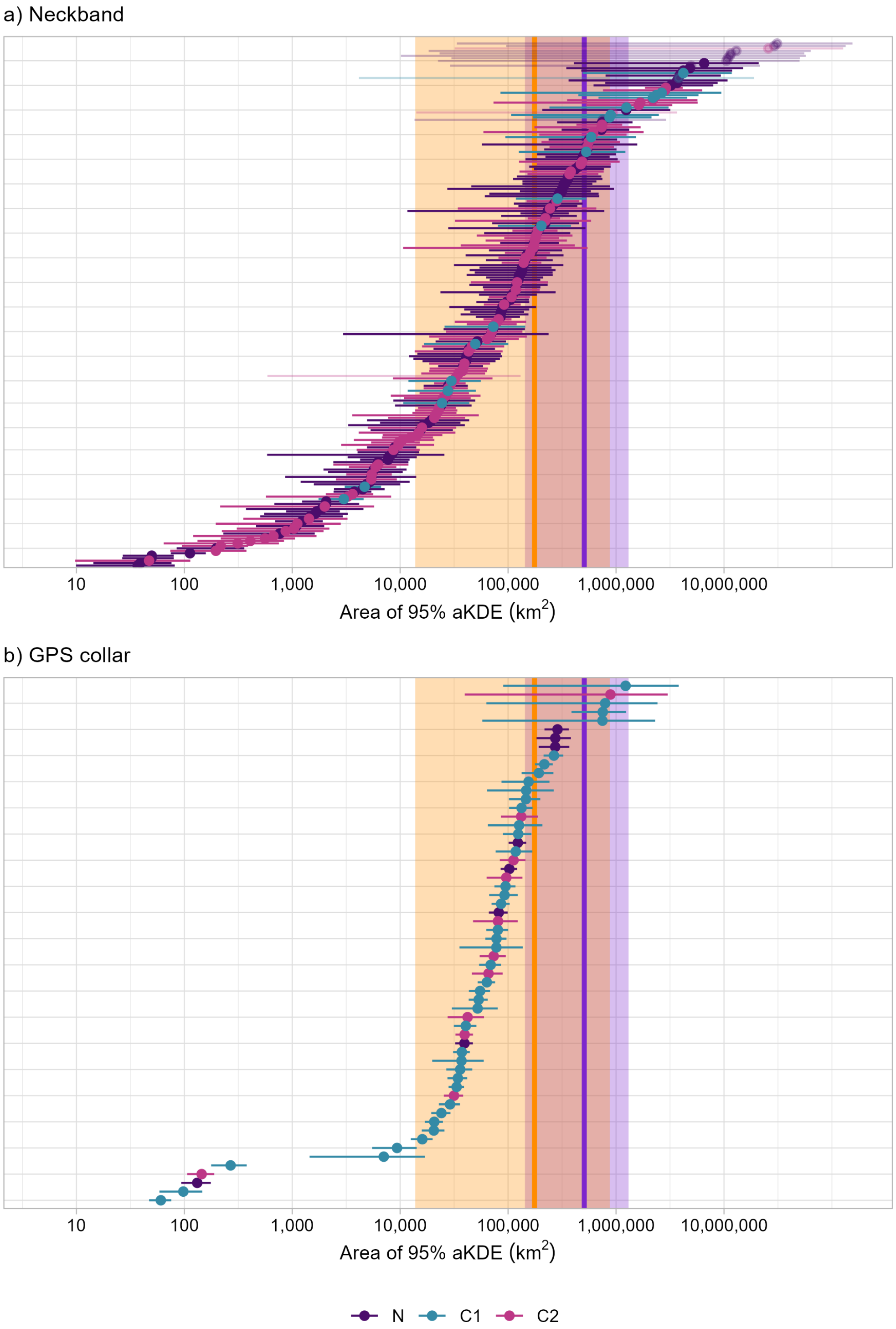

Fig. S2 Individual-level mean estimates for year-round space use. Shown are the mean and 95% confidence interval of each individual for which a computing the respective aKDE from tracking data and ctmm was successful. Individuals included in the estimation of global means are shown in opaque colours, whereas individuals with a effective sample size ≤ 1 are transparent. Vertical lines show the estimates for the global means of both neckband birds (purple) and GPS collar birds (orange), 95% confidence interval on the mean are indicated by transparent rectangles.

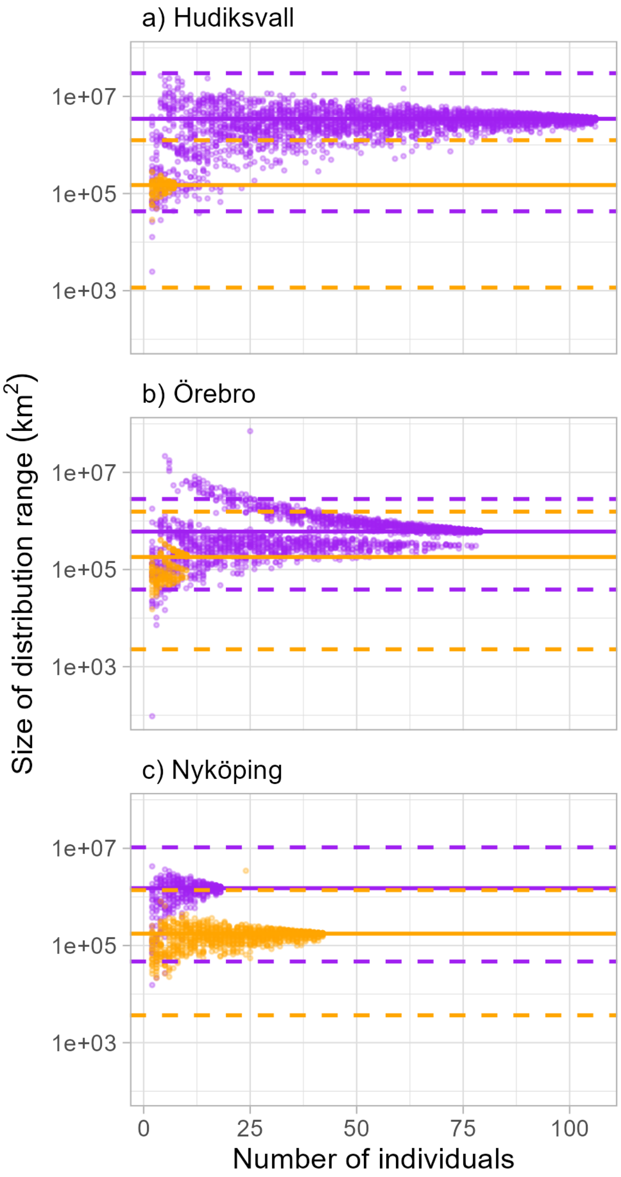

Fig. S3 Effect of sample size on mean estimates of space use. The figure shows mean space use estimated during the k-fold cross-validation. Each point represents the average estimate for model-based (i.e. Gaussian) space use for every replicate and sample size. The overall mean for all individuals per capture site and method is shown as a solid horizontal line, whereas dashed lines reflect the upper and lower bounds of the 95% confidence interval for the overall mean.

Additional Tables

Table S1 Overview over reports for neckband birds. This includes all the birds marked with neckband in the study, and all reports collected over the entire study period (June 2017 – January 2023). The numbers reflect the number of all reports for individuals included in the study, prior to any filtering steps. Note that we here defined summer (May-July) and winter (November - January) in the same way as in the main manuscript.

Capture site	Marked birds	Total reports (mean per bird)	Summer reports (mean per bird)	Winter reports (mean per bird)
Hudiksvall	263	4,266 (16.2)	2,038 (7.7)	780 (3.0)
Örebro	236	798 (3.4)	265 (1.1)	208 (0.9)
Nyköping	181	3,104 (17.1)	669 (3.7)	869 (4.8)
Svedala	83	49 (0.6)	8 (0.1)	24 (0.3)
Kristianstad	58	50 (0.9)	25 (0.9)	15 (0.3)
**total**	821	8,267 (10.1)	3,005 (3.7)	1,896 (2.3)
