# Supplementary material for: Do GPS collars and coded neckbands tell the same story about year-round movements in geese?
Source: Mov Ecol. 2026 Jan 12;14:3. doi: 10.1186/s40462-025-00620-y (PMC12829013; doi:10.1186/s40462-025-00620-y)
Supplement: Supplementary file 2 — Supplementary Material 2 [file 40462_2025_620_MOESM2_ESM.zip › Appendix_B_code/tracking_method_comparison_ANNOTATED_CODE.html]

Do GPS collars and coded neckbands tell the same story about year-round movements in geese? (Annotated code)


## Table of contents

- Note
- Session set-up
- Data overview and preparation
  - Master sheet
  - Study site map
  - Neckband data
    - Data import
    - Data pre-processing
  - GPS tracks
    - Data import & cleaning
  - Censoring of locations biased by family members
    - Censoring of data collected by juveniles
    - Censoring of data collected by partners
    - Comments from geese.org reports
    - Pairwise distance of GPS-tracked birds
  - Export of neckband observations, GPS tracking data, and metadata on individuals for upload on zenodo
  - Data overview
    - Sample sizes
    - Summary figures
    - Map
- Continuous-time movement models
  - ctmm - Neckband data
    - Model fitting and selection
    - Model checks
    - Parameter estimates for reporting in the manuscript
  - ctmm - GPS data
    - Model fitting and selection
    - Model checks
    - Total averages for migratory sites
    - Parameter estimates for reporting in the manuscript
    - Combine results & plot
- Range estimation
  - Complete annual ranges
    - Prepare grid
    - Range estimation - GPS data
    - Range estimation - Neckband data
    - Average migratory range *areas*
    - Population-level kernel density estimates
- Congruence of full annual range estimates of neckbands and GPS collars
- Congruence of winter and summer range estimates of neckbands and GPS collars
  - Fit sub-annual models & compute aKDEs
    - Summer, GPS collars
    - Summer, Neckbands
    - Winter, GPS collars
    - Winter, Neckbands
  - Estimate pairwise overlap
    - Overlap between GPS collared birds in the same location
    - Overlap between neckband birds in the same location
    - Overlap between GPS and neckband birds in the same location
    - Plot
  - Estimate population-level overlap
    - Summer
    - Winter
- Congruence between debiased and non-debiased UDs
  - GPS
    - Summer
    - Winter
  - Neckbands
    - Summer
    - Winter
  - Results & Plot
- Sensitivity to sample size
- Tendency to migrate
- Session info

# Do GPS collars and coded neckbands tell the same story about year-round movements in geese? (Annotated code)

Author

Mariëlle van Toor

# Note

The document below includes code and additional figures for all the analyses conducted for the study, and is meant to allow for the replication of the study. The document also includes most of the preparation of data for analyses, which at the end of the preparation stage are exported - the product of which is the data that are shared alongside the study. If you want to re-run the analyses by yourself, you will need to start at section @data-import. You will also need to rename some of the column headers (which were changed for the data export so as to be more intuitive and consistent across data sets).

In the document, you will see text with explanations, tables, and figures. Code is folded by default, and you can show chunks of code by clicking on the little arrows you will find throughout the document. They look like this:

Example for a code chunk. Click on the arrow to fold out the code.

```
# No code in this chunk as this is just an example.
```

> This document depends on an additional file containing some functions which make pulling parameter estimates from models a bit more easy. This file (get\_par\_ctmm.r) is included in the .zip-file.

---

# Session set-up

Loading of required packages, definition of some CRS strings, and some other bits that will be required throughout the dodument.

```
#################################################
# packages

## data handling
library(plyr)
library(lubridate)

## spatial libraries
library(sf)
library(geosphere)
library(sp)
library(suntools)
library(terra)

## visualisation
library(ggplot2)
library(viridisLite)
library(patchwork)
library(tidyterra)

## continuous-time movement models
library(ctmm)

#################################################
## projection statements

proj.ll <- '+proj=longlat +datum=WGS84'
proj.se <- '' # Swedish coordinate reference system
proj.tpq <- '' # two-point equidistant projection, specific to the data set
# oblique Lambert azimuthal equal area projection
proj.olaea <- '+proj=laea +lon_0=9.0966797 +lat_0=53.7604986 +datum=WGS84 +units=m +no_defs'


#################################################
## locations
all.sites <- c('Svedala', 'Kristianstad', 'Hudiksvall', 'Örebro', 'Nyköping')
nb.sites <- c('Hudiksvall', 'Örebro', 'Nyköping')

#################################################
## some spatial data for visualisation
adm <- st_read('ne_10m_admin_0_countries/', quiet=T)

#################################################
## some functions for pulling parameter estimates out of averaged ctmm models
source('get_par_ctmm.r')

#################################################
## set seed

set.seed(38567221)

# theme for figures
theme_geese <- theme_light() + 
  theme(legend.title=element_text(color = "#000000", size=10),
        legend.text=element_text(color = "#000000", size=9),
        axis.text=element_text(color = "#000000", size=9),
        axis.title=element_text(color = "#000000", size=10),
        plot.title=element_text(color = "#000000", size=11),
        strip.text=element_text(colour='#000000', face='bold', size=11, hjust=0),
        strip.background=element_rect(fill='white'),
        legend.background = element_rect(fill = 'white', color = NA), 
        legend.key=element_rect(fill='white', colour=NA))

# function for catching print statements while compiling the document (purely aesthetic)
suppress_output <- function(expr) {
  capture.output(result <- eval(expr), file = NULL)
  invisible(result)
}
```

---

# Data overview and preparation

## Master sheet

The master sheet contains all capture and deployment information for birds in this study. It includes information on capture location and date, information about the individual (age, sex, bodymass), and a number of identifiers that will be crucial to connect information in the CMR/tracking data with the relevant information (number on the tarsal steel band, neckband code, transmitter ID where relevant).

Import of the master sheet. This data set contains about time and place of capture of all geese, individual and neckband/GPS collar information, along with sex, age at capture, and some measurements.

```
# import master sheet
load('data/tracking_data/reference_data.RData')

# make sure that identifiers match with how they are listed in the tracking data
inf$individual.local.identifier <- ifelse(inf$population=='Hudiksvall', 
                                          paste(inf$neckband.code, 'white', 'Sweden', sep='_'), 
                                          inf$steel.ringnumber)

# rename population in Kvismaren as Örebro, in line with previous publications
inf$population[inf$population=='Kvismaren'] <- 'Örebro'
```

## Study site map

Code

```
locs <- ddply(inf, 'population', function(x){
  data.frame(x=x$long[1], y=x$lat[1], n=sum(x$capture.status=='new'))
})
locs$x.label <- locs$x + c(0, 7, 5, -5, -7)
locs$y.label <- locs$y + c(2, rep(0,4))

range.x <- st_bbox(adm[adm$NAME=='Sweden',])[c(1,3)]+c(-7,7)
range.y <- st_bbox(adm[adm$NAME=='Sweden',])[c(2,4)]+c(-1,1)

theme_map <- theme_light() +
  theme(axis.title=element_blank(), 
        axis.text=element_blank(),
        axis.ticks=element_blank(),
        panel.grid=element_blank(),
        text = element_text(color = "#22211d"), 
        legend.title=element_text(color = "#22211d", size=10),
        legend.text=element_text(color = "#22211d", size=9),
        plot.background = element_rect(fill = 'white', color = NA), 
        panel.background = element_rect(fill = '#dcebf0', color = NA), 
        legend.background = element_rect(fill = NA, color = NA), ##f5f5f2
        strip.background = element_rect(fill='white'),
        strip.text=element_text(colour='black', face='bold'),
        panel.border = element_blank())

ggplot() +
  geom_sf(data=adm, fill='antiquewhite', colour='grey20', linewidth=0.2) +
  geom_segment(data=locs, aes(x=x, y=y, xend=x.label, yend=y.label)) +
  geom_point(data=locs, aes(x=x, y=y), shape=21, colour='black', fill='white', stroke=2, size=3) +
  geom_label(data=locs, aes(x=x.label, y=y.label, label=population), label.r = unit(0, 'lines'), label.size=0) +
  theme_map +
  scale_colour_viridis_d(name='', end=0.5, option='C', direction=-1) +
  guides(colour=guide_legend(override.aes = list(size=2.5, alpha=1))) +
  xlim(range.x) +
  ylim(range.y) +
  theme(legend.position='none')
```

Map of Scandinavia showing the location of different capture sites. The map is shown in plain geographic coordinates.

## Neckband data

### Data import

By default, this data set contains a number of observations of different species, and is otherwise not pre-processed. Here I’m only retaining observations of greylag geese fulfilling the following criteria:

- sightings earliest in 2017 (when birds were first captured for this project)
- neckband code is present in master sheet + 1 individual that had its ring misspelled (091 instead of O91)
- only observations up until an individual is reported dead (not marked as L or empty)
- observations that do not assume excessive speed (all observations have a maximum speed of 315km/day, so this filter does not actually do anything)

Read the neckband observations from a .csv-file downloaded from geese.org. Filter the data so it only contains observations from greylag geese, and convert the date information into Date-format.

```
df <- read.delim('data/neckband_data/download-obs (1).csv', header=T, sep=',')

unique(df$EURING)

species.code <- data.frame(
  euring=c(1520, 1570, 1600, 1610, 1660),
  species=c('Cygnus olor', 'Anser fabalis/serrirostris', 'Anser erythropus', 
            'Anser anser', 'Branta candensis'))

# retain only greylag geese
df <- df[df$EURING==species.code$euring[species.code$species=='Anser anser'],]

# convert to date format
df$date <- as.Date(unlist(lapply(strsplit(df$OBS_DATE, '\\/'), function(d){
  paste(d[3], d[2], d[1], sep='-')
})))
```

### Data pre-processing

The following code chunk contains some cleaning steps, each of which is commented with the purpose of each step. Here we also merge the CMR data with the master sheet so all information are available in a single table.

```
# exclude sightings prior to 2017
df <- df[year(df$date)>=2017,]

# only birds ringed as part of the project
# load('data/tracking_data/reference_data.RData')

missing <- sort(unique(df$NECK_BAND[which(!df$NECK_BAND %in% inf$neckband.code)]))
available <- sort(inf$neckband.code)

# neckbands starting with O might have been entered as 0 - it seems there is only one
missing[grep('0', missing)] 
df$NECK_BAND[df$NECK_BAND=='091'] <- 'O91'

df <- df[df$NECK_BAND %in% inf$neckband.code,]

# merge with master sheet
df$neckband.code <- df$NECK_BAND
df <- merge(df, inf[,c('capture.date', 'neckband.code', 'age', 'sex', 
                       'bodymass', 'tarsus', 'population', 'transmitter', 
                       'transmitter.id', 'steel.ringnumber')], all.x=T)

# do, for each individual:
# no observations after reported dead
# no observations before capture
df <- ddply(df, 'NECK_BAND', function(x){
  x <- x[with(x, order(date)),]
  x <- x[x$date>=x$capture.date,]
  if(!all(x$ALIVE_DEAD %in% c('', 'L'))){
    x <- x[1:(which(!x$ALIVE_DEAD %in% c('', 'L'))[1]),]
  }
  return(x)
})

# Calculate speed in km/day for eventual application of speed filter
df$julian <- as.numeric(strftime(df$date, format='%j'))

df <- ddply(df, 'NECK_BAND', function(x){
  x$tdiff <- abs(c(diff(as.numeric(x$date)),NA))*24
  x$distance <- distGeo(x[,c('LONGITUDE', 'LATITUDE')])/1000
  return(x)
})

df$speed <- NA
df$speed[complete.cases(df[,c('tdiff', 'distance')])] <- 
  df$distance[complete.cases(df[,c('tdiff', 'distance')])]/
  df$tdiff[complete.cases(df[,c('tdiff', 'distance')])]
df$speed[!is.na(df$tdiff) & df$tdiff==0] <- 
  df$distance[!is.na(df$tdiff) & df$tdiff==0]
## no speed filter is necessary as the maximum observed speed is 315 km/day which is well within line with what to expect

# save for easy access
save(df, file='data/daily/240828_neckband_data.RData')
```

## GPS tracks

### Data import & cleaning

The tracking data were collected using GPS transmitters in the form of solar-powered neckbands. Tags from two different manufacturers were used in the project, and as a consequence of that, the data were stored in slightly different ways. Therefore, we need to import the data in two different ways, and make sure that the naming conventions and available sensory information match up.

> Note: While it is not technically necessary to thin the GPS trajectories to ensure comparability between neckband reports and GPS tracks using continuous-time movment models, we will thin the GPS trajectories to daily relocations. This thinning is done only for the reason of making the computations feasible, but the results should be robust against changes in temporal resolution.

#### Population Hudiksvall

The birds captured in Hudiksvall were deployed with made-by-theo tags. Data were accessed from Movebank (study name: LifeTrack Greylag Scandinavia.csv).

Read tracking data of Hudiksvall birds from .csv-file downloaded from Movebank.

```
# import of individual tracking data
df <- read.delim(header=T, sep=',',
                 file='data/tracking_data/tracks_madebytheo/LifeTrack Greylag Scandinavia.csv')
df <- df[df$tag.local.identifier %in% 
           gsub('ID', '', inf$transmitter.id[inf$population=='Hudiksvall']),]

df$timestamp <- as.POSIXct(df$timestamp, tz='UTC')

# remove individual captured in Norway
df <- df[df$individual.local.identifier!='D27_white_Norway',]
```

We will be able to avoid issues with duplicated locations by thinning, but prior to any thinning we want to censor any locations that could be otherwise problematic. This involves removing erroneous GPS locations (locations with a satellite count of 0, which were passed on to Movebank) and using a speed filter of 40 m/s.

Some cleaning steps are required as the data included quite some faulty positioning attempts, including location attempts with a satellite count of 0, locations placed at the intersection of equator/null-meridian, and locations that are outside the realm of movement speeds of greylag geese. Here, we filter out these problematic locations.

```
# filter locations at (0/0) and locations apparently collected with 0 GPS satellites
df <- df[df$gps.satellite.count>0,] 
df <- df[df$location.long!=0,] 

# filter outlier locations using speed filter (40 m/s as cut-off)
df <- ddply(df, 'individual.local.identifier', function(x){
  step <- distGeo(x[,c('location.long', 'location.lat')])[-nrow(x)]
  time <- abs(diff(as.numeric(x$timestamp)))
  speed <- step/time
  x <- x[c(speed, NA)<=40,]
  return(x)
})

# remove locations that do not seem to be deployed on an individual
df <- df[!is.na(df$individual.local.identifier),]

# backwards elimination of locations with ground speed of 0 m/s
df <- ddply(df, 'individual.local.identifier', function(x){
  check <- rev(x$ground.speed)[1]<0.5
  while(check & nrow(x)>0){
    x <- x[-nrow(x),]
    check <- rev(x$ground.speed)[1]<.5
  }
  return(x)
})

save(df, file='data/misc/241007_temp_hudiksvall.RData')
```

Now we need to determine tracking endpoints in case the reverse deletion of inactive locations was insufficient. Here I will have a look at the timeline of individuals, with suggested cut-off points for the trajectories based on exploration of the data:

Code

```
load('data/misc/241007_temp_hudiksvall.RData')

copy <- df
copy$date <- as.Date(copy$timestamp)
copy <- copy[!duplicated(copy[,c('individual.local.identifier', 'date')]),]
copy <- ddply(copy, 'individual.local.identifier', function(x){
  x$d2start <- distGeo(x[1,c('location.long', 'location.lat')], x[,c('location.long', 'location.lat')])/1000
  return(x)
})

cutoff <- data.frame(
  individual.local.identifier=c('D13_white_Sweden', 'D67_white_Sweden', 'DM2_white_Sweden'),
  date=as.Date(c('2021-04-19', '2019-03-29', '2018-06-19'))
)

ggplot(copy, aes(x=date, y=d2start)) +
  geom_path() +
  geom_vline(data=cutoff, aes(xintercept=date), colour='red', linetype=1) +
  facet_wrap(~individual.local.identifier, scales='free') +
  theme_light() +
  scale_y_sqrt() +
  labs(x='', y='Distance to first location (km)')
```

Visualisation of individual timelines, with date on the x-axis and distance to the first location on the y-axis.

Code

```
rm(list=c('df', 'copy'))
```

Censor locations after cut-off points:

Remove data after cut-off points for individuals in question.

```
df <- df[-which(df$individual.local.identifier=='D13_white_Sweden' & as.Date(df$timestamp)>as.Date('2021-04-19')),]
df <- df[-which(df$individual.local.identifier=='D67_white_Sweden' & as.Date(df$timestamp)>as.Date('2019-03-29')),]
df <- df[df$individual.local.identifier!='DM2_white_Sweden',]
```

Now we can proceed to the actual thinning of the data to daily locations. Here, we will try to retain the location that is closest to noon, as experienced by the birds. We will calculate time of day by determining solar noon at locations to do so.

Thin data to daily relocations, retaining the observation closest to solar noon.

```
# calculate actual time of day (relative to solar noon, in fractional hours)
p <- st_as_sf(df, coords=c('location.long', 'location.lat'), crs=st_crs('+proj=longlat +datum=WGS84'))
noon <- solarnoon(p, df$timestamp, POSIXct.out=TRUE)$time
tod <- as.numeric(difftime(df$timestamp, noon, units='hours'))
df$solar.time <- (tod-12)%%24

rm(p)

# separate tracking days by rolling over solar time
df <- ddply(df, 'individual.local.identifier', function(x){
  x$day <- c(0, cumsum(diff(x$solar.time)<(-0.1))) + 1
  return(x)
})

# add year & ordinal day
df$year <- year(df$timestamp)
df$julian <- as.numeric(strftime(df$timestamp, format='%j'))


# retain daily locations; location closest to solar noon
tracks01 <- ddply(df, c('individual.local.identifier', 'day'), function(x){
  x$tdist <- abs(x$solar.time - 12)
  return(x[which.min(x$tdist),])
})

tracks01$population <- 'Hudiksvall'

# use steel ring numbers as individual identifiers to make data compatible with the other study sites
tracks01$individual.local.identifier <- 
  unlist(lapply(tracks01$individual.local.identifier, function(id){
  tmp <- inf[inf$neckband.code==gsub('_.*', '', id) & !is.na(inf$neckband.code),]
  if(nrow(tmp)>1){
    min.year <- min(year(tracks01$timestamp[tracks01$individual.local.identifier==id]))
    tmp <- tmp[year(tmp$capture.date)==min.year,]
  }
  return(tmp$steel.ringnumber)
}))

rm(df); gc()

# save for easy access
save(tracks01, file='data/daily/240828_regularised_hudiksvall.RData')
```

#### Other capture locations

Birds at all other capture locations were marked with GPS nackbands supplied by Ornitela. Data collected by the tags were only available through the company’s own database at the time the study was conducted, so the procedure for importing the data differs slightly from the above procedure. Any filters and preparatory steps are however the same.

Import tracking data from other capture sites

```
# other populations
# using same procedure as for Sundsvall individuals
files <- list.files('data/tracking_data/by_location', pattern='.RData', full.names=T)

df <- rbind.fill(lapply(files, function(f){
  load(f)
  return(tracks)
}))
```

Using the same filtering steps as for the Hudiksvall birds. The only difference here are naming conventions.

Repeat the same cleaning steps as performed for the Hudiksvall data to avoid biases.

```
# filter locations at (0/0) and locations apparently collected with 0 GPS satellites
df <- df[df$satcount>0,] 
df <- df[df$Longitude!=0,] 

# filter outlier locations using speed filter (40 m/s as cut-off)
df <- ddply(df, 'individual.id', function(x){
  step <- distGeo(x[,c('Longitude', 'Latitude')])[-nrow(x)]
  time <- abs(diff(as.numeric(x$timestamp)))
  speed <- step/time
  x <- x[c(speed, NA)<=40,]
  return(x)
})

# remove locations that do not seem to be deployed on an individual
df <- df[!is.na(df$individual.id),]

# backwards elimination of locations with ground speed of 0 m/s
df$ground.speed <- df$speed_km_h/3.6

df <- ddply(df, 'individual.id', function(x){
  check <- rev(x$ground.speed)[1]<0.5
  while(check & nrow(x)>0){
    x <- x[-nrow(x),]
    check <- rev(x$ground.speed)[1]<.5
  }
  return(x)
})

save(df, file='data/misc/241007_temp_otherlocs.RData')
```

Again, we need to make sure to censor data from dead individuals/dropped tags, using the same kind of plot as for the Hudiksvall birds:

Code

```
load('data/misc/241007_temp_otherlocs.RData')

copy <- df[df$population=='Nyköping',]
copy$date <- as.Date(copy$timestamp)
copy <- copy[!duplicated(copy[,c('individual.id', 'date')]),]
copy <- ddply(copy, 'individual.id', function(x){
  x$d2start <- distGeo(x[1,c('Longitude', 'Latitude')], x[,c('Longitude', 'Latitude')])/1000
  return(x)
})

cutoff <- data.frame(
  individual.id=c('92V02197'),
  date=as.Date(c('2021-03-15'))
)

ggplot(copy, aes(x=date, y=d2start)) +
  geom_path() +
  geom_vline(data=cutoff, aes(xintercept=date), colour='red', linetype=1) +
  facet_wrap(~individual.id, scales='free') +
  theme_light() +
  scale_y_sqrt() +
  labs(x='', y='Distance to first location (km)', title='Nyköping')
```

Visualisation of individual timelines for birds captured in Nyköping, with date on the x-axis and distance to the first location on the y-axis.

Code

```
copy <- df[df$population=='Kvismaren',]
copy$date <- as.Date(copy$timestamp)
copy <- copy[!duplicated(copy[,c('individual.id', 'date')]),]
copy <- ddply(copy, 'individual.id', function(x){
  x$d2start <- distGeo(x[1,c('Longitude', 'Latitude')], x[,c('Longitude', 'Latitude')])/1000
  return(x)
})

cutoff <- data.frame(
  individual.id=c('9269806', '9269808', '9269828', '9269838', '9269846', 
                  '9269847', '9269848', '9269915', '9269920', '9269925',
                  '9269929', '9269931', '92V01801', '92V01865', '92V02752', 
                  '92V02756'),
  date=as.Date(c('2018-10-19', '2019-05-24', '2018-06-18', '2017-07-21', '2017-11-18', 
                 '2018-07-16', '2017-07-13', '2017-06-21', '2017-07-12', '2017-08-17',
                 '2017-07-13', '2017-07-13', '2017-07-14', '2018-10-25', '2020-01-01', 
                 '2019-08-01'))
)

ggplot(copy, aes(x=date, y=d2start)) +
  geom_path() +
  geom_vline(data=cutoff, aes(xintercept=date), colour='red', linetype=1) +
  facet_wrap(~individual.id, scales='free', ncol=4) +
  theme_light() +
  scale_y_sqrt() +
  labs(x='', y='Distance to first location (km)', title='Örebro')
```

Visualisation of individual timelines for birds captured in Örebro, with date on the x-axis and distance to the first location on the y-axis.

Code

```
copy <- df[df$population=='Svedala',]
copy$date <- as.Date(copy$timestamp)
copy <- copy[!duplicated(copy[,c('individual.id', 'date')]),]
copy <- ddply(copy, 'individual.id', function(x){
  x$d2start <- distGeo(x[1,c('Longitude', 'Latitude')], x[,c('Longitude', 'Latitude')])/1000
  return(x)
})

cutoff <- data.frame(
  individual.id=c('92699783', '92699783', '92699793', '92V02033', '92V02035', '92V02036'),
  date=as.Date(c('2017-10-16', '2017-11-14', '2018-04-06', '2019-07-31', '2018-11-21', '2019-01-06'))
)

ggplot(copy, aes(x=date, y=d2start)) +
  geom_path() +
  geom_vline(data=cutoff, aes(xintercept=date), colour='red', linetype=1) +
  facet_wrap(~individual.id, scales='free', ncol=4) +
  theme_light() +
  scale_y_sqrt() +
  labs(x='', y='Distance to first location (km)', title='Svedala')
```

Visualisation of individual timelines for birds captured in Svedala, with date on the x-axis and distance to the first location on the y-axis.

Code

```
copy <- df[df$population=='Kristianstad',]
copy$date <- as.Date(copy$timestamp)
copy <- copy[!duplicated(copy[,c('individual.id', 'date')]),]
copy <- ddply(copy, 'individual.id', function(x){
  x$d2start <- distGeo(x[1,c('Longitude', 'Latitude')], x[,c('Longitude', 'Latitude')])/1000
  return(x)
})

cutoff <- data.frame(
  individual.id=c('9269943', '92699760', '92699763', '92V02467'),
  date=as.Date(c('2018-09-25', '2017-09-15', '2018-04-10', '2020-04-01'))
)

ggplot(copy, aes(x=date, y=d2start)) +
  geom_path() +
  geom_vline(data=cutoff, aes(xintercept=date), colour='red', linetype=1) +
  facet_wrap(~individual.id, scales='free', ncol=4) +
  theme_light() +
  scale_y_sqrt() +
  labs(x='', y='Distance to first location (km)', title='Kristianstad')
```

Visualisation of individual timelines for birds captured in Kristianstad, with date on the x-axis and distance to the first location on the y-axis.

Censor locations after cut-off points:

Censor locations collected after cut-off points.

```
check <- unlist(lapply(1:nrow(cutoff), function(i){
  length(which(df$individual.id==cutoff$individual.id[i] & 
                  as.Date(df$timestamp)>cutoff$date[i]))
}))

for(i in which(check>0)){
  df <- df[-which(df$individual.id==cutoff$individual.id[i] & 
                  as.Date(df$timestamp)>cutoff$date[i]),]
}
```

Now we can proceed with the thinning of the data as done before the Hudiksvall data set:

Thin tracking data to daily relocations, retaining the locations closest to solar noon.

```
# calculate actual time of day (relative to solar noon, in fractional hours)
p <- st_as_sf(df, coords=c('Longitude', 'Latitude'), crs=st_crs('+proj=longlat +datum=WGS84'))
noon <- solarnoon(p, df$timestamp, POSIXct.out=TRUE)$time
tod <- as.numeric(difftime(df$timestamp, noon, units='hours'))
df$solar.time <- (tod-12)%%24

rm(p)

# separate tracking days by rolling over solar time
df <- ddply(df, 'individual.id', function(x){
  x$day <- c(0, cumsum(diff(x$solar.time)<(-0.1))) + 1
  return(x)
})

# add year & ordinal day
df$year <- year(df$timestamp)
df$julian <- as.numeric(strftime(df$timestamp, format='%j'))

# retain daily locations; location closest to solar noon
tracks02 <- ddply(df, c('individual.id', 'day'), function(x){
  x$tdist <- abs(x$solar.time - 12)
  return(x[which.min(x$tdist),])
})

# renomae Kvismaren to Örebro
tracks02$population[tracks02$population=='Kvismaren'] <- 'Örebro'

# save for easy access
save(tracks02, file='data/daily/240828_regularised_other_locations.RData')
```

#### Combine tracking data sets

Here we will align naming conventions and sensory information shared between the two types of tags, and merge the GPS data into a single data set. Here we also merge the data with the master sheet to attach information about individuals as recorded during capture.

Combine tracking data from Hudiksvall and other locations in a single table, and merge tracking data with the master sheet.

```
keep <- c('population', 'timestamp', 'individual.local.identifier', 
          'tag.local.identifier', 'location.long', 'location.lat', 
          'year', 'day', 'julian', 'solar.time')

names(tracks02)[c(3,6,2,1,7,8,20,19,21,17)] <- keep

all.tracks <- rbind(tracks01[,keep], tracks02[,keep])
all.tracks <- all.tracks[all.tracks$location.lat>5,]

# merge with master sheet
add.inf <- rbind.fill(lapply(1:nrow(all.tracks), function(j){
  tmp <- inf[inf$steel.ringnumber==all.tracks$individual.local.identifier[j],]
  tmp <- tmp[tmp$capture.status=='new',]
  return(tmp[,c('age', 'sex', 'tarsus', 'bodymass')])
}))

all.tracks <- cbind(all.tracks, add.inf)
rm(list=c('add.inf', 'tracks01', 'tracks02')); gc()
```

## Censoring of locations biased by family members

During the capture on the moulting sites, it was not feasible to identify families. Consequently, it is possible that more than one member of a family was captured and marked, making the resulting tracks non-independent. To avoid families biasing the results, we decided to only retain data for one individual per family.

Some information on family members were collected in the field when observing the birds. This we will leverage to identify potential members of the same family. We will also use pairwise distances between GPS-tagged individuals and comments provided by the geese.org observers to try and identify family members.

### Censoring of data collected by juveniles

Juvenile geese can (but do not necessarily) stay with their parents until spring migration. This is relatively straightforward as we know the age of all birds at capture. We will censor the data of juveniles up until April 01 of their second year, and only retain data that is collected after.

Censor locations of individuals that were captured as juveniles up to April 01 of their second calendar year.

```
# determine threshold date for each individual
# if adult at capture: day of capture
# if juvenile at capture: April 1st of the following year
inf <- ddply(inf, 'steel.ringnumber', function(x){
  x <- x[x$capture.status=='new',]
  x$threshold.date <- ifelse(x$age=='adult', x$capture.date, as.Date(paste(year(x$capture.date)+1, '04-01', sep='-')))
  return(x)
})

# censor juvenile data in neckband data set
nb <- ddply(nb, 'steel.ringnumber', function(x){
  id <- unique(x$steel.ringnumber)
  x <- x[as.numeric(x$date)>as.numeric(inf$threshold.date[inf$steel.ringnumber==id]),]
  return(x)
})

# censor juvenile data in GPS tracking data set
all.tracks$date <- as.Date(all.tracks$timestamp)
all.tracks <- ddply(all.tracks, 'individual.local.identifier', function(x){
  id <- unique(x$individual.local.identifier)
  x <- x[x$date>inf$threshold.date[inf$steel.ringnumber==id],]
  return(x)
})
```

### Censoring of data collected by partners

For partners in a pair, we will determine total number of observations and observation duration. The partner that has a shorter observation period and/or fewer observations will be excluded from the data set.

#### Own observations

Some individuals were identified as mating partners through field observations. Here, we will determine the number of observations and total observation period for each individual to help with the decision-making of which individual to keep from a pair.

```
# "untagged partner" was set to NA since we have no additional information
pairs <- read.delim('data/goose_pairs/pairs.csv', header=T, sep=';')
pairs <- pairs[!is.na(pairs$Partner) & pairs$Partner!='CAMILLA',]

# import second copy of master sheet, for modification
inf.tmp <- rbind.fill(lapply('data/tracking_data/reference_data.RData', function(f){load(f); return(inf)}))
inf.tmp <- inf.tmp[!is.na(inf.tmp$neckband.code),]
inf.tmp <- inf.tmp[inf.tmp$steel.ringnumber!='92V02065',]
inf.tmp <- inf.tmp[inf.tmp$capture.status=='new',]

pairs$set <- 1:nrow(pairs)

# reshape data into pairwise 
partners.obs <- ddply(pairs, 'set', function(x){
  if(grepl('and', x$Partner)){
    partner <- strsplit(x$Partner, ' and ')[[1]]
    partner <- gsub(' ', '', partner)
  }else{
    partner <- x$Partner
  }
  new <- expand.grid(id01=c(x$Neckband.code, partner), id02=c(x$Neckband.code, partner), KEEP.OUT.ATTRS = F)
  new <- new[new$id01!=new$id02,]
  return(new)
})
partners.obs <- partners.obs[!duplicated(partners.obs[,-1]),]

# add duration of observations (for juveniles only after April of the second year), age, number of locations to combinations

partners.obs <- ddply(partners.obs, c('id01', 'id02'), function(x){
  bird1 <- x$id01; bird2 <- x$id02
  inf.tmp01 <- inf.tmp[!is.na(inf.tmp$neckband.code) & inf.tmp$neckband.code==bird1,]
  if(!inf.tmp01$transmitter){
    type1 <- 'neckband'
    if(inf.tmp01$age=='adult'){
      dur1 <- as.numeric(max(nb$date[nb$steel.ringnumber==inf.tmp01$steel.ringnumber]) - inf.tmp01$capture.date)
      n1 <- nrow(nb[nb$steel.ringnumber==inf.tmp01$steel.ringnumber,])
    }else{
      y <- year(inf.tmp01$capture.date)
      tmp <- nb[nb$steel.ringnumber==inf.tmp01$steel.ringnumber,]
      tmp <- tmp[tmp$date>=as.Date(paste(y+1, '04-01', sep='-')),]
      dur1 <- as.numeric(max(tmp$date) - min(tmp$date))
      n1 <- nrow(tmp)
    }
  }else{
    type1 <- 'gps'
    if(inf.tmp01$age=='adult'){
      tmp <- all.tracks[all.tracks$individual.local.identifier==inf.tmp01$steel.ringnumber,]
      dur1 <- as.numeric(max(as.Date(tmp$timestamp)) - min(as.Date(tmp$timestamp)))
      n1 <- nrow(tmp)
    }else{
      y <- year(inf.tmp01$capture.date)
      tmp <- all.tracks[all.tracks$individual.local.identifier==inf.tmp01$steel.ringnumber,]
      tmp <- tmp[as.Date(tmp$timestamp)>=as.Date(paste(y+1, '04-01', sep='-')),]
      dur1 <- as.numeric(max(as.Date(tmp$timestamp)) - min(as.Date(tmp$timestamp)))
      n1 <- nrow(tmp)
    }
  }
  
  inf.tmp02 <- inf.tmp[!is.na(inf.tmp$neckband.code) & inf.tmp$neckband.code==bird2,]
  if(!inf.tmp02$transmitter){
    type2 <- 'neckband'
    if(inf.tmp02$age=='adult'){
      dur2 <- as.numeric(max(nb$date[nb$steel.ringnumber==inf.tmp02$steel.ringnumber]) - inf.tmp02$capture.date)
      n2 <- nrow(nb[nb$steel.ringnumber==inf.tmp02$steel.ringnumber,])
    }else{
      y <- year(inf.tmp02$capture.date)
      tmp <- nb[nb$steel.ringnumber==inf.tmp02$steel.ringnumber,]
      tmp <- tmp[tmp$date>=as.Date(paste(y+1, '04-01', sep='-')),]
      dur2 <- as.numeric(max(tmp$date) - min(tmp$date))
      n2 <- nrow(tmp)
    }
  }else{
    type2 <- 'gps'
    if(inf.tmp02$age=='adult'){
      tmp <- all.tracks[all.tracks$individual.local.identifier==inf.tmp02$steel.ringnumber,]
      dur2 <- as.numeric(max(as.Date(tmp$timestamp)) - min(as.Date(tmp$timestamp)))
      n2 <- nrow(tmp)
    }else{
      y <- year(inf.tmp02$capture.date)
      tmp <- all.tracks[all.tracks$individual.local.identifier==inf.tmp02$steel.ringnumber,]
      tmp <- tmp[as.Date(tmp$timestamp)>=as.Date(paste(y+1, '04-01', sep='-')),]
      dur2 <- as.numeric(max(as.Date(tmp$timestamp)) - min(as.Date(tmp$timestamp)))
      n2 <- nrow(tmp)
    }
  }
  x$dur01 <- dur1; x$n01 <- n1; x$type01 <- type1
  x$dur02 <- dur2; x$n02 <- n2; x$type02 <- type2
  return(x)
})

partners.obs$population <- unlist(lapply(as.character(partners.obs$id01), function(id){unique(inf$population[inf$neckband.code==id & !is.na(inf$neckband.code)])}))
```

Now we can censor the data for those birds that have the shorter trajectory with less observations. Where partners in a pair are subjected to different tracking methods, this decision gets somewhat more tricky. We will likely be unable to fit a meaningful movement model to a birds with just few neckband observations, but those that have many neckband observations likely outweigh a GPS-tracked bird in how much it contributes to the population-level estimates. Here, we will decide on a case-by-case basis.

Use information from above to censor partners with less observations.

```
#################################################
# Pairs with two gps-tracked partners

# identify partner with shorter track
rem.gps <- partners.obs[partners.obs$type01=='gps' & partners.obs$type01==partners.obs$type02,]
rem.gps <- as.character(unlist(lapply(1:nrow(rem.gps), function(j){
  if(rem.gps$dur01[j]>rem.gps$dur02[j]){
    return(inf$steel.ringnumber[inf$neckband.code==rem.gps$id02[j]])
  }else{
    return(inf$steel.ringnumber[inf$neckband.code==rem.gps$id01[j]])
  }
}))); rem.gps <- unique(rem.gps[complete.cases(rem.gps)])

# remove suggested individuals
all.tracks <- all.tracks[!all.tracks$individual.local.identifier %in% rem.gps,]

#################################################
# Pairs with two neckband partners
rem.nb <- partners.obs[partners.obs$type01=='nb' & partners.obs$type01==partners.obs$type02,]
nrow(rem.nb)
# no need as no observed pairs have two neckband birds

#################################################
# Pairs with one neckband and one GPS-tracked bird
rem <- partners.obs[partners.obs$type01!=partners.obs$type02 & !duplicated(partners.obs$set),]
print(rem)
remove.nb <- c('747', '7HF', '7SD', 'L17', 'L16', # Hudiksvall
               '418', '7BF', # Nyköping
               '4EH') # Örebro
remove.gps <- c('D13', 'D27', 'D67', 'DM2', 'DM4', 'DM5', # Hudiksvall
                'S30', 'D74', 'S71', 'S73', # Nyköping
                'S89', '4EH', 'S94') # Örebro
remove.gps.id <- unlist(lapply(remove.gps, function(id){inf$steel.ringnumber[inf$neckband.code==id & !is.na(inf$neckband.code)]}))

nb <- nb[!nb$neckband.code %in% remove.nb,] # remove total 117 locations (~1.7 %)
all.tracks <- all.tracks[!all.tracks$individual.local.identifier %in% remove.gps.id,] # removes 6,694 locations (~7 %)
```

### Comments from geese.org reports

We can also have a look at how frequently birds were reported to be seen together in the geese.org observation reports. After looking the reports, we decided to just search the reporting data for keywords meaning “with” or “together” or similar in different languages. We then tally up the number of these kinds of observations. Most individuals are reported together only once, and we decided that birds need to be observed together at least twice to qualify for censoring. Note that sometimes birds were reported together with a GPS-tagged birds without giving the code on the GPS, so we won’t be able to identify some pairings using this method.

Using pattern recognition to determine which neckband birds were reported in presence of other birds. Looking through the data, I found that people tend to report when they see a neckband bird together with a GPS collar bird, but don’t mention the code on the GPS collar.

```
# master sheet
inf.tmp <- rbind.fill(lapply('data/tracking_data/reference_data.RData', function(f){load(f); return(inf)}))
inf.tmp <- inf.tmp[!is.na(inf.tmp$neckband.code),]

# import geese_org observations (state of 2023-02-21)
load('data/neckband_data/20230221_neckband_data.RData')
nb.data <- nb.data[nb.data$OBS_REMARK!='',]
nb.data <- nb.data[!duplicated(nb.data$OAS_ID),]

# remove juvenile observations before April of their second year
threshold.date <- as.Date(paste(year(nb.data$capture.date)+1, '04-01', sep='-'))
nb.data <- nb.data[nb.data$age=='adult' | nb.data$date >= threshold.date,]

# some variations of together/with observed in the observation comments. Note that this list might not be comprehensive for other studies
pattern <- c('together', 'zusammen', 'samen', 'avec', 'with', 'pair', 'partner', 'paar', 'ihop', 'mate')

new <- ddply(nb.data, 'OAS_ID', function(tmp){
  x <- unlist(lapply(pattern, grepl, x=tmp$OBS_REMARK))
  if(!any(x)){
    return(NULL)
  }else{
    x <- strsplit(tmp$OBS_REMARK, ' ')[[1]]
    if(any(x %in% inf$neckband.code)){
      partner <- x[which(x %in% inf$neckband.code)]
    }else{
      partner <- NA
    }
    tmp <- do.call('rbind', lapply(1:length(partner), function(j){tmp}))
    tmp$potential.partner <- partner
    return(tmp[,c('neckband.code', 'date', 'OBS_REMARK', 'potential.partner')])
  }
})

new <- new[!is.na(new$potential.partner),]
partners.nb <- as.data.frame(t(combn(unique(c(new$neckband.code, new$potential.partner)), 2)))
partners.nb$count <- unlist(lapply(1:nrow(partners.nb), function(j){
  a <- new[new$neckband.code==partners.nb$V1[j] & new$potential.partner==partners.nb$V2[j],]
  b <- new[new$potential.partner==partners.nb$V1[j] & new$neckband.code==partners.nb$V2[j],]
  return(nrow(a)+nrow(b))
}))
partners.nb$pop01 <- unlist(lapply(partners.nb$V1, function(id){inf$population[inf$neckband.code==id & !is.na(inf$neckband.code)]}))
partners.nb$pop02 <- unlist(lapply(partners.nb$V2, function(id){inf$population[inf$neckband.code==id & !is.na(inf$neckband.code)]}))
partners.nb <- partners.nb[partners.nb$count>1 & partners.nb$pop01==partners.nb$pop02,]

print(partners.nb)

# are they still present in the data set?
table(c(partners.nb$V1, partners.nb$V2) %in% nb)
# no need to remove anything as these individuals are no longer present in the data
```

### Pairwise distance of GPS-tracked birds

It is unnecessary to check for potential partners in the Hudiksvall population as only adult female birds were tagged there, but for the other locations we can have a look at pairwise distances of male and female birds during June (when birds are mostly flightless).

Calculate pairwise distances between individuals from the same capture site during the month of June.

```
inf <- inf[inf$steel.ringnumber %in% all.tracks$individual.local.identifier,]

# make a data frame with all possible pairings of female and male birds per location:
combi <- ddply(inf, 'population', function(x){
  females <- x$steel.ringnumber[x$sex=='female']
  males <- x$steel.ringnumber[x$sex=='male']
  new <- expand.grid(male=males, female=females, KEEP.OUT.ATTRS=F, stringsAsFactors=F)
})

# calculate pairwise distance for all pairings during July
partners.gps <- ddply(combi, c('population', 'male', 'female'), function(tmp){
  check <- which(combi$male==tmp$male & combi$female==tmp$female)
  # print(check)
  
  male <- all.tracks[all.tracks$individual.local.identifier==tmp$male,]
  male.july <- unique(as.Date(male$timestamp[month(male$timestamp)==6]))
  
  female <- all.tracks[all.tracks$individual.local.identifier==tmp$female,]
  female.july <- unique(as.Date(male$timestamp[month(female$timestamp)==6]))
  
  same.dates <- intersect(male.july, female.july)
  
  if(length(same.dates)>0){
    d <- unlist(lapply(same.dates, function(d){
      m <- male[as.Date(male$timestamp)==d,]
      f <- female[as.Date(female$timestamp)==d,]
      
      dist <- unlist(lapply(1:nrow(m), function(j){
        p1 <- m[j,c('location.long', 'location.lat')]
        td <- abs(as.numeric(m$timestamp[j]) - as.numeric(f$timestamp[j]))
        if(any(td<1800) & !is.na(td)){
          p2 <- f[which.min(td),c('location.long', 'location.lat')]  
          return(distGeo(p1,p2))
        }else{return(NULL)}
      }))
      if(is.null(dist)){return(NULL)}else{return(min(dist))}
    }))
    tmp$shared.years <- length(unique(year(as.Date(same.dates, origin='1970-01-01'))))
    tmp$overlap <- length(same.dates)
    tmp$range.male <- as.numeric(abs(diff(range(as.Date(male$timestamp, origin='1970-01-01')))))
    tmp$range.female <- as.numeric(abs(diff(range(as.Date(female$timestamp, origin='1970-01-01')))))
    tmp$mean.dist <- ifelse(length(d)>0, mean(d, na.rm=T), NA)
  }else{
    tmp$shared.years <- tmp$overlap <- 0
    tmp$range.male <- tmp$range.female <- tmp$mean.dist <- NA
  }
  
  return(tmp)
})

# retain combinations that have a mean pairwise distance < 1000m
partners.gps <- partners.gps[partners.gps$mean.dist<1000 & !is.na(partners.gps$mean.dist),]

# add neckband codes
partners.gps$male_nb <- unlist(lapply(partners.gps$male, function(ring){inf$neckband.code[inf$steel.ringnumber==ring]}))
partners.gps$female_nb <- unlist(lapply(partners.gps$female, function(ring){inf$neckband.code[inf$steel.ringnumber==ring]}))

save(partners.gps, file='data/misc/241007_gps_partners.RData')
```

Code

```
(load('data/misc/241007_gps_partners.RData'))
```

```
[1] "partners.gps"
```

Code

```
ggplot(partners.gps[partners.gps$population=='Kristianstad',], aes(x=male_nb, y=female_nb, fill=mean.dist)) +
  geom_tile(colour='grey20', linewidth=0.1) + 
  scale_fill_viridis_b(name='Mean distance [m]', trans='log1p', limits=c(1,1000), breaks=c(1,10,50,100,1000), na.value=NA) +
  guides(colour=guide_legend(keywidth = unit(5, 'cm'))) +
  coord_equal() +
  theme_light() + 
  theme(strip.text = element_text(face='bold', size=10), 
        axis.text.x=element_text(angle=90, vjust=1),
        legend.position='bottom') +
  labs(x='Male', y='Female', title='Kristianstad')
```

Mean pairwise distance between individuals during the month of June, with one panel per capture location. There are no figures for Nyköping or Hudiksvall as there were no possible pairings for these populations.

Code

```
ggplot(partners.gps[partners.gps$population=='Svedala',], aes(x=male_nb, y=female_nb, fill=mean.dist)) +
  geom_tile(colour='grey20', linewidth=0.1) + 
  scale_fill_viridis_b(name='Mean distance [m]', trans='log1p', limits=c(1,1000), breaks=c(1,10,50,100,1000), na.value=NA) +
  guides(colour=guide_legend(keywidth = unit(5, 'cm'))) +
  coord_equal() +
  theme_light() + 
  theme(strip.text = element_text(face='bold', size=10), 
        axis.text.x=element_text(angle=90, vjust=1),
        legend.position='bottom') +
  labs(x='Male', y='Female', title='Svedala')
```

Mean pairwise distance between individuals during the month of June, with one panel per capture location. There are no figures for Nyköping or Hudiksvall as there were no possible pairings for these populations.

Code

```
ggplot(partners.gps[partners.gps$population=='Örebro',], aes(x=male_nb, y=female_nb, fill=mean.dist)) +
  geom_tile(colour='grey20', linewidth=0.1) + 
  scale_fill_viridis_b(name='Mean distance [m]', trans='log1p', limits=c(1,1000), breaks=c(1,10,50,100,1000), na.value=NA) +
  guides(colour=guide_legend(keywidth = unit(5, 'cm'))) +
  coord_equal() +
  theme_light() + 
  theme(strip.text = element_text(face='bold', size=10), 
        axis.text.x=element_text(angle=90, vjust=1),
        legend.position='bottom') +
  labs(x='Male', y='Female', title='Örebro')
```

Mean pairwise distance between individuals during the month of June, with one panel per capture location. There are no figures for Nyköping or Hudiksvall as there were no possible pairings for these populations.

From the figures, it seems that there is only S15 and K18 from the Svedala population that were in very close spatial proximity throughout. Which of these individuals was tracked for longer, and had more locations?

Determine number of observations/duration of observation for S15 and K18.

```
partners.gps[partners.gps$female_nb=='S15' & partners.gps$male_nb=='K18',]

# female
diff(range(all.tracks$date[all.tracks$individual.local.identifier=='92699792']))
nrow(all.tracks[all.tracks$individual.local.identifier=='92699792',])

# male
diff(range(all.tracks$date[all.tracks$individual.local.identifier=='92V01763']))
nrow(all.tracks[all.tracks$individual.local.identifier=='92V01763',])
```

The female has a decidedly longer span of observation with more locations, so the best choice would be to censor the male bird:

Censor the individual with fewer observations.

```
all.tracks <- all.tracks[all.tracks$individual.local.identifier!='92V01763',]
```

With all cleaning step complete, I will write the data to file for convenience:

Write data to file.

```
save(nb, all.tracks, file='data/daily/240828_neckband_gps_clean.RData')
```

## Export of neckband observations, GPS tracking data, and metadata on individuals for upload on zenodo

Reformat and export the data for upload on zenodo.

```
###################################################################################################
### Neckband data

# comma-separated values
# population: site that the individual was captured at
# population.short: shorthand for the capture site as used in the article
# transmitter: logical; was the individual equipped with a GPS-transmitter?
# transmitter.id: numerical identifier for the deployed GPS-transmitter; NA if none
# neckband.code: 3-letter code of the neckband/GPS transmitter to be reported by observers
# steel.ringnumber: unique identifier of the steel tarsal band of that bird
# date: date when the individual was observed, as reported
# location.lat: Latitude of the reported observation, in decimal degrees
# location.long: Longitude of the reported observation, in decimal degrees

keep <- c('population', 'transmitter', 'transmitter.id', 'neckband.code', 'steel.ringnumber', 'date', 'LATITUDE', 'LONGITUDE')
rename <- c('population', 'transmitter', 'transmitter.id', 'neckband.code', 'steel.ringnumber', 'date', 'location.lat', 'location.long')

nb.data <- nb[,keep]
names(nb.data) <- rename

# add shorthands for capture sites
nb.data$population.short <- ifelse(nb.data$population=='Kristianstad', 'S2',
                                   ifelse(nb.data$population=='Svedala', 'S1',
                                          ifelse(nb.data$population=='Nyköping', 'C2',
                                                 ifelse(nb.data$population=='Örebro', 'C1',
                                                        ifelse(nb.data$population=='Hudiksvall', 'N', NA)))))
nb.data <- nb.data[,c(1,ncol(nb.data),2:(ncol(nb.data)-1))]

write.table(nb.data, file='250826_greylag_goose_cmr_data.csv', row.names=F, sep=',')

###################################################################################################
### GPS tracking data

# comma-separated values
# population: site that the individual was captured at
# population.short: shorthand for the capture site as used in the article
# transmitter: logical; was the individual equipped with a GPS-transmitter?
# transmitter.id: numerical identifier for the deployed GPS-transmitter; NA if none
# neckband.code: 3-letter code of the neckband/GPS transmitter to be reported by observers
# steel.ringnumber: unique identifier of the steel tarsal band of that bird
# timestamp: time and date of the acquisition of the GPS location (timezone: UTC, format: yyyy-MM-dd HH:mm:ss)
# location.lat: Latitude of the reported observation, in decimal degrees
# location.long: Longitude of the reported observation, in decimal degrees

keep <- c('population', 'tag.local.identifier', 'individual.local.identifier', 'timestamp', 'location.long', 'location.lat')
rename <- c('population', 'transmitter.id', 'steel.ringnumber', 'timestamp', 'location.long', 'location.lat')

# 'transmitter', 'transmitter.id', 'neckband.code', 

gps.data <- all.tracks[,keep]
names(gps.data) <- rename

# gps-transmitter birds do have a transmitter
gps.data$transmitter <- TRUE

# add neckband code
gps.data$neckband.code <- unlist(lapply(gps.data$steel.ringnumber, function(id){
  inf$neckband.code[inf$steel.ringnumber==id]
}))

# add shorthands for capture sites
gps.data$population.short <- ifelse(gps.data$population=='Kristianstad', 'S2',
                                    ifelse(gps.data$population=='Svedala', 'S1',
                                           ifelse(gps.data$population=='Nyköping', 'C2',
                                                  ifelse(gps.data$population=='Örebro', 'C1',
                                                         ifelse(gps.data$population=='Hudiksvall', 'N', NA)))))

# sort columns
sort.names <- names(nb.data); sort.names[sort.names=='date'] <- 'timestamp'
gps.data <- gps.data[,sort.names]

write.table(gps.data, file='250826_greylag_goose_gps_data.csv', row.names=F, sep=',')

###################################################################################################
### Capture details

# comma-separated values
# population: site that the individual was captured at
# population.short: shorthand for the capture site as used in the article
# transmitter: logical; was the individual equipped with a GPS-transmitter?
# transmitter.id: numerical identifier for the deployed GPS-transmitter; NA if none
# neckband.code: 3-letter code of the neckband/GPS transmitter to be reported by observers
# steel.ringnumber: unique identifier of the steel tarsal band of that bird
# capture.date: date of first capture of the individual
# capture.lat: Latitude of the capture site, in decimal degrees
# capture.long: Longitude of the capture.site, in decimal degrees
# age: age of the individual at capture
# sex: sex of the individual
# bodymass: bodymass of the individual at capture, in grams

keep <- c('population', 'transmitter', 'transmitter.id', 'neckband.code', 'steel.ringnumber', 'capture.date', 'lat', 'long', 'age', 'sex', 'bodymass')
rename <- c('population', 'transmitter', 'transmitter.id', 'neckband.code', 'steel.ringnumber', 'capture.date', 'capture.lat', 'capture.long', 'age', 'sex', 'bodymass')

inf.data <- inf[inf$capture.status=='new',keep]
names(inf.data) <- rename

# individuals with GPS-data/neckband reports only
inf <- inf[inf$steel.ringnumber %in% c(unique(nb.data$steel.ringnumber), unique(gps.data$steel.ringnumber)),]

# add shorthands for capture sites
inf.data$population.short <- ifelse(inf.data$population=='Kristianstad', 'S2',
                                    ifelse(inf.data$population=='Svedala', 'S1',
                                           ifelse(inf.data$population=='Nyköping', 'C2',
                                                  ifelse(inf.data$population=='Örebro', 'C1',
                                                         ifelse(inf.data$population=='Hudiksvall', 'N', NA)))))

# sort columns
inf.data <- inf.data[,c(1,ncol(inf.data),2:(ncol(inf.data)-1))]

write.table(inf.data, file='250826_greylag_goose_metadata.csv', row.names=F, sep=',')
```

## Data overview

### Sample sizes

Print remaining sample sizes (here that refers to the number of individuals) for each capture site and tracking method.

```
knitr::kable(as.data.frame(table(nb$population[!duplicated(nb$neckband.code)])), col.names = c('Capture site', 'Number of individuals'), caption = 'Neckband birds')
```

Neckband birds

| Capture site | Number of individuals |
| --- | --- |
| Hudiksvall | 237 |
| Kristianstad | 6 |
| Nyköping | 152 |
| Örebro | 90 |
| Svedala | 8 |

Print remaining sample sizes (here that refers to the number of individuals) for each capture site and tracking method.

```
knitr::kable(as.data.frame(table(all.tracks$population[!duplicated(all.tracks$individual.local.identifier)])), col.names = c('Capture site', 'Number of individuals'), caption = 'Neckband birds')
```

Neckband birds

| Capture site | Number of individuals |
| --- | --- |
| Hudiksvall | 8 |
| Kristianstad | 24 |
| Nyköping | 11 |
| Örebro | 45 |
| Svedala | 31 |

### Summary figures

### Map

Code

```
all.tracks$method <- 'GPS collar'
nb$method <- 'Neckband'

range.x <- range(nb$LONGITUDE)+c(-1,1)
range.y <- range(nb$LATITUDE)+c(-1,1)

theme_map <- theme_light() +
  theme(axis.title=element_blank(), 
        axis.text=element_blank(),
        axis.ticks=element_blank(),
        panel.grid=element_blank(),
        text = element_text(color = "#22211d"), 
        legend.title=element_text(color = "#22211d", size=10),
        legend.text=element_text(color = "#22211d", size=9),
        plot.background = element_rect(fill = 'white', color = NA), 
        panel.background = element_rect(fill = '#dcebf0', color = NA), 
        legend.background = element_rect(fill = NA, color = NA), ##f5f5f2
        strip.background = element_rect(fill='white'),
        strip.text=element_text(colour='black', face='bold'),
        panel.border = element_blank())

ggplot() +
  geom_sf(data=adm, fill='antiquewhite', colour='grey20', linewidth=0.2) +
  geom_point(data=all.tracks, aes(x=location.long, y=location.lat, colour=method, group=individual.local.identifier), size=1, alpha=.5) +
  geom_point(data=nb, aes(x=LONGITUDE, y=LATITUDE, colour=method, group=neckband.code), size=1, alpha=.5) +
  theme_map +
  facet_grid(population~method) +
  scale_colour_viridis_d(name='', end=0.5, option='C', direction=-1) +
  guides(colour=guide_legend(override.aes = list(size=2.5, alpha=1))) +
  xlim(range.x) +
  ylim(range.y) +
  theme(legend.position='none')
```

Maps show the data collected using the two different tracking methods side by side, and per capture location.

Code

```
locs <- ddply(inf, 'population', function(x){
  data.frame(x=x$long[1], y=x$lat[1], n=sum(x$capture.status=='new'))
})
locs <- locs[with(locs, order(y)),]
locs <- st_as_sf(locs, coords=c('x', 'y'), crs=st_crs(proj.ll))

labels <- data.frame(
  x=rep(-1000000,5), 
  y=seq(0, 1100000, length.out=5), 
  label=rev(c('Hudiksvall', 'Örebro', 'Nyköping', 'Svedala', 'Kristianstad')),
  xend=st_coordinates(st_transform(locs, st_crs(proj.olaea)))[,1]-50000,
  yend=st_coordinates(st_transform(locs, st_crs(proj.olaea)))[,2]
)
labels$label <- factor(labels$label, levels=c('Hudiksvall', 'Örebro', 'Nyköping', 'Svedala', 'Kristianstad'),
                       labels=c('N', 'C1', 'C2', 'S1', 'S2'))

col <- c(
  N='#FDC926FF', # Hudiksvall
  C1='#ED7953FF', # Örebro
  C2='#BD3786FF', # Nyköping
  S1='#348AA6FF', # Svedala
  S2='#4B0C6BFF') # Kristianstad
names(col) <- rev(names(col))

p1 <- st_as_sf(all.tracks, coords=c('location.long', 'location.lat'), crs=st_crs(proj.ll))
p1$method <- factor(p1$method, levels=c('GPS collar', 'Neckband'), labels=c('a) GPS collar', 'b) Neckband'))
p1$population <- factor(p1$population, levels=c('Hudiksvall', 'Örebro', 'Nyköping', 'Svedala', 'Kristianstad'),
                        labels=c('N', 'C1', 'C2', 'S1', 'S2'))
p2 <- st_as_sf(nb, coords=c('LONGITUDE', 'LATITUDE'), crs=st_crs(proj.ll))
p2$method <- factor(p2$method, levels=c('GPS collar', 'Neckband'), labels=c('a) GPS collar', 'b) Neckband'))
p2$population <- factor(p2$population, levels=c('Hudiksvall', 'Örebro', 'Nyköping', 'Svedala', 'Kristianstad'),
                        labels=c('N', 'C1', 'C2', 'S1', 'S2'))

range.x <- c(-1400000, 900000)
range.y <- c(-1700000, 1300000)

p <- ggplot() +
  geom_sf(data=st_transform(adm, st_crs(proj.olaea)), fill='#D2D2D1', colour='#22211d', linewidth=0.2) +
  geom_sf(data=st_graticule(adm, lon = seq(-10,50,10), lat=seq(40,90,10)), colour='#22211d', linewidth=0.05) +
  geom_sf(data=p1, aes(colour=population), size=1, alpha=.5) +
  geom_sf(data=p2, aes(colour=population), size=1, alpha=.5) +
  geom_sf(data=locs, shape=21, colour='black', fill=NA, stroke=2, size=3) +
  geom_label(data=labels, aes(x=x, y=y, label=label), 
                alpha=.8, label.r=unit(0,'mm'), label.size=unit(0,'mm')) +
  # geom_segment(data=labels, aes(x=x+c(370,400,350,300,300)*1000, y=y, xend=xend, yend=yend), linewidth=0.5) +
  geom_segment(data=labels, aes(x=x + 150000, y=y, xend=xend, yend=yend), linewidth=0.5) +
  facet_wrap(~method, ncol=2) +
  scale_colour_manual(name='', values=col) +
  guides(colour=guide_legend(override.aes = list(size=3.5, alpha=1))) +
  xlim(range.x) +
  ylim(range.y) +
  labs(x='Longitude', y='Latitude') +
  theme_geese +
  theme(plot.background = element_rect(fill = 'white', color = NA), 
        panel.background = element_rect(fill = 'white', color = "#22211d"), 
        legend.position='bottom')

# ggsave(plot=p, filename='250113_data_overview.png', width=166, height=120, unit='mm')
# ggsave(plot=p, filename='figure01_tracking_data.png', width=170, height=120, unit='mm', dpi=300)

print(p)
```

---

# Continuous-time movement models

The next step is converting the data to the object class expected by the ctmm-package. Following that, I will compute semivariograms for each individual, average the semivariograms for each tracking method and capture site so as to allow for a visual comparison.

Calculate and summarise semivariogramsper tracking method and capture location.

```
## reshape neckband observation data into pseudo-Movebank data
nb <- ddply(nb, 'neckband.code', function(x){
  bands <- x[,c('population', 'steel.ringnumber', 'LONGITUDE', 'LATITUDE', 
                'age', 'sex', 'transmitter')]
  names(bands)[c(2:4)] <- 
    c('individual.local.identifier', 'location.long', 'location.lat')
  bands$year <- year(x$date); bands$julian <- as.numeric(strftime(x$date, format='%j'))
  bands$type <- 'neckband'
  bands$timestamp <- as.POSIXct(paste(x$OBS_DATE, x$OBS_TIME), 
                                tz='UTC', format='%d/%m/%Y %H:%M')

  if(nrow(bands)>1){
    return(bands)
  }else{return(NULL)}
})

nb <- ddply(nb, 'individual.local.identifier', function(x){
  x <- x[!duplicated(x[,c('timestamp')]),]
  x <- x[with(x, order(timestamp)),]
  return(x)
})

# convert to telemetry object
tel.nb <- as.telemetry(nb)
tel.nb <- tel.nb[!is.na(summary(tel.nb)$`interval (day)`)]

# calculate semivariance & average per population
var.nb <- lapply(unique(nb$population), function(p){
  keep <- unique(nb$individual.local.identifier[nb$population==p])
  keep <- keep[keep %in% names(tel.nb)]
  var <- lapply(keep, function(id){variogram(tel.nb[[id]], dt=7%#%'days')})
  return(mean(var))
}); names(var.nb) <- unique(nb$population)

svf.nb <- rbind.fill(lapply(names(var.nb), function(p){
  d <- as.data.frame(var.nb[[p]]@.Data)
  names(d) <- var.nb[[p]]@names
  d$lag <- d$lag / 86400 # convert to days
  d$SVF <- d$SVF / 1000000 # convert to km²
  d$population <- p; d$type <- 'Neckband'
  return(d)
}))

svf.nb$lower <- 
  Vectorize(function(df){ ctmm:::CI.lower(df,0.05) })(svf.nb$DOF) * svf.nb$SVF
svf.nb$upper <- 
  Vectorize(function(df){ ctmm:::CI.upper(df,0.05) })(svf.nb$DOF) * svf.nb$SVF

#################################################
## GPS birds

# convert tracking data; calculate mean SVF per population
tel.gps <- as.telemetry(all.tracks)
tel.gps <- tel.gps[!is.na(summary(tel.gps)$`interval (hr)`)]
var.gps <- lapply(unique(all.tracks$population), function(p){
  keep <- unique(all.tracks$individual.local.identifier[all.tracks$population==p])
  keep <- keep[keep %in% names(tel.gps)]
  var <- lapply(keep, function(id){variogram(tel.gps[[id]], dt=7%#%'days')})
  return(mean(var))
}); names(var.gps) <- unique(all.tracks$population)

svf.gps <- rbind.fill(lapply(names(var.gps), function(p){
  d <- as.data.frame(var.gps[[p]]@.Data)
  names(d) <- var.gps[[p]]@names
  d$lag <- d$lag / 86400 # convert to days
  d$SVF <- d$SVF / 1000000 # convert to km²
  d$population <- p; d$type <- 'GPS collar'
  return(d)
}))

svf.gps$lower <- 
  Vectorize(function(df){ ctmm:::CI.lower(df,0.05) })(svf.gps$DOF) * svf.gps$SVF
svf.gps$upper <- 
  Vectorize(function(df){ ctmm:::CI.upper(df,0.05) })(svf.gps$DOF) * svf.gps$SVF
```

Code

```
# plot
keep.pop <- c('Hudiksvall', 'Örebro', 'Nyköping', 'Svedala', 'Kristianstad')

d.nb <- svf.nb[svf.nb$population %in% keep.pop & svf.nb$lag <= 3*365,]
d.gps <- svf.gps[svf.gps$population %in% keep.pop & svf.gps$lag <= 3*365,]

ggplot() +
  geom_ribbon(data=d.nb, aes(x=lag, ymin=lower, ymax=upper, fill=type), colour=NA, alpha=0.2) +
  geom_path(data=d.nb, aes(x=lag, y=SVF, colour=type), alpha=0.5) +
  geom_ribbon(data=d.gps, aes(x=lag, ymin=lower, ymax=upper, fill=type), colour=NA, alpha=0.2) +
  geom_path(data=d.gps, aes(x=lag, y=SVF, colour=type), linewidth=1) +
  theme_light() + theme(legend.position='bottom', strip.background = element_rect(fill='white', colour=NA), strip.text=element_text(size=11, colour='black')) +
  facet_wrap(~factor(population, levels=c('Hudiksvall', 'Örebro', 'Nyköping', 'Svedala', 'Kristianstad'), labels=c('N (Hudiksvall)', 'C1 (Örebro)', 'C2 (Nyköping)', 'S1 (Svedala)', 'S2 (Kristianstad)')), scale='free_y', ncol=1) +
  scale_colour_manual(name='', values=c('GPS collar'='orange2', 'Neckband'='purple')) +
  scale_fill_viridis_d(name='', end=0.5, option='C', direction=-1) +
  labs(x='Time lag [days]', y='Semivariance [km²]')
```

Mean and 95% confidence interval of semivariograms averaged across all individuals, per location and tracking method.

Code

```
# ggsave(filename='figure_s1_semivariogram.png', width=140, height=200, unit='mm')
```

## ctmm - Neckband data

Fit continuous-time movement models to the neckband data. Calculate the semivariogram with a lag bin width of 1 day (the function defaults to the median sampling interval), and fit the model with a periodicity of 1 year.

Fitting continuous-time models to observations of neckband birds.

```
check <- summary(tel.nb)
n.obs <- sapply(tel.nb, nrow)

# we need at least three observations per bird for attempting to fit a model
tel.nb <- tel.nb[n.obs>2]

ctmm.nb <- lapply(names(tel.nb), function(id){
  # print(id)
  f.new <- paste0('output/240828_ctmm/neckband/', id, '.RData')
  if(!file.exists(f.new)){
    tel <- tel.nb[[id]]
    var <- variogram(tel, dt=1%#%'day') # compute variogram
    PROTO <- ctmm(mean="periodic", period=c(1 %#% "year"), circle=FALSE, range=T) # set-up periodic model
    GUESS <- ctmm.guess(tel, CTMM=PROTO, var=var, interactive=FALSE) # guess initial parameters
    m <- try(ctmm.select(tel, CTMM=GUESS, trace=1)) # model selection
    if(class(m)=='try-error'){
      m <- NULL
      save(tel, var, m, file=f.new)
    }else{
      save(tel, var, m, file=f.new)
    }
  }else{
  load(f.new)
  }
  return(m)
}); names(ctmm.nb) <- names(tel.nb)

# for how many individuals was model selection successful?
knitr::kable(as.data.frame(table(sapply(ctmm.nb, class))))
```

| Var1 | Freq |
| --- | --- |
| ctmm | 329 |
| NULL | 89 |

Fitting continuous-time models to observations of neckband birds.

```
ctmm.nb <- ctmm.nb[sapply(ctmm.nb, class)=='ctmm']

# summarise which models were selected
models <- rbind.fill(lapply(ctmm.nb, function(m){
  tmp <- summary(m)$name
  data.frame(individual=m@info$identity,
             population=inf$population[inf$steel.ringnumber==m@info$identity],
             model=gsub(' .*', '', tmp),
             isotropic=strsplit(tmp, ' ')[[1]][2],
             harmonic=as.numeric(strsplit(tmp, ' ')[[1]][4]))
}))

knitr::kable(as.data.frame(table(models$model)), caption='Tabulation of which models were selected', col.names = c('Type of model', 'Number of individuals'))
```

Tabulation of which models were selected

| Type of model | Number of individuals |
| --- | --- |
| IID | 117 |
| OU | 39 |
| OUf | 125 |
| OUF | 14 |
| OUΩ | 48 |

Code

```
# quick plot: harmonics of selected models. A harmonic of zero means that the model suggested disregarding the periodicity 
ggplot(models, aes(x=harmonic, y=population)) +
  geom_boxplot() + theme_light()
```

A boxplot showing the harmonics for the period among the selected models. Note that a harmonic of 0 indicates that there was not sufficient support for a periodic model, and that a model without periodicity was a better fit for that individual.

### Model fitting and selection

### Model checks

So, even though we have very few data points to go on for some of those individuals, the selection process still selected a range-restricted movement model more often than not. This suggests it should be feasible to estimate ranges for neckband birds using auto-correlated kernel density estimator. Before committing to the computation of ranges, however, it is worthwhile investigating whether we can draw any preliminary conclusions from the models, based on the parameters included in the models. The number of estimated parameters differs somewhat depending on the selected model - these are the ones we can expect, and how to interpret them:

- \(\tau[position]\), or position auto-correlation. In the case of this study, it represents the time that it takes a goose to cross its entire migratory range.
- \(\tau[velocity]\), or velocity auto-correlation. This parameter can be interpreted as behavioural persistence; here of course related to the annual cycle
- \(\tau\), in case the model cannot distinguish between position and velocity auto-correlation
- area of the estimated range. Note that this is the Gaussian estimate, and not the same as the migratory range derived using aKDEs. The latter takes into account the observed movement as well. This is not meaningful for models that do not have a finite range.
- speed; average speed of the movement process (Gaussian root-mean-square velocity)
- diffusion; again related to the movement process
- rotation/deviation: how much of the mean position of the movement process can be explained by the periodicity of the movement. This parameter is included only in models that have a harmonic > 0
- rotation/speed: as above, but for speed instead of position.Only for models with harmonic > 0

Each model also comes with several estimates for effective sample sizes (degrees of freedom/DOF), which will have implications for the uncertainty of estimates. A DOF for the area parameter for example depends on the estimate for position auto-correlation, as this indicates how many times an individual will have crossed its range during the observation period. But every estimate in **ctmm** comes with confidence intervals, so we can take eventual uncertainties resulting from low effective sample sizes into account.

Prior to visualising the parameter estimates, we have to pull them from the models for the neckband data:

Computing population-level means for the target model parameters (position auto-correlation, velocity auto-correlation, periodicity). Pulling some parameter estimates from the models for neckband birds.

```
# which individuals to keep?
ind.nb <- lapply(c('Hudiksvall', 'Nyköping', 'Örebro'), function(s){
  
  ind <- inf$steel.ringnumber[inf$population==s]
  ind <- ind[ind%in%names(ctmm.nb)]
  
  fit.mod <- ctmm.nb[ind]
  
  # retain only successful models (ctmm objects)
  fit.mod <- fit.mod[sapply(fit.mod, class)=='ctmm']
  names(fit.mod) <- sapply(fit.mod, function(x){x@info$identity})

  # determine for which individuals the model selection suggested ou*-models
  n <- unlist(lapply(fit.mod, function(m)summary(m)$name))
  ou.mod <- unique(names(fit.mod)[!grepl('IID', n)])
  
  dof <- unlist(lapply(ctmm.nb[ou.mod], function(x){summary(x)$DOF[1]}))
  
  return(ou.mod[dof>1]) # keep only OU or related models with an effective size >1
}); names(ind.nb) <- c('Hudiksvall', 'Nyköping', 'Örebro')


#################################################
# meta-analysis of models on the population level
param.mean.nb <- rbind.fill(lapply(c('Hudiksvall', 'Nyköping', 'Örebro'), function(s){
  print(s)

  # which individuals?
  ids <- ind.nb[[s]]
  
  # which individuals to base the total mean position autocorrelation estimates on?
  finite.taupos <- unlist(sapply(ctmm.nb[ids], function(x){
    check <- any(grepl('position', row.names(summary(x)$CI)))
    if(check){
      v <- grep('position', row.names(summary(x)$CI))
      return(summary(x, units=F)$CI[v, 'high'])
    }else{
      return(NA)
    }
  }))
  keep.taupos <- names(finite.taupos)[!is.na(finite.taupos)]
  
  # meta(all.ctmm.nb[keep.taupos], variable='tau position')
  
  # which individuals to base the total mean position autocorrelation estimates on?
  finite.tauv <- unlist(sapply(ctmm.nb[ids], function(x){
    check <- any(grepl('velocity', row.names(summary(x)$CI)))
    if(check){
      v <- grep('velocity', row.names(summary(x)$CI))
      return(summary(x, units=F)$CI[v, 'high'])
    }else{
      return(NA)
    }
  }))
  keep.tauv <- names(finite.tauv)[!is.na(finite.tauv)]
  
  # meta(all.ctmm.nb[keep.tauv], variable='tau velocity')
  
  # which individuals to base the total mean position autocorrelation estimates on?
  finite.period <- unlist(sapply(ctmm.nb[ids], function(x){
    check <- any(grepl('period.1', row.names(summary(x)$CI)))
    if(check){
      v <- grep('period.1', row.names(summary(x)$CI))
      return(summary(x, units=F)$CI[v, 'high'])
    }else{
      return(NA)
    }
  }))
  keep.period <- names(finite.period)[!is.na(finite.period)]
  
  # population-level averages for parameter estimates for OU models
  # param.mean <- get.par.meta(ctmm.nb[ids], variables=c('tau position', 'tau velocity', 'periodicity'))
  param.mean <- rbind(
  get.par.meta(ctmm.nb[keep.taupos], variables='tau position'),
  get.par.meta(ctmm.nb[keep.tauv], variables='tau velocity'),
  get.par.meta(ctmm.nb[keep.period], variables='periodicity'))

  param.mean$type <- 'mean'
  param.mean$method <- 'Neckband'
  param.mean$site <- s
  param.mean$migratory <- s %in% c('Hudiksvall', 'Nyköping', 'Örebro')

  return(param.mean)
  })
)

#################################################
## parameter estimates for individuals

param.nb <- lapply(1:length(ctmm.nb), function(j){

  id <- ctmm.nb[[j]]@info$identity
  param <- summary(ctmm.nb[[j]], units=F)
  summ <- summary(tel.nb[[id]])
  dur.unit <- gsub('.*\\(', '', 
                   gsub('\\)', '', names(summ)[grepl('period', names(summ))]))
  duration <- "days" %#% summ[grepl('period', names(summ))][[1]] %#% dur.unit
  
  n <- dimnames(param$CI)[[1]]
  p <- as.data.frame(param$CI); p$param <- n
  p$param <- gsub('%', '(percent)', p$param)

  loc <- inf$population[inf$steel.ringnumber==id]
  
  model.df <- data.frame(id=summ$identity,
                         location=unique(loc), #site=site,
                         model=gsub(' .*', '', param$name),
                         isotropy=strsplit(param$name, ' ')[[1]][2],
                         harmonic=as.numeric(gsub('.* ', '', param$name)),
                         duration.days=duration, DOF.area=param$DOF['area'],
                         DOF.mean=param$DOF['mean'], DOF.speed=param$DOF['speed'],
                         method='nb')
  
  param.df <- data.frame(id=summ$identity,
                         location=unique(loc), #site=site,
                         model=gsub(' .*', '', param$name),
                         param.name=gsub(' \\(.*', '', p$param), 
                         unit=gsub('.* \\(', '', gsub('\\)', '', p$param)),
                         low=as.vector(p$low),
                         ML=as.vector(p$est),
                         high=as.vector(p$high),
                         dof.mean=unique(param$DOF['mean']),
                         method='nb')
  
  return(list(model.df=model.df, param.df=param.df))
})

model.nb <- rbind.fill(lapply(param.nb, function(tmp){
  tmp$model.df
}))

table(model.nb$model)

param.nb <- rbind.fill(lapply(param.nb, function(tmp){
  tmp$param.df
}))
```

Calculate population-level estimates for space use.

> Note: this next part of the code is placed here for convenience but depends on the results from section @range-estimation! If someone were to replicate this study, one would have to first run the code in the section @range-estimation before running this next part.

Compute population-level averages of UD area and pull out the relevant estimates.

```
#################################################
# meta-analysis of UDs

mean.ud.nb <- rbind.fill(lapply(c('Hudiksvall', 'Nyköping', 'Örebro'), function(s){
  # print(s)
  f.new <- paste0('output/akde_5000/neckband/nb_', s, '.RData')
  load(f.new)
  
  # which individuals?
  ids <- ind.nb[[s]]
  
  # some filtering
  ud <- ud[names(ud) %in% ids]
  ud <- ud[sapply(ud, class)=='UD']
  ud <- ud[sapply(ud, function(x){summary(x)$CI[1,2]})>0]

  param.ud <- get.par.meta.ud(ud)
  param.ud$type <- 'mean'
  param.ud$method <- 'GPS collar'
  param.ud$site <- s
  param.ud$migratory <- s %in% c('Hudiksvall', 'Nyköping', 'Örebro')

  return(param.ud)
}))
```

### Parameter estimates for reporting in the manuscript

Using the *sigfig()*-function for keeping two significant figures:

Get estimates for reporting in the manuscript

```
# get mean estimates with sigfig for reporting

# model parameters
sf.model.nb <- expand.grid(site=c('Hudiksvall', 'Nyköping', 'Örebro', 'total'),
                           par=c('tau position', 'tau velocity', 'periodicity'),
                           KEEP.OUT.ATTRS=F)

sf.model.nb <- ddply(sf.model.nb, c('site', 'par'), function(x){
  # print(paste(x$site, x$par, sep=' - '))
  if(x$site=='total'){
    ids <- unlist(ind.nb)
  }else{
    ids <- ind.nb[[x$site]]
  }
  
  pattern <- ifelse(x$par=='tau position', 'position', 
                    ifelse(x$par=='tau velocity', 'velocity', 'period.1'))
  
  finite.par <- unlist(sapply(ctmm.nb[ids], function(x){
      check <- any(grepl(pattern, row.names(summary(x)$CI)))
      if(check){
        v <- grep(pattern, row.names(summary(x)$CI))
        return(summary(x, units=F)$CI[v, 'high'])
      }else{
        return(NA)
      }
    }))
  keep <- names(finite.par)[!is.na(finite.par)]
  
  if(length(keep)>1){
    v <- suppress_output(enquote(sigfig(meta(ctmm.nb[keep], variable=x$par, plot=F))))
    v <- v[grepl('mean', names(v))]
  }else{
    v <- sigfig(summary(ctmm.nb[[keep]])$CI)
    v <- v[grepl(pattern, names(v))]
  }
  
  x$print <- v
  x$unit <- ifelse(x$par=='periodicity', '%', gsub('\\)', '', strsplit(names(v), ' \\(')[[1]][2]))
  x$sample.size <- length(ids)

  return(x)
})

# space use estimates
sf.ud.nb <- data.frame(site=c('Hudiksvall', 'Nyköping', 'Örebro', 'total'), par='area')

sf.ud.nb <- ddply(sf.ud.nb, c('site', 'par'), function(x){
  # print(paste(x$site, x$par, sep=' - '))
  files <- list.files('output/akde_5000/neckband/', pattern='nb_', full.names=T)
  if(x$site=='total'){
    load(files[1]); ud1 <- ud
    load(files[2]); ud2 <- ud
    load(files[3]); ud3 <- ud
    ud <- c(ud1, ud2, ud3)
    ids <- unlist(ind.nb)
    rm(list=c('ud1', 'ud2', 'ud3'))
  }else{
    load(paste0('output/akde_5000/neckband/nb_', x$site, '.RData'))
    ids <- ind.nb[[x$site]]
  }
  
  # some filtering
  ud <- ud[names(ud) %in% ids]
  ud <- ud[sapply(ud, class)=='UD']
  ud <- ud[sapply(ud, function(x){summary(x)$CI[1,2]})>0]

  if(length(ud)>1){
    v <- suppress_output(enquote(sigfig(meta(ud, variable=x$par, plot=F))))
    v <- v[grepl('mean', names(v))]
  }else{
    v <- sigfig(summary(ctmm.nb[[keep]])$CI)
    v <- v[grepl(pattern, names(v))]
  }
  
  x$print <- v
  x$unit <- ifelse(x$par=='periodicity', '%', gsub('\\)', '', strsplit(names(v), ' \\(')[[1]][2]))
  x$sample.size <- length(ud)

  return(x)
})
```

Code

```
knitr::kable(sf.model.nb, col.names=c('Capture site', 'Parameter', 'Mean estimate (95% CI)', 'Unit', 'Sample size'))
```

Statistical estimates for mean and 95% confidence interval of movement parameters derived for neckband birds, with two significant digits.

| Capture site | Parameter | Mean estimate (95% CI) | Unit | Sample size |
| --- | --- | --- | --- | --- |
| Hudiksvall | tau position | 5.6 (2.2—11.3) | mon | 107 |
| Hudiksvall | tau velocity | 0.21 (0.00—1.4) | yr | 107 |
| Hudiksvall | periodicity | 98.84 (98.37—99.23) | % | 107 |
| Nyköping | tau position | 3.5 (1.0—8.3) | mon | 80 |
| Nyköping | tau velocity | 0.13 (0.00—1.2) | yr | 80 |
| Nyköping | periodicity | 96.82 (95.91—97.62) | % | 80 |
| Örebro | tau position | 0.43 (0.06—1.42) | yr | 19 |
| Örebro | tau velocity | 16 (00—380) | days | 19 |
| Örebro | periodicity | 94.1 (89.7—97.3) | % | 19 |
| total | tau position | 4.9 (2.5—8.5) | mon | 206 |
| total | tau velocity | 0.17 (0.00—1.09) | yr | 206 |
| total | periodicity | 97.53 (96.96—98.05) | % | 206 |

Code

```
knitr::kable(sf.ud.nb[,-2], col.names=c('Capture site', 'Mean estimate (95% CI)', 'Unit', 'Sample size'))
```

Statistical estimates for mean and 95% confidence interval of UD area of neckband birds, with two significant digits.

| Capture site | Mean estimate (95% CI) | Unit | Sample size |
| --- | --- | --- | --- |
| Hudiksvall | 3200000 (0000000—24000000) | km² | 105 |
| Nyköping | 600000 (50000—2500000) | km² | 80 |
| Örebro | 1400000 (0000000—8200000) | km² | 19 |
| total | 1800000 (100000—8100000) | km² | 204 |

## ctmm - GPS data

Repeat the above steps for the GPS-tracking data

### Model fitting and selection

Fitting continuous-time movement models to the GPS data.

```
check <- summary(tel.gps)
n.obs <- sapply(tel.gps, nrow)

tel.gps <- tel.gps[n.obs>2]

ctmm.gps <- lapply(names(tel.gps), function(id){
  # print(id)
  f.new <- paste0('output/240828_ctmm/gps/', id, '.RData')
  if(!file.exists(f.new)){
    tel <- tel.gps[[id]]
    var <- variogram(tel, dt=1%#%'day')
    PROTO <- ctmm(mean="periodic", period=c(1 %#% "year"), circle=FALSE, range=T)
    GUESS <- ctmm.guess(tel, CTMM=PROTO, var=var, interactive=FALSE)
    m <- try(ctmm.select(tel, CTMM=GUESS, trace=1))
    if(class(m)=='try-error'){
      m <- NULL
      save(tel, var, m, file=f.new)
    }else{
      save(tel, var, m, file=f.new)
    }
  }else{
  load(f.new)
  }
  return(m)
}); names(ctmm.gps) <- names(tel.gps)

# for how many individuals was model selection successful?
knitr::kable(as.data.frame(table(sapply(ctmm.gps, class))))
```

| Var1 | Freq |
| --- | --- |
| ctmm | 114 |
| NULL | 5 |

Fitting continuous-time movement models to the GPS data.

```
ctmm.gps <- ctmm.gps[sapply(ctmm.gps, class)=='ctmm']

# summarise which models were selected
models <- rbind.fill(lapply(ctmm.gps, function(m){
  tmp <- summary(m)$name
  data.frame(individual=m@info$identity,
             population=inf$population[inf$steel.ringnumber==m@info$identity],
             model=gsub(' .*', '', tmp),
             isotropic=strsplit(tmp, ' ')[[1]][2],
             harmonic=as.numeric(strsplit(tmp, ' ')[[1]][4]))
}))

knitr::kable(as.data.frame(table(models$model)), caption='Tabulation of which models were selected', col.names = c('Type of model', 'Number of individuals'))
```

Tabulation of which models were selected

| Type of model | Number of individuals |
| --- | --- |
| IID | 3 |
| OU | 50 |
| OUf | 7 |
| OUF | 54 |

Code

```
ggplot(models, aes(x=harmonic, y=population)) +
  geom_boxplot() + theme_light()
```

A boxplot showing the harmonics for the period among the selected models. Note that a harmonic of 0 indicates that there was not sufficient support for a periodic model, and that a model without periodicity was a better fit for that individual.

### Model checks

Pull information from the models

```
param.gps <- lapply(1:length(ctmm.gps), function(j){
  id <- ctmm.gps[[j]]@info$identity
  param <- summary(ctmm.gps[[j]], units=F)
  summ <- summary(tel.gps[[id]])
  dur.unit <- gsub('.*\\(', '', 
                   gsub('\\)', '', names(summ)[grepl('period', names(summ))]))
  duration <- "days" %#% summ[grepl('period', names(summ))][[1]] %#% dur.unit
  
  n <- dimnames(param$CI)[[1]]
  p <- as.data.frame(param$CI); p$param <- n
  p$param <- gsub('%', '(percent)', p$param)

  loc <- inf$population[inf$steel.ringnumber==id]
  if(id=='92V02065'){loc <- 'Hudiksvall'}
  #site <- unique(move.seg$site[move.df$individual.local.identifier==id])
  
  model.df <- data.frame(id=summ$identity,
                         location=loc, #site=site,
                         model=gsub(' .*', '', param$name),
                         isotropy=strsplit(param$name, ' ')[[1]][2],
                         harmonic=as.numeric(gsub('.* ', '', param$name)),
                         duration.days=duration, DOF.area=param$DOF['area'],
                         DOF.mean=param$DOF['mean'], DOF.speed=param$DOF['speed'],
                         method='GPS')
  
  param.df <- data.frame(id=summ$identity,
                         location=loc, #site=site,
                         param.name=gsub(' \\(.*', '', p$param), 
                         unit=gsub('.* \\(', '', gsub('\\)', '', p$param)),
                         low=as.vector(p$low),
                         ML=as.vector(p$est),
                         high=as.vector(p$high),
                         method='GPS')
  
  return(list(model.df=model.df, param.df=param.df))
})

model.gps <- rbind.fill(lapply(param.gps, function(tmp){
  tmp$model.df
}))

table(model.gps$model)

param.gps <- rbind.fill(lapply(param.gps, function(tmp){
  tmp$param.df
}))
```

Compute population-level averages for parameter estimates of interest

Computing population-level means for the target model parameters (position autocorrelation, velocity autocorrelation, periodicity). Pulling some parameter estimates from the models for GPS collar birds.

```
# which individuals to keep?

ind.gps <- lapply(unique(inf$population), function(s){
  
  ind <- inf$steel.ringnumber[inf$population==s]
  ind <- ind[ind%in%names(ctmm.gps)]
  
  fit.mod <- ctmm.gps[ind]
  
  # retain only successful models (ctmm objects)
  fit.mod <- fit.mod[sapply(fit.mod, class)=='ctmm']
  names(fit.mod) <- sapply(fit.mod, function(x){x@info$identity})

  # determine for which individuals the model selection suggested ou*-models
  n <- unlist(lapply(fit.mod, function(m)summary(m)$name))
  ou.mod <- unique(names(fit.mod)[!grepl('IID', n)])
  
  # tracking duration, number of locations, sampling frequency
  tel <- tel.gps[ou.mod]
  tel.df <- data.frame(
    dur=sapply(tel, function(t){diff(as.numeric(range(t@.Data[[1]])))}),
    n.loc=sapply(tel, nrow),
    interval=summary(tel)[,1]
  )
  keep <- row.names(tel.df)[tel.df$interval<48]
  return(keep)
}); names(ind.gps) <- unique(inf$population)

# meta-analysis of models

param.mean.gps <- rbind.fill(lapply(unique(inf$population), function(s){
  
  # which individuals?
  ids <- ind.gps[[s]]
  
  ids <- ids[sapply(ctmm.gps[ids], function(x){summary(x)$DOF['mean']})>10]
  
  # population-level averages for parameter estimates for OU models
  param.mean <- get.par.meta(ctmm.gps[ids], variables=c('tau position', 'tau velocity', 'periodicity'))
  param.mean$type <- 'mean'
  param.mean$method <- 'GPS collar'
  param.mean$site <- s
  param.mean$migratory <- s %in% c('Hudiksvall', 'Nyköping', 'Örebro')

  return(param.mean)
  })
)
```

### Total averages for migratory sites

Above we estimated average model parameters per location and tracking method, but we can also include total averages for all individuals tracked with either of the methods.

You might notice below that velocity auto-correlation can only be computed for a small subset of the neckband individuals. The reason for this is that out of the remaining 206 models, the data from only twelve individuals were actually able to support the velocity auto-correlation; all the remaining ones have less complex models. Sample sizes are recorded for every variable.

Compute averages for all birds tracked with the same method, irrespective of capture site.

```
###################################################################################################
## GPS collars
all.ctmm.gps <- ctmm.gps[unlist(ind.gps[c('Hudiksvall', 'Nyköping', 'Örebro')])]
all.ctmm.gps <- all.ctmm.gps[sapply(all.ctmm.gps, function(x){summary(x)$DOF['mean']})>10]

param.all.gps <- get.par.meta(all.ctmm.gps, variables=c('tau position', 'tau velocity', 'periodicity'))
param.all.gps$type <- 'mean'
param.all.gps$method <- 'GPS collar'
param.all.gps$site <- 'total'
param.all.gps$migratory <- T


###################################################################################################
## Neckbands
all.ctmm.nb <- ctmm.nb[unlist(ind.nb[c('Hudiksvall', 'Nyköping', 'Örebro')])]

# which individuals to base the total mean position autocorrelation estimates on?
finite.taupos <- unlist(sapply(all.ctmm.nb, function(x){
  check <- any(grepl('position', row.names(summary(x)$CI)))
  if(check){
    v <- grep('position', row.names(summary(x)$CI))
    return(summary(x, units=F)$CI[v, 'high'])
  }else{
    return(NA)
  }
}))
keep.taupos <- names(finite.taupos)[!is.na(finite.taupos)]

# meta(all.ctmm.nb[keep.taupos], variable='tau position')

# which individuals to base the total mean position autocorrelation estimates on?
finite.tauv <- unlist(sapply(all.ctmm.nb, function(x){
  check <- any(grepl('velocity', row.names(summary(x)$CI)))
  if(check){
    v <- grep('velocity', row.names(summary(x)$CI))
    return(summary(x, units=F)$CI[v, 'high'])
  }else{
    return(NA)
  }
}))
keep.tauv <- names(finite.tauv)[!is.na(finite.tauv)]

# meta(all.ctmm.nb[keep.tauv], variable='tau velocity')

# which individuals to base the total mean position autocorrelation estimates on?
finite.period <- unlist(sapply(all.ctmm.nb, function(x){
  check <- any(grepl('period.1', row.names(summary(x)$CI)))
  if(check){
    v <- grep('period.1', row.names(summary(x)$CI))
    return(summary(x, units=F)$CI[v, 'high'])
  }else{
    return(NA)
  }
}))
keep.period <- names(finite.period)[!is.na(finite.period)]


# for some reason, my summary function does not work for periodicity, so I have to do it manually here:
all <- expand.grid(variable='periodicity', #, "speed"
                   level=c(0.5, 0.75, 0.95), KEEP.OUT.ATTRS=F)
  
res <- ddply(all, c('variable', 'level'), function(x){
  res <- suppress_output(enquote(as.data.frame(ctmm::meta(all.ctmm.nb[keep.period], variable=x$variable, level=x$level))))
  res$estimate <- gsub(' .*', '', row.names(res)); row.names(res) <- 1:nrow(res)
  return(res)
})
res <- tidyr:::pivot_wider(res, names_from=level, names_prefix='ci_', values_from=3:5)[,-c(7,8)]
res$n <- length(all.ctmm.nb[keep.period])

param.all.nb <- rbind(
  get.par.meta(all.ctmm.nb[keep.taupos], variables='tau position'),
  get.par.meta(all.ctmm.nb[keep.tauv], variables='tau velocity'),
  res)
param.all.nb$type <- 'mean'
param.all.nb$method <- 'Neckband'
param.all.nb$site <- 'total'
param.all.nb$migratory <- T

param.total <- rbind(param.all.gps, param.all.nb)
rm(list=c('param.all.gps', 'param.all.nb', 'all.ctmm.gps', 'all.ctmm.nb'))

save(param.total, file='data/misc/241121_total_averages.RData')
```

### Parameter estimates for reporting in the manuscript

Using the *sigfig()*-function for keeping two significant figures:

Pull estimates for reporting in the manuscript.

```
# get mean estimates with sigfig for reporting

# model parameters
sf.model.gps <- expand.grid(site=c('Hudiksvall', 'Nyköping', 'Örebro', 'Svedala', 'Kristianstad', 'total'),
                           par=c('tau position', 'tau velocity', 'periodicity'),
                           KEEP.OUT.ATTRS=F)

sf.model.gps <- ddply(sf.model.gps, c('site', 'par'), function(x){
  # print(paste(x$site, x$par, sep=' - '))
  if(x$site=='total'){
    ids <- unlist(ind.gps[c('Hudiksvall', 'Nyköping', 'Örebro')])
  }else{
    ids <- ind.gps[[x$site]]
  }
  
  pattern <- ifelse(x$par=='tau position', 'position', 
                    ifelse(x$par=='tau velocity', 'velocity', 'period.1'))
  
  finite.par <- unlist(sapply(ctmm.gps[ids], function(x){
      check <- any(grepl(pattern, row.names(summary(x)$CI)))
      if(check){
        v <- grep(pattern, row.names(summary(x)$CI))
        return(summary(x, units=F)$CI[v, 'high'])
      }else{
        return(NA)
      }
    }))
  keep <- names(finite.par)[!is.na(finite.par)]
  
  if(length(keep)>1){
    m <- suppress_output(enquote(meta(ctmm.gps[keep], variable=x$par, plot=F)))
    v <- sigfig(m[1,])
    names(v) <- row.names(m)[1]
  }else{
    v <- sigfig(summary(ctmm.gps[[keep]])$CI)
    v <- v[grepl(pattern, names(v))]
  }
  
  x$print <- v
  x$unit <- ifelse(x$par=='periodicity', '%', gsub('\\)', '', strsplit(names(v), ' \\(')[[1]][2]))
  x$sample.size <- length(ids)

  return(x)
})

# space use estimates
sf.ud.gps <- data.frame(site=c('Hudiksvall', 'Nyköping', 'Örebro', 'Svedala', 'Kristianstad', 'total'), par='area')

sf.ud.gps <- ddply(sf.ud.gps, c('site', 'par'), function(x){
  print(paste(x$site, x$par, sep=' - '))
  files <- list.files('output/akde_5000/gps/', pattern='gps_', full.names=T)
  if(x$site=='total'){
    load(files[1]); ud1 <- ud
    load(files[3]); ud2 <- ud
    load(files[4]); ud3 <- ud
    ud <- c(ud1, ud2, ud3)
    ids <- unlist(ind.gps)
    rm(list=c('ud1', 'ud2', 'ud3'))
  }else{
    load(paste0('output/akde_5000/gps/gps_', x$site, '.RData'))
    ids <- ind.gps[[x$site]]
  }
  
  # some filtering
  ud <- ud[names(ud) %in% ids]
  ud <- ud[sapply(ud, class)=='UD']
  ud <- ud[sapply(ud, function(x){summary(x)$CI[1,2]})>0]

  if(length(ud)>1){
    v <- suppress_output(enquote(sigfig(meta(ud, variable=x$par, plot=F))))
    v <- v[grepl('mean', names(v))]
  }else{
    v <- sigfig(summary(ctmm.gps[[keep]])$CI)
    v <- v[grepl(pattern, names(v))]
  }
  
  x$print <- v
  x$unit <- ifelse(x$par=='periodicity', '%', gsub('\\)', '', strsplit(names(v), ' \\(')[[1]][2]))
  x$sample.size <- length(ud)

  return(x)
})
```

Code

```
knitr::kable(sf.model.gps, col.names=c('Capture site', 'Parameter', 'Mean estimate (95% CI)', 'Unit', 'Sample size'))
```

Code

```
knitr::kable(sf.ud.gps[,-2], col.names=c('Capture site', 'Mean estimate (95% CI)', 'Unit', 'Sample size'))
```

### Combine results & plot

Combine and reshape the results:

Combining the model estimates from above into a single object for plotting.

```
param.all <- rbind(param.mean.gps, param.mean.nb, param.total)

# param.all$site <- factor(param.all$site, 
#                          levels=c('total', 'Kristianstad', 'Svedala', 'Nyköping', 'Örebro', 'Hudiksvall'), 
#                          labels=c('total', 'Kristianstad', 'Svedala', 'Nyköping', 'Örebro', 'Hudiksvall'))

means <- dlply(param.all, 'variable', function(x){
  v <- unique(x$variable)
  convert <- ifelse(v %in% c('tau position', 'tau velocity'), 86400, ifelse(v=='area', 1000000, 1))
  x[x$estimate=='mean',grep('_ci_', names(x))] <- x[x$estimate=='mean',grep('_ci_', names(x))]/convert
  x[,grep('_ci_', names(x))] <- round(x[,grep('_ci_', names(x))], 2)
  x <- x[,-which(names(x) %in% c('low_ci_0.5', 'low_ci_0.75', 'high_ci_0.5', 'high_ci_0.75'))]
  x <- tidyr:::pivot_wider(x, names_from='estimate', values_from=c('est_ci_0.5', 'low_ci_0.95', 'high_ci_0.95'))
  x <- x[with(x, order(site, method)),c('site', 'method', 'n', 
                                        'est_ci_0.5_mean', 'low_ci_0.95_mean', 'high_ci_0.95_mean',
                                        'est_ci_0.5_CoV²', 'low_ci_0.95_CoV²', 'high_ci_0.95_CoV²',
                                        'est_ci_0.5_CoV', 'low_ci_0.95_CoV', 'high_ci_0.95_CoV')]
  return(x)
})
```

Convert means of parameter estimates from SI-units to units that are more easy to parse

Visual representation of the above:

Set up individual plots, to be printed as one in the chunk below.

```
mean.est$label <- unlist(lapply(mean.est$site, switch, 
                                'Kristianstad'='S2', 'Svedala'='S1', 'Nyköping'='C2', 'Örebro'='C1', 'Hudiksvall'='N', 'total'='total'))

# base plot
base <- ggplot() +
  scale_colour_manual(name='', values=c('GPS collar'='darkorange', 'Neckband'='purple3')) +
  # scale_y_continuous(name='', breaks=seq(1:6), labels=c('total', 'Kristianstad', 'Svedala', 'Nyköping', 'Örebro', 'Hudiksvall'), minor_breaks = NULL) +
  scale_y_continuous(name='', breaks=seq(1:6), labels=c('total', 'S2', 'S1', 'C2', 'C1', 'N'), minor_breaks = NULL) +
  scale_alpha_manual(values=c(0.33, 0.55, 1)) +
  guides(alpha=guide_none()) +
  theme_minimal() +
  facet_wrap(~var) +#, label="label_parsed") +
  theme_geese

###################
# tau position
v <- 'tau position'
axis.x <- scale_x_continuous(
  breaks=c(1, 'day'%#%1%#%'week', 'day'%#%1%#%'month',  'day'%#%1%#%'year', 'day'%#%5%#%'years'), 
  labels=c('1 day', '1 week', '1 month', '1 year', '5 years'),
  limits=c(3.5,1000), trans='log')

a <- base + 
  geom_linerange(data=mean.est[mean.est$variable==v,], aes(y=ypos, colour=method, xmin=low, xmax=high, alpha=level.UD), linewidth=1.5) +
  geom_point(data=mean.est[mean.est$variable==v,], aes(x=est, y=ypos, colour=method), shape=21, fill='white', size=2, stroke=1.25) + 
  geom_hline(data=data.frame(y=seq(1.5, 5.5, 1)), aes(yintercept=y), linetype=c(1, rep(2,4)), colour='grey50') +
  geom_text(data=mean.est[mean.est$variable==v & as.character(mean.est$level.UD)=='0.95',], aes(label=paste0('n=', n), y=ypos, x=550), colour='grey40', size=9, size.unit='pt', hjust=0) +
  axis.x  + theme(axis.title.x=element_blank())

###################
# tau velocity
v <- 'tau velocity'
axis.x <- scale_x_continuous(
  breaks=c('day'%#%1%#%'hour', 1, 'day'%#%1%#%'week', 'day'%#%3%#%'months'), 
  labels=c('1 hour', '1 day', '1 week', '3 months'), 
  limits=c(.1, 1500), trans='log')
labs <- labs(x=expression(paste(tau[v], '(d)')), y='')

b <- base + 
  geom_linerange(data=mean.est[mean.est$variable==v,], aes(y=ypos, colour=method, xmin=low, xmax=high, alpha=level.UD), linewidth=1.5) +
  geom_point(data=mean.est[mean.est$variable==v,], aes(x=est, y=ypos, colour=method), shape=21, fill='white', size=2, stroke=1.25) + 
    geom_hline(data=data.frame(y=seq(1.5, 5.5, 1)), aes(yintercept=y), linetype=c(1, rep(2,4)), colour='grey50') +
  geom_text(data=mean.est[mean.est$variable==v & as.character(mean.est$level.UD)=='0.95',], aes(label=paste0('n=', n), y=ypos, x=650), colour='grey40', size=9, size.unit='pt', hjust=0) +
  axis.x  + theme(axis.title.x=element_blank())

###################
# periodicity
v <- 'periodicity'
axis.x <- scale_x_continuous(breaks=seq(0,100,10), limits=c(55,110))
labs <- labs(x='Periodicity (%)', y='')

c <- base + 
  geom_linerange(data=mean.est[mean.est$variable==v,], aes(y=ypos, colour=method, xmin=low, xmax=high, alpha=level.UD), linewidth=1.5) +
  geom_point(data=mean.est[mean.est$variable==v,], aes(x=est, y=ypos, colour=method), shape=21, fill='white', size=2, stroke=1.25) + 
    geom_hline(data=data.frame(y=seq(1.5, 5.5, 1)), aes(yintercept=y), linetype=c(1, rep(2,4)), colour='grey50') +
  geom_text(data=mean.est[mean.est$variable==v & as.character(mean.est$level.UD)=='0.95',], aes(label=paste0('n=', n), y=ypos, x=102), colour='grey40', size=9, size.unit='pt', hjust=0) +
  axis.x + labs(x='Estimate')

plot.all <- a + b + c + plot_layout(ncol=1, guides='collect') & theme(legend.position='bottom')
# ggsave(plot=plot.all, filename='250113_comparison_ctmm.png', width=80, height=200, unit='mm')
# ggsave(plot=plot.all, filename='250113_comparison_ctmm.png', width=85, height=180, unit='mm')
ggsave(plot=plot.all, filename='figure02_ctmm_estimates.png', width=85, height=180, unit='mm')
```

Code

```
# plot everything in one
a + b + c + plot_layout(ncol=1, guides='collect') & theme(legend.position='bottom')
```

Population level estimates for three parameters estimated by the continuous-time movement models. The figure shows the mean estimate for each population and tracking method separately, including the 50, 75, and 95% confidence intervals around the mean estimates.

Sample sizes for the figure above

```
sample_sizes <- ddply(mean.est, c('site', 'method', 'variable'), function(x){data.frame(n=unique(x$n))})

sample_sizes <- sample_sizes[with(sample_sizes, order(-as.numeric(site))),]

knitr::kable(sample_sizes)
```

|  | site | method | variable | n |
| --- | --- | --- | --- | --- |
| 25 | Hudiksvall | GPS collar | tau position | 8 |
| 26 | Hudiksvall | GPS collar | tau velocity | 8 |
| 27 | Hudiksvall | GPS collar | periodicity | 8 |
| 28 | Hudiksvall | Neckband | tau position | 28 |
| 29 | Hudiksvall | Neckband | tau velocity | 6 |
| 30 | Hudiksvall | Neckband | periodicity | 68 |
| 19 | Örebro | GPS collar | tau position | 33 |
| 20 | Örebro | GPS collar | tau velocity | 33 |
| 21 | Örebro | GPS collar | periodicity | 33 |
| 22 | Örebro | Neckband | tau position | 5 |
| 23 | Örebro | Neckband | tau velocity | 1 |
| 24 | Örebro | Neckband | periodicity | 10 |
| 13 | Nyköping | GPS collar | tau position | 9 |
| 14 | Nyköping | GPS collar | tau velocity | 9 |
| 15 | Nyköping | GPS collar | periodicity | 9 |
| 16 | Nyköping | Neckband | tau position | 15 |
| 17 | Nyköping | Neckband | tau velocity | 5 |
| 18 | Nyköping | Neckband | periodicity | 57 |
| 10 | Svedala | GPS collar | tau position | 20 |
| 11 | Svedala | GPS collar | tau velocity | 20 |
| 12 | Svedala | GPS collar | periodicity | 20 |
| 7 | Kristianstad | GPS collar | tau position | 17 |
| 8 | Kristianstad | GPS collar | tau velocity | 17 |
| 9 | Kristianstad | GPS collar | periodicity | 17 |
| 1 | total | GPS collar | tau position | 50 |
| 2 | total | GPS collar | tau velocity | 50 |
| 3 | total | GPS collar | periodicity | 50 |
| 4 | total | Neckband | tau position | 48 |
| 5 | total | Neckband | tau velocity | 12 |
| 6 | total | Neckband | periodicity | 135 |

Code

```
#################################################
# Neckbands

tmp.ind <- param.nb[param.nb$param.name=='rotation/deviation' & param.nb$method=='nb',]
tmp.ind$population <- 
  ifelse(tmp.ind$location=='Hudiksvall', 'N', 
    ifelse(tmp.ind$location=='Örebro', 'C1', 
      ifelse(tmp.ind$location=='Nyköping', 'C2', 'S')))
tmp.ind <- tmp.ind[tmp.ind$population!='S',]
tmp.ind$id <- factor(tmp.ind$id, levels=tmp.ind$id[order(rank(tmp.ind$ML))])
breaks <- levels(tmp.ind$id)[seq(0, length(tmp.ind$id), 3)]

# only non-IID models with periodicity
tmp.ind <- tmp.ind[tmp.ind$model!='IID',]

tmp.mean <- mean.est[mean.est$level.UD==.5 & mean.est$variable=='periodicity' & mean.est$site=='total' & mean.est$method=='Neckband',]


a <- ggplot(tmp.ind, aes(x=ML, xmin=low, xmax=high, y=id, colour=population)) +
  geom_rect(data=tmp.mean, aes(xmin=low, xmax=high, ymin=0, ymax=length(id)-1), fill='purple3', inherit.aes=F, alpha=.3) +
  geom_vline(data=tmp.mean, aes(xintercept=est), colour='purple3', linewidth=1.2) +
  geom_pointrange(size=.3) +
  scale_colour_manual(name='', values=col) +
  # scale_fill_manual(name='', values=c('nb collar'='darkorange', 'Neckband'='purple3')) +
  scale_alpha_manual(name='', values=c(0.3, 1), drop=FALSE) +
  guides(alpha=guide_none(), fill=guide_none()) +
  scale_x_continuous(limits=c(0,100), breaks=seq(0,100,10)) +
  scale_y_discrete(expand=c(0,0), breaks=breaks) +
  labs(x='Periodicity (%)', title='b) Neckband') +
  theme_geese +
  theme(axis.title.y=element_blank(), axis.text.y=element_blank(), axis.ticks.y=element_blank())

#################################################
# GPS collars

tmp.ind <- param.gps[param.gps$param.name=='rotation/deviation' & param.gps$method=='GPS',]
tmp.ind$population <- 
  ifelse(tmp.ind$location=='Hudiksvall', 'N', 
    ifelse(tmp.ind$location=='Örebro', 'C1', 
      ifelse(tmp.ind$location=='Nyköping', 'C2', 'S')))
tmp.ind <- tmp.ind[tmp.ind$population!='S',]
tmp.ind$id <- factor(tmp.ind$id, levels=tmp.ind$id[order(rank(tmp.ind$ML))])
breaks <- levels(tmp.ind$id)[seq(0, length(tmp.ind$id), 3)]

tmp.mean <- mean.est[mean.est$level.UD==.5 & mean.est$variable=='periodicity' & mean.est$site=='total' & mean.est$method=='GPS collar',]


b <- ggplot(tmp.ind, aes(x=ML, xmin=low, xmax=high, y=id, colour=population)) +
  geom_rect(data=tmp.mean, aes(xmin=low, xmax=high, ymin=0, ymax=length(id)-1), fill='darkorange', inherit.aes=F, alpha=.3) +
  geom_vline(data=tmp.mean, aes(xintercept=est), colour='darkorange', linewidth=1.2) +
  geom_pointrange(size=.3) +
  scale_colour_manual(name='', values=col) +
  # scale_fill_manual(name='', values=c('GPS collar'='darkorange', 'Neckband'='purple3')) +
  scale_alpha_manual(name='', values=c(0.3, 1), drop=FALSE) +
  guides(alpha=guide_none(), fill=guide_none()) +
  scale_x_continuous(limits=c(0,100), breaks=seq(0,100,10)) +
  scale_y_discrete(expand=c(0,0), breaks=breaks) +
  labs(x='Periodicity (%)', title='b) GPS collar') +
  theme_geese +
  theme(axis.title.y=element_blank(), axis.text.y=element_blank(), axis.ticks.y=element_blank())

#################################################
# combine in single plot

a + b + plot_layout(ncol=1, guides='collect') & theme(legend.position='bottom')
```

Mean and individual estimates for periodicity. Shown are mean and 95% CI at both individual (points + whiskers) and population level (line/rectangle in background) for a) coded neckbands and b) GPS collars. Colour of points reflect capture site.

Code

```
ggsave(filename='250710_periodicity_all.png', width=100, height=250, unit='mm')

# percentage of neckband birds within 95% CI of GPS collar total mean
v <- param.nb$ML[param.nb$param.name=='rotation/deviation' & param.nb$model!='IID']
table(v >= tmp.mean$low & v <= tmp.mean$high)
```

```
FALSE  TRUE 
  142     4
```

---

# Range estimation

In this section, we show the estimation of ranges for the entire annual movements. The results in the paper are based on individual range estimates, for which we average the *area parameter* per tracking method and capture site above. However, it is also possible to derive population-level estimates for the UD at the population level, spatially. In the case of this study, this step proved feasible only for some capture sites, and only for GPS collar birds. The computation is demanding, and the data underlying the estimation turned out to be insufficient for some capture sites when it comes to actual migratory movements *per individual*. We have included the code and an example in this document nonetheless, for visualisation purposes and for showing that this could be an option for future studies, if only the data are sufficiently comprehensive.

## Complete annual ranges

### Prepare grid

In this step, we will estimate utilisation distributions for Neckband and GPS collar birds using auto-correlated kernel density estimators.

It will be necessary to estimate all ranges for the same underlying grid (grid cells need to align) as otherwise we cannot make summaries or estimate overlap. Here I will prepare the large-scale grid we will be using, with a spatial resolution of 10 km on this large spatial scale.

Prepare a spatial grid for which to compute the autocorrelated kernel density estimators. The point of doing this is so as to not bias the computation, and to be able to compute averages for groups of individuals.

```
(load('data/daily/240828_neckband_gps_clean.RData'))
all.tracks$type <- 'GPS collar'

# all.tracks <- ddply(all.tracks, 'individual.local.identifier', function(x){
#   x <- x[with(x, order('timestamp')),]
#   return(x)
# })

nb$gps.satellite.count <- NA
nb$type <- 'Neckband'

nb <- ddply(nb, 'neckband.code', function(x){
  bands <- x[,c('population', 'steel.ringnumber', 'LONGITUDE', 'LATITUDE', 
                'age', 'sex', 'transmitter')]
  names(bands)[c(2:4)] <- 
    c('individual.local.identifier', 'location.long', 'location.lat')
  bands$year <- year(x$date); bands$julian <- as.numeric(strftime(x$date, format='%j'))
  bands$type <- 'neckband'
  bands$timestamp <- as.POSIXct(paste(x$OBS_DATE, x$OBS_TIME), 
                                tz='UTC', format='%d/%m/%Y %H:%M')

  if(nrow(bands)>1){
    return(bands)
  }else{return(NULL)}
})

nb <- ddply(nb, 'individual.local.identifier', function(x){
  x <- x[!duplicated(x[,c('timestamp')]),]
  x <- x[with(x, order(timestamp)),]
  return(x)
})

shared <- intersect(names(all.tracks), names(nb))
tel.all <- as.telemetry(rbind(all.tracks[,shared], nb[,shared]))
ex <- extent(tel.all)
ex$x <- round(ex$x/1000 + c(-5000,5000))*1000
ex$y <- round(ex$y/1000 + c(-5000,5000))*1000

proj.all <- tel.all[[1]]@info$projection
grid <- list(dr=c(5000,5000), extent=raster:::extent(ex$x, ex$y))
```

### Range estimation - GPS data

Compute aKDEs for GPS collar birds using the grid defined above.

```
sites <- unique(inf$population)

problematic <- c(names(tel.gps)[summary(tel.gps)[,1]>'hr'%#%0.5%#%'day'], "9269816")

# get individual estimates for each site:
ud.gps <- lapply(all.sites, function(s){
  print(s)
  f.new <- paste0('output/akde_5000/gps/gps_', s, '.RData')
  ids <- inf$steel.ringnumber[inf$transmitter & inf$population==s]
  files <- list.files('output/240828_ctmm/gps/', full.names=T)
  files <- files[unlist(lapply(ids, function(x){grep(x, files)}))]

  if(!file.exists(f.new)){
    # import models
    fit.mod <- lapply(files, function(f){
      load(f); return(m)
    })
    
    # retain only successful models (ctmm objects)
    fit.mod <- fit.mod[sapply(fit.mod, class)=='ctmm']
    
    # assign individual ID to list of models
    names(fit.mod) <- sapply(fit.mod, function(x){x@info$identity})
    # fit.mod <- fit.mod[!names(fit.mod) %in% problematic]
    
    # get model names
    n <- unlist(lapply(fit.mod, function(m)summary(m)$name))
    ou.mod <- unique(names(fit.mod)[!grepl('IID', n)])
    ou.mod <- ou.mod[!is.na(ou.mod)]
    
    mod <- fit.mod[ou.mod]
    tel <- tel.all[ou.mod]
    
    ud <- lapply(ou.mod, function(id){
      akde(tel[[id]], CTMM=mod[[id]], grid=grid)
    }); names(ud) <- ou.mod
    
    save(ud, tel, mod, file=f.new)
    return(ud)
  }else{
    load(f.new)
    return(ud)
  }
}); names(ud.gps) <- all.sites
```

### Range estimation - Neckband data

Compute aKDEs for GPS collar birds using the grid defined above.

```
# get individual estimates for each site:
ud.nb <- lapply(nb.sites, function(s){
  print(s)
  f.new <- paste0('output/akde_5000/neckband/nb_', s, '.RData')
  ids <- inf$steel.ringnumber[inf$population==s]
  files <- list.files('output/240828_ctmm/neckband/', full.names=T)
  files <- files[unlist(lapply(ids, function(x){grep(x, files)}))]
  
  if(!file.exists(f.new)){
    # import models
    fit.mod <- lapply(files, function(f){
      load(f); return(m)
    })
    
    # retain only successful models (ctmm objects)
    fit.mod <- fit.mod[sapply(fit.mod, class)=='ctmm']
    
    # assign individual ID to list of models
    names(fit.mod) <- sapply(fit.mod, function(x){x@info$identity})
    # fit.mod <- fit.mod[!names(fit.mod) %in% problematic]
    
    # get model names
    n <- unlist(lapply(fit.mod, function(m)summary(m)$name))
    ou.mod <- unique(names(fit.mod)[!grepl('IID', n)])
    ou.mod <- ou.mod[!is.na(ou.mod)]
    
    mod <- fit.mod[ou.mod]
    tel <- tel.all[ou.mod]
    
    ud <- lapply(ou.mod, function(id){
      akde(tel[[id]], CTMM=mod[[id]], grid=grid)
    }); names(ud) <- ou.mod
    
    save(ud, tel, mod, file=f.new)
    return(ud)
  }else{
    load(f.new)
    return(ud)
  }

}); names(ud.nb) <- nb.sites
```

### Average migratory range *areas*

With the estimation of ranges complete, we can estimate the average of the area parameter per capture site and method. This won’t tell us anything about the degree of overlap, spatially, between neckband and GPS collar birds, but gives us an indication whether the tracking method affects how large an area we expect an individual to occupy throughout the year.

Average area of the 95% UD, per tracking method and capture site, as well as the total averages per tracking method.

```
#################################################
# meta-analysis of UDs - Neckbands

mean.ud.nb <- rbind.fill(lapply(c('Hudiksvall'), function(s){ #, 'Nyköping', 'Örebro'
  print(s)
  f.new <- paste0('output/akde_5000/neckband/nb_', s, '.RData')
  load(f.new)
  
  # which individuals?
  ids <- ind.nb[[s]]
  
  # some filtering
  ud <- ud[names(ud) %in% ids]
  ud <- ud[sapply(ud, class)=='UD']
  ud <- ud[sapply(ud, function(x){summary(x)$DOF[1]})>1]
  ud <- ud[sapply(ud, function(x){summary(x)$CI[1,2]})>0]

  param.ud <- get.par.meta.ud(ud)
  param.ud$type <- 'mean'
  param.ud$method <- 'Neckband'
  param.ud$site <- s
  param.ud$migratory <- s %in% c('Hudiksvall', 'Nyköping', 'Örebro')

  return(param.ud)
}))

# repeat for all individuals irrespective of capture location
all.akde.nb <- unlist(lapply(c('Hudiksvall', 'Nyköping', 'Örebro'), function(s){
  f.new <- paste0('output/akde_5000/neckband/nb_', s, '.RData')
  load(f.new)
  return(ud[names(ud) %in% ind.nb[[s]]])
}), recursive=F)

all.akde.nb <- all.akde.nb[sapply(all.akde.nb, function(x){summary(x)$DOF[1]})>1]
all.akde.nb <- all.akde.nb[sapply(all.akde.nb, function(x){summary(x)$CI[1,2]})>0]

total.ud.nb <- get.par.meta.ud(all.akde.nb)
total.ud.nb$type <- 'mean'
total.ud.nb$method <- 'Neckband'
total.ud.nb$site <- 'total'
total.ud.nb$migratory <- T


#################################################
# meta-analysis of UDs - GPS collars

mean.ud.gps <- rbind.fill(lapply(unique(inf$population), function(s){
  print(s)
  f.new <- paste0('output/akde_5000/gps/gps_', s, '.RData')
  load(f.new)
  
  # which individuals?
  ids <- ind.gps[[s]]
  
  # some filtering
  ud <- ud[names(ud) %in% ids]
  ud <- ud[sapply(ud, class)=='UD']
  ud <- ud[sapply(ud, function(x){summary(x)$DOF[1]})>1]
  ud <- ud[sapply(ud, function(x){summary(x)$CI[1,2]})>0]

  param.ud <- get.par.meta.ud(ud)
  param.ud$type <- 'mean'
  param.ud$method <- 'GPS collar'
  param.ud$site <- s
  param.ud$migratory <- s %in% c('Hudiksvall', 'Nyköping', 'Örebro')

  return(param.ud)
}))

all.akde.gps <- unlist(lapply(c('Hudiksvall', 'Nyköping', 'Örebro'), function(s){
  f.new <- paste0('output/akde_5000/gps/gps_', s, '.RData')
  load(f.new)
  return(ud[names(ud) %in% ind.gps[[s]]])
}), recursive=F)

all.akde.gps <- all.akde.gps[sapply(all.akde.gps, function(x){summary(x)$DOF[1]})>1]
all.akde.gps <- all.akde.gps[sapply(all.akde.gps, function(x){summary(x)$CI[1,2]})>0]

total.ud.gps <- get.par.meta.ud(all.akde.gps)
total.ud.gps$type <- 'mean'
total.ud.gps$method <- 'GPS collar'
total.ud.gps$site <- 'total'
total.ud.gps$migratory <- T


# save for easy access
save(mean.ud.nb, total.ud.nb, mean.ud.gps, total.ud.gps, file='data/misc/meta_annual_akde.RData')
```

Now we can plot the computed results much as we did for the averaged model estimates:

Code

```
param.ud.all <- rbind(mean.ud.nb, total.ud.nb, mean.ud.gps, total.ud.gps)
rm(list=c('mean.ud.nb', 'total.ud.nb', 'mean.ud.gps', 'total.ud.gps'))

param.ud.all$site <- factor(param.ud.all$site, 
                         levels=c('total', 'Kristianstad', 'Svedala', 'Nyköping', 'Örebro', 'Hudiksvall'),
                         labels=c('total', 'Kristianstad', 'Svedala', 'Nyköping', 'Örebro', 'Hudiksvall'))

means <- dlply(param.ud.all, 'variable', function(x){
  v <- unique(x$variable)
  convert <- ifelse(v %in% c('tau position', 'tau velocity'), 86400, ifelse(v=='area', 1000000, 1))
  x[x$estimate=='mean',grep('_ci_', names(x))] <- x[x$estimate=='mean',grep('_ci_', names(x))]/convert
  x[,grep('_ci_', names(x))] <- round(x[,grep('_ci_', names(x))], 2)
  x <- x[,-which(names(x) %in% c('low_ci_0.5', 'low_ci_0.75', 'high_ci_0.5', 'high_ci_0.75'))]
  x <- tidyr:::pivot_wider(x, names_from='estimate', values_from=c('est_ci_0.5', 'low_ci_0.95', 'high_ci_0.95'))
  # x <- x[with(x, order(site, method)),c('site', 'method', 'n', 
  #                                       'est_ci_0.5_mean', 'low_ci_0.95_mean', 'high_ci_0.95_mean')]#,
                                        #'est_ci_0.5_CoV²', 'low_ci_0.95_CoV²', 'high_ci_0.95_CoV²',
                                        #'est_ci_0.5_CoV', 'low_ci_0.95_CoV', 'high_ci_0.95_CoV')]
  return(x)
})


convert.means <- ddply(param.ud.all, 'variable', function(x){
  v <- unique(x$variable)
  convert <- ifelse(v %in% c('tau position', 'tau velocity'), 86400, ifelse(v=='area', 1000000, 1))
  x[x$estimate=='mean',grep('_ci_', names(x))] <- x[x$estimate=='mean',grep('_ci_', names(x))]/convert
  x <- tidyr:::pivot_longer(x, cols=grep('_ci_', names(x)), names_to='level', values_to='value')
  x$level.UD <- as.numeric(gsub('.*_', '', x$level))
  x$where <- gsub('_.*', '', x$level)
  x <- tidyr:::pivot_wider(x, names_from='where', values_from='value', id_cols=c('method', 'site', 'n', 'estimate', 'level.UD'))
  return(x)
})

convert.means$ypos <- as.numeric(convert.means$site)
convert.means$ypos[convert.means$method=='GPS collar' & convert.means$site %in% nb.sites] <- 
  convert.means$ypos[convert.means$method=='GPS collar' & convert.means$site %in% nb.sites]-0.2
convert.means$ypos[convert.means$method=='Neckband'] <- convert.means$ypos[convert.means$method=='Neckband']+0.2
convert.means$level.UD <- factor(as.numeric(gsub('.*_', '', convert.means$level)), levels=c('0.95', '0.75', '0.5'))

mean.est <- convert.means[convert.means$estimate=='mean',]
mean.est$var <- ifelse(mean.est$variable=='tau position', 'a) Position autocorrelation (d)',
                       ifelse(mean.est$variable=='tau velocity', 'b) Velocity autocorrelation (d)',
                              ifelse(mean.est$variable=='periodicity', 'c) Periodicity (%)', "d) Area of distribution range (km²)")))

v <- 'area'
axis.x <- scale_x_continuous(
  breaks=c(1000,10000,100000,1000000), 
  labels=c('1,000', '10,000', '100,000', '1,000,000'), 
  limits=c(1000,6500000), trans='log10')
labs <- labs(x=expression(paste("Area of 95% aKDE " (km^2) )), title='')

d <- ggplot() +
  scale_colour_manual(name='', values=c('GPS collar'='darkorange', 'Neckband'='purple3')) +
  # scale_y_continuous(name='', breaks=seq(1:6), labels=c('total', 'Kristianstad', 'Svedala', 'Nyköping', 'Örebro', 'Hudiksvall'), minor_breaks = NULL) +
  scale_y_continuous(name='', breaks=seq(1:6), labels=c('total', 'S2', 'S1', 'C2', 'C1', 'N'), minor_breaks = NULL) +
  scale_alpha_manual(values=c(0.33, 0.55, 1)) +
  guides(alpha=guide_none()) +
  theme_light() +
  theme(strip.background = element_rect(colour=NA, fill=NA), 
        strip.text=element_text(colour='black', size=11, hjust = 0),
        axis.title.y=element_blank(),
        panel.grid.major.y=element_line(colour=NA)) +
  geom_linerange(data=mean.est[mean.est$variable==v,], aes(y=ypos, colour=method, xmin=low, xmax=high, alpha=level.UD), linewidth=2) +
  geom_point(data=mean.est[mean.est$variable==v,], aes(x=est, y=ypos, colour=method), shape=21, fill='white', size=2.5, stroke=1.5) + 
  geom_hline(aes(yintercept=1.5), linetype=1) +
  geom_hline(data=data.frame(y=seq(2.5, 5.5, 1)), aes(yintercept=y), linetype=2, colour='grey50') +
  geom_text(data=mean.est[mean.est$variable==v & as.character(mean.est$level.UD)=='0.95',], aes(label=paste0('n=', n), y=ypos, x=1000), colour='grey40', size=9, size.unit='pt', hjust=0) +
  axis.x + labs +
  theme_geese +
  theme(legend.position='none')

print(d)
```

Population-level averages of UD area. Here we show the mean estimate for the area of the 95% UD, including the 50, 75, and 95% confidence interval for that estimate.

Code

```
# plot.all <- a + b + c + d + plot_layout(ncol=2)
ggsave(plot=d, filename='figure03_ud_estimates.png', width=85, height=85, unit='mm')
```

Plot showing individual estimates + total average in a single figure

Code

```
# total means
mean.est <- mean.est[mean.est$site=='total',]

convert <- 1000000

# neckbands
par.ud.nb <- rbind.fill(lapply(c('Hudiksvall', 'Nyköping', 'Örebro'), function(s){
  # print(s)
  (load(paste0('output/akde_5000/neckband/nb_', s, '.RData')))
  par <- rbind.fill(lapply(ud, get.par.ud))
  par$method <- 'Neckband'
  par$population <- s
  return(par)
}))

par.ud.nb$individual <- factor(par.ud.nb$individual, 
                                levels=par.ud.nb$individual[order(rank(par.ud.nb$est_ci_0.5))])
par.ud.nb$population <- factor(par.ud.nb$population, levels=c('Hudiksvall', 'Örebro', 'Nyköping', 'Svedala', 'Kristianstad'),
                                labels=c('N', 'C1', 'C2', 'S1', 'S2'))
par.ud.nb$included <- factor(ifelse(par.ud.nb$dof.area>1, 'yes', 'no'), levels=c('no', 'yes'))

a <- ggplot(par.ud.nb[par.ud.nb$low_ci_0.95>0.0001,], 
            aes(x=est_ci_0.5/convert, xmin=low_ci_0.95/convert, xmax=high_ci_0.95/convert, y=individual, colour=population, alpha=included)) +
  geom_rect(data=mean.est[mean.est$level.UD==.95,], 
            aes(xmin=low, xmax=high, ymin=0, ymax=nrow(par.ud.nb)-1, fill=method),
            inherit.aes=F, alpha=.3) +
  geom_vline(data=mean.est[mean.est$method=='Neckband' & mean.est$level.UD==.5,], aes(xintercept=est), colour='purple3', linewidth=1.2) +
  geom_vline(data=mean.est[mean.est$method=='GPS collar' & mean.est$level.UD==.5,], aes(xintercept=est), colour='darkorange', linewidth=1.2) +
  geom_pointrange(size=.3) +
  scale_colour_manual(name='', values=col) +
  scale_fill_manual(name='', values=c('GPS collar'='darkorange', 'Neckband'='purple3')) +
  scale_alpha_manual(name='', values=c(0.3, 1), drop=FALSE) +
  guides(alpha=guide_none(), fill=guide_none()) +
  scale_x_continuous(breaks=c(10,100,1000,10000,100000,1000000,10000000),
                     labels=c('10', '100', '1,000', '10,000', '100,000', '1,000,000', '10,000,000'), trans='log10', limits=c(5, 155000000)) +
  scale_y_discrete(expand=c(0,0), breaks=levels(par.ud.nb$individual)[seq(0,nrow(par.ud.nb),10)]) +
  labs(x=expression(paste("Area of 95% aKDE " (km^2) )), title='a) Neckband') +
  theme_geese +
  theme(axis.title.y=element_blank(), axis.text.y=element_blank(), axis.ticks.y=element_blank(), panel.grid.minor.y = element_blank())

# gps
par.ud.gps <- rbind.fill(lapply(c('Hudiksvall', 'Nyköping', 'Örebro'), function(s){
  # print(s)
  (load(paste0('output/akde_5000/gps/gps_', s, '.RData')))
  par <- rbind.fill(lapply(ud, get.par.ud))
  par$method <- 'GPS collar'
  par$population <- s
  return(par)
}))

par.ud.gps$individual <- factor(par.ud.gps$individual, 
                                levels=par.ud.gps$individual[order(rank(par.ud.gps$est_ci_0.5))])
par.ud.gps$population <- factor(par.ud.gps$population, levels=c('Hudiksvall', 'Örebro', 'Nyköping', 'Svedala', 'Kristianstad'),
                                labels=c('N', 'C1', 'C2', 'S1', 'S2'))
par.ud.gps$included <- factor(ifelse(par.ud.gps$dof.area>1, 'yes', 'no'), levels=c('no', 'yes'))

b <- ggplot(par.ud.gps[par.ud.gps$est_ci_0.5>0,], 
            aes(x=est_ci_0.5/convert, xmin=low_ci_0.95/convert, xmax=high_ci_0.95/convert, y=individual, colour=population, alpha=included)) +
  geom_rect(data=mean.est[mean.est$level.UD==.95,], 
            aes(xmin=low, xmax=high, ymin=0, ymax=nrow(par.ud.gps)-1, fill=method),
            inherit.aes=F, alpha=.3) +
  geom_vline(data=mean.est[mean.est$method=='Neckband' & mean.est$level.UD==.5,], aes(xintercept=est), colour='purple3', linewidth=1.2) +
  geom_vline(data=mean.est[mean.est$method=='GPS collar' & mean.est$level.UD==.5,], aes(xintercept=est), colour='darkorange', linewidth=1.2) +
  geom_pointrange(size=.3) +
  scale_colour_manual(name='', values=col) +
  scale_fill_manual(name='', values=c('GPS collar'='darkorange', 'Neckband'='purple3')) +
  scale_alpha_manual(name='', values=c(0.3, 1), drop=FALSE) +
  guides(alpha=guide_none(), fill=guide_none()) +
  scale_x_continuous(breaks=c(10,100,1000,10000,100000,1000000,10000000),
                     labels=c('10', '100', '1,000', '10,000', '100,000', '1,000,000', '10,000,000'), trans='log10', limits=c(5, 155000000)) +
  scale_y_discrete(expand=c(0,0), breaks=levels(par.ud.gps$individual)[seq(0,nrow(par.ud.gps),3)]) +
  labs(x=expression(paste("Area of 95% aKDE " (km^2) )), title='b) GPS collar') +
  theme_geese +
  theme(axis.title.y=element_blank(), axis.text.y=element_blank(), axis.ticks.y=element_blank())

a + b + plot_layout(ncol=1, guides='collect') & theme(legend.position='bottom')
```

Code

```
ggsave(filename='250115_area_all.png', width=150, height=250, unit='mm')

# percentage of neckband birds within 95% CI of GPS collar total mean
table(par.ud.nb$est_ci_0.5/convert > mean.est$low[6] & par.ud.nb$est_ci_0.5/convert < mean.est$high[6])/nrow(par.ud.nb)
```

```
    FALSE      TRUE 
0.3963134 0.6036866
```

And finally, population-level means in table form:

Code

```
pos <- convert.means[convert.means$estimate=='mean' & convert.means$variable=='area' & convert.means$method=='GPS collar',]
for(i in 1:nrow(pos)){
  if(is.na(pos$est[i])){
    v <- pos$est[1:i][!is.na(pos$est[1:i])]
    pos$est[i] <- rev(v)[1]
  }
}
pos <- pos[pos$level.UD==.95,]
pos$est <- round(pos$est, 2)
pos$ci.range <- paste(round(pos$low,2), round(pos$high,2), sep=' - ')

knitr::kable(pos[,c('site', 'n', 'est', 'ci.range')], 
             col.names=c('Population', 'Sample size', 'Mean estimate (km^2)', '95% CI'))
```

Mean estimates of population level space use for GPS collar birds, in square kilometers


|  | Population | Sample size | Mean estimate (km^2) | 95% CI |
| --- | --- | --- | --- | --- |
| 39 | Kristianstad | 21 | 30155.58 | 3019.93 - 136954.89 |
| 48 | Svedala | 24 | 12031.64 | 1664.93 - 46275.14 |
| 57 | Hudiksvall | 8 | 149155.18 | 1578.57 - 1264460.2 |
| 66 | Nyköping | 11 | 179075.70 | 2657.94 - 1504486.67 |
| 75 | Örebro | 41 | 179811.72 | 10212.42 - 1031012.51 |
| 84 | total | 60 | 175721.81 | 13782.36 - 877625.17 |

Code

```
pos <- convert.means[convert.means$estimate=='mean' & convert.means$variable=='area' & convert.means$method=='Neckband',]
for(i in 1:nrow(pos)){
  if(is.na(pos$est[i])){
    v <- pos$est[1:i][!is.na(pos$est[1:i])]
    pos$est[i] <- rev(v)[1]
  }
}
pos <- pos[pos$level.UD==.95,]
pos$est <- round(pos$est, 2)
pos$ci.range <- paste(round(pos$low,2), round(pos$high,2), sep=' - ')


knitr::kable(pos[,c('site', 'n', 'est', 'ci.range')], 
             col.names=c('Population', 'Sample size', 'Mean estimate (km^1)', '95% CI'))
```

Mean estimates of population level space use for Neckband birds, in square kilometers


|  | Population | Sample size | Mean estimate (km^1) | 95% CI |
| --- | --- | --- | --- | --- |
| 3 | Hudiksvall | 95 | 569442.8 | 76910.5 - 2168074.5 |
| 12 | Nyköping | 77 | 299248.2 | 68893.65 - 870853.65 |
| 21 | Örebro | 18 | 1121851.5 | 72738.45 - 6012505.08 |
| 30 | total | 190 | 507368.4 | 143260.54 - 1305812.62 |

### Population-level kernel density estimates

Different from the above, the population-level kernel density estimator (pKDE) returns the actual spatial distribution of where you would birds from a particular capture location to spend their time. The coding to do this is quite straightforward, though the actual process is computationally demaning and requires the estimation of a lot of parameters.

> As mentioned above, this section is mostly included for demonstration. Please be aware that executing the code below will be computationally demanding and will take a long time to complete. Please use at your own disgression.

Computation of population-level UDs. Note that this takes a lot of RAM and time to compute, and for our study did not work for every tracking method/capture site as ideally one would need more complete seasonal migrations.

```
# GPS collars

lapply(unique(inf$population)[-2], function(s){
  print(s)
  f.new <- paste('output/pkde_5000/gps/gps_', s, '.RData')
  
  if(!file.exists(f.new)){
    (load(paste0('output/akde_5000/gps/gps_', s, '.RData')))
    
    # keep only actual UD objects
    ud <- ud[sapply(ud, class)=='UD']
    
    # effective sample sizes > 1
    ud <- ud[sapply(ud, function(x){summary(x)$DOF[1]})>1]
    
    print(paste('...based on', length(ud), 'individuals.'))
    
    pop.ud <- ctmm::pkde(data=tel[names(ud)], UD=ud, kernel='individual', grid=grid)
    save(pop.ud, file=paste('output/pkde_5000/gps/gps_', s, '.RData'))
  }
  
  return(file.exists(paste('output/pkde_5000/gps/gps_', s, '.RData')))
})

# Neckbands

lapply(unique(inf$population)[-2], function(s){
  print(s)
  f.new <- paste('output/pkde_5000/gps/gps_', s, '.RData')
  
  if(!file.exists(f.new)){
    (load(paste0('output/akde_5000/gps/gps_', s, '.RData')))
    
    # keep only actual UD objects
    ud <- ud[sapply(ud, class)=='UD']
    
    # effective sample sizes > 1
    ud <- ud[sapply(ud, function(x){summary(x)$DOF[1]})>1]
    
    # area > 0
    ud <- ud[sapply(ud, function(x){summary(x)$CI[1,2]})>0]
    
    print(paste('...based on', length(ud), 'individuals.'))
    
    pop.ud <- ctmm::pkde(data=tel[names(ud)], UD=ud, kernel='individual', grid=grid)
    save(pop.ud, file=paste('output/pkde_5000/gps/gps_', s, '.RData'))
  }
  
  return(file.exists(paste('output/pkde_5000/gps/gps_', s, '.RData')))
})

# her we need to filter out uds with 0 effective sample size and 0 area
```

Here is the result for Nyköping:

Code

```
load('output/pkde_all/gps/gps_Nyköping.RData')

m <- rast(raster(pop.ud, DF='PMF'))

ggplot() +
  geom_sf(data=adm, fill='antiquewhite', colour='grey20', linewidth=0.2) +
  geom_spatraster(data=m, aes(fill=(after_stat(value)))) +
  scale_fill_viridis_c(name='PDF', option='G', na.value=NA, direction=-1, transform='log') +
  theme_map +
  scale_colour_viridis_d(name='', end=0.5, option='C', direction=-1) +
  guides(colour=guide_legend(override.aes = list(size=2.5, alpha=1))) +
  xlim(2,20) +
  ylim(48,60) +
  theme(legend.position='none')
```

The figure shows the (log-transformed) probability mass function of the population-level aKDE for Nyköping birds deployed with GPS collars. Because of the log-transformation, the figures shows only those areas with a non-zero probability mass are highlighted on the map.

Alternative visualisation, showing the contour of the 95% UD (mean and 95% confidence interval).

Code

```
load('output/pkde_all/gps/gps_Nyköping.RData')

m <- as.sf(pop.ud)
m$type <- c('CI', 'mean', 'CI')

ggplot() +
  geom_sf(data=adm, fill='antiquewhite', colour='grey20', linewidth=0.2) +
  geom_sf(data=m, aes(linetype=type), show.legend=FALSE, fill=NA, linewidth=.7, colour=viridisLite::inferno(10)[7]) +
  scale_linetype_manual(values=c(2,1)) +
  theme_map +
  scale_colour_viridis_d(name='', end=0.5, option='C', direction=-1) +
  guides(colour=guide_legend(override.aes = list(size=2.5, alpha=1))) +
  xlim(2,20) +
  ylim(48,60) +
  theme(legend.position='none')
```

Contour of the 95% UD (mean and 95% confidence interval) for GPS-collared birds captured in Nyköping. The solid line indicates the contour for the mean UD, the dashed lines the lower and higher 95% confidence estimates.

# Congruence of full annual range estimates of neckbands and GPS collars

Calculate pairwise overlap between GPS and neckband birds. Note that this does not work for our data due to limitations imposed by too few seasonal migratory movements in the data. We instead applied this approach to summer and winter ranges - see below.

```
load('output/akde_5000/gps/gps_Hudiksvall.RData')
ud.gps <- ud; rm(ud)

load('output/akde_5000/neckband/nb_Hudiksvall.RData')
ud.nb <- ud; rm(ud)

combi <- expand.grid('gps'=names(ud.gps), 'neckband'=names(ud.nb), KEEP.OUT.ATTRS = F)

overlaps <- lapply(1:nrow(combi), function(j){
  ctmm::overlap(list(ud.gps[[combi$gps[j]]], ud.nb[[combi$neckband[j]]]))
})

res <- get.par.meta.ud(overlaps)
res$type <- 'nb-gps'
res$season <- 'summer'
res$site <- s
res$migratory <- s %in% c('Hudiksvall', 'Nyköping', 'Örebro')
```

# Congruence of winter and summer range estimates of neckbands and GPS collars

Due to the limitations on the pKDEs for the full annual range, we have to make our comparisons on a smaller scale, i.e. for the wintering period, and if possible the breeding area (this will depend on the availability of neckband observations during the summer). As the issue derives from the complexity of the model, we will have to fit models specifically to the wintering and breeding area:

## Fit sub-annual models & compute aKDEs

Fit non-periodic models for wintering and summer period. Prior to any decisions of how to define summer and winter, I will use three months each, just to illustrate the process and give preliminary results. This process will be simpler than before, and we will shorten it further by fitting models only to those individuals which we decided to keep for the analyses.

- winter: November, December, January
- summer: May, June, July

### Summer, GPS collars

Fit continuous-time movement models to GPS data collected during the summer. Compute debiased, and not debiased aKDEs using the models.

```
mod.gps.summer <- lapply(names(ind.gps), function(s){
  print(s)
  
  lapply(ind.gps[[s]], function(id){
    # print(id)
    f.new <- paste0('output/ctmm_seasonal/summer/gps/gps_', id, '.RData')
    if(!file.exists(f.new)){
      tel <- tel.all[[id]]
      tel <- tel[month(tel@.Data[[1]])%in%c(5:7),]
      if(nrow(tel)==0){return(NULL)}
      var <- variogram(tel, dt=c(1%#%'day', 1%#%'year')) #
      GUESS <- ctmm.guess(tel, var=var, interactive=FALSE)
      m <- try(ctmm.select(tel, CTMM=GUESS, trace=1))
      if(class(m)=='try-error'){
        m <- NULL
        save(tel, var, m, file=f.new)
      }else{
        save(tel, var, m, file=f.new)
      }
    }else{
      load(f.new)
    }
    return(m)
  })
})

mod.gps.summer <- lapply(mod.gps.summer, function(m){
  m <- m[sapply(m, class)=='ctmm']
  m <- m[sapply(m, function(x){summary(x)$DOF['area']>0})]
  names(m) <- sapply(m, function(x){x@info$identity})
  return(m)
})
names(mod.gps.summer) <- names(ind.gps)

# compute akdes
ud.gps.summer <- lapply(names(mod.gps.summer), function(s){
  print(s)
  ids <- names(mod.gps.summer[[s]])
  
  ud <- lapply(ids, function(id){
    print(id)
    tel <- tel.all[[id]]
    tel <- tel[month(tel@.Data[[1]])%in%c(5:7),]
    if(nrow(tel)>0){
      return(akde(tel, CTMM=mod.gps.summer[[s]][[id]], grid=grid))
    }else{return(NULL)}
  })
  ud <- ud[sapply(ud, class)=='UD']
  names(ud) <- sapply(ud, function(x){x@info$identity})
  
  return(ud)
})
names(ud.gps.summer) <- names(ind.gps)

# compute akdes
ud.gps.summer.bias <- lapply(names(mod.gps.summer), function(s){
  print(s)
  ids <- names(mod.gps.summer[[s]])
  
  ud <- lapply(ids, function(id){
    print(id)
    tel <- tel.all[[id]]
    tel <- tel[month(tel@.Data[[1]])%in%c(5:7),]
    if(nrow(tel)>0){
      return(akde(tel, CTMM=mod.gps.summer[[s]][[id]], grid=grid, debias=F))
    }else{return(NULL)}
  })
  ud <- ud[sapply(ud, class)=='UD']
  names(ud) <- sapply(ud, function(x){x@info$identity})
  
  return(ud)
})
names(ud.gps.summer.bias) <- names(ind.gps)


# save
save(mod.gps.summer, ud.gps.summer, ud.gps.summer.bias, file='data/misc/2411113_gps_summer.RData')
```

### Summer, Neckbands

Fit continuous-time movement models to Neckband data collected during the summer. Compute debiased, and not debiased aKDEs using the models.

```
mod.nb.summer <- lapply(names(ind.nb), function(s){
  print(s)
  
  lapply(ind.nb[[s]], function(id){
    # print(id)
    f.new <- paste0('output/ctmm_seasonal/summer/neckband/nb_', id, '.RData')
    if(!file.exists(f.new)){
      tel <- tel.all[[id]]
      tel <- tel[month(tel@.Data[[1]])%in%c(5:7),]
      if(nrow(tel)==0){return(NULL)}
      var <- variogram(tel, dt=c(1%#%'day', 1%#%'year')) #
      GUESS <- ctmm.guess(tel, var=var, interactive=FALSE)
      m <- try(ctmm.select(tel, CTMM=GUESS, trace=1))
      if(class(m)=='try-error'){
        m <- NULL
        save(tel, var, m, file=f.new)
      }else{
        save(tel, var, m, file=f.new)
      }
    }else{
      load(f.new)
    }
    return(m)
  })
})

mod.nb.summer <- lapply(mod.nb.summer, function(m){
  m <- m[sapply(m, class)=='ctmm']
  m <- m[sapply(m, function(x){summary(x)$DOF['area']>0})]
  names(m) <- sapply(m, function(x){x@info$identity})
  return(m)
})
names(mod.nb.summer) <- names(ind.nb)

# compute akdes
ud.nb.summer <- lapply(names(mod.nb.summer), function(s){
  print(s)
  ids <- names(mod.nb.summer[[s]])
  
  ud <- lapply(ids, function(id){
    # print(id)
    tel <- tel.all[[id]]
    tel <- tel[month(tel@.Data[[1]])%in%c(5:7),]
    akde(tel, CTMM=mod.nb.summer[[s]][[id]], grid=grid)
  })
  names(ud) <- sapply(ud, function(x){x@info$identity})
  
  return(ud)
})
names(ud.nb.summer) <- names(ind.nb)

# compute akdes without debiasing
ud.nb.summer.bias <- lapply(names(mod.nb.summer), function(s){
  print(s)
  ids <- names(mod.nb.summer[[s]])
  
  ud <- lapply(ids, function(id){
    # print(id)
    tel <- tel.all[[id]]
    tel <- tel[month(tel@.Data[[1]])%in%c(5:7),]
    akde(tel, CTMM=mod.nb.summer[[s]][[id]], grid=grid, debias=F)
  })
  names(ud) <- sapply(ud, function(x){x@info$identity})
  
  return(ud)
})
names(ud.nb.summer.bias) <- names(ind.nb)


# save
save(mod.nb.summer, ud.nb.summer, ud.nb.summer.bias, file='data/misc/241113_nb_summer.RData')
```

### Winter, GPS collars

Fit continuous-time movement models to GPS data collected during the winter. Compute debiased, and not debiased aKDEs using the models.

```
mod.gps.winter <- lapply(names(ind.gps), function(s){
  print(s)
  
  lapply(ind.gps[[s]], function(id){
    print(id)
    f.new <- paste0('output/ctmm_seasonal/winter/gps/gps_', id, '.RData')
    if(!file.exists(f.new)){
      tel <- tel.all[[id]]
      tel <- tel[month(tel@.Data[[1]])%in%c(11,12,1),]
      if(nrow(tel)==0){return(NULL)}
      var <- variogram(tel, dt=c(1%#%'day', 1%#%'year')) #
      GUESS <- ctmm.guess(tel, var=var, interactive=FALSE)
      m <- try(ctmm.select(tel, CTMM=GUESS, trace=1))
      if(class(m)=='try-error'){
        m <- NULL
        save(tel, var, m, file=f.new)
      }else{
        save(tel, var, m, file=f.new)
      }
    }else{
      load(f.new)
    }
    return(m)
  })
})

mod.gps.winter <- lapply(mod.gps.winter, function(m){
  m <- m[sapply(m, class)=='ctmm']
  m <- m[sapply(m, function(x){summary(x)$DOF['area']>0})]
  names(m) <- sapply(m, function(x){x@info$identity})
  return(m)
})
names(mod.gps.winter) <- names(ind.gps)

# compute akdes
ud.gps.winter <- lapply(names(mod.gps.winter), function(s){
  print(s)
  ids <- names(mod.gps.winter[[s]])
  
  ud <- lapply(ids, function(id){
    print(id)
    tel <- tel.all[[id]]
    tel <- tel[month(tel@.Data[[1]])%in%c(11,12,1),]
    if(nrow(tel)>0){
      return(akde(tel, CTMM=mod.gps.winter[[s]][[id]], grid=grid))
    }else{return(NULL)}
  })
  ud <- ud[sapply(ud, class)=='UD']
  names(ud) <- sapply(ud, function(x){x@info$identity})
  
  return(ud)
})
names(ud.gps.winter) <- names(ind.gps)

# compute akdes without debiasing
ud.gps.winter.bias <- lapply(names(mod.gps.winter), function(s){
  print(s)
  ids <- names(mod.gps.winter[[s]])
  
  ud <- lapply(ids, function(id){
    print(id)
    tel <- tel.all[[id]]
    tel <- tel[month(tel@.Data[[1]])%in%c(11,12,1),]
    if(nrow(tel)>0){
      return(akde(tel, CTMM=mod.gps.winter[[s]][[id]], grid=grid, debias=F))
    }else{return(NULL)}
  })
  ud <- ud[sapply(ud, class)=='UD']
  names(ud) <- sapply(ud, function(x){x@info$identity})
  
  return(ud)
})
names(ud.gps.winter.bias) <- names(ind.gps)


# save
save(mod.gps.winter, ud.gps.winter, ud.gps.winter.bias, file='data/misc/241113_gps_winter.RData')
```

### Winter, Neckbands

Fit continuous-time movement models to Neckband data collected during the winter. Compute debiased, and not debiased aKDEs using the models.

```
mod.nb.winter <- lapply(names(ind.nb), function(s){
  print(s)
  
  lapply(ind.nb[[s]], function(id){
    # print(id)
    f.new <- paste0('output/ctmm_seasonal/winter/neckband/nb_', id, '.RData')
    if(!file.exists(f.new)){
      tel <- tel.all[[id]]
      tel <- tel[month(tel@.Data[[1]])%in%c(11,12,1),]
      if(nrow(tel)==0){return(NULL)}
      var <- variogram(tel, dt=c(1%#%'day', 1%#%'year')) #
      GUESS <- ctmm.guess(tel, var=var, interactive=FALSE)
      m <- try(ctmm.select(tel, CTMM=GUESS, trace=1))
      if(class(m)=='try-error'){
        m <- NULL
        save(tel, var, m, file=f.new)
      }else{
        save(tel, var, m, file=f.new)
      }
    }else{
      load(f.new)
    }
    return(m)
  })
})

mod.nb.winter <- lapply(mod.nb.winter, function(m){
  m <- m[sapply(m, class)=='ctmm']
  m <- m[sapply(m, function(x){summary(x)$DOF['area']>0})]
  names(m) <- sapply(m, function(x){x@info$identity})
  return(m)
})
names(mod.nb.winter) <- names(ind.nb)

# compute akdes
ud.nb.winter <- lapply(names(mod.nb.winter), function(s){
  print(s)
  ids <- names(mod.nb.winter[[s]])
  
  ud <- lapply(ids, function(id){
    # print(id)
    tel <- tel.all[[id]]
    tel <- tel[month(tel@.Data[[1]])%in%c(11,12,1),]
    akde(tel, CTMM=mod.nb.winter[[s]][[id]], grid=grid)  
  })
  names(ud) <- sapply(ud, function(x){x@info$identity})
  
  return(ud)
})
names(ud.nb.winter) <- names(ind.nb)

# compute akdes without debiasing
ud.nb.winter.bias <- lapply(names(mod.nb.winter), function(s){
  print(s)
  ids <- names(mod.nb.winter[[s]])
  
  ud <- lapply(ids, function(id){
    # print(id)
    tel <- tel.all[[id]]
    tel <- tel[month(tel@.Data[[1]])%in%c(11,12,1),]
    akde(tel, CTMM=mod.nb.winter[[s]][[id]], grid=grid, debias=F)  
  })
  names(ud) <- sapply(ud, function(x){x@info$identity})
  
  return(ud)
})
names(ud.nb.winter.bias) <- names(ind.nb)


# save
save(mod.nb.winter, ud.nb.winter, ud.nb.winter.bias, file='data/misc/241113_nb_winter.RData')
```

## Estimate pairwise overlap

### Overlap between GPS collared birds in the same location

Note: this operation requires a computer with at least 8GB of RAM and will take some time to complete.

Calculate pairwise overlap for all possible combinations of GPS collar birds, per capture location.

```
# Summer

gg.summer <- rbind.fill(lapply(names(ind.gps), function(s){
  print(s)
  
  if(!file.exists(paste0('output/gg_summer_', s, '.RData'))){
    load('data/misc/2411113_gps_summer.RData')
    ud <- ud.gps.summer[[s]]
    rm(list=c('ud.gps.summer', 'ud.gps.summer.bias')); gc()
    
    combi <- expand.grid('id1'=names(ud), 'id2'=names(ud), KEEP.OUT.ATTRS = F)
    combi <- combi[1:(nrow(combi)/2),]
    combi <- combi[combi$id1!=combi$id2,]
    
    overlaps <- lapply(1:nrow(combi), function(j){
      ctmm::overlap(list(ud[[combi$id1[j]]], ud[[combi$id2[j]]]), method='Bhattacharyya')
    })
    check <- suppress_output(enquote(unlist(lapply(1:length(overlaps), function(j){class(try(ctmm::meta(overlaps[j], variable='distance', plot=F)))[1]}))))
    overlaps <- overlaps[check=='matrix']
    
    save(overlaps, file=paste0('output/gg_summer_', s, '.RData'))
  }else{
    load(paste0('output/gg_summer_', s, '.RData'))
  }
  
  overlaps <- overlaps[(sapply(overlaps, function(x){x$DOF[1,2]}))>1]
  overlaps <- overlaps[(sapply(overlaps, function(x){x$CI[,,'est'][1,2]}))>1e-12]
  
  res <- get.par.meta(overlaps, variable='distance')[1,]
  res$type <- 'gps-gps'
  res$season <- 'summer'
  res$site <- s
  res$migratory <- s %in% c('Hudiksvall', 'Nyköping', 'Örebro')
  res$print <- suppress_output(enquote(sigfig(ctmm::meta(overlaps, variable='distance')[1,])))

  return(res)
}))

# Winter
load('data/misc/241113_gps_winter.RData')
# ud <- ud[sapply(ud, function(x){summary(x)$DOF[1]})]
rm(list=c('ud.gps.winter.bias')); gc()

gg.winter <- rbind.fill(lapply(names(ind.gps), function(s){
  print(s)
  
  if(!file.exists(paste0('output/gg_winter_', s, '.RData'))){
    ud <- ud.gps.winter[[s]]
    n <- names(ud)
    # n <- n[-which(n %in% c('92699763'))]
    combi <- expand.grid('id1'=n, 'id2'=n, KEEP.OUT.ATTRS = F)
    combi <- combi[1:(nrow(combi)/2),]
    combi <- combi[combi$id1!=combi$id2,]
    
    overlaps <- lapply(1:nrow(combi), function(j){
      ctmm::overlap(list(ud[[combi$id1[j]]], ud[[combi$id2[j]]]), method='Bhattacharyya')
    })
    check <- suppress_output(enquote(unlist(lapply(1:length(overlaps), function(j){class(try(ctmm::meta(overlaps[j], variable='distance', plot=F)))[1]}))))
    overlaps <- overlaps[check=='matrix']
    
    save(overlaps, file=paste0('output/gg_winter_', s, '.RData'))
  }else{
    load(paste0('output/gg_winter_', s, '.RData'))
  }

  overlaps <- overlaps[(sapply(overlaps, function(x){x$DOF[1,2]}))>1]
  overlaps <- overlaps[(sapply(overlaps, function(x){x$CI[,,'est'][1,2]}))>1e-12]
  
  res <- get.par.meta(overlaps, variables='distance')[1,]
  res$type <- 'gps-gps'
  res$season <- 'winter'
  res$site <- s
  res$migratory <- s %in% c('Hudiksvall', 'Nyköping', 'Örebro')
  res$print <- suppress_output(enquote(sigfig(ctmm::meta(overlaps, variable='distance')[1,])))

  return(res)
}))

overlap.gg <- rbind(gg.summer, gg.winter)
rm(list=c('gg.summer', 'gg.winter'))

save(overlap.gg, file='data/misc/241008_overlap_gg.RData')
```

### Overlap between neckband birds in the same location

Note: this operation requires a computer with at leats 8GB of RAM and will take some time to complete.

Calculate pairwise overlap for all possible combinations of Neckband birds, per capture location.

```
load('data/misc/241113_nb_summer.RData')
rm(list=c('ud.nb.summer.bias')); gc()

# Summer
nn.summer <- rbind.fill(lapply(names(ind.nb), function(s){
  print(s)
  if(!file.exists(paste0('output/nn_summer_', s, '.RData'))){
    ud <- ud.nb.summer[[s]]
    ud <- ud[sapply(ud, function(x){summary(x)$CI[2]})>0]
    
    combi <- expand.grid('id1'=names(ud), 'id2'=names(ud), KEEP.OUT.ATTRS = F)
    combi <- combi[1:(nrow(combi)/2),]
    combi <- combi[combi$id1!=combi$id2,]
    combi <- combi[!combi$id1 %in% c('92V02294', '92V02758') & !combi$id2 %in% c('92V02294', '92V02758'),]
    
    overlaps <- lapply(1:nrow(combi), function(j){
      ctmm::overlap(list(ud[[combi$id1[j]]], ud[[combi$id2[j]]]), method='Bhattacharyya')
    })
    check <- suppress_output(enquote(unlist(lapply(1:length(overlaps), function(j){class(try(ctmm::meta(overlaps[j], variable='distance', plot=F)))[1]}))))
    overlaps <- overlaps[check=='matrix']
    
    save(overlaps, file=paste0('output/nn_summer_', s, '.RData'))
  }else{
    load(paste0('output/nn_summer_', s, '.RData'))
  }

  overlaps <- overlaps[(sapply(overlaps, function(x){x$DOF[1,2]}))>1]
  overlaps <- overlaps[(sapply(overlaps, function(x){x$CI[,,'est'][1,2]}))>1e-12]
  
  res <- get.par.meta(overlaps, variables='distance')[1,]
  res$type <- 'nb-nb'
  res$season <- 'summer'
  res$site <- s
  res$migratory <- s %in% c('Hudiksvall', 'Nyköping', 'Örebro')
  res$print <- suppress_output(enquote(sigfig(ctmm::meta(overlaps, variable='distance')[1,])))

  return(res)
}))

rm(mod.nb.summer)
rm(ud.nb.summer)
gc()

# Winter
load('data/misc/241113_nb_winter.RData')
rm(ud.nb.winter.bias); gc()

nn.winter <- rbind.fill(lapply(names(ind.nb), function(s){
  print(s)
  
  if(!file.exists(paste0('output/nn_winter_', s, '.RData'))){
    ud <- ud.nb.winter[[s]]
    
    ud <- ud[sapply(ud, function(x){summary(x)$CI[2]})>0]
    if(s=='Hudiksvall'){
      ud <- ud[-which(names(ud)%in%c('92V02061', '92V02294'))]
    }
    
    combi <- expand.grid('id1'=names(ud), 'id2'=names(ud), KEEP.OUT.ATTRS = F)
    combi <- combi[1:(nrow(combi)/2),]
    combi <- combi[combi$id1!=combi$id2,]
    
    overlaps <- lapply(1:nrow(combi), function(j){
      ctmm::overlap(list(ud[[combi$id1[j]]], ud[[combi$id2[j]]]), method='Bhattacharyya')
    })
    check <- suppress_output(enquote((lapply(1:length(overlaps), function(j){class(try(ctmm::meta(overlaps[j], variable='distance', plot=F)))[1]}))))
    overlaps <- overlaps[check=='matrix']
    
    save(overlaps, file=paste0('output/nn_winter_', s, '.RData'))
  }else{
    load(paste0('output/nn_winter_', s, '.RData'))
  }

  overlaps <- overlaps[(sapply(overlaps, function(x){x$DOF[1,2]}))>1]
  overlaps <- overlaps[(sapply(overlaps, function(x){x$CI[,,'est'][1,2]}))>1e-12]
  
  res <- get.par.meta(overlaps, variables='distance')[1,]
  res$type <- 'nb-nb'
  res$season <- 'winter'
  res$site <- s
  res$migratory <- s %in% c('Hudiksvall', 'Nyköping', 'Örebro')
  res$print <- suppress_output(enquote(sigfig(ctmm::meta(overlaps, variable='distance')[1,])))

  return(res)
}))

overlap.nn <- rbind(nn.summer, nn.winter)
rm(list=c('nn.summer', 'nn.winter'))

save(overlap.nn, file='data/misc/241008_overlap_nn.RData')
```

### Overlap between GPS and neckband birds in the same location

Note: this operation requires a computer with at leats 8GB of RAM and will take some time to complete.

Calculate pairwise overlap for all possible pairs comprised of one GPS collar and one Neckband bird, per capture location.

```
# Summer
ng.summer <- rbind.fill(lapply(names(ind.nb), function(s){
  print(s)
  if(!file.exists(paste0('output/ng_summer_', s, '.RData'))){
    load('data/misc/2411113_gps_summer.RData')
    ud.gps <- ud.gps.summer[[s]]
    rm(list=c('ud.gps.summer', 'ud.gps.summer.bias')); gc()
    
    load('data/misc/241113_nb_summer.RData')
    ud.nb <- ud.nb.summer[[s]]
    rm(list=c('ud.nb.summer', 'ud.nb.summer.bias')); gc()
    ud.nb <- ud.nb[sapply(ud.nb, function(x){summary(x)$CI[2]})>1e0-12]
    
    combi <- expand.grid('gps'=names(ud.gps), 'neckband'=names(ud.nb), KEEP.OUT.ATTRS = F)
    
    overlaps <- lapply(1:nrow(combi), function(j){
      ctmm::overlap(list(ud.gps[[combi$gps[j]]], ud.nb[[combi$neckband[j]]]), method='Bhattacharyya')
    })
    check <- suppress_output(enquote(unlist(lapply(1:length(overlaps), function(j){class(try(ctmm::meta(overlaps[j], variable='distance', plot=F)))[1]}))))
    overlaps <- overlaps[check=='matrix']
    
    save(overlaps, file=paste0('output/ng_summer_', s, '.RData'))
  }else{
    load(paste0('output/ng_summer_', s, '.RData'))
  }
  
  overlaps <- overlaps[(sapply(overlaps, function(x){x$DOF[1,2]}))>1]
  overlaps <- overlaps[(sapply(overlaps, function(x){x$CI[,,'est'][1,2]}))>0]
  
  res <- get.par.meta(overlaps, variables='distance')[1,]
  res$type <- 'nb-gps'
  res$season <- 'summer'
  res$site <- s
  res$migratory <- s %in% c('Hudiksvall', 'Nyköping', 'Örebro')
  res$print <- suppress_output(enquote(sigfig(ctmm::meta(overlaps, variable='distance')[1,])))

  return(res)
}))

# Winter
ng.winter <- rbind.fill(lapply(names(ind.nb), function(s){
  print(s)
  if(!file.exists(paste0('output/ng_winter_', s, '.RData'))){
    load('data/misc/241113_gps_winter.RData')
    ud.gps <- ud.gps.winter[[s]]
    rm(ud.gps.winter); rm(ud.gps.winter.bias); gc()
    
    load('data/misc/241113_nb_winter.RData')
    ud.nb <- ud.nb.winter[[s]]
    rm(ud.nb.winter); rm(ud.nb.winter.bias); gc()
    ud.nb <- ud.nb[sapply(ud.nb, function(x){summary(x)$CI[2]})>0]
    # ud.nb <- ud.nb[sapply(ud.nb, function(x){summary(x)$DOF[1]})%%1!=0]
    
    combi <- expand.grid('gps'=names(ud.gps), 'neckband'=names(ud.nb), KEEP.OUT.ATTRS = F)
    
    if(s=='Nyköping'){
      combi <- combi[combi$neckband!='92V02159',]
      combi <- combi[!combi$neckband%in%combi$neckband[c(1,21,31,81,91,101,201,341,361,371,381,391,401,421,431,451,471,481,501,521,561)],]
    }
    if(s=='Örebro'){
      combi <- combi[!combi$neckband %in% c('92V01871', '92V01881', '92V01886', '92V02754', '92V02762', '92V02505'),]
    }
    
    overlaps <- lapply(1:nrow(combi), function(j){
      ctmm::overlap(list(ud.gps[[combi$gps[j]]], ud.nb[[combi$neckband[j]]]), method='Bhattacharyya')
    })
    
    save(overlaps, file=paste0('output/ng_winter_', s, '.RData'))
  }else{
    load(paste0('output/ng_winter_', s, '.RData'))
  }

  overlaps <- overlaps[(sapply(overlaps, function(x){x$DOF[1,2]}))>1]
  overlaps <- overlaps[(sapply(overlaps, function(x){x$CI[,,'est'][1,2]}))>1e-12]

  res <- get.par.meta(overlaps, variables='distance')[1,]
  res$type <- 'nb-gps'
  res$season <- 'winter'
  res$site <- s
  res$migratory <- s %in% c('Hudiksvall', 'Nyköping', 'Örebro')
  res$print <- suppress_output(enquote(sigfig(ctmm::meta(overlaps, variable='distance')[1,])))

  return(res)
}))

overlap.ng <- rbind(ng.summer, ng.winter)
rm(list=c('ng.summer', 'ng.winter'))


save(overlap.ng, file='data/misc/241008_overlap_ng.RData')

load('output/ng_winter_Hudiksvall.RData')
```

Average overlap across all sites per method and season

Averaging of overlaps, by season and type of combinations.

```
###################################################################################################
## GPS - GPS

# summer
overlaps <- unlist(lapply(names(ind.nb), function(s){
  load(paste0('output/gg_summer_', s, '.RData'))
  overlaps <- overlaps[(sapply(overlaps, function(x){x$DOF[1,2]}))>1]
  overlaps <- overlaps[(sapply(overlaps, function(x){x$CI[,,'est'][1,2]}))>1e-12]
  return(overlaps)
}), recursive=F)

all.summer.gg <- get.par.meta(overlaps, variables='distance')[1,]
all.summer.gg$type <- 'gps-gps'
all.summer.gg$season <- 'summer'
all.summer.gg$site <- 'total'
all.summer.gg$migratory <- T
all.summer.gg$print <- suppress_output(enquote(sigfig(ctmm::meta(overlaps, variable='distance', trace=FALSE, plot=FALSE)[1,])))
rm(overlaps)

# winter
overlaps <- unlist(lapply(names(ind.nb), function(s){
  load(paste0('output/gg_winter_', s, '.RData'))
  overlaps <- overlaps[(sapply(overlaps, function(x){x$DOF[1,2]}))>1]
  overlaps <- overlaps[(sapply(overlaps, function(x){x$CI[,,'est'][1,2]}))>1e-12]
  return(overlaps)
}), recursive=F)

all.winter.gg <- get.par.meta(overlaps, variables='distance')[1,]
all.winter.gg$type <- 'gps-gps'
all.winter.gg$season <- 'winter'
all.winter.gg$site <- 'total'
all.winter.gg$migratory <- T
all.winter.gg$print <- suppress_output(enquote(sigfig(ctmm::meta(overlaps, variable='distance', trace=FALSE, plot=FALSE)[1,])))
rm(overlaps)


###################################################################################################
## NB-NB

# summer
overlaps <- unlist(lapply(names(ind.nb), function(s){
  load(paste0('output/nn_summer_', s, '.RData'))
  overlaps <- overlaps[(sapply(overlaps, function(x){x$DOF[1,2]}))>1]
  overlaps <- overlaps[(sapply(overlaps, function(x){x$CI[,,'est'][1,2]}))>1e-12]
  return(overlaps)
}), recursive=F)

all.summer.nn <- get.par.meta(overlaps, variables='distance')[1,]
all.summer.nn$type <- 'nb-nb'
all.summer.nn$season <- 'summer'
all.summer.nn$site <- 'total'
all.summer.nn$migratory <- T
all.summer.nn$print <- suppress_output(enquote(sigfig(ctmm::meta(overlaps, variable='distance', trace=FALSE, plot=FALSE)[1,])))
rm(overlaps)

# winter
overlaps <- unlist(lapply(names(ind.nb), function(s){
  load(paste0('output/nn_winter_', s, '.RData'))
  overlaps <- overlaps[(sapply(overlaps, function(x){x$DOF[1,2]}))>1]
  overlaps <- overlaps[(sapply(overlaps, function(x){x$CI[,,'est'][1,2]}))>1e-12]
  return(overlaps)
}), recursive=F)

all.winter.nn <- get.par.meta(overlaps, variables='distance')[1,]
all.winter.nn$type <- 'nb-nb'
all.winter.nn$season <- 'winter'
all.winter.nn$site <- 'total'
all.winter.nn$migratory <- T
all.winter.nn$print <- suppress_output(enquote(sigfig(ctmm::meta(overlaps, variable='distance', trace=FALSE, plot=FALSE)[1,])))
rm(overlaps)

###################################################################################################
## NB - GPS

# summer
overlaps <- unlist(lapply(names(ind.nb), function(s){
  load(paste0('output/ng_summer_', s, '.RData'))
  overlaps <- overlaps[(sapply(overlaps, function(x){x$DOF[1,2]}))>1]
  overlaps <- overlaps[(sapply(overlaps, function(x){x$CI[,,'est'][1,2]}))>1e-12]
  return(overlaps)
}), recursive=F)

all.summer.ng <- get.par.meta(overlaps, variables='distance')[1,]
all.summer.ng$type <- 'nb-gps'
all.summer.ng$season <- 'summer'
all.summer.ng$site <- 'total'
all.summer.ng$migratory <- T
all.summer.ng$print <- suppress_output(enquote(sigfig(ctmm::meta(overlaps, variable='distance', trace=FALSE, plot=FALSE)[1,])))
rm(overlaps)

# winter
overlaps <- unlist(lapply(names(ind.nb), function(s){
  load(paste0('output/ng_winter_', s, '.RData'))
  overlaps <- overlaps[(sapply(overlaps, function(x){x$DOF[1,2]}))>1]
  overlaps <- overlaps[(sapply(overlaps, function(x){x$CI[,,'est'][1,2]}))>1e-12]
  return(overlaps)
}), recursive=F)

all.winter.ng <- get.par.meta(overlaps, variables='distance')[1,]
all.winter.ng$type <- 'nb-gps'
all.winter.ng$season <- 'winter'
all.winter.ng$site <- 'total'
all.winter.ng$migratory <- T
all.winter.ng$print <- suppress_output(enquote(sigfig(ctmm::meta(overlaps, variable='distance', trace=FALSE, plot=FALSE)[1,])))
rm(overlaps)

total <- do.call('rbind', list(all.summer.gg, all.winter.gg,
                               all.summer.nn, all.winter.nn,
                               all.summer.ng, all.winter.ng))

save(total, file='data/misc/250717_overlap_methods_total.RData')
```

### Plot

Code

```
load('data/misc/241008_overlap_gg.RData')
load('data/misc/241008_overlap_nn.RData')
load('data/misc/241008_overlap_ng.RData')
load('data/misc/250717_overlap_methods_total.RData')

overlap.all <- rbind(overlap.gg, overlap.nn, overlap.ng, total)
rm(list=c('overlap.gg', 'overlap.nn', 'overlap.ng', 'total'))

means <- dlply(overlap.all, 'variable', function(x){
  v <- unique(x$variable)
  convert <- 1000000
  x[x$estimate=='mean',grep('_ci_', names(x))] <- x[x$estimate=='mean',grep('_ci_', names(x))]/convert
  x[,grep('_ci_', names(x))] <- round(x[,grep('_ci_', names(x))], 2)
  x <- x[,-which(names(x) %in% c('low_ci_0.5', 'low_ci_0.75', 'high_ci_0.5', 'high_ci_0.75'))]
  x <- tidyr:::pivot_wider(x, names_from='estimate', values_from=c('est_ci_0.5', 'low_ci_0.95', 'high_ci_0.95'))
  x <- x[with(x, order(site, type)),c('site', 'type', 'n')]
  return(x)
})

convert.means <- ddply(overlap.all[overlap.all$estimate=='mean',], 'variable', function(x){
  v <- unique(x$variable)
  # convert <- 1000000
  x[x$estimate=='mean',grep('_ci_', names(x))] <- exp(-1*x[x$estimate=='mean',grep('_ci_', names(x))])
  x <- tidyr:::pivot_longer(x, cols=grep('_ci_', names(x)), names_to='level', values_to='value')
  x$level.UD <- as.numeric(gsub('.*_', '', x$level))
  x$where <- gsub('_.*', '', x$level)
  x <- tidyr:::pivot_wider(x, names_from='where', values_from='value', id_cols=c('type', 'site', 'season', 'n', 'estimate', 'level.UD'))
  return(x)
})

convert.means$site <- factor(convert.means$site, 
                         levels=c('total', 'Kristianstad', 'Svedala', 'Nyköping', 'Örebro', 'Hudiksvall'),
                         labels=c('total', 'Kristianstad', 'Svedala', 'Nyköping', 'Örebro', 'Hudiksvall'))
convert.means$ypos <- as.numeric(convert.means$site)
convert.means$ypos[convert.means$type=='gps-gps'] <- convert.means$ypos[convert.means$type=='gps-gps']-0.4
convert.means$ypos[convert.means$type=='nb-nb'] <- convert.means$ypos[convert.means$type=='nb-nb']+0.4
convert.means$level.UD <- factor(as.numeric(gsub('.*_', '', convert.means$level)), levels=c('0.95', '0.75', '0.5'))

convert.means$type <- factor(convert.means$type, levels=c('nb-nb', 'nb-gps', 'gps-gps'), labels=c('Neckband', 'GPS - Neckband', 'GPS collar'))


base <- ggplot() +
  scale_colour_manual(name='', values=c('GPS collar'='darkorange', 'Neckband'='purple3', 'GPS - Neckband'='#75e0bc')) +
  # scale_y_continuous(name='', breaks=seq(1:5), labels=c('Kristianstad', 'Svedala', 'Nyköping', 'Örebro', 'Hudiksvall'), minor_breaks = NULL) +
  scale_y_continuous(name='', breaks=seq(1:6), labels=c('total', 'S2', 'S1', 'C2', 'C1', 'N'), minor_breaks = NULL) +
  scale_alpha_manual(values=c(0.33, 0.55, 1)) +
  guides(alpha=guide_none()) +
  theme_minimal() +
  theme_light() +
  theme(strip.background = element_rect(colour=NA, fill=NA), 
        strip.text=element_text(colour='black', size=11, hjust = 0),
        axis.title.y=element_blank(),
        panel.grid.major.y=element_line(colour=NA),
        legend.position='bottom')


summer <- base + 
  geom_linerange(data=convert.means[convert.means$season=='summer',], 
                 aes(y=ypos, colour=type, xmin=low, xmax=high, alpha=level.UD), linewidth=2) +
  geom_point(data=convert.means[convert.means$season=='summer' & !is.na(convert.means$est),], 
             aes(x=est, y=ypos, colour=type), shape=21, fill='white', size=2.5, stroke=1.5) + 
  geom_hline(data=data.frame(y=seq(1.5, 5.5, 1)), aes(yintercept=y), linetype=2, colour='grey50') +
  geom_hline(aes(yintercept=1.5), linetype=1) +
  # labs(x=expression(paste("Pairwise distance " (km^2) ))) +
  labs(x='Pairwise overlap') +
  # scale_x_log10(limits=c(1e01, 1e07)) +
  geom_text(data=convert.means[convert.means$season=='summer',], aes(label=paste0('n=', n), y=ypos, x=1), colour='grey40', size=9, size.unit='pt', hjust=0) +
  scale_x_continuous(limits=c(0,1.15), breaks=seq(0,1,.25)) +
  guides(colour=guide_legend(direction='horizontal')) + 
  theme()

winter <- base + 
  geom_linerange(data=convert.means[convert.means$season=='winter',], 
                 aes(y=ypos, colour=type, xmin=low, xmax=high, alpha=level.UD), linewidth=2) +
  geom_point(data=convert.means[convert.means$season=='winter' & !is.na(convert.means$est),], 
             aes(x=est, y=ypos, colour=type), shape=21, fill='white', size=2.5, stroke=1.5) + 
  geom_hline(data=data.frame(y=seq(1.5, 5.5, 1)), aes(yintercept=y), linetype=2, colour='grey50') +
  geom_hline(aes(yintercept=1.5), linetype=1) +
  # labs(x=expression(paste("Pairwise distance " (km^2) ))) +
  labs(x='Pairwise overlap') +
  # scale_x_log10(limits=c(1e01, 1e07)) +
  geom_text(data=convert.means[convert.means$season=='winter',], aes(label=paste0('n=', n), y=ypos, x=1), colour='grey40', size=9, size.unit='pt', hjust=0) +
  scale_x_continuous(limits=c(0,1.15), breaks=seq(0,1,.25)) +
  guides(colour=guide_legend(direction='horizontal')) + 
  theme(axis.text.y=element_blank(), axis.ticks.y=element_blank())

summer + labs(title='a) Summer') + winter  + labs(title='b) Winter') + plot_layout(ncol=2, guides='collect') & theme(legend.position='bottom')
```

Population level estimates for pairwise overlap between individuls for a) all movement data collected during summer (May, June, July) and b) winter (November, December, January).

Code

```
p <- summer + winter + plot_layout(ncol=2, guides='collect') + plot_annotation(tag_levels='a', tag_suffix=')') & theme(legend.position='bottom')

print(p)
```

Population level estimates for pairwise overlap between individuls for a) all movement data collected during summer (May, June, July) and b) winter (November, December, January).

Code

```
ggsave(filename='figure04_congruence.png', width=170, height=140, unit='mm')
```

We can also print out the numbers with two significant digits. Note that the *meta()*-function converts overlap to distance using a -log() (so as to avoid issues by overlap being an interval parameter with bounds [0,1]) at the time of writing (July 2025). This is why we convert the mean estimates returned from the *meta()*-function back to (the geometric average) of overlap. The corresponding conversion is \(e^{-x}\).

Mean estimates for pairwise overlap of the seasonal range for summer. Overlap ranges from 0 (no overlap) to 1 (ranges are identical).

|  | Season | Comparison | Capture site | Mean overlap | 95% CI |
| --- | --- | --- | --- | --- | --- |
| 3 | summer | gps-gps | Hudiksvall (N) | 0.35 | 0.28 – 0.44 |
| 11 | summer | nb-nb | Hudiksvall (N) | 0.19 | 0.17 – 0.22 |
| 17 | summer | nb-gps | Hudiksvall (N) | 0.20 | 0.18 – 0.21 |
| 5 | summer | gps-gps | Örebro (C1) | 0.27 | 0.25 – 0.29 |
| 13 | summer | nb-nb | Örebro (C1) | 0.70 | 0.34 – 0.97 |
| 19 | summer | nb-gps | Örebro (C1) | 0.00 | 0 – 0 |
| 4 | summer | gps-gps | Nyköping (C2) | 0.59 | 0.51 – 0.67 |
| 12 | summer | nb-nb | Nyköping (C2) | 0.28 | 0.25 – 0.3 |
| 18 | summer | nb-gps | Nyköping (C2) | 0.23 | 0.21 – 0.26 |
| 2 | summer | gps-gps | Svedala (S1) | 0.50 | 0.47 – 0.53 |
| 1 | summer | gps-gps | Kristianstad (S2) | 0.29 | 0.27 – 0.32 |
| 23 | summer | gps-gps | Total (S, C1, C2) | 0.29 | 0.27 – 0.31 |
| 25 | summer | nb-nb | Total (S, C1, C2) | 0.23 | 0.22 – 0.25 |
| 27 | summer | nb-gps | Total (S, C1, C2) | 0.24 | 0.22 – 0.25 |

Mean estimates for pairwise overlap of the seasonal range for winter. Overlap ranges from 0 (no overlap) to 1 (ranges are identical).

|  | Season | Comparison | Capture site | Mean overlap | 95% CI |
| --- | --- | --- | --- | --- | --- |
| 8 | winter | gps-gps | Hudiksvall (N) | 0.49 | 0.39 – 0.6 |
| 14 | winter | nb-nb | Hudiksvall (N) | 0.15 | 0.13 – 0.17 |
| 20 | winter | nb-gps | Hudiksvall (N) | 0.02 | 0.01 – 0.03 |
| 10 | winter | gps-gps | Örebro (C1) | 0.05 | 0.03 – 0.06 |
| 16 | winter | nb-nb | Örebro (C1) | 0.01 | 0 – 0.04 |
| 22 | winter | nb-gps | Örebro (C1) | 0.05 | 0.02 – 0.09 |
| 9 | winter | gps-gps | Nyköping (C2) | 0.03 | 0 – 0.2 |
| 15 | winter | nb-nb | Nyköping (C2) | 0.06 | 0.05 – 0.07 |
| 21 | winter | nb-gps | Nyköping (C2) | 0.08 | 0.06 – 0.11 |
| 7 | winter | gps-gps | Svedala (S1) | 0.02 | 0 – 0.09 |
| 6 | winter | gps-gps | Kristianstad (S2) | 0.34 | 0.28 – 0.39 |
| 24 | winter | gps-gps | Total (S, C1, C2) | 0.05 | 0.03 – 0.07 |
| 26 | winter | nb-nb | Total (S, C1, C2) | 0.08 | 0.07 – 0.08 |
| 28 | winter | nb-gps | Total (S, C1, C2) | 0.03 | 0.02 – 0.05 |

## Estimate population-level overlap

### Summer

Compute population-level aKDEs for data collected during the summer, one per capture method and capture site.

```
# compute pkde
load('data/misc/2411113_gps_summer.RData')
rm(ud.gps.summer.bias); gc()
names(ud.gps.summer) <- names(mod.gps.summer)
pkde.gps.summer <- lapply(names(ud.gps.summer), function(s){
  print(s)
  f.new <- paste0('output/pkde_summer/gps/', s, '.RData')
  if(!file.exists(f.new)){
    data <- lapply(tel.all[names(tel.all) %in% names(ud.gps.summer[[s]])], function(tel){
      tel[month(tel@.Data[[1]])%in%c(5:7),]
    })
    UD <- ud.gps.summer[[s]]
    UD <- UD[sapply(UD, function(x){summary(x)$DOF[1]})>0]
    UD <- pkde(data, UD, kernel='population')
    save(UD, file=f.new)
  }else{
    load(f.new)
  }
  return(UD)
}); names(pkde.gps.summer) <- names(ud.gps.summer)
rm(list=c('pkde.gps.summer', 'ud.gps.summer')); gc()

load('data/misc/241113_nb_summer.RData')
rm(ud.nb.summer.bias); gc()
names(ud.nb.summer) <- names(mod.nb.summer)
pkde.nb.summer <- lapply(names(ud.nb.summer), function(s){
  f.new <- paste0('output/pkde_summer/neckband/', s, '.RData')
  if(!file.exists(f.new)){
    data <- lapply(tel.all[names(tel.all) %in% names(ud.nb.summer[[s]])], function(tel){
      tel[month(tel@.Data[[1]])%in%c(5:7),]
    })
    UD <- ud.nb.summer[[s]]
    UD <- UD[sapply(UD, function(x){summary(x)$DOF[1]})>0]
    UD <- pkde(data, UD, kernel='population')
    save(UD, file=f.new)
  }else{
    load(f.new)
  }
  return(UD)
}); names(pkde.nb.summer) <- names(ud.nb.summer)
rm(list=c('pkde.nb.summer', 'ud.nb.summer')); gc()

# overlaps <- lapply(names(pkde.nb.summer), function(s){
#   o <- overlap(pkde.gps.summer[[s]], pkde.nb.summer[[s]])
# })
```

### Winter

Compute population-level aKDEs for data collected during the winter, one per capture method and capture site.

```
# compute pkde
load('data/misc/241113_gps_winter.RData')
rm(ud.gps.winter.bias); gc()
names(ud.gps.winter) <- names(mod.gps.winter)
pkde.gps.winter <- lapply(names(ud.gps.winter), function(s){
  f.new <- paste0('output/pkde_winter/gps/', s, '.RData')
  if(!file.exists(f.new)){
    data <- lapply(tel.all[names(tel.all) %in% names(ud.gps.winter[[s]])], function(tel){
      tel[month(tel@.Data[[1]])%in%c(11,12,1),]
    })
    UD <- ud.gps.winter[[s]]
    UD <- UD[sapply(UD, function(x){summary(x)$DOF[1]})>0]
    UD <- pkde(data, UD, kernel='population')
    save(UD, file=f.new)
  }else{
    load(f.new)
  }
  return(UD)
}); names(pkde.gps.winter) <- names(ud.gps.winter)
rm(list=c('pkde.gps.winter', 'ud.gps.winter')); gc()

load('data/misc/241113_nb_winter.RData')
names(ud.nb.winter) <- names(mod.nb.winter)
pkde.nb.winter <- lapply(names(ud.nb.winter), function(s){
  f.new <- paste0('output/pkde_winter/neckband/', s, '.RData')
  if(!file.exists(f.new)){
    data <- lapply(tel.all[names(tel.all) %in% names(ud.nb.winter[[s]])], function(tel){
      tel[month(tel@.Data[[1]])%in%c(11,12,1),]
    })
    UD <- ud.nb.winter[[s]]
    UD <- UD[sapply(UD, function(x){summary(x)$DOF[1]})>0]
    UD <- pkde(data, UD, kernel='population')
    save(UD, file=f.new)
  }else{
    load(f.new)
  }
  return(UD)
}); names(pkde.nb.winter) <- names(ud.nb.winter)
rm(list=c('pkde.nb.winter', 'ud.nb.winter')); gc()

# overlaps <- lapply(names(pkde.nb.winter), function(s){
#   o <- overlap(pkde.gps.winter[[s]], pkde.nb.winter[[s]])
# })
```

# Congruence between debiased and non-debiased UDs

## GPS

### Summer

Calculate overlap between debiased and not-debiased aKDEs, once per capture site and tracking method. This is the code for GPS data, summer.

```
load('data/misc/2411113_gps_summer.RData')
names(ud.gps.summer) <- names(mod.gps.summer)
names(ud.gps.summer.bias) <- names(mod.gps.summer)

res.gps.summer <- rbind.fill(lapply(names(ud.gps.summer), function(s){
  print(s)
  n <- intersect(names(ud.gps.summer[[s]]), names(ud.gps.summer.bias[[s]]))
  
  overlaps <- lapply(n, function(id){
    overlap(list(ud.gps.summer[[s]][[id]], ud.gps.summer.bias[[s]][[id]]), method='Bhattacharyya')
  })
  overlaps <- overlaps[sapply(overlaps, function(x){x$DOF[1,2]})>1]
  overlaps <- overlaps[(sapply(overlaps, function(x){x$CI[,,'est'][1,2]}))>1e-12]
  
  res <- get.par.meta(overlaps, variables='distance')[1,]
  res$method <- 'GPS collar'
  res$season <- 'Summer'
  res$site <- s
  res$migratory <- s %in% c('Hudiksvall', 'Nyköping', 'Örebro')
  res$print <- sigfig(ctmm::meta(overlaps, variable='distance', 
                                 trace=FALSE, plot=FALSE)[1,])

  return(res)
}))

ud.gps.summer <- unlist(ud.gps.summer[c('Hudiksvall', 'Örebro', 'Nyköping')], recursive=F)
ud.gps.summer.bias <- unlist(ud.gps.summer.bias[c('Hudiksvall', 'Örebro', 'Nyköping')], recursive=F)

total <- lapply(names(ud.gps.summer), function(id){
  overlap(list(ud.gps.summer[[id]], ud.gps.summer.bias[[id]]), method='Bhattacharyya')
})
names(total) <- names(ud.gps.summer)
total <- total[sapply(total, function(x){x$DOF[1,2]})>1]
total <- total[(sapply(total, function(x){x$CI[,,'est'][1,2]}))>1e-12]


# why are population-level means so much higher than the global mean for all individuals? -> skewed distribution of values more impactful when looking at population-level only!
col <- viridis(5); names(col) <- names(mod.gps.summer)
suppress_output(enquote(ctmm::meta(total, 'distance', col=col[gsub('\\..*', '', names(total))], trace=FALSE, plot=FALSE)))

total.o <- get.par.meta(total, variable='distance')[1,]
total.o$method <- 'GPS collar'
total.o$season <- 'Summer'
total.o$site <- 'total'
total.o$migratory <- NA
total.o$print <- suppress_output(enquote(sigfig(ctmm::meta(total, variable='distance', trace=FALSE, plot=FALSE)[1,], digits=2)))

res.gps.summer <- rbind(res.gps.summer, total.o)

save(res.gps.summer, file='output/debiasing/gps_summer.RData')
```

### Winter

Calculate overlap between debiased and not-debiased aKDEs, once per capture site and tracking method. This is the code for GPS data, winter.

```
load('data/misc/241113_gps_winter.RData')
names(ud.gps.winter) <- names(mod.gps.winter)
names(ud.gps.winter.bias) <- names(mod.gps.winter)

res.gps.winter <- rbind.fill(lapply(names(ud.gps.winter), function(s){
  print(s)
  n <- intersect(names(ud.gps.winter[[s]]), names(ud.gps.winter.bias[[s]]))
  
  if(length(n)==0){return(NULL)}
  
  overlaps <- lapply(n, function(id){
    overlap(list(ud.gps.winter[[s]][[id]], ud.gps.winter.bias[[s]][[id]]), method='Bhattacharyya')
  })
  overlaps <- overlaps[sapply(overlaps, function(x){x$DOF[1,2]})>1]
  overlaps <- overlaps[(sapply(overlaps, function(x){x$CI[,,'est'][1,2]}))>1e-12]
  
  res <- get.par.meta(overlaps, variable='distance')[1,]
  res$method <- 'GPS collar'
  res$season <- 'Winter'
  res$site <- s
  res$migratory <- s %in% c('Hudiksvall', 'Nyköping', 'Örebro')
  res$print <- suppress_output(enquote(sigfig(ctmm::meta(overlaps, variable='distance',trace=FALSE, plot=FALSE)[1,], digits=2)))

  return(res)
}))

ud.gps.winter <- unlist(ud.gps.winter, recursive=F)
ud.gps.winter.bias <- unlist(ud.gps.winter.bias, recursive=F)

total <- lapply(names(ud.gps.winter), function(id){
  overlap(list(ud.gps.winter[[id]], ud.gps.winter.bias[[id]]), method='Bhattacharyya')
})

total <- total[sapply(total, function(x){x$DOF[1,2]})>1]
total <- total[(sapply(total, function(x){x$CI[,,'est'][1,2]}))>1e-12]

total.o <- get.par.meta(total, variable='distance')[1,]
total.o$method <- 'GPS collar'
total.o$season <- 'Winter'
total.o$site <- 'total'
total.o$migratory <- NA
total.o$print <- suppress_output(enquote(sigfig(ctmm::meta(total, variable='distance', trace=FALSE, plot=FALSE)[1,], digits=2)))

res.gps.winter <- rbind(res.gps.winter, total.o)

save(res.gps.winter, file='output/debiasing/gps_winter.RData')
```

## Neckbands

### Summer

Calculate overlap between debiased and not-debiased aKDEs, once per capture site and tracking method. This is the code for Neckband data, summer.

```
load('data/misc/241113_nb_summer.RData')
names(ud.nb.summer) <- names(mod.nb.summer)
names(ud.nb.summer.bias) <- names(mod.nb.summer)

res.nb.summer <- rbind.fill(lapply(names(ud.nb.summer), function(s){
  n <- intersect(names(ud.nb.summer[[s]]), names(ud.nb.summer.bias[[s]]))
  
  overlaps <- lapply(n, function(id){
    overlap(list(ud.nb.summer[[s]][[id]], ud.nb.summer.bias[[s]][[id]]), method='Bhattacharyya')
  })
  overlaps <- overlaps[sapply(overlaps, function(x){x$DOF[1,2]})>1]
  overlaps <- overlaps[(sapply(overlaps, function(x){x$CI[,,'est'][1,2]}))>1e-12]
  
  res <- get.par.meta(overlaps, variable='distance')[1,]
  res$method <- 'Neckband'
  res$season <- 'Summer'
  res$site <- s
  res$migratory <- s %in% c('Hudiksvall', 'Nyköping', 'Örebro')
  res$print <- suppress_output(enquote(sigfig(ctmm::meta(overlaps, variable='distance', trace=FALSE, plot=FALSE)[1,], digits=2)))

  return(res)
}))

ud.nb.summer <- unlist(ud.nb.summer, recursive=F)
ud.nb.summer.bias <- unlist(ud.nb.summer.bias, recursive=F)

total <- lapply(names(ud.nb.summer), function(id){
  overlap(list(ud.nb.summer[[id]], ud.nb.summer.bias[[id]]), method='Bhattacharyya')
})
names(total) <- names(ud.nb.summer)
total <- total[sapply(total, function(x){x$DOF[1,2]})>1]
total <- total[(sapply(total, function(x){x$CI[,,'est'][1,2]}))>1e-12]


# why are population-level means so much higher than the global mean for all individuals? -> skewed distribution of values more impactful when looking at population-level only!
col <- viridis(5); names(col) <- names(mod.nb.summer)
suppress_output(enquote(ctmm::meta(total, 'distance', col=col[gsub('\\..*', '', names(total))])))

total.o <- get.par.meta(total, variable='distance')[1,]
total.o$method <- 'Neckband'
total.o$season <- 'Summer'
total.o$site <- 'total'
total.o$migratory <- NA
total.o$print <- suppress_output(enquote(sigfig(ctmm::meta(total, variable='distance', trace=FALSE, plot=FALSE)[1,], digits=2)))

res.nb.summer <- rbind(res.nb.summer, total.o)

save(res.nb.summer, file='output/debiasing/nb_summer.RData')
```

### Winter

Calculate overlap between debiased and not-debiased aKDEs, once per capture site and tracking method. This is the code for Neckband data, winter.

```
load('data/misc/241113_nb_winter.RData')
names(ud.nb.winter) <- names(mod.nb.winter)
names(ud.nb.winter.bias) <- names(mod.nb.winter)

res.nb.winter <- rbind.fill(lapply(names(ud.nb.winter), function(s){
  print(s)
  n <- intersect(names(ud.nb.winter[[s]]), names(ud.nb.winter.bias[[s]]))
  
  if(length(n)==0){return(NULL)}
  
  overlaps <- lapply(n, function(id){
    overlap(list(ud.nb.winter[[s]][[id]], ud.nb.winter.bias[[s]][[id]]), method='Bhattacharyya')
  })
  overlaps <- overlaps[sapply(overlaps, function(x){x$DOF[1,2]})>1]
  overlaps <- overlaps[(sapply(overlaps, function(x){x$CI[,,'est'][1,2]}))>1e-12]
  
  res <- get.par.meta(overlaps, variable='distance')[1,]
  res$method <- 'Neckband'
  res$season <- 'Winter'
  res$site <- s
  res$migratory <- s %in% c('Hudiksvall', 'Nyköping', 'Örebro')
  res$print <- suppress_output(enquote(sigfig(ctmm::meta(overlaps, variable='distance', trace=FALSE, plot=FALSE)[1,], digits=2)))

  return(res)
}))

ud.nb.winter <- unlist(ud.nb.winter, recursive=F)
ud.nb.winter.bias <- unlist(ud.nb.winter.bias, recursive=F)

total <- lapply(names(ud.nb.winter), function(id){
  overlap(list(ud.nb.winter[[id]], ud.nb.winter.bias[[id]]), method='Bhattacharyya')
})
total <- total[sapply(total, function(x){x$DOF[1,2]})>1]
total <- total[(sapply(total, function(x){x$CI[,,'est'][1,2]}))>1e-12]

total.o <- get.par.meta(total, variable='distance')[1,]
total.o$method <- 'Neckband'
total.o$season <- 'Winter'
total.o$site <- 'total'
total.o$migratory <- NA
total.o$print <- suppress_output(enquote(sigfig(ctmm::meta(total, variable='distance', trace=FALSE, plot=FALSE)[1,], digits=2)))

res.nb.winter <- rbind(res.nb.winter, total.o)

save(res.nb.winter, file='output/debiasing/nb_winter.RData')
```

## Results & Plot

Combine results for plotting

```
overlap.all <- rbind(res.gps.summer, res.gps.winter, res.nb.summer, res.nb.winter)
rm(list=c('res.gps.summer', 'res.gps.winter', 'res.nb.summer', 'res.nb.winter'))

means <- dlply(overlap.all, 'variable', function(x){
  v <- unique(x$variable)

  # convert back to geometric overlap
  x[x$estimate=='mean',grep('_ci_', names(x))] <- exp(-1*x[x$estimate=='mean',grep('_ci_', names(x))])
  x[,grep('_ci_', names(x))] <- round(x[,grep('_ci_', names(x))], 2)
  x <- x[,-which(names(x) %in% c('low_ci_0.5', 'low_ci_0.75', 'high_ci_0.5', 'high_ci_0.75'))]
  x <- tidyr:::pivot_wider(x, names_from='estimate', values_from=c('est_ci_0.5', 'low_ci_0.95', 'high_ci_0.95'))
  x <- x[with(x, order(site, method)),c('site', 'method', 'n', 
                                        'est_ci_0.5_mean', 'low_ci_0.95_mean', 'high_ci_0.95_mean')]
  return(x)
})

convert.means <- ddply(overlap.all[overlap.all$estimate=='mean',], 'variable', function(x){
  v <- unique(x$variable)
  x[x$estimate=='mean',grep('_ci_', names(x))] <- exp(-1*x[x$estimate=='mean',grep('_ci_', names(x))])
  x <- tidyr:::pivot_longer(x, cols=grep('_ci_', names(x)), names_to='level', values_to='value')
  x$level.UD <- as.numeric(gsub('.*_', '', x$level))
  x$where <- gsub('_.*', '', x$level)
  x <- tidyr:::pivot_wider(x, names_from='where', values_from='value', id_cols=c('method', 'site', 'season', 'n', 'estimate', 'level.UD'))
  return(x)
})

convert.means$site <- factor(convert.means$site, 
                         levels=c('total', 'Kristianstad', 'Svedala', 'Nyköping', 'Örebro', 'Hudiksvall'),
                         labels=c('total', 'Kristianstad', 'Svedala', 'Nyköping', 'Örebro', 'Hudiksvall'))
convert.means$ypos <- as.numeric(convert.means$site)
convert.means$ypos[convert.means$method=='GPS collar'] <- convert.means$ypos[convert.means$method=='GPS collar']-0.2
convert.means$ypos[convert.means$method=='Neckband'] <- convert.means$ypos[convert.means$method=='Neckband']+0.2
convert.means$level.UD <- factor(as.numeric(gsub('.*_', '', convert.means$level)), levels=c('0.95', '0.75', '0.5'))

base <- ggplot() +
  scale_colour_manual(name='', values=c('GPS collar'='darkorange', 'Neckband'='purple3')) + # '#4AB599'
  scale_x_continuous(limits=c(0.85,1), breaks=seq(.85, 1, .05)) +
  # scale_y_continuous(name='', breaks=seq(1:5), labels=c('Kristianstad', 'Svedala', 'Nyköping', 'Örebro', 'Hudiksvall'), minor_breaks = NULL) +
  scale_y_continuous(name='', breaks=seq(1:6), labels=c('total', 'S2', 'S1', 'C2', 'C1', 'N'), minor_breaks = NULL) +
  scale_alpha_manual(values=c(0.33, 0.55, 1)) +
  guides(alpha=guide_none()) +
  theme_minimal() +
  theme_light() +
  theme(strip.background = element_rect(colour=NA, fill=NA), 
        strip.text=element_text(colour='black', size=11, hjust = 0),
        axis.title.y=element_blank(),
        panel.grid.major.y=element_line(colour=NA),
        legend.position='bottom')


summer <- base + 
  geom_linerange(data=convert.means[convert.means$season=='Summer',], 
                 aes(y=ypos, colour=method, xmin=low, xmax=high, alpha=level.UD), linewidth=2) +
  geom_point(data=convert.means[convert.means$season=='Summer' & !is.na(convert.means$est),], 
             aes(x=est, y=ypos, colour=method), shape=21, fill='white', size=2.5, stroke=1.5) + 
  geom_hline(data=data.frame(y=seq(1.5, 5.5, 1)), aes(yintercept=y), linetype=2, colour='grey50') +
  geom_hline(data=data.frame(y=1.5), aes(yintercept=y), linetype=1, colour='grey50') +
  labs(x='Distance') +
  guides(colour=guide_legend(direction='horizontal')) + 
  theme()

winter <- base + 
  geom_linerange(data=convert.means[convert.means$season=='Winter',], 
                 aes(y=ypos, colour=method, xmin=low, xmax=high, alpha=level.UD), linewidth=2) +
  geom_point(data=convert.means[convert.means$season=='Winter' & !is.na(convert.means$est),], 
             aes(x=est, y=ypos, colour=method), shape=21, fill='white', size=2.5, stroke=1.5) + 
  geom_hline(data=data.frame(y=seq(1.5, 5.5, 1)), aes(yintercept=y), linetype=2, colour='grey50') +
  geom_hline(data=data.frame(y=1.5), aes(yintercept=y), linetype=1, colour='grey50') +
  labs(x='Distance') +
  guides(colour=guide_legend(direction='horizontal')) + 
  theme(axis.text.y=element_blank(), axis.ticks.y=element_blank())

p <- summer + labs(title='a) Summer') + winter  + labs(title='b) Winter') + plot_layout(ncol=2, guides='collect') & theme(legend.position='bottom')

ggsave(plot=p, filename='241113_debiasing_overlap.png', width=166, height=100, unit='mm')

##########################################################################
# alternative, split by tracking method

convert.means$ypos <- as.numeric(convert.means$site)
convert.means$ypos[convert.means$season=='Summer'] <- convert.means$ypos[convert.means$season=='Summer']-0.2
convert.means$ypos[convert.means$season=='Winter'] <- convert.means$ypos[convert.means$season=='Winter']+0.2
convert.means$level.UD <- factor(as.numeric(gsub('.*_', '', convert.means$level)), levels=c('0.95', '0.75', '0.5'))

base <- ggplot() +
  scale_colour_manual(name='', values=c('Summer'="#551E4FFF", 'Winter'="#54C9ADFF")) + # '#4AB599'
  scale_x_continuous(limits=c(0.85,1), breaks=seq(.85, 1, .05)) +
  # scale_y_continuous(name='', breaks=seq(1:5), labels=c('Kristianstad', 'Svedala', 'Nyköping', 'Örebro', 'Hudiksvall'), minor_breaks = NULL) +
  scale_y_continuous(name='', breaks=seq(1:6), labels=c('total', 'S2', 'S1', 'C2', 'C1', 'N'), minor_breaks = NULL) +
  scale_alpha_manual(values=c(0.33, 0.55, 1)) +
  guides(alpha=guide_none()) +
  theme_minimal() +
  theme_light() +
  theme(strip.background = element_rect(colour=NA, fill=NA), 
        strip.text=element_text(colour='black', size=11, hjust = 0),
        axis.title.y=element_blank(),
        panel.grid.major.y=element_line(colour=NA),
        legend.position='bottom')


gps <- base + 
  geom_linerange(data=convert.means[convert.means$method=='GPS collar',], 
                 aes(y=ypos, colour=season, xmin=low, xmax=high, alpha=level.UD), linewidth=2) +
  geom_point(data=convert.means[convert.means$method=='GPS collar' & !is.na(convert.means$est),], 
             aes(x=est, y=ypos, colour=season), shape=21, fill='white', size=2.5, stroke=1.5) + 
  geom_hline(data=data.frame(y=seq(1.5, 5.5, 1)), aes(yintercept=y), linetype=2, colour='grey50') +
  geom_hline(data=data.frame(y=1.5), aes(yintercept=y), linetype=1, colour='grey50') +
  labs(x='Distance') +
  
  guides(colour=guide_legend(direction='horizontal')) + 
  theme()

nb <- base + 
  geom_linerange(data=convert.means[convert.means$method=='Neckband',], 
                 aes(y=ypos, colour=season, xmin=low, xmax=high, alpha=level.UD), linewidth=2) +
  geom_point(data=convert.means[convert.means$method=='Neckband' & !is.na(convert.means$est),], 
             aes(x=est, y=ypos, colour=season), shape=21, fill='white', size=2.5, stroke=1.5) + 
  geom_hline(data=data.frame(y=seq(1.5, 5.5, 1)), aes(yintercept=y), linetype=2, colour='grey50') +
  geom_hline(data=data.frame(y=1.5), aes(yintercept=y), linetype=1, colour='grey50') +
  labs(x='Distance') +
  guides(colour=guide_legend(direction='horizontal')) + 
  theme(axis.text.y=element_blank(), axis.ticks.y=element_blank())

p <- gps + labs(title='a) GPS collars') + nb  + labs(title='b) Neckbands') + plot_layout(ncol=2, guides='collect') & theme(legend.position='bottom')

ggsave(plot=p, filename='241113_debiasing_overlap_alternative.png', width=166, height=100, unit='mm')

###################################################################################################
# yet another alternative: totals only

base <- ggplot() +
  scale_colour_manual(name='', values=c('GPS collar'='darkorange', 'Neckband'='purple3')) + # '#4AB599'
  scale_x_continuous(limits=c(0.9,1), breaks=seq(.9, 1, .02)) +
  scale_alpha_manual(values=c(0.33, 0.55, 1)) +
  guides(alpha=guide_none()) +
  theme_minimal() +
  theme_light() +
  theme(strip.background = element_rect(colour=NA, fill=NA), 
        strip.text=element_text(colour='black', size=11, hjust = 0),
        axis.title.y=element_blank(),
        panel.grid.major.y=element_line(colour=NA),
        legend.position='bottom')


tmp <- convert.means[convert.means$site=='total',]
tmp$season <- factor(tmp$season, levels=c('Winter', 'Summer'))
tmp$ypos <- ifelse(tmp$method=='GPS collar', as.numeric(tmp$season)-.2, as.numeric(tmp$season)+.2)

base + 
  geom_linerange(data=tmp, 
                 aes(y=ypos, colour=method, xmin=low, xmax=high, alpha=level.UD), linewidth=2) +
  geom_point(data=tmp, 
             aes(x=est, y=ypos, colour=method), shape=21, fill='white', size=2.5, stroke=1.5) + 
  scale_y_continuous(breaks=c(1,2), labels=c('Winter', 'Summer')) +
  geom_hline(data=data.frame(y=1.5), aes(yintercept=y), linetype=2, colour='grey50') +
  labs(x='Overlap') +
  geom_text(data=tmp, aes(label=paste0('n=', n), y=ypos, x=.9), colour='grey40', size=9, size.unit='pt', hjust=0) +
  guides(colour=guide_legend(direction='horizontal'))
```

Combine results for plotting

```
# ggsave(filename='241125_debiasing_alternative2.png', width=88, height=80, unit='mm')
ggsave(filename='figure05_ud_bias.png', width=85, height=80, unit='mm')
```

In table form, with two significant figures:

Code

```
tmp <- overlap.all[,c('variable', 'method', 'season', 'n', 'site', 'print')]
tmp$mean <- round(exp(-1*as.numeric(gsub(' .*', '', tmp$print))),2)
tmp$high <- exp(-1*as.numeric(gsub('—.*', '', gsub('.*\\(', '', tmp$print))))
tmp$low <- exp(-1*as.numeric(gsub('.*—', '', gsub('\\)', '', tmp$print))))

tmp$print <- paste(round(tmp$low, 2), round(tmp$high, 2), sep=' -- ')
tmp$site <- factor(tmp$site, levels=c('Hudiksvall', 'Örebro', 'Nyköping', 'Svedala', 'Kristianstad', 'total'),
                   labels=c('Hudiksvall (N)', 'Örebro (C1)', 'Nyköping (C2)', 'Svedala (S1)', 'Kristianstad (S2)', 'Total (S, C1, C2)'))
tmp <- tmp[with(tmp, order(site)),]

knitr::kable(tmp[tmp$season=='Summer',c('season', 'site', 'method', 'mean', 'print')], 
             col.names=c('Season', 'Capture site', 'Tracking method', 'Mean overlap', '95% CI'))
```

|  | Season | Capture site | Tracking method | Mean overlap | 95% CI |
| --- | --- | --- | --- | --- | --- |
| 3 | Summer | Hudiksvall (N) | GPS collar | 0.98 | 0.97 – 0.99 |
| 13 | Summer | Hudiksvall (N) | Neckband | 0.96 | 0.94 – 0.98 |
| 4 | Summer | Örebro (C1) | GPS collar | 0.98 | 0.97 – 0.99 |
| 15 | Summer | Örebro (C1) | Neckband | 0.95 | 0.94 – 0.97 |
| 5 | Summer | Nyköping (C2) | GPS collar | 0.99 | 0.97 – 0.99 |
| 14 | Summer | Nyköping (C2) | Neckband | 0.95 | 0.95 – 0.96 |
| 1 | Summer | Svedala (S1) | GPS collar | 0.97 | 0.97 – 0.98 |
| 2 | Summer | Kristianstad (S2) | GPS collar | 0.99 | 0.98 – 0.99 |
| 6 | Summer | Total (S, C1, C2) | GPS collar | 0.98 | 0.97 – 0.99 |
| 16 | Summer | Total (S, C1, C2) | Neckband | 0.96 | 0.95 – 0.97 |

And winter:

Code

```
knitr::kable(tmp[tmp$season=='Winter',c('season', 'site', 'method', 'mean', 'print')], 
             col.names=c('Season', 'Capture site', 'Tracking method', 'Mean overlap', '95% CI'))
```

|  | Season | Capture site | Tracking method | Mean overlap | 95% CI |
| --- | --- | --- | --- | --- | --- |
| 9 | Winter | Hudiksvall (N) | GPS collar | 0.98 | 0.97 – 0.98 |
| 17 | Winter | Hudiksvall (N) | Neckband | 0.96 | 0.95 – 0.96 |
| 11 | Winter | Örebro (C1) | GPS collar | 0.98 | 0.96 – 0.99 |
| 19 | Winter | Örebro (C1) | Neckband | 0.94 | 0.89 – 0.97 |
| 10 | Winter | Nyköping (C2) | GPS collar | 0.97 | 0.95 – 0.99 |
| 18 | Winter | Nyköping (C2) | Neckband | 0.94 | 0.92 – 0.95 |
| 8 | Winter | Svedala (S1) | GPS collar | 0.98 | 0.97 – 0.99 |
| 7 | Winter | Kristianstad (S2) | GPS collar | 0.98 | 0.97 – 0.99 |
| 12 | Winter | Total (S, C1, C2) | GPS collar | 0.98 | 0.97 – 0.98 |
| 20 | Winter | Total (S, C1, C2) | Neckband | 0.95 | 0.94 – 0.95 |

# Sensitivity to sample size

Or, more accurately: how large of a sample size is necessary for mean estimates to converge around a number? Here we use cross-validation, specifically a leave-out-k algorithm over a range of values for k. This will be as follows:

- choose k, with k<n, where n is the sample size for a group of birds (capture site + tracking method)
- randomly sample k birds from all birds in a group
- compute mean estimate (here: range area) for those k birds

We will replicate these steps several time for each k and group of birds so we get an indication of variance of mean estimates.

In this way, we can get some kind of indication how many birds are necessary to converge around the mean value for all individuals from a given group. I will choose an error of 10% of the actual value as a threshold.

Start by pulling out the individual identifiers for each capture site and tracking method.

```
inf$method <- ifelse(inf$transmitter, 'GPS collar', ifelse(inf$neckband, 'Neckband', 'other'))
individuals <- dlply(inf[inf$method!='other' & inf$population %in% nb.sites,], c('population', 'method'), function(x){
  ids <- unique(x$steel.ringnumber)
  s <- unique(x$population)
  m <- unique(x$method)
  if(m=='GPS collar'){
    return(ind.gps[[s]])
  }else{
    return(ind.nb[[s]])
  }
})
individuals <- individuals[sapply(individuals, length)>0]
```

K-fold cross-validation of the (Gaussian, model-derived) estimate for UD area for varying sample sizes.

```
# how many times do we want to sample k birds randomly?
n <- 100

# for each combination of site & method, apply leave-out-k algorithm to determine area estimate + coefficient of variation:
res.gps <- rbind.fill(lapply(names(individuals), function(x){
  print(x)
  pop <- strsplit(x, '\\.')[[1]][1]
  type <- strsplit(x, '\\.')[[1]][2]
  if(type=='Neckband' & pop %in% c('Svedala', 'Kristianstad')){
    return(NULL)
  }
  ids <- individuals[[x]]
  
  # determine combinations of individuals to test
  do <- unlist(lapply(1:(length(ids)-2), function(j){
    print(j)
    replicate(n, sample(ids, length(ids) - j), simplify=FALSE)
  }), recursive=F)
  
  if(type=='GPS collar'){
    mod <- ctmm.gps[ids]
  }else{
    mod <- ctmm.nb[ids]
  }
  
  test <- rbind.fill(lapply(1:length(do), function(j){
    tmp <- suppress_output(enquote(as.data.frame(ctmm::meta(mod[do[[j]]], variable='area', plot=F, trace=F))))
    tmp$var <- row.names(tmp); tmp$combination <- j; 
    tmp$sample.size <- length(do[[j]]); tmp$site <- pop; tmp$method <- type
    return(tmp)
  }))

}))

save(res, file='data/misc/cross_validation_k100.RData')
```

Pull the results from the cross-validation and make plot.

```
load('data/misc/cross_validation_k100.RData')

res <- res[res$site %in% c('Hudiksvall', 'Örebro', 'Nyköping'),]
res <- res[res$var=="mean (km²)",]

global.mean <- rbind.fill(lapply(c('Hudiksvall', 'Örebro', 'Nyköping'), function(x){
  rbind.fill(lapply(c('Neckband', 'GPS collar'), function(y){
    if(y=='GPS collar'){
      ids <- ind.gps[[x]]
      mod <- ctmm.gps[ids]
    }else{
      ids <- ind.nb[[x]]
      mod <- ctmm.nb[ids]
    }
    
    m <- suppress_output(enquote(as.data.frame(ctmm::meta(mod, 'area', plot=F, trace=F))[1,]))
    m$site <- x; m$method <- y
    return(m)
  }))
}))

ggplot(res, aes(x=sample.size, y=est, colour=method)) + 
  geom_point(data=res[res$method=='Neckband' & res$var=="mean (km²)",], alpha=0.3, size=0.5) + 
  geom_point(data=res[res$method=='GPS collar' & res$var=="mean (km²)",], alpha=0.3, size=0.5) + 
  geom_hline(data=global.mean, aes(yintercept=est, colour=method), linetype=1, linewidth=0.7) +
  geom_hline(data=global.mean, aes(yintercept=low, colour=method), linetype=2, linewidth=0.7) +
  geom_hline(data=global.mean, aes(yintercept=high, colour=method), linetype=2, linewidth=0.7) +
  scale_colour_manual(name='', values=c('GPS collar'='orange', 'Neckband'='purple')) +
  guides(colour=guide_none()) +
  theme_light() + 
  theme(strip.background=element_rect(fill='white', colour='white'), 
        strip.text=element_text(size=10, colour='black', hjust = 0)) +
  facet_wrap(~factor(site, labels=c('a) Hudiksvall', 'b) Örebro', 'c) Nyköping')), ncol=1) + 
  scale_y_log10() +
  labs(x='Number of individuals', y=expression(paste("Size of distribution range " (km^2) )))
```

Pull the results from the cross-validation and make plot.

```
ggsave(filename='figure_S_sample_size.png', width=85, height=160, unit='mm')
```

Now we will use the results from the cross-validation to determine which sample size is required for achieving good estimates for the average distribution range of the population. We chose that being within 10% of the global mean derived from all individuals we have tracking data for can be considered a good estimate.

The next steps thus are:

- calculate the deviation from the corresponding global mean for each randomised mean, in %
- visually show how many birds, on average, are needed to be within 10% of the global mean

For each sample size in the cross-validation, calculate how the respective mean deviates from the mean derived from all individuals for a given cpature site and tracking method.

```
# calculate deviation from the global mean estimate in %
tmp <- ddply(res, c('method', 'site', 'sample.size'), function(x){
  global.est <- global.mean[global.mean$method==unique(x$method) & global.mean$site==unique(x$site),]
  
  deviation <- data.frame(
    low=(x$low - global.est$low)/global.est$low * 100,
    est=(x$est - global.est$est)/global.est$est * 100,
    high=(x$high - global.est$high)/global.est$high * 100
  )
  
  deviation=apply(deviation,2,median)
  
  return(t(as.data.frame(deviation)))
})

tmp$site1 <- factor(tmp$site, levels=c('Örebro', 'Nyköping', 'Hudiksvall', 'total'), labels=c('C1', 'C2', 'N', 'total'))
tmp$site2 <- factor(tmp$site, levels=c('total', 'Nyköping', 'Örebro', 'Hudiksvall'), labels=c('total', 'C2', 'C1', 'N'))

p1 <- ggplot(tmp, aes(x=sample.size, y=abs(est), colour=site1, shape=site1)) + 
  geom_point() + 
  geom_hline(aes(yintercept=10), colour='red', linetype=2) +
  facet_wrap(~method, ncol=2) + 
  scale_colour_grey(name='', end=.7) +
  scale_shape(name='') +
  scale_x_continuous(name = 'Sample size') +
  scale_y_continuous(name='Absolute deviation (%)', limits=c(0,100), breaks=seq(0,100,20)) +
  theme_light() +
  theme(strip.background=element_rect(fill='white'),
        strip.text=element_text(size=11, face='bold', colour='black'),
        legend.position='bottom',
        axis.title.x=element_blank(), axis.text.x=element_blank(), axis.ticks.x=element_blank())

copy <- tmp
copy$site2 <- 'total'
copy <- rbind(tmp, copy)

p2 <- ggplot(copy[copy$est<=10,], aes(y=site2, x=sample.size)) + 
  geom_boxplot() +
  geom_hline(aes(yintercept=1.5), colour='grey20') +
  facet_wrap(~method, ncol=2) + 
  scale_x_continuous(name='Sample size') +
  theme_light() +
  theme(strip.background=element_blank(),
        strip.text=element_blank(),
        axis.title.y=element_blank())

p1 + p2 + plot_layout(ncol=1, guides='collect') & theme(legend.position='bottom')
```

For each sample size in the cross-validation, calculate how the respective mean deviates from the mean derived from all individuals for a given cpature site and tracking method.

```
ggsave(filename='figure06_sample_size.png', width=170, height=110, unit='mm')
```

Quick table with summary of results:

Code

```
q <- ddply(tmp, 'method', function(x){
  data.frame(median=median(x$sample.size[x$est<=10]))
})

ind <- c('GPS collar'=sum(sapply(ind.gps[names(ind.gps) %in% nb.sites], length)),
         'Neckband'=sum(sapply(ind.nb[names(ind.nb) %in% nb.sites], length)))

q$prop.all <- round(q$median/ind * 100, digits=1)

knitr::kable(q, row.names=F, col.names=c('Tracking method', 'Number of individuals', '% of total sample size'),
             caption='Table shows the median number of individuals required to be within 10% deviation from the global area estimate for population-level distribution ranges.')
```

Table shows the median number of individuals required to be within 10% deviation from the global area estimate for population-level distribution ranges.

| Tracking method | Number of individuals | % of total sample size |
| --- | --- | --- |
| GPS collar | 16.5 | 26.6 |
| Neckband | 38.0 | 18.4 |

Code

```
ddply(tmp, c('site', 'method'), function(x){
  data.frame(median=median(x$sample.size[x$est<=10]))
})
```

```
        site     method median
1 Hudiksvall GPS collar    5.0
2 Hudiksvall   Neckband   56.0
3   Nyköping GPS collar    5.0
4   Nyköping   Neckband   27.5
5     Örebro GPS collar   22.5
6     Örebro   Neckband   10.5
```

# Tendency to migrate

Just a quick overview over the number of individuals of the two southernmost capture sites that could be considered migratory. For this quick check we consider as migratory an individual that was at least once observed at a distance >150 km from the respective capture site.

Code

```
check.mig <- ddply(all.tracks[all.tracks$population %in% c('Kristianstad', 'Svedala'),], c('population', 'individual.local.identifier'), function(x){
  p <- st_as_sf(x, coords=c('location.long', 'location.lat'), crs=st_crs(proj.ll)) # individual locations
  start <- st_as_sf(inf[inf$steel.ringnumber==unique(x$individual.local.identifier),], coords=c('long', 'lat'), crs=st_crs(proj.ll)) # capture site
  circle <- st_buffer(start, dist=150000, nQuadSegs=25) # 100 km buffer

  check <- st_within(p, circle, sparse=F)[,1]
  return(any(!check))
})

check.mig$result <- ifelse(check.mig$V1, 'migratory', 'not migratory')

table(check.mig$population, check.mig$result)
```

```
               migratory not migratory
  Kristianstad        10            14
  Svedala              5            26
```

# Session info

Code

```
sessionInfo()
```

```
R version 4.3.2 (2023-10-31 ucrt)
Platform: x86_64-w64-mingw32/x64 (64-bit)
Running under: Windows 11 x64 (build 26100)

Matrix products: default


locale:
[1] LC_COLLATE=en_US.UTF-8  LC_CTYPE=en_US.UTF-8    LC_MONETARY=en_US.UTF-8
[4] LC_NUMERIC=C            LC_TIME=en_US.UTF-8    

time zone: Europe/Stockholm
tzcode source: internal

attached base packages:
[1] stats     graphics  grDevices utils     datasets  methods   base     

other attached packages:
 [1] ctmm_1.2.1        tidyterra_0.4.0   patchwork_1.2.0   viridisLite_0.4.2
 [5] ggplot2_3.5.1     terra_1.7-29      suntools_1.0.0    sp_1.6-0         
 [9] geosphere_1.5-18  sf_1.0-19         lubridate_1.9.2   plyr_1.8.8       

loaded via a namespace (and not attached):
 [1] gtable_0.3.3        xfun_0.50           raster_3.6-20      
 [4] htmlwidgets_1.6.2   lattice_0.21-9      numDeriv_2016.8-1.1
 [7] vctrs_0.6.2         tools_4.3.2         generics_0.1.3     
[10] tibble_3.2.1        proxy_0.4-27        fansi_1.0.4        
[13] DEoptimR_1.0-13     pkgconfig_2.0.3     Matrix_1.6-1.1     
[16] KernSmooth_2.23-22  lifecycle_1.0.3     compiler_4.3.2     
[19] farver_2.1.1        textshaping_0.3.6   statmod_1.5.0      
[22] munsell_0.5.0       codetools_0.2-19    Bessel_0.6-0       
[25] htmltools_0.5.8.1   class_7.3-22        gmp_0.7-1          
[28] yaml_2.3.7          pillar_1.9.0        tidyr_1.3.0        
[31] classInt_0.4-9      wk_0.7.3            RSpectra_0.16-1    
[34] robustbase_0.95-1   tidyselect_1.2.0    digest_0.6.31      
[37] dplyr_1.1.2         purrr_1.0.1         labeling_0.4.2     
[40] fastmap_1.2.0       grid_4.3.2          colorspace_2.1-0   
[43] expm_0.999-7        cli_3.6.1           magrittr_2.0.3     
[46] utf8_1.2.3          e1071_1.7-13        Rmpfr_0.9-2        
[49] withr_2.5.0         scales_1.3.0        timechange_0.2.0   
[52] rmarkdown_2.21      Gmedian_1.2.7       ragg_1.2.5         
[55] evaluate_0.21       knitr_1.43          rgdal_1.6-6        
[58] s2_1.1.4            rlang_1.1.1         Rcpp_1.0.10        
[61] glue_1.6.2          DBI_1.1.3           rstudioapi_0.15.0  
[64] jsonlite_1.8.4      R6_2.5.1            systemfonts_1.2.3  
[67] units_0.8-2
```
