# Supplementary figures and images for: Do GPS collars and coded neckbands tell the same story about year-round movements in geese?
Source: Mov Ecol. 2026 Jan 12;14:3. doi: 10.1186/s40462-025-00620-y (PMC12829013; doi:10.1186/s40462-025-00620-y)

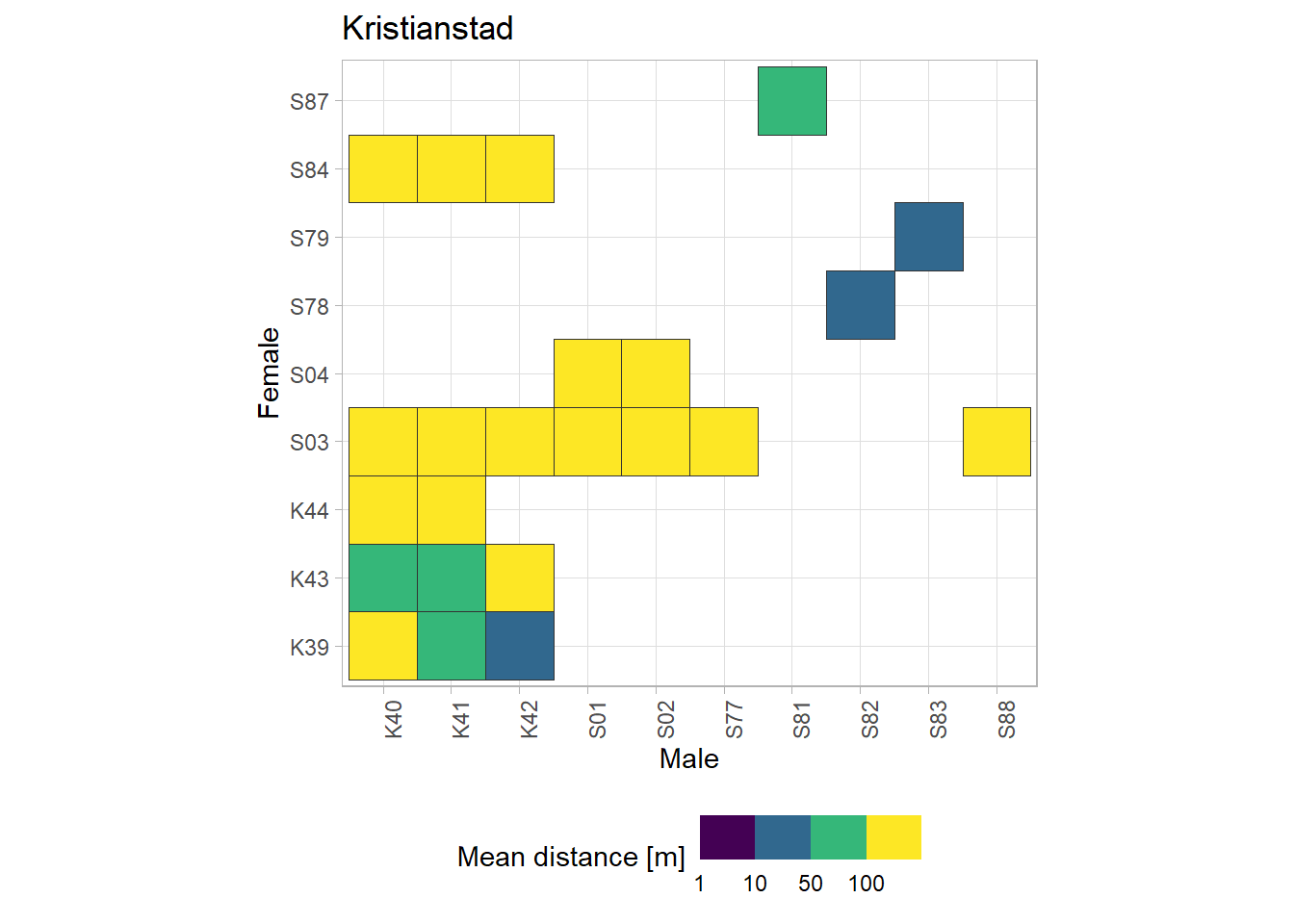

Supplement: Supplementary file 2 — Supplementary Material 2 [file 40462_2025_620_MOESM2_ESM.zip › Appendix_B_code/tracking_method_comparison_ANNOTATED_CODE_files/figure-html/censor-families-plot-pairwisedist-1.png]

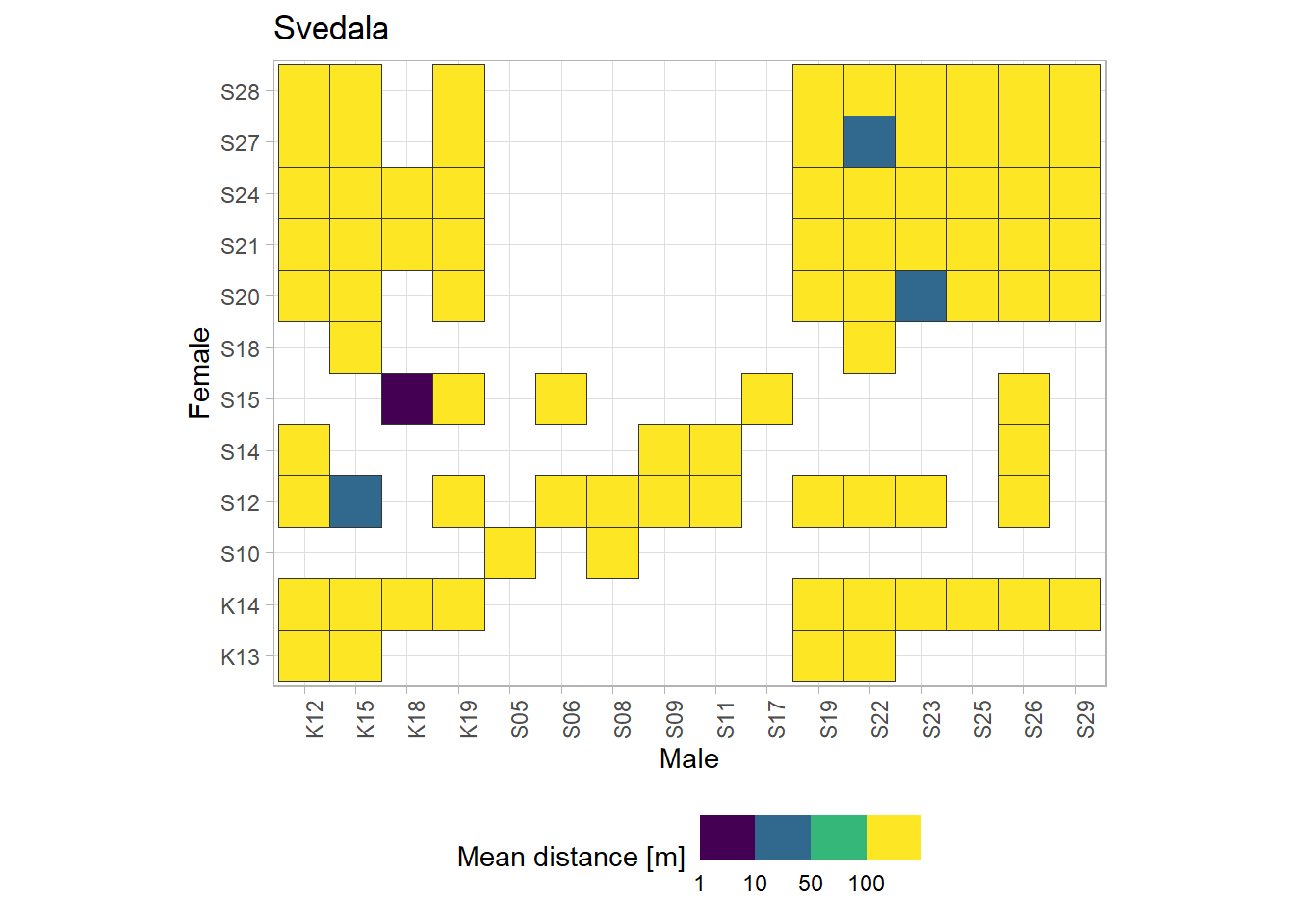

Supplement: Supplementary file 2 — Supplementary Material 2 [file 40462_2025_620_MOESM2_ESM.zip › Appendix_B_code/tracking_method_comparison_ANNOTATED_CODE_files/figure-html/censor-families-plot-pairwisedist-2.png]

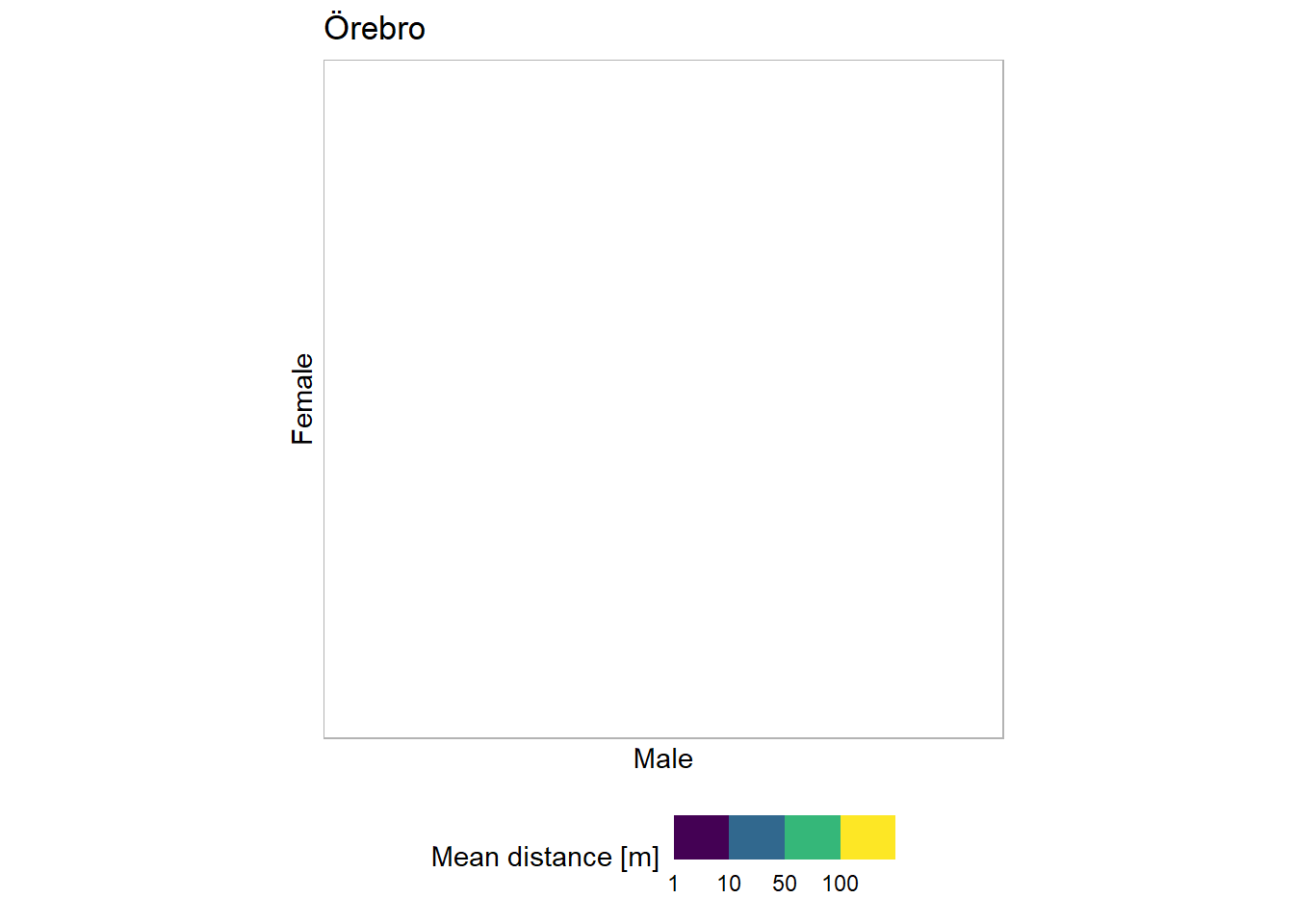

Supplement: Supplementary file 2 — Supplementary Material 2 [file 40462_2025_620_MOESM2_ESM.zip › Appendix_B_code/tracking_method_comparison_ANNOTATED_CODE_files/figure-html/censor-families-plot-pairwisedist-3.png]

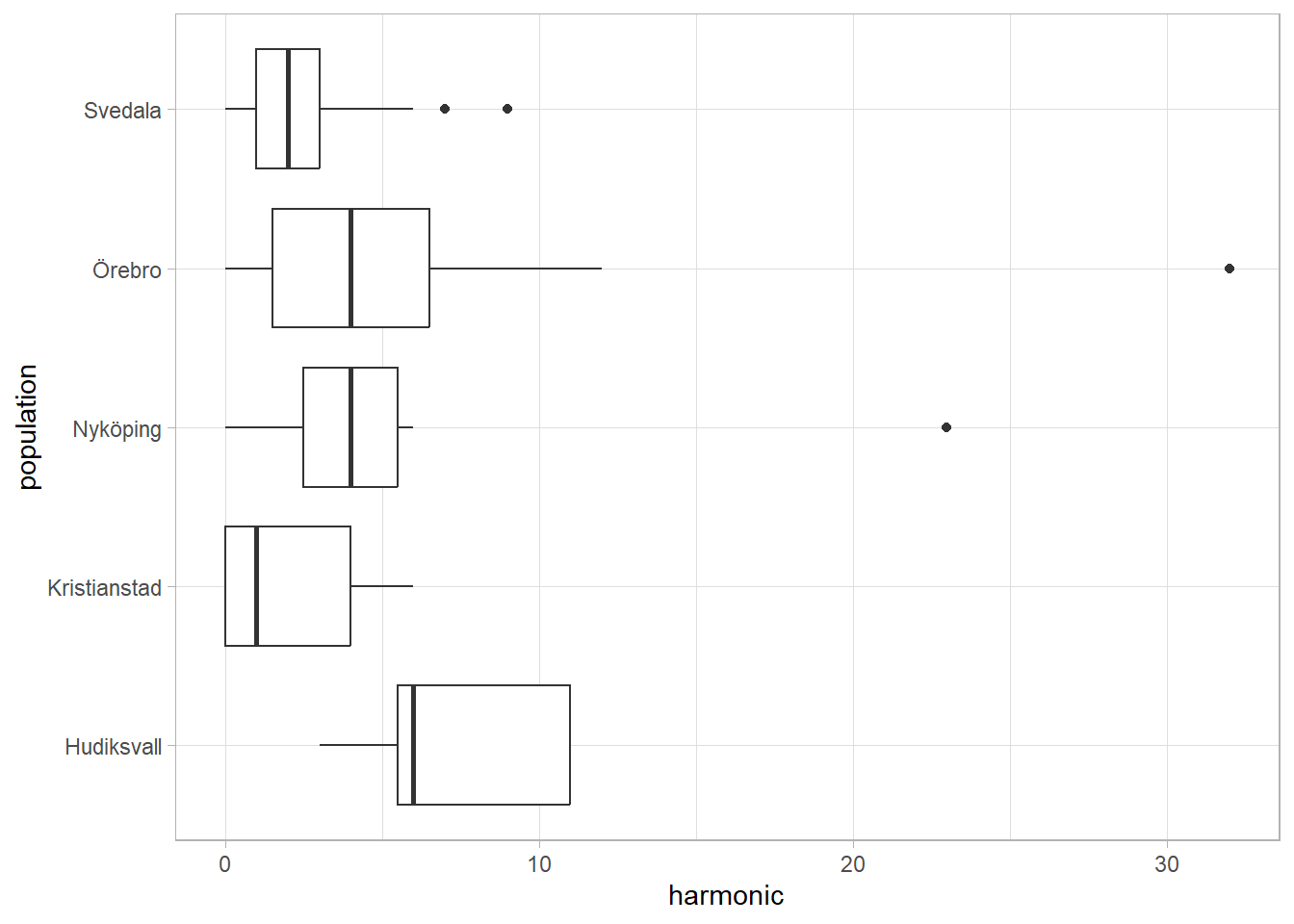

Supplement: Supplementary file 2 — Supplementary Material 2 [file 40462_2025_620_MOESM2_ESM.zip › Appendix_B_code/tracking_method_comparison_ANNOTATED_CODE_files/figure-html/gps-annual-gps-harmonics-1.png]

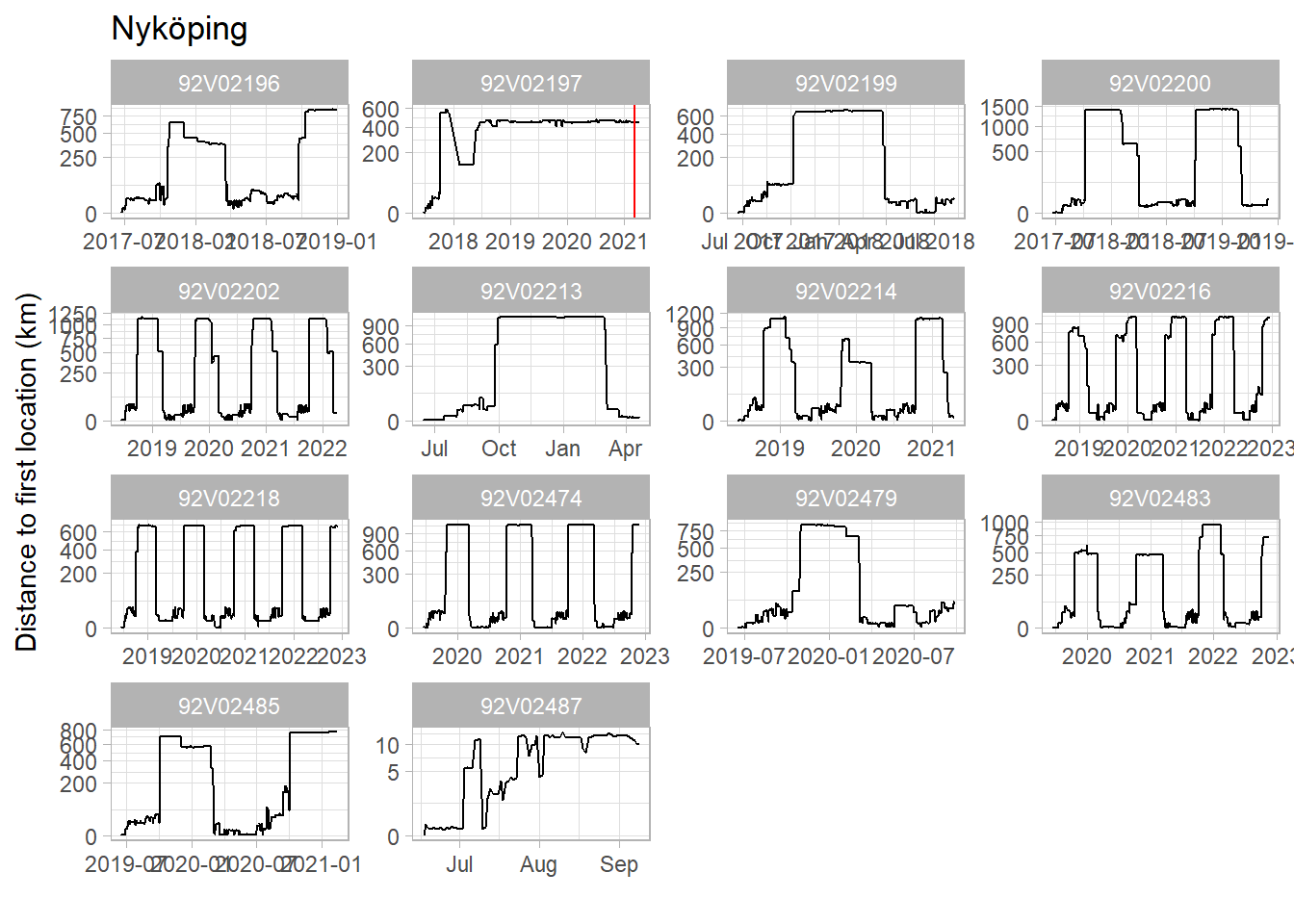

Supplement: Supplementary file 2 — Supplementary Material 2 [file 40462_2025_620_MOESM2_ESM.zip › Appendix_B_code/tracking_method_comparison_ANNOTATED_CODE_files/figure-html/gps-censor-other-1.png]

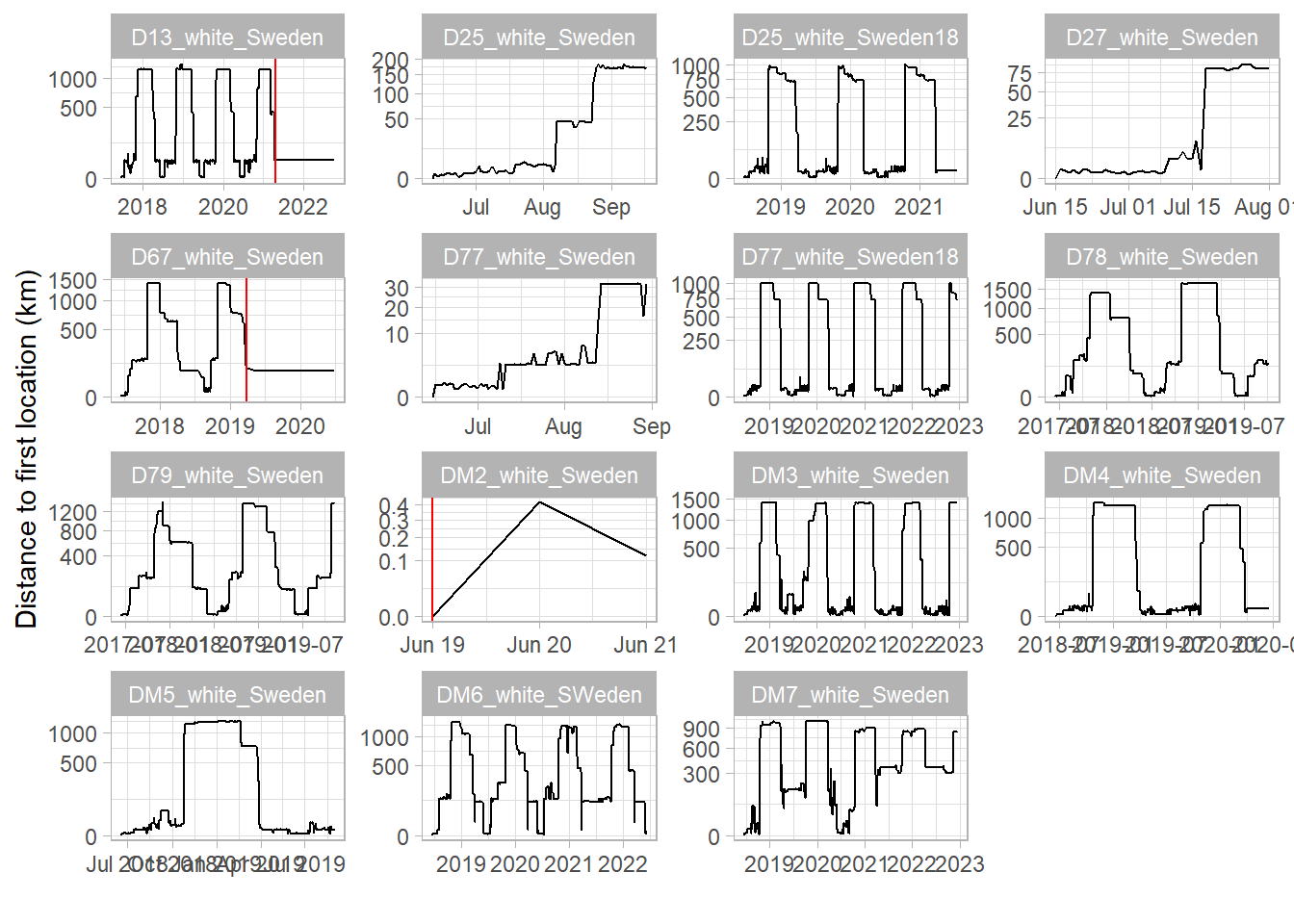

Supplement: Supplementary file 2 — Supplementary Material 2 [file 40462_2025_620_MOESM2_ESM.zip › Appendix_B_code/tracking_method_comparison_ANNOTATED_CODE_files/figure-html/gps-timelines-hudiksvall-1.png]

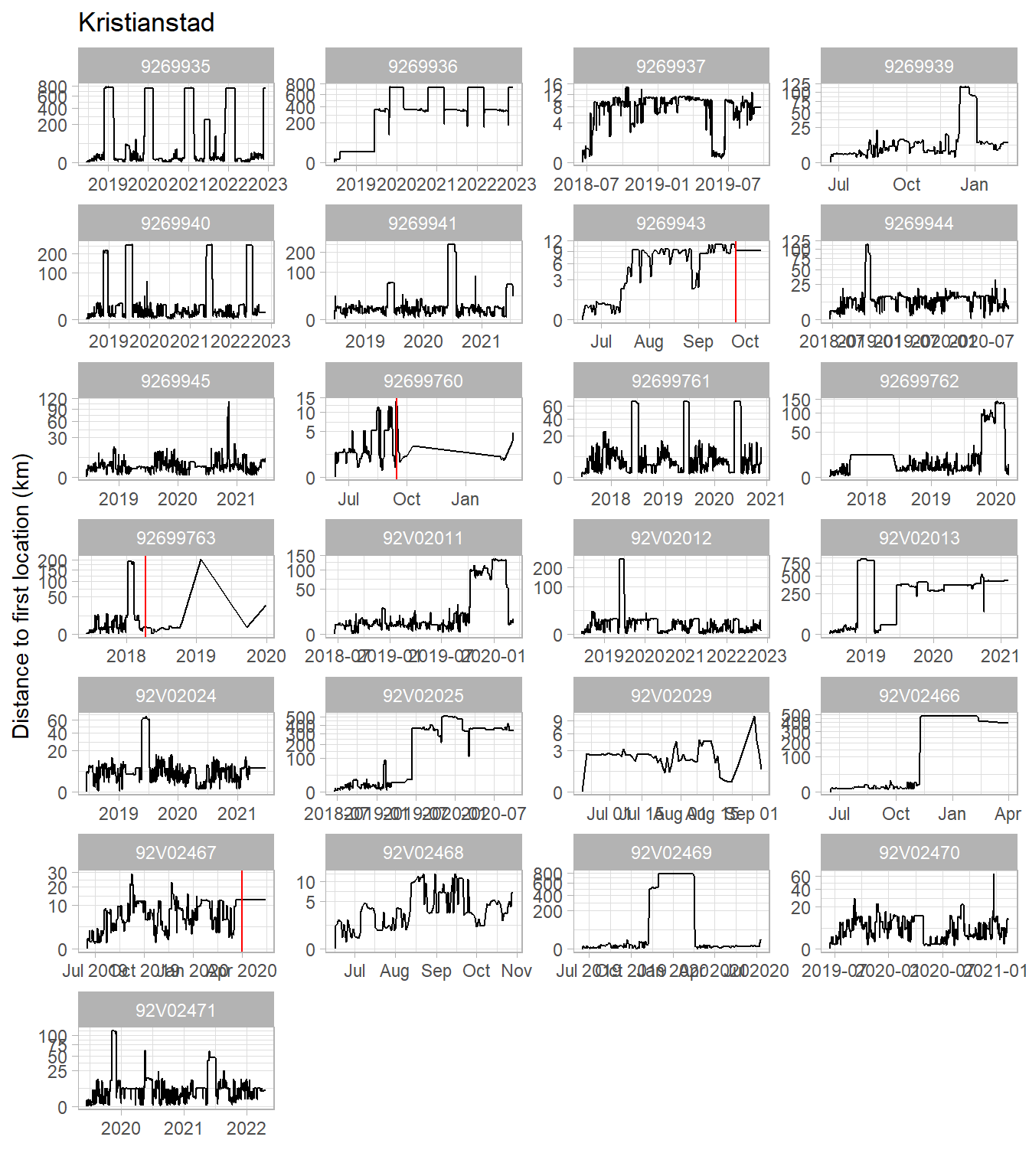

Supplement: Supplementary file 2 — Supplementary Material 2 [file 40462_2025_620_MOESM2_ESM.zip › Appendix_B_code/tracking_method_comparison_ANNOTATED_CODE_files/figure-html/gps-timelines-kristianstad-1.png]

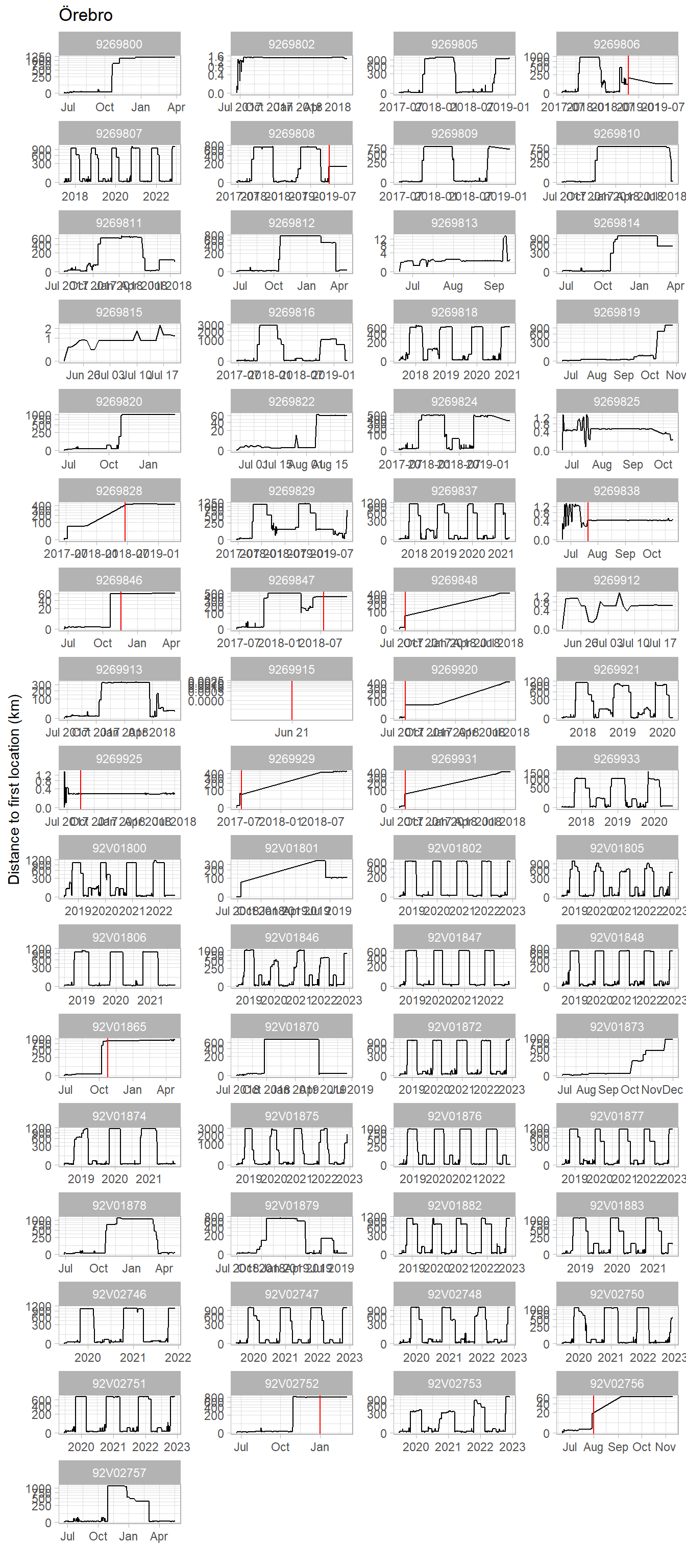

Supplement: Supplementary file 2 — Supplementary Material 2 [file 40462_2025_620_MOESM2_ESM.zip › Appendix_B_code/tracking_method_comparison_ANNOTATED_CODE_files/figure-html/gps-timelines-orebro-1.png]

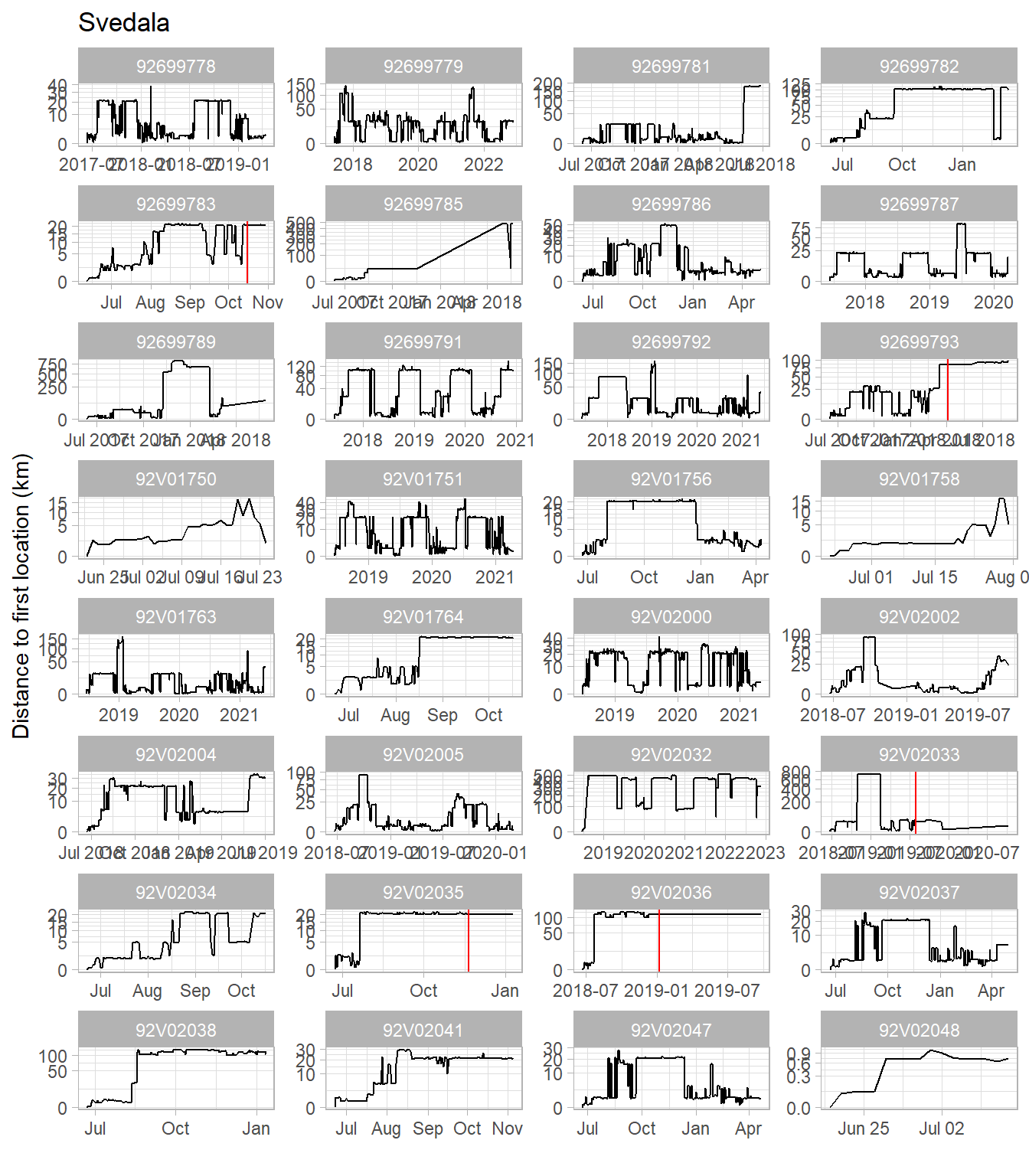

Supplement: Supplementary file 2 — Supplementary Material 2 [file 40462_2025_620_MOESM2_ESM.zip › Appendix_B_code/tracking_method_comparison_ANNOTATED_CODE_files/figure-html/gps-timelines-svedala-1.png]

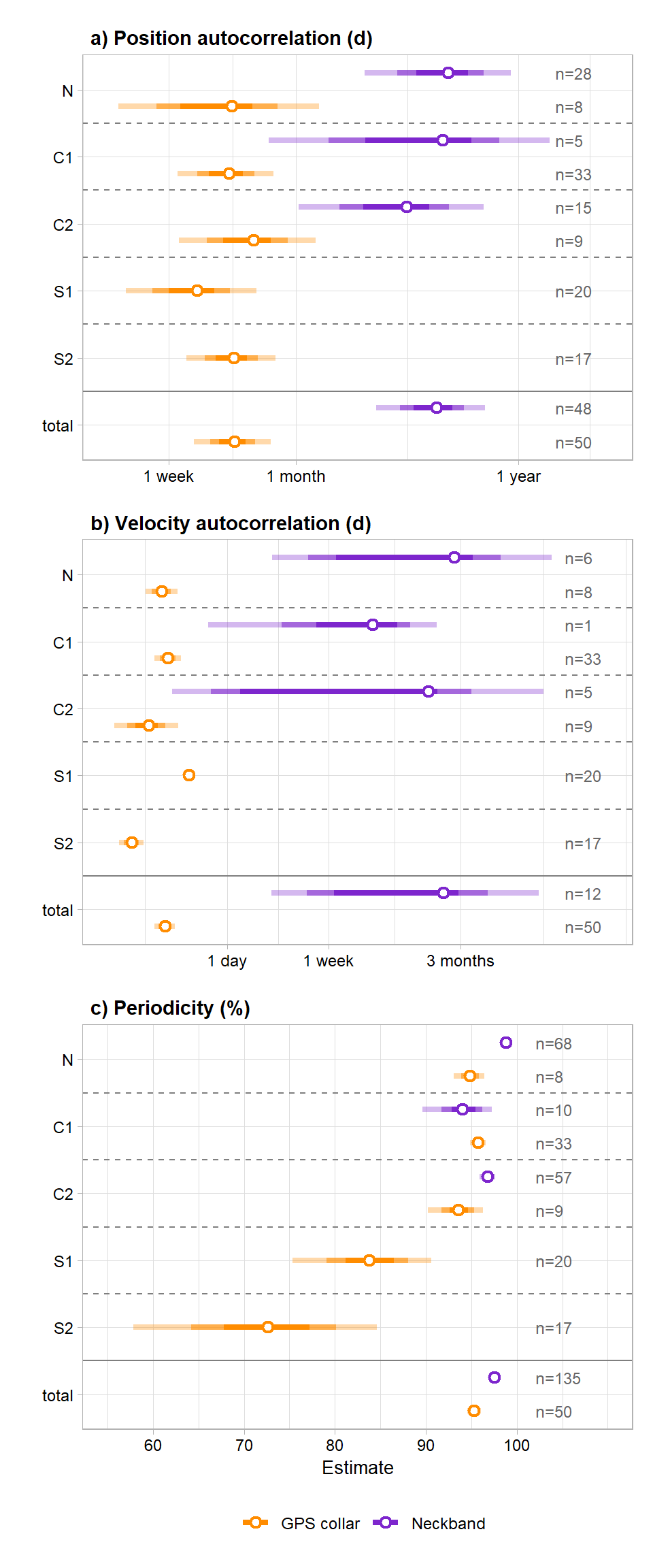

Supplement: Supplementary file 2 — Supplementary Material 2 [file 40462_2025_620_MOESM2_ESM.zip › Appendix_B_code/tracking_method_comparison_ANNOTATED_CODE_files/figure-html/migratory-models-plots2-1.png]

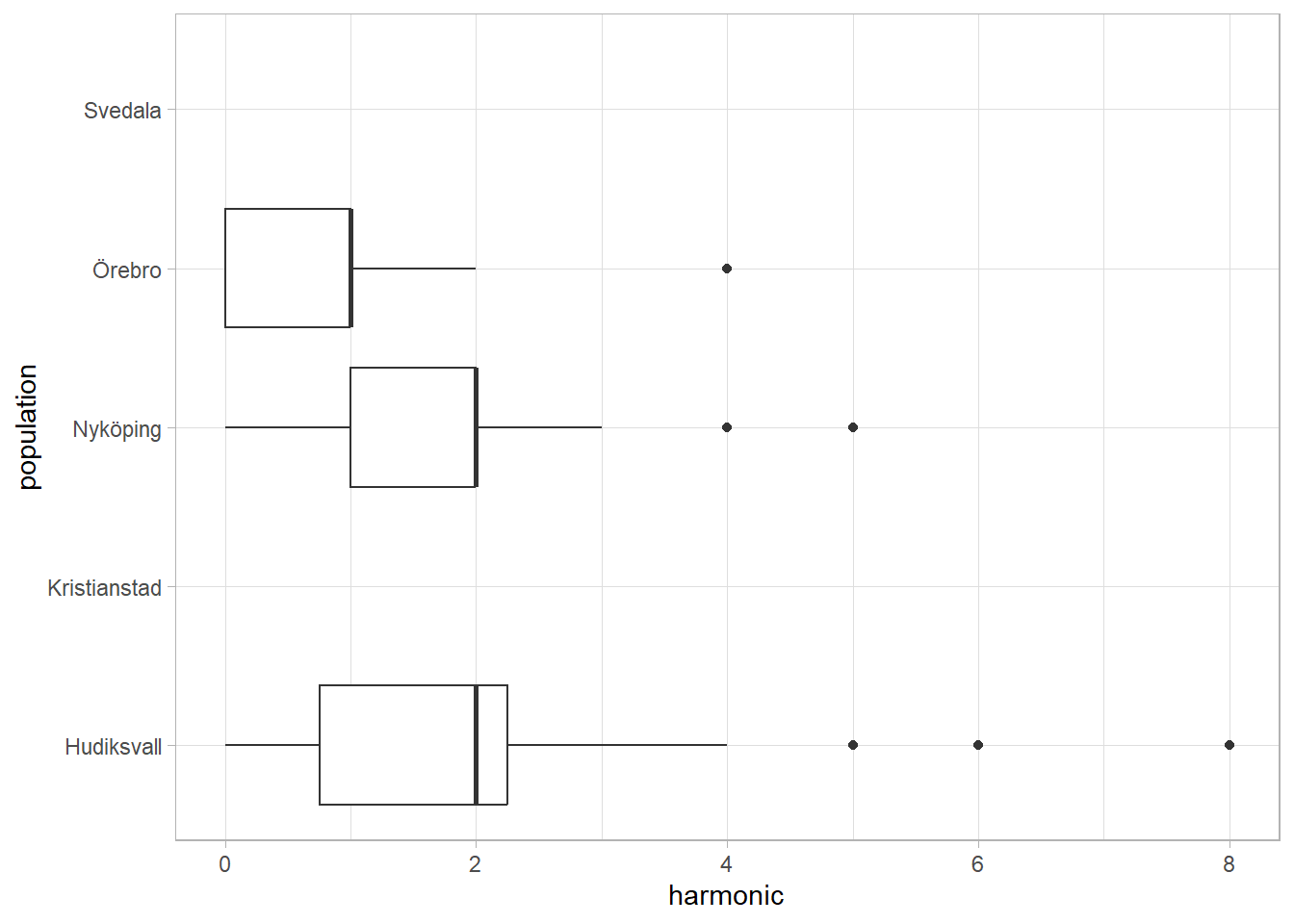

Supplement: Supplementary file 2 — Supplementary Material 2 [file 40462_2025_620_MOESM2_ESM.zip › Appendix_B_code/tracking_method_comparison_ANNOTATED_CODE_files/figure-html/neckband-annual-ctmm-harmonics-1.png]

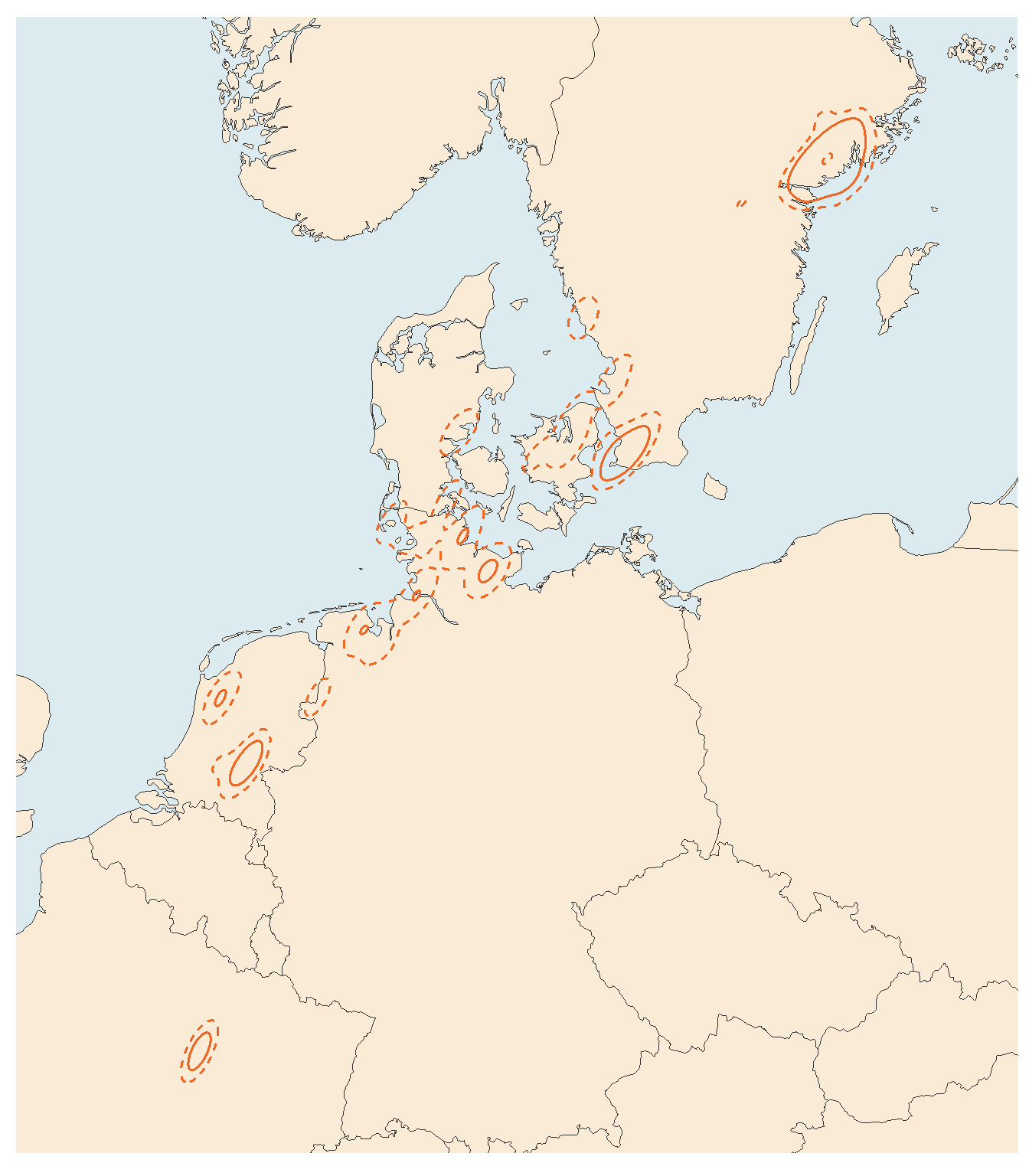

Supplement: Supplementary file 2 — Supplementary Material 2 [file 40462_2025_620_MOESM2_ESM.zip › Appendix_B_code/tracking_method_comparison_ANNOTATED_CODE_files/figure-html/pkde-plot-contour-1.png]

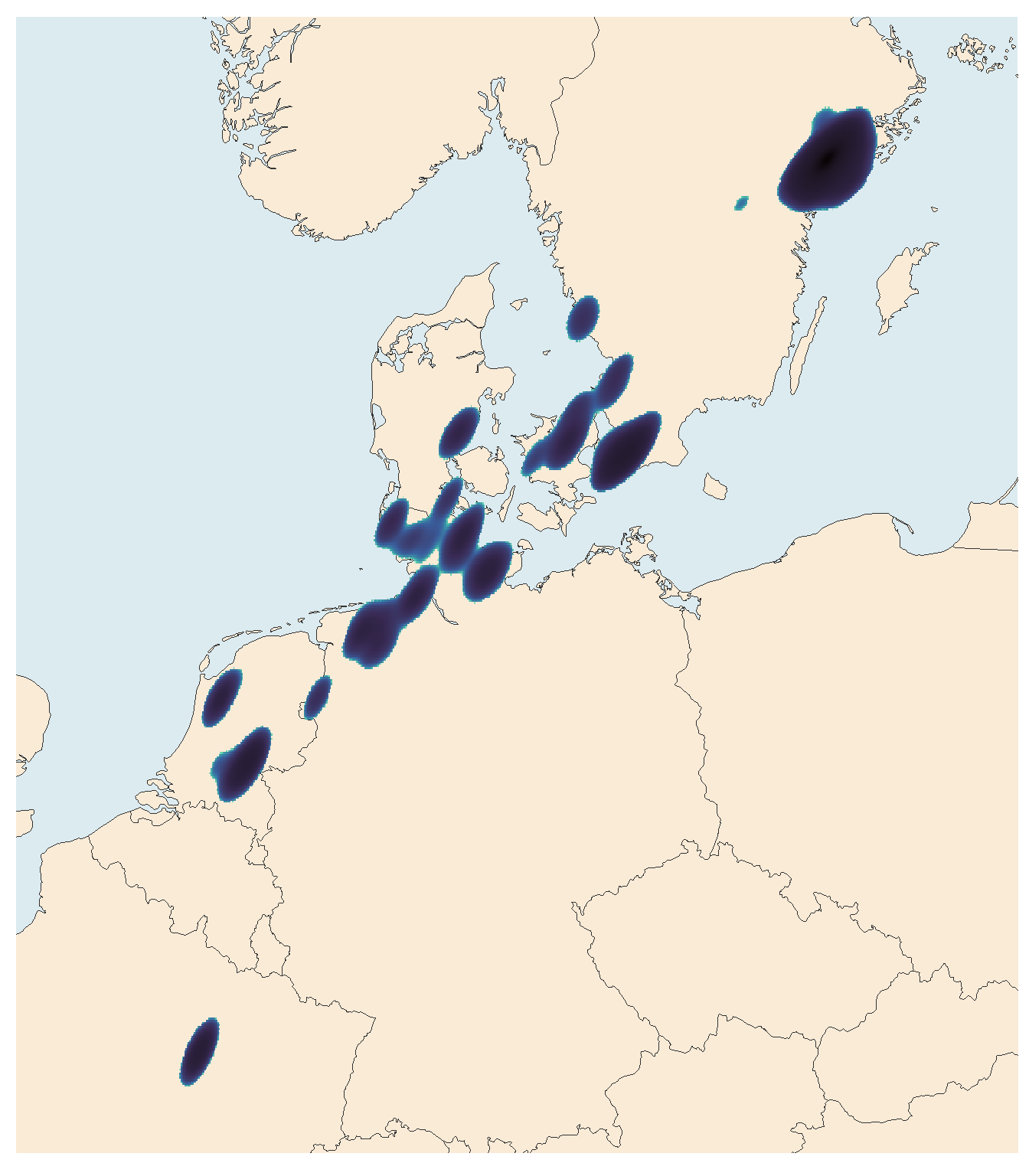

Supplement: Supplementary file 2 — Supplementary Material 2 [file 40462_2025_620_MOESM2_ESM.zip › Appendix_B_code/tracking_method_comparison_ANNOTATED_CODE_files/figure-html/pkde-plot-pmf-1.png]

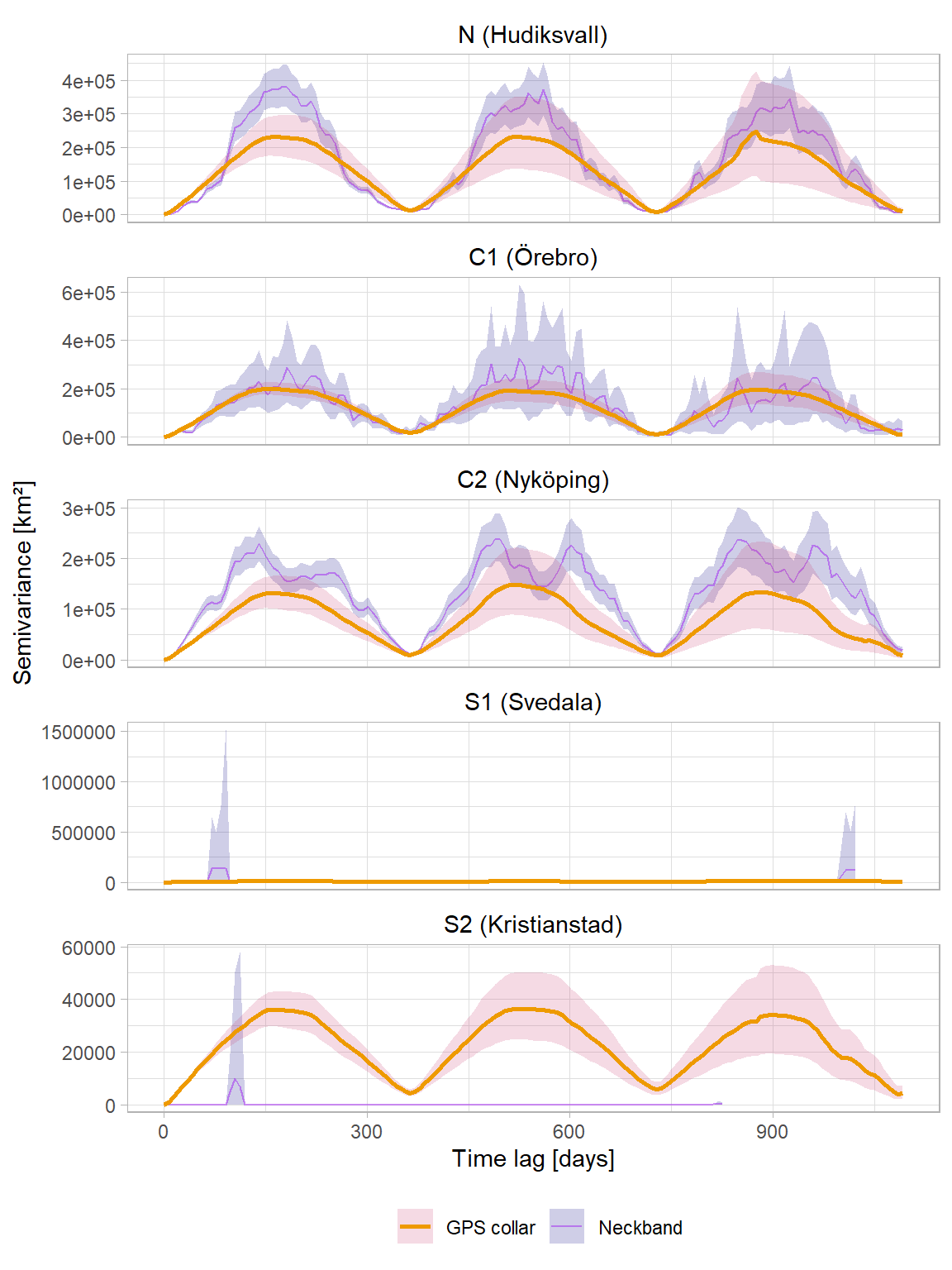

Supplement: Supplementary file 2 — Supplementary Material 2 [file 40462_2025_620_MOESM2_ESM.zip › Appendix_B_code/tracking_method_comparison_ANNOTATED_CODE_files/figure-html/semivariograms-plot-1.png]

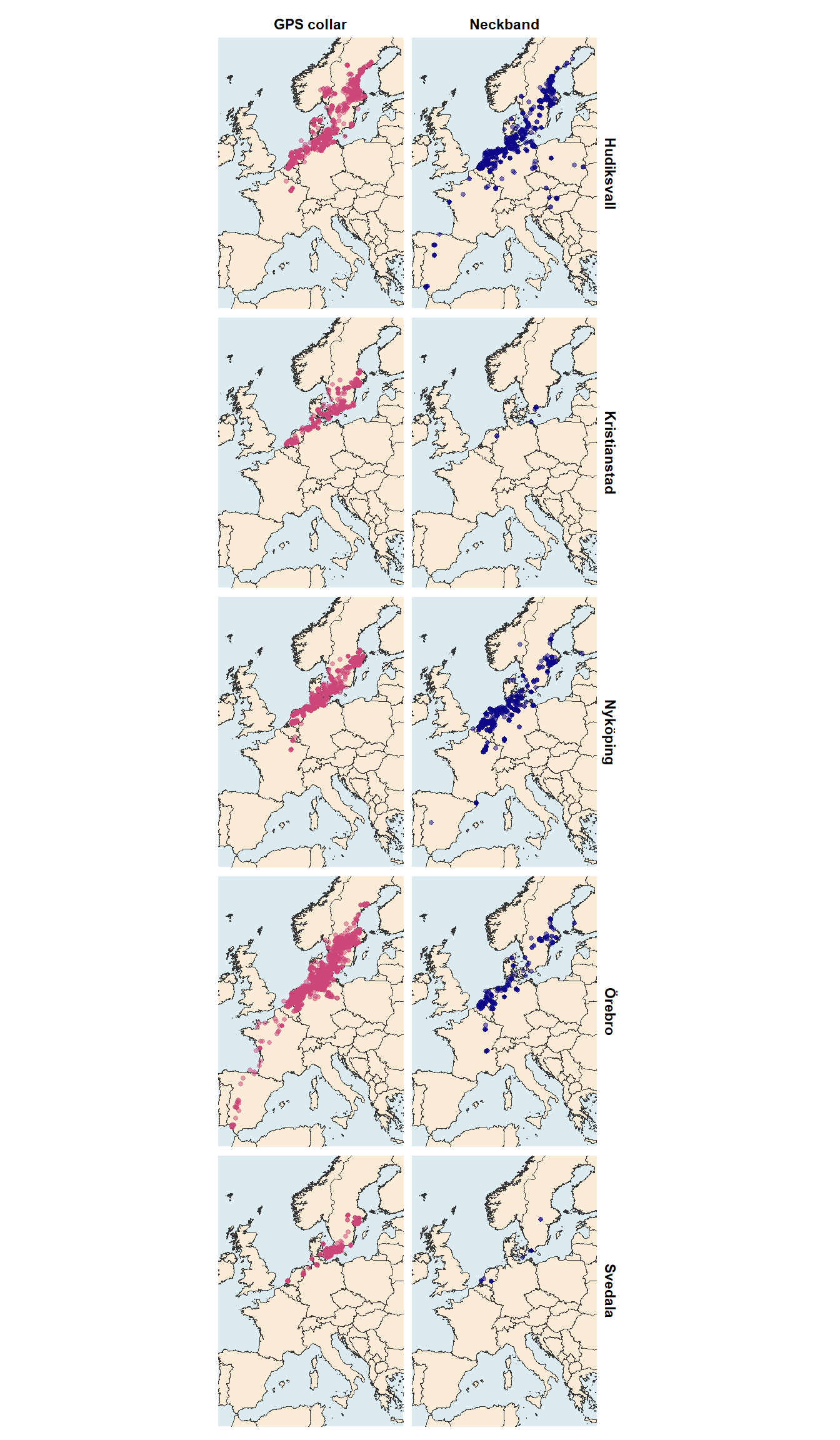

Supplement: Supplementary file 2 — Supplementary Material 2 [file 40462_2025_620_MOESM2_ESM.zip › Appendix_B_code/tracking_method_comparison_ANNOTATED_CODE_files/figure-html/tracking-maps-1.png]

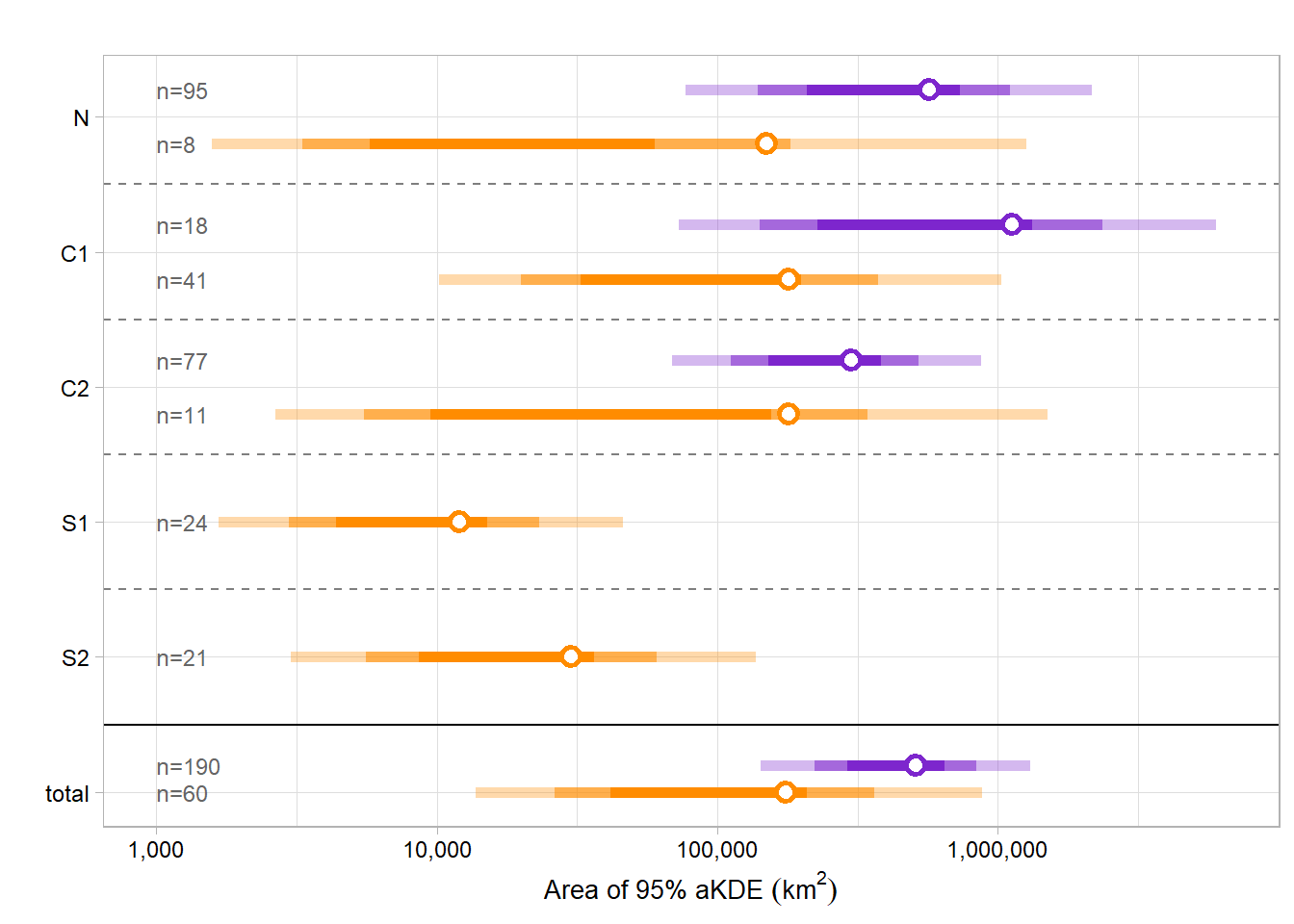

Supplement: Supplementary file 2 — Supplementary Material 2 [file 40462_2025_620_MOESM2_ESM.zip › Appendix_B_code/tracking_method_comparison_ANNOTATED_CODE_files/figure-html/ud-meta-plot-1.png]

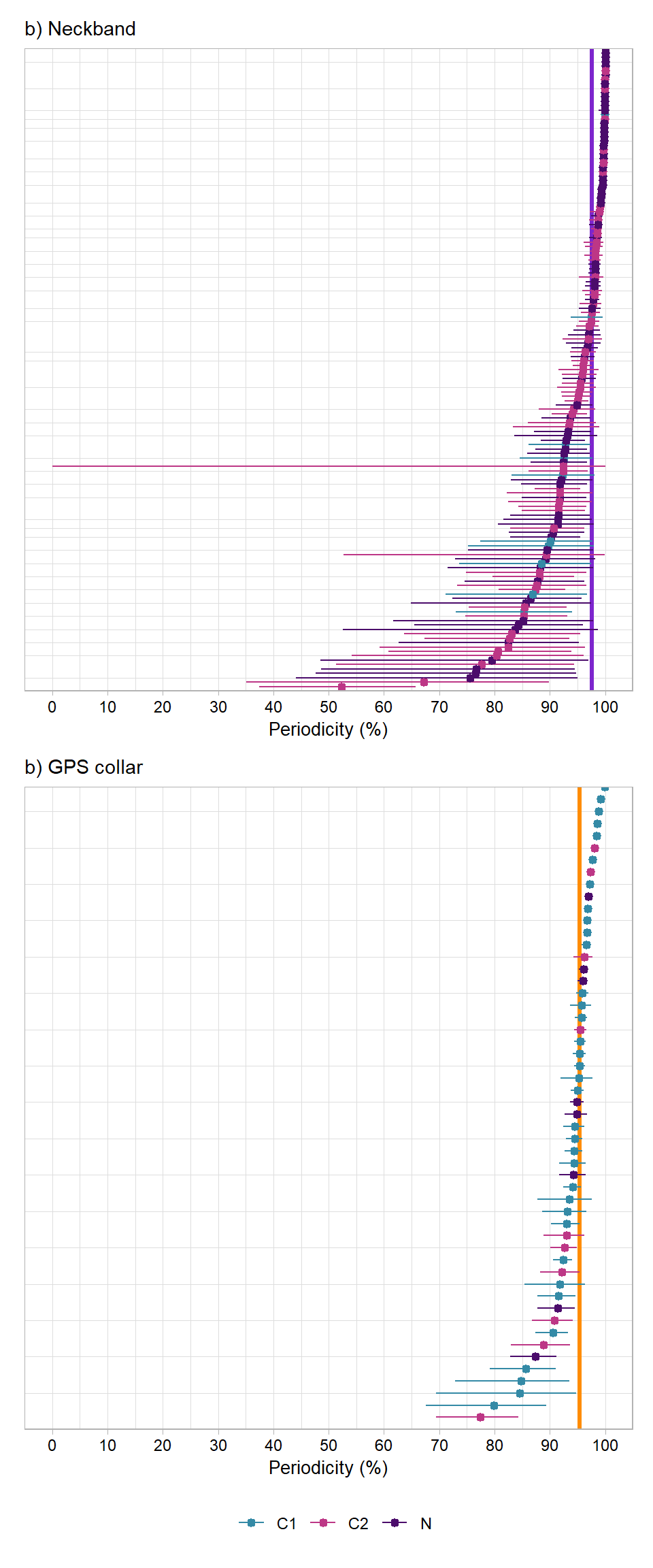

Supplement: Supplementary file 2 — Supplementary Material 2 [file 40462_2025_620_MOESM2_ESM.zip › Appendix_B_code/tracking_method_comparison_ANNOTATED_CODE_files/figure-html/unnamed-chunk-19-1.png]

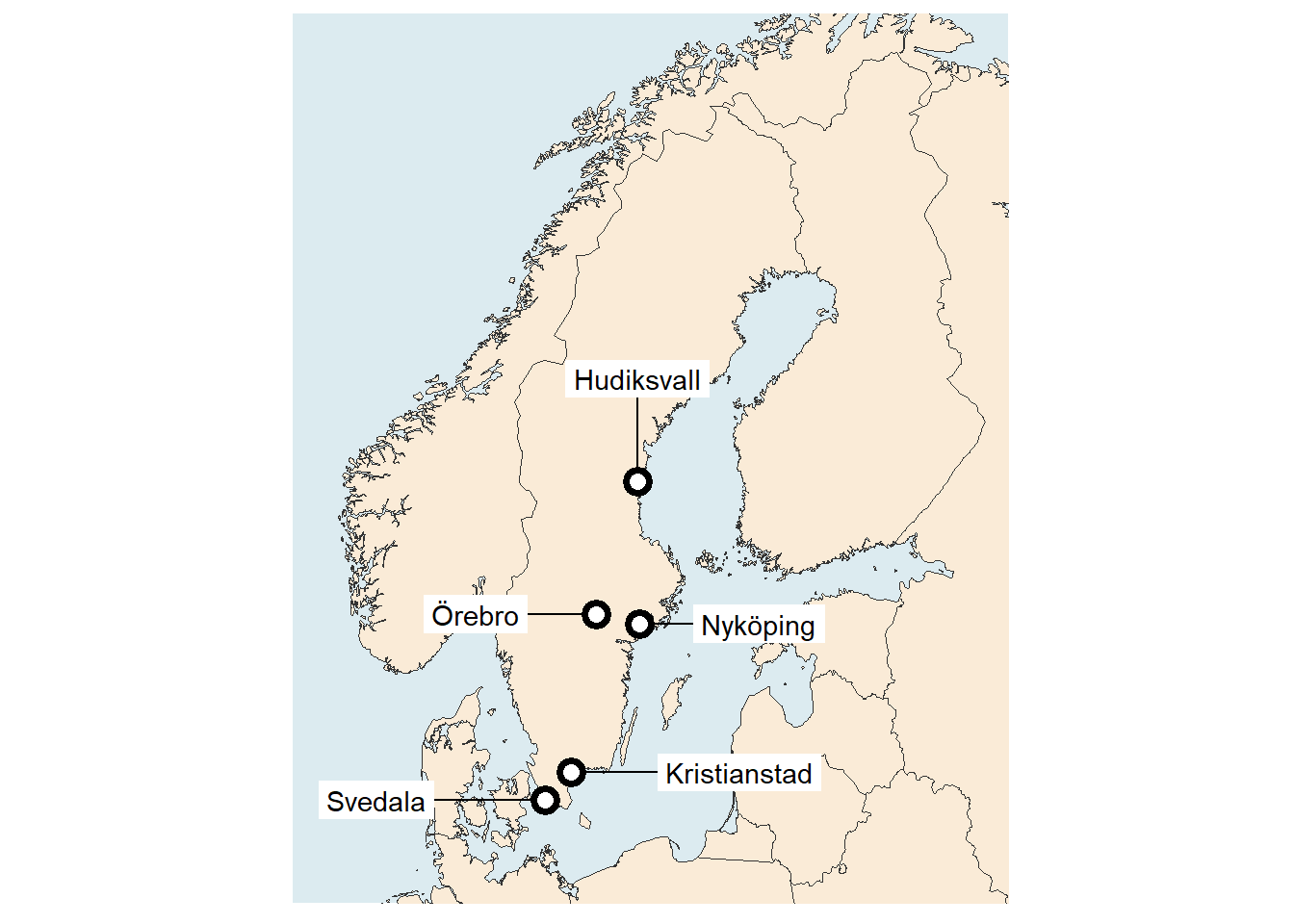

Supplement: Supplementary file 2 — Supplementary Material 2 [file 40462_2025_620_MOESM2_ESM.zip › Appendix_B_code/tracking_method_comparison_ANNOTATED_CODE_files/figure-html/unnamed-chunk-2-1.png]

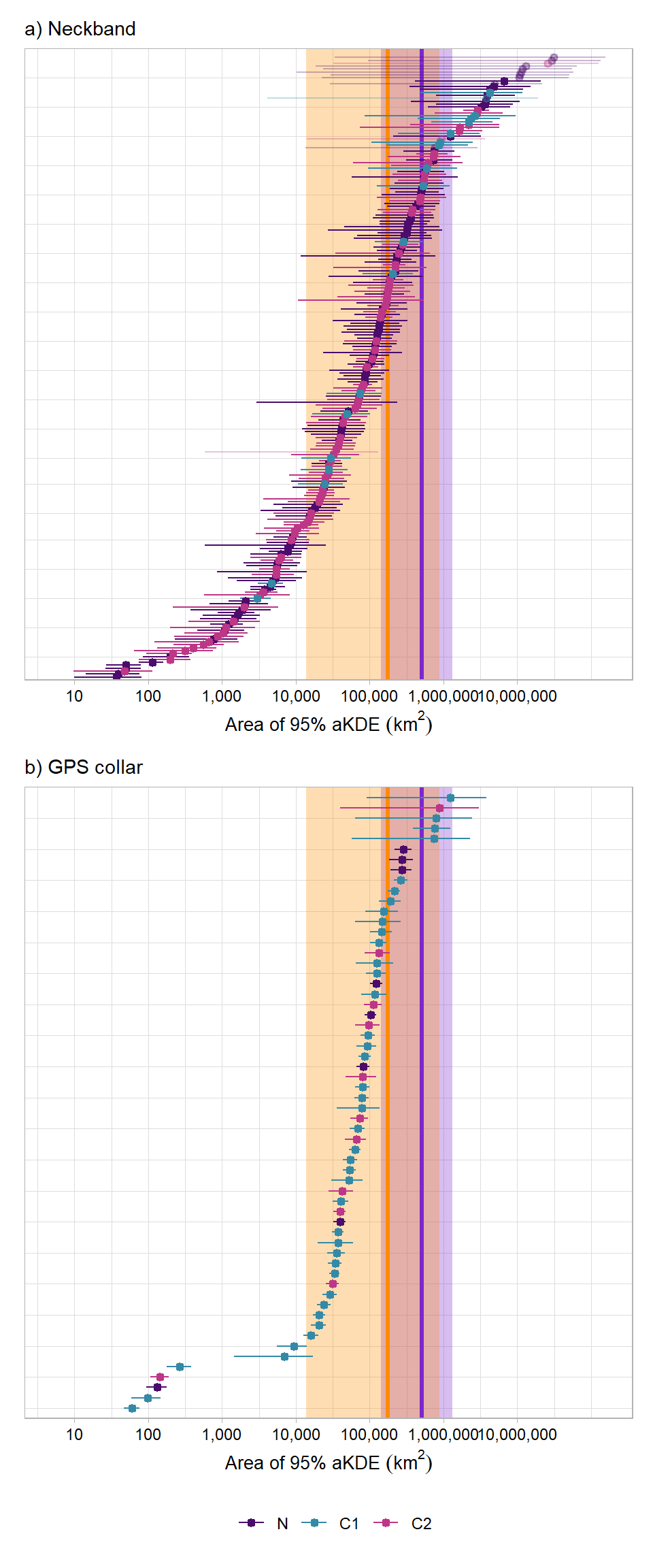

Supplement: Supplementary file 2 — Supplementary Material 2 [file 40462_2025_620_MOESM2_ESM.zip › Appendix_B_code/tracking_method_comparison_ANNOTATED_CODE_files/figure-html/unnamed-chunk-22-1.png]

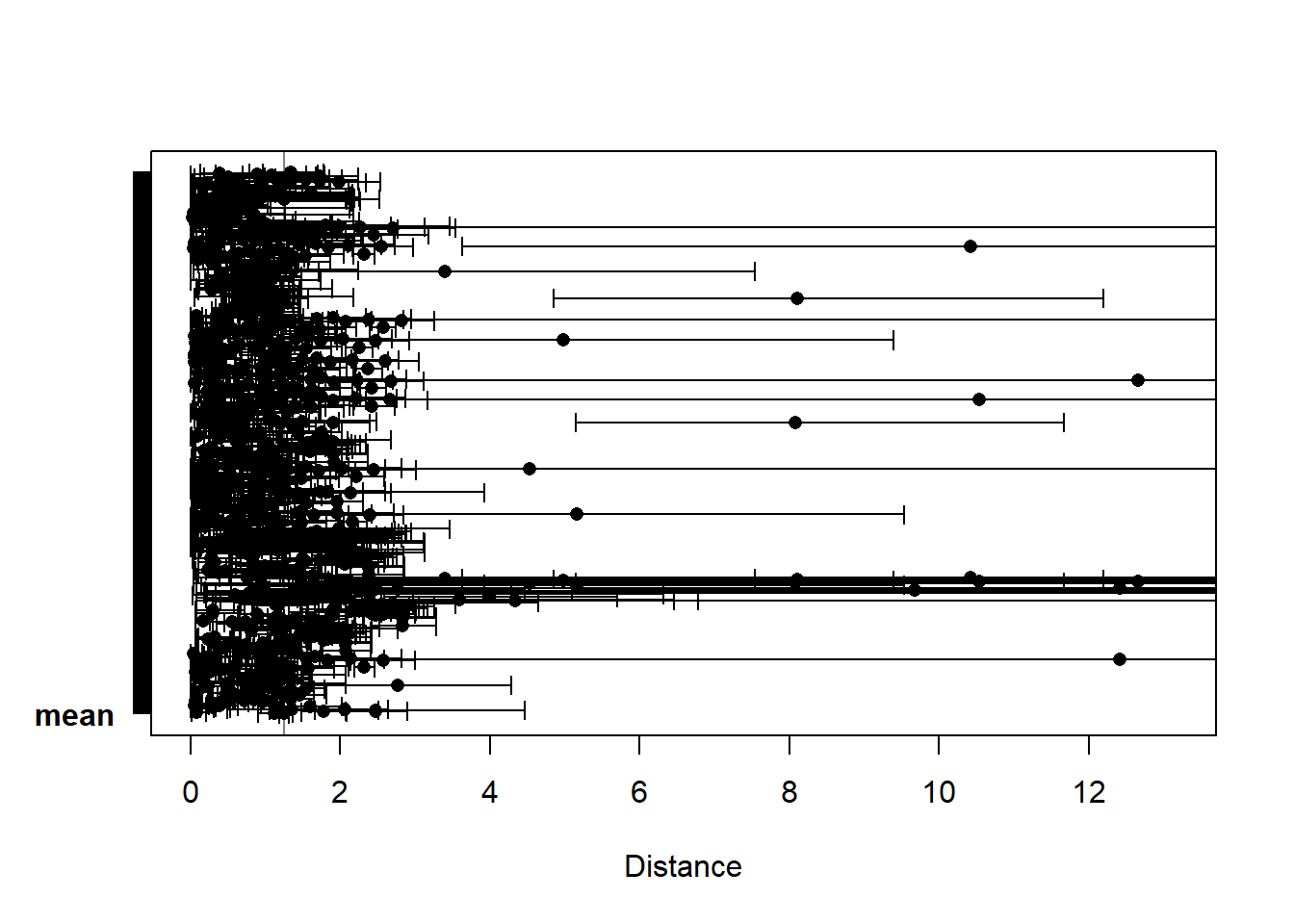

Supplement: Supplementary file 2 — Supplementary Material 2 [file 40462_2025_620_MOESM2_ESM.zip › Appendix_B_code/tracking_method_comparison_ANNOTATED_CODE_files/figure-html/unnamed-chunk-29-1.png]

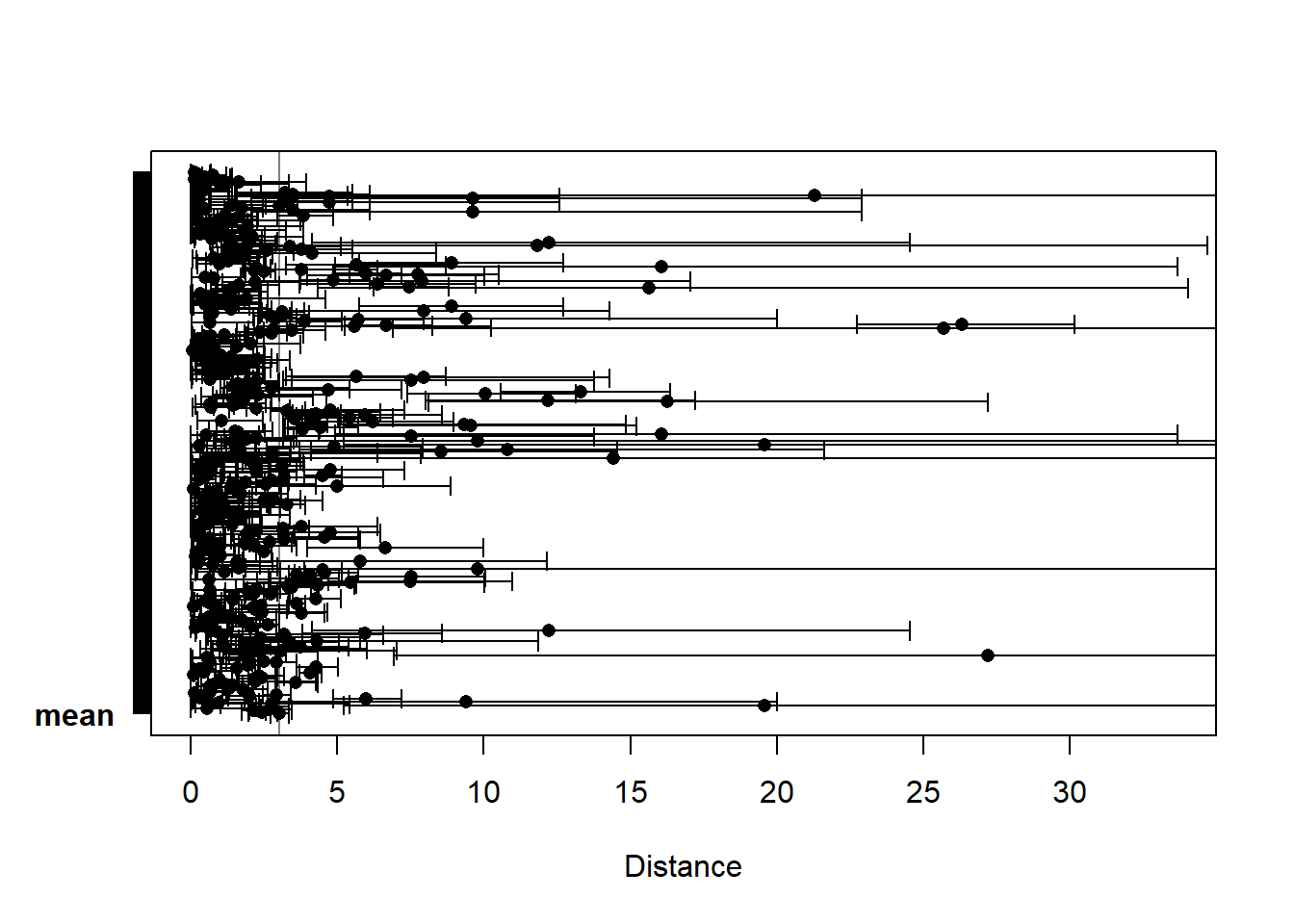

Supplement: Supplementary file 2 — Supplementary Material 2 [file 40462_2025_620_MOESM2_ESM.zip › Appendix_B_code/tracking_method_comparison_ANNOTATED_CODE_files/figure-html/unnamed-chunk-29-2.png]

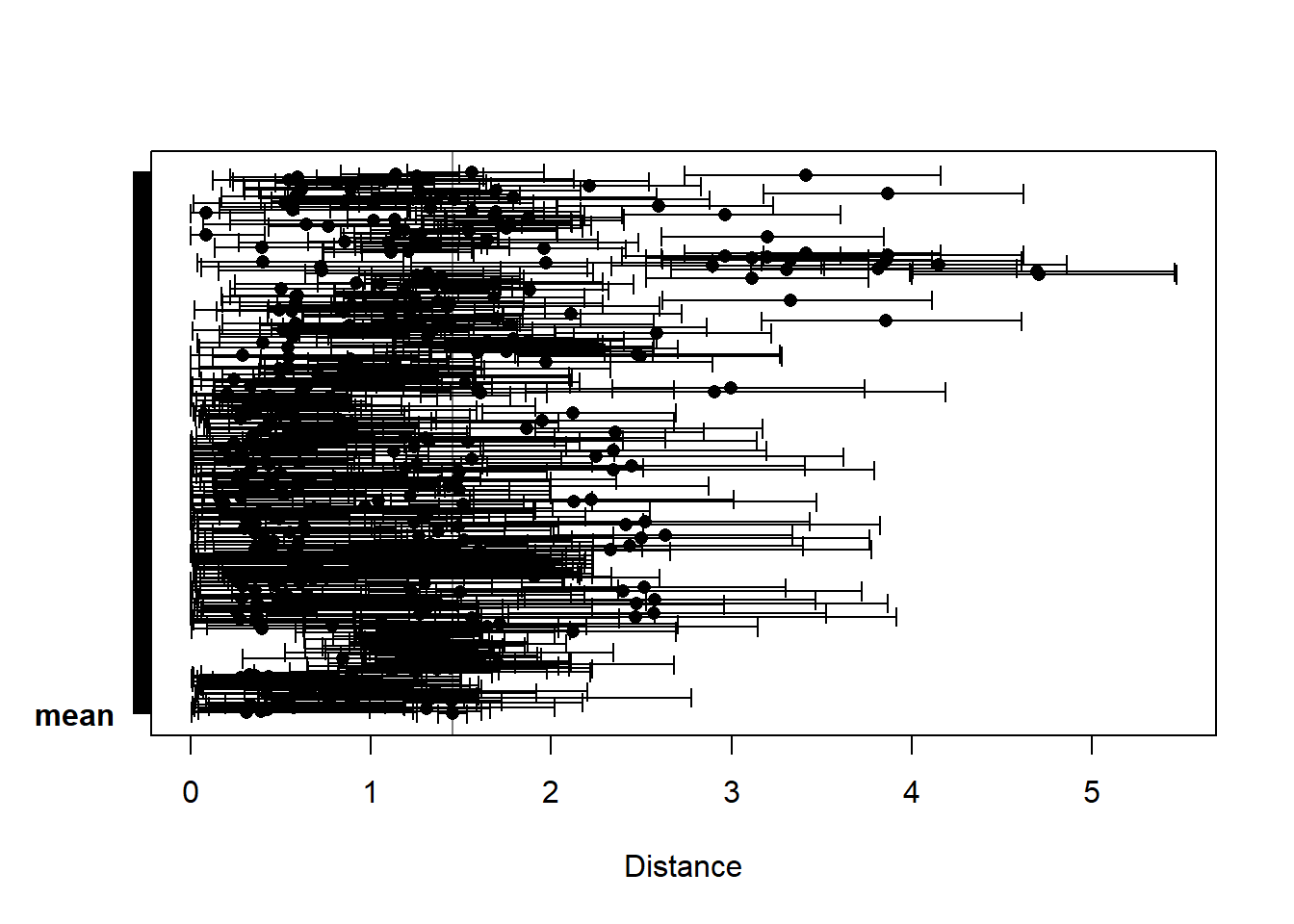

Supplement: Supplementary file 2 — Supplementary Material 2 [file 40462_2025_620_MOESM2_ESM.zip › Appendix_B_code/tracking_method_comparison_ANNOTATED_CODE_files/figure-html/unnamed-chunk-29-3.png]

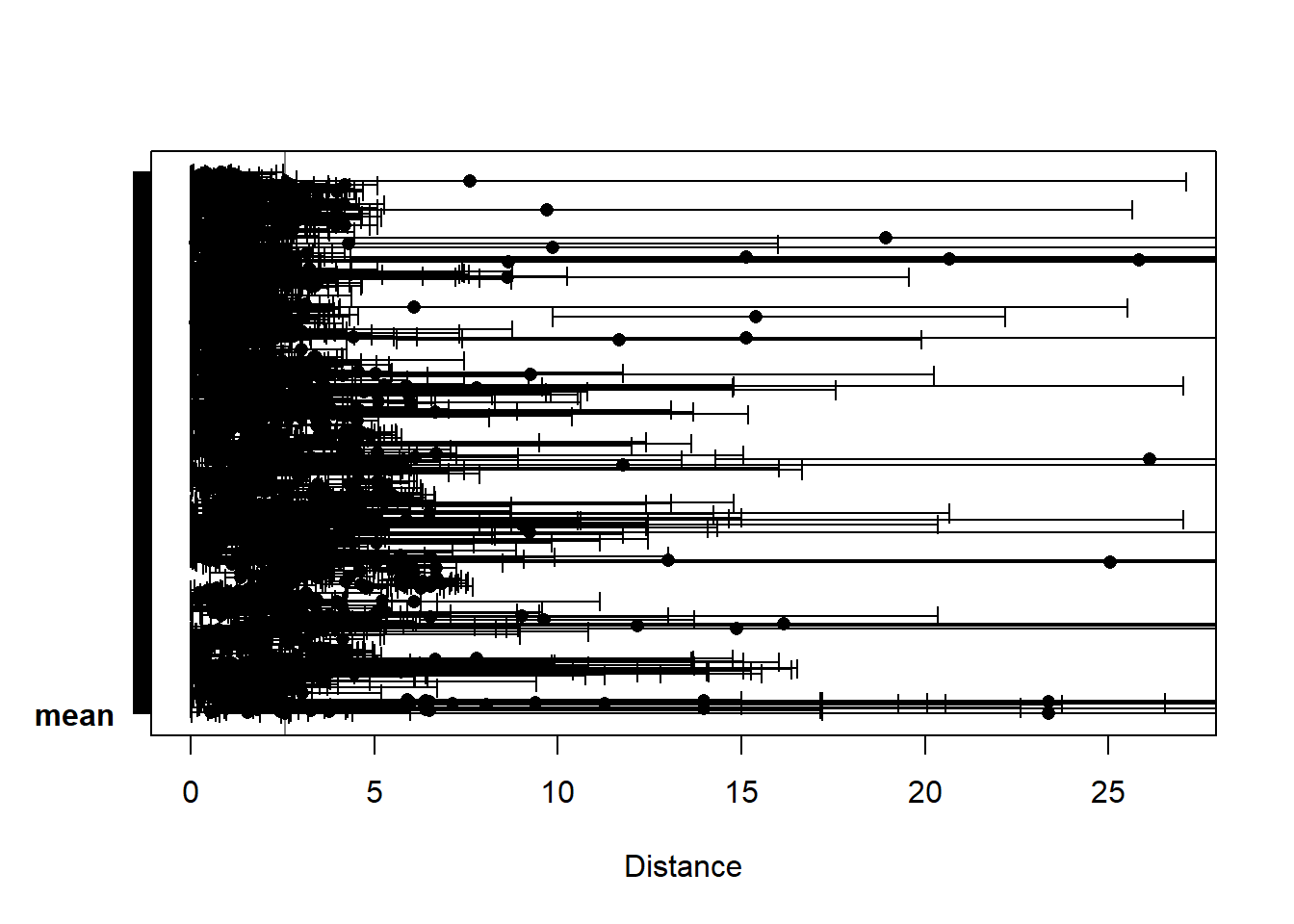

Supplement: Supplementary file 2 — Supplementary Material 2 [file 40462_2025_620_MOESM2_ESM.zip › Appendix_B_code/tracking_method_comparison_ANNOTATED_CODE_files/figure-html/unnamed-chunk-29-4.png]

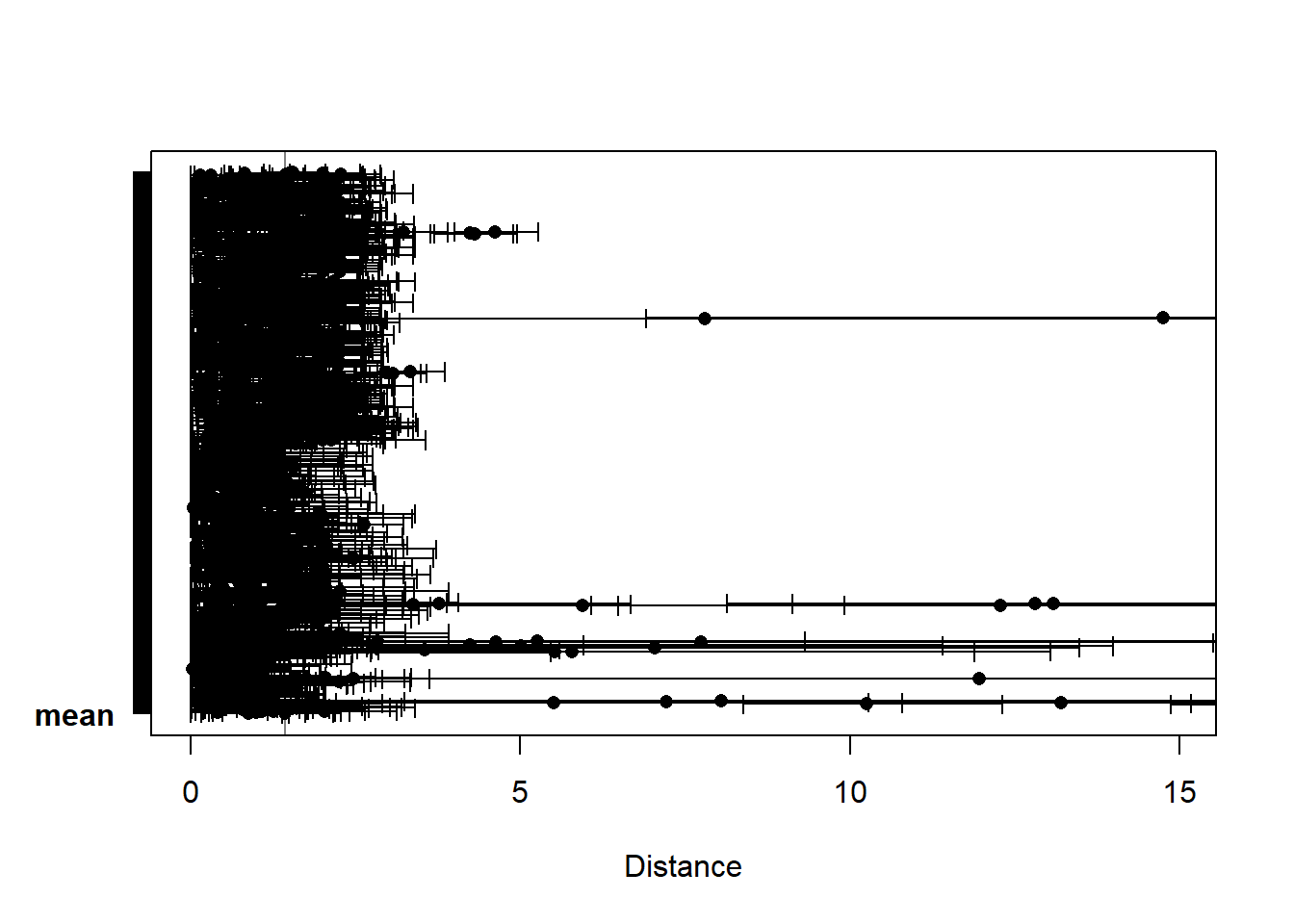

Supplement: Supplementary file 2 — Supplementary Material 2 [file 40462_2025_620_MOESM2_ESM.zip › Appendix_B_code/tracking_method_comparison_ANNOTATED_CODE_files/figure-html/unnamed-chunk-29-5.png]

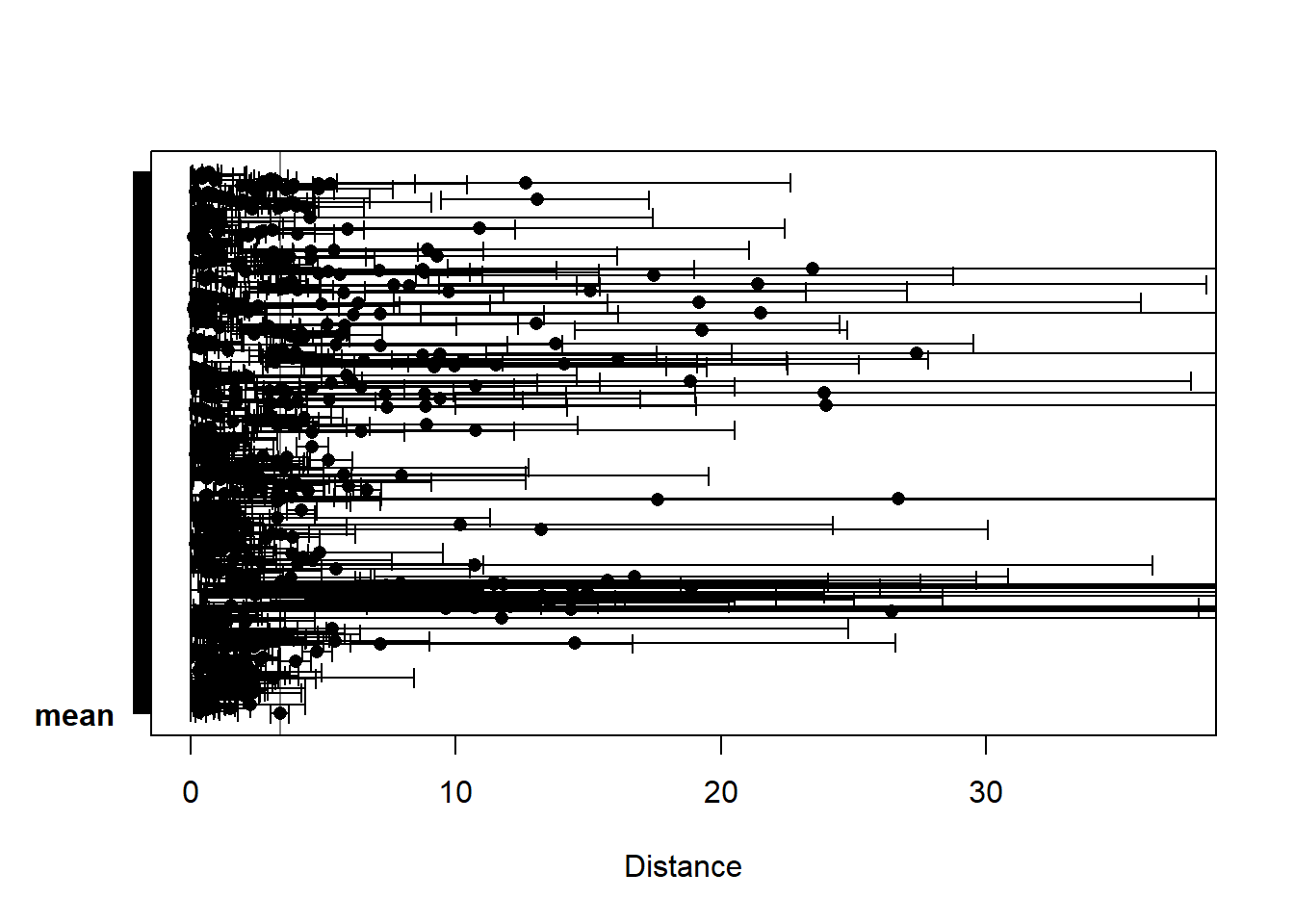

Supplement: Supplementary file 2 — Supplementary Material 2 [file 40462_2025_620_MOESM2_ESM.zip › Appendix_B_code/tracking_method_comparison_ANNOTATED_CODE_files/figure-html/unnamed-chunk-29-6.png]

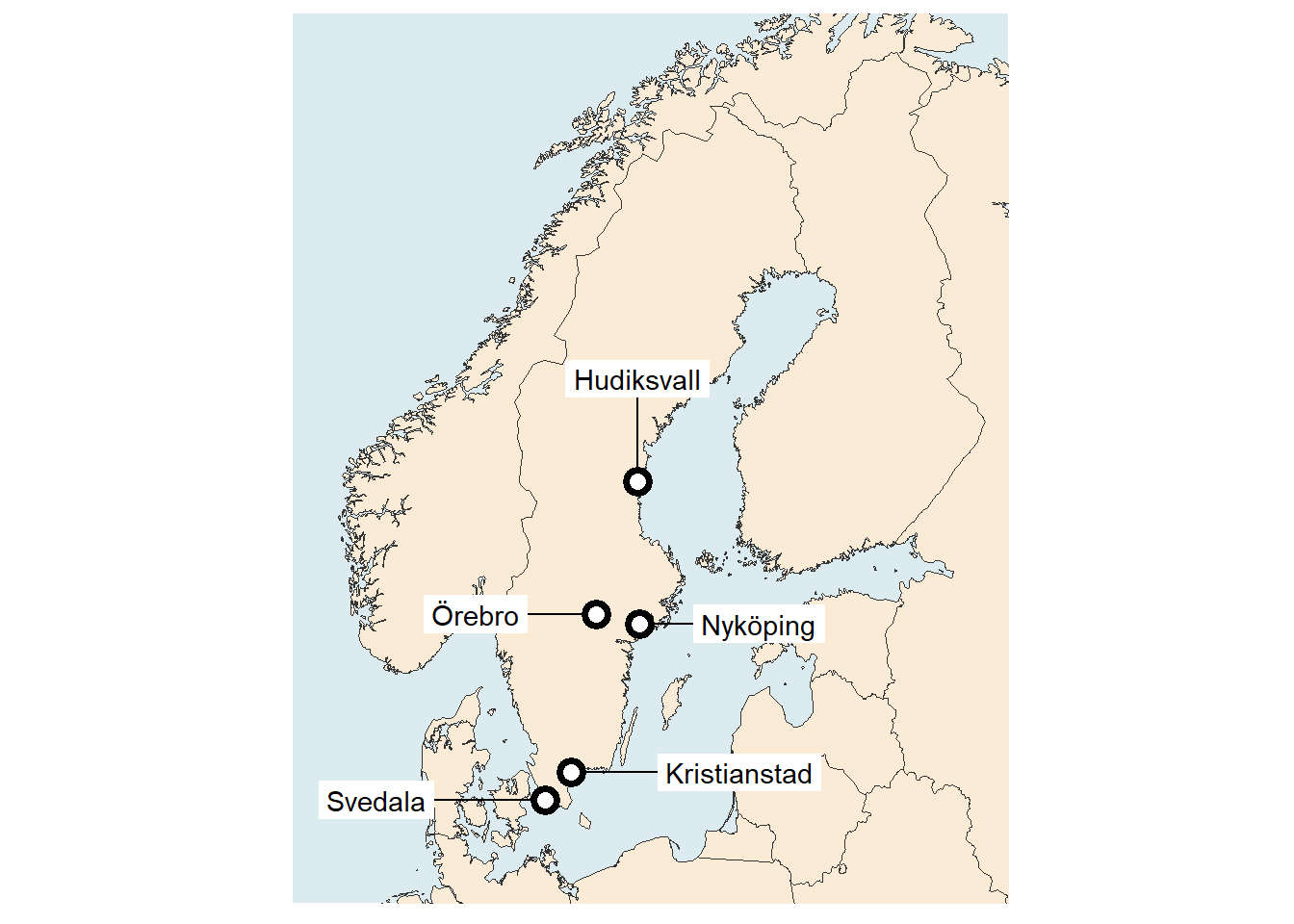

Supplement: Supplementary file 2 — Supplementary Material 2 [file 40462_2025_620_MOESM2_ESM.zip › Appendix_B_code/tracking_method_comparison_ANNOTATED_CODE_files/figure-html/unnamed-chunk-3-1.png]

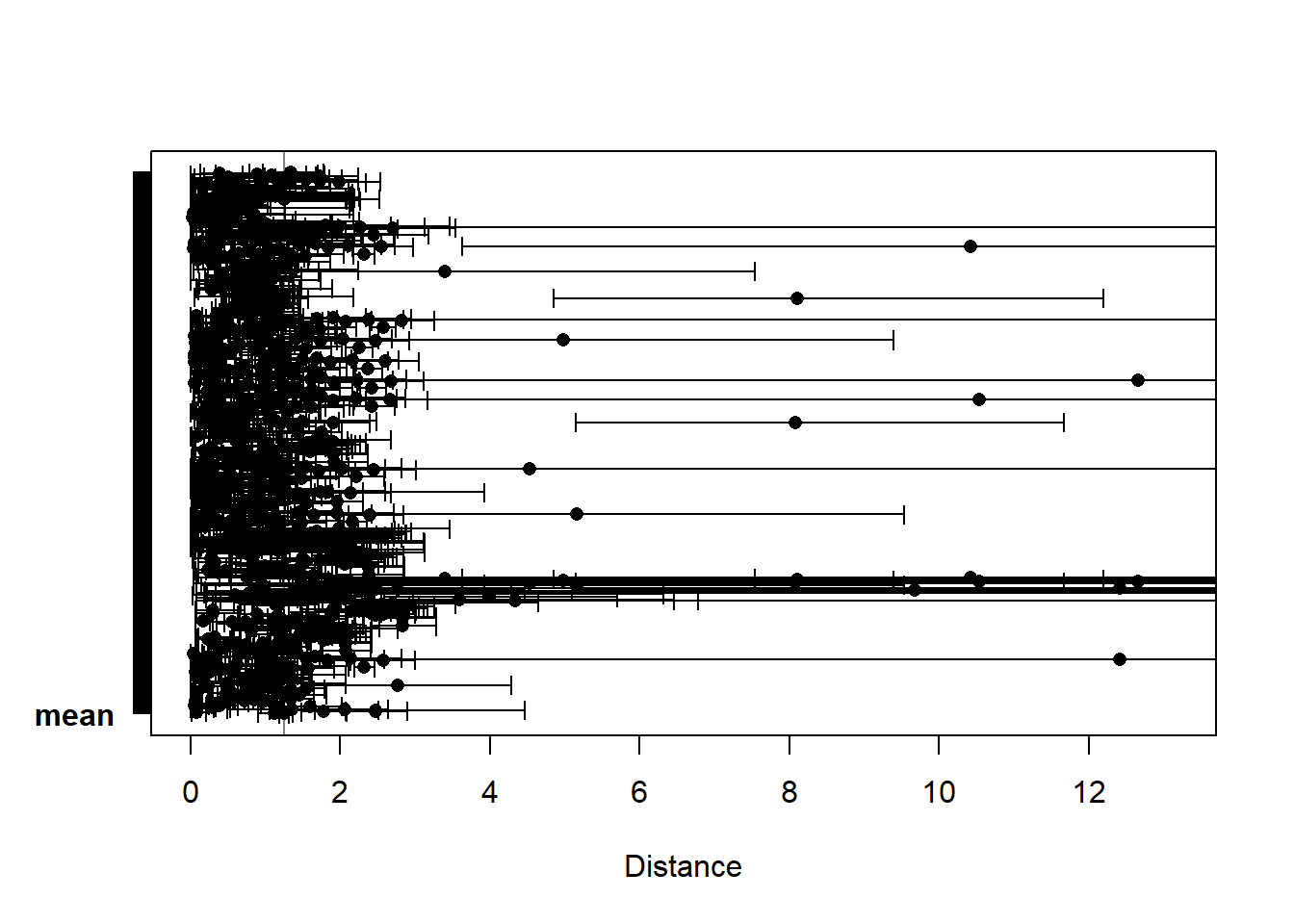

Supplement: Supplementary file 2 — Supplementary Material 2 [file 40462_2025_620_MOESM2_ESM.zip › Appendix_B_code/tracking_method_comparison_ANNOTATED_CODE_files/figure-html/unnamed-chunk-30-1.png]

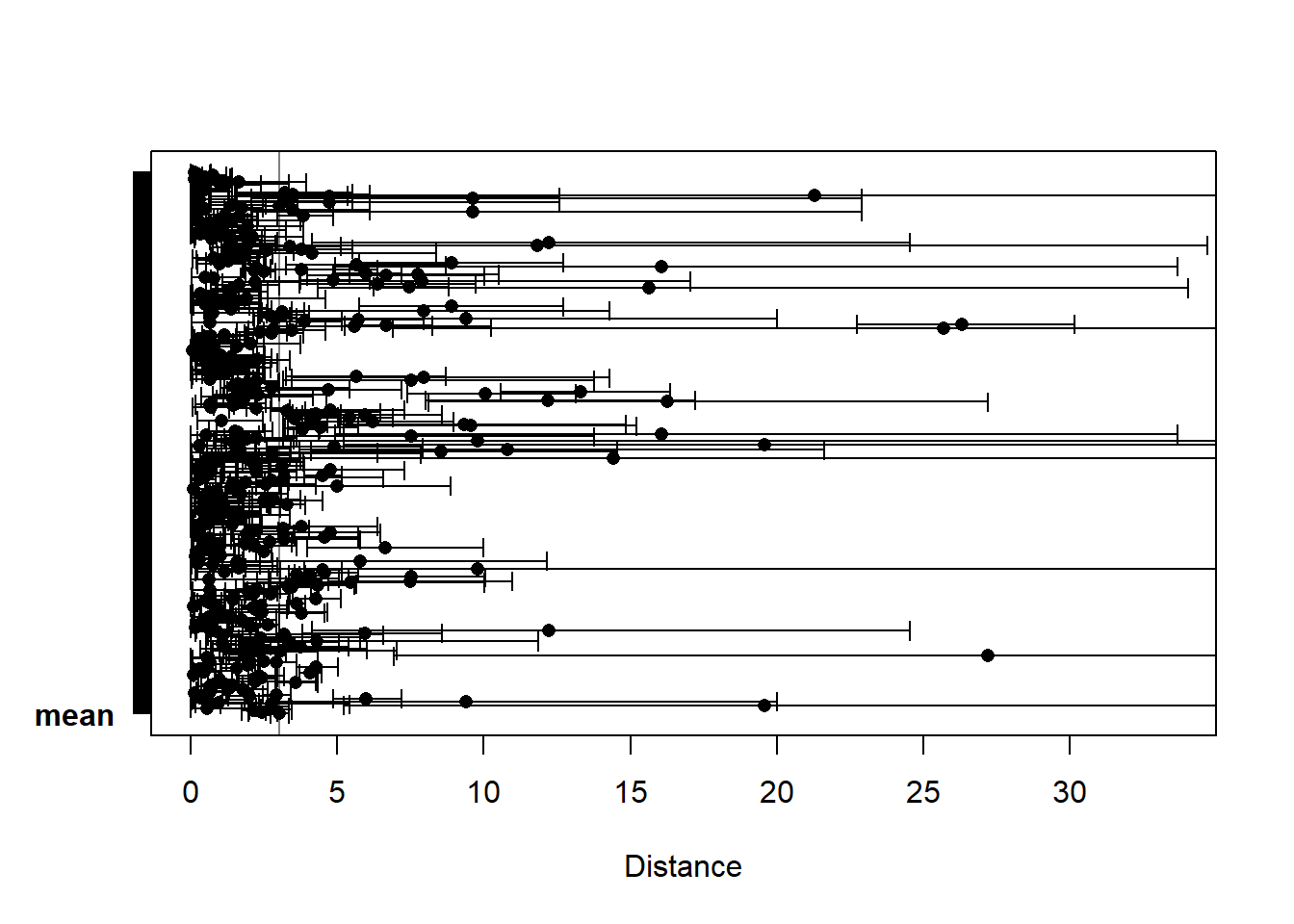

Supplement: Supplementary file 2 — Supplementary Material 2 [file 40462_2025_620_MOESM2_ESM.zip › Appendix_B_code/tracking_method_comparison_ANNOTATED_CODE_files/figure-html/unnamed-chunk-30-2.png]

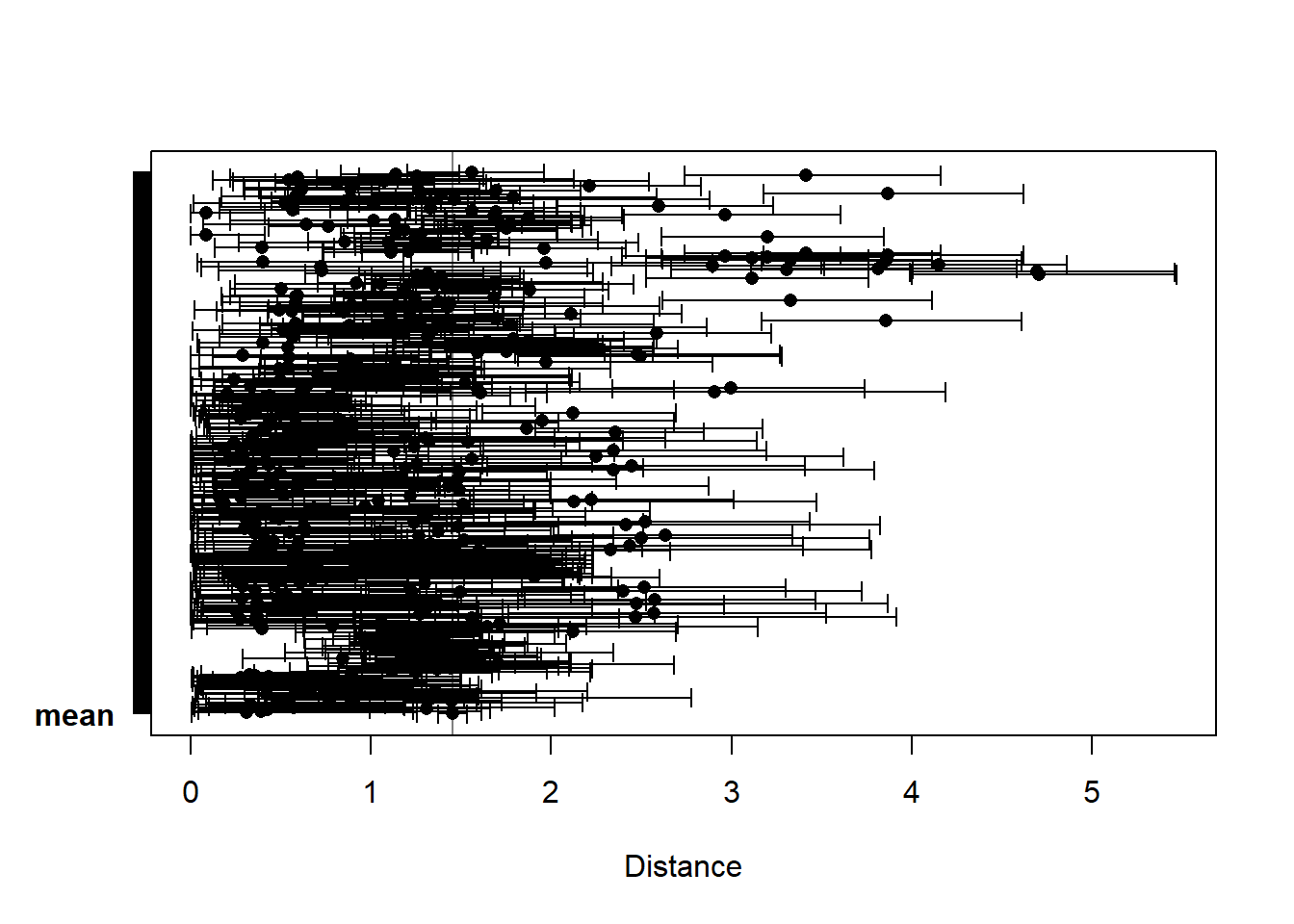

Supplement: Supplementary file 2 — Supplementary Material 2 [file 40462_2025_620_MOESM2_ESM.zip › Appendix_B_code/tracking_method_comparison_ANNOTATED_CODE_files/figure-html/unnamed-chunk-30-3.png]

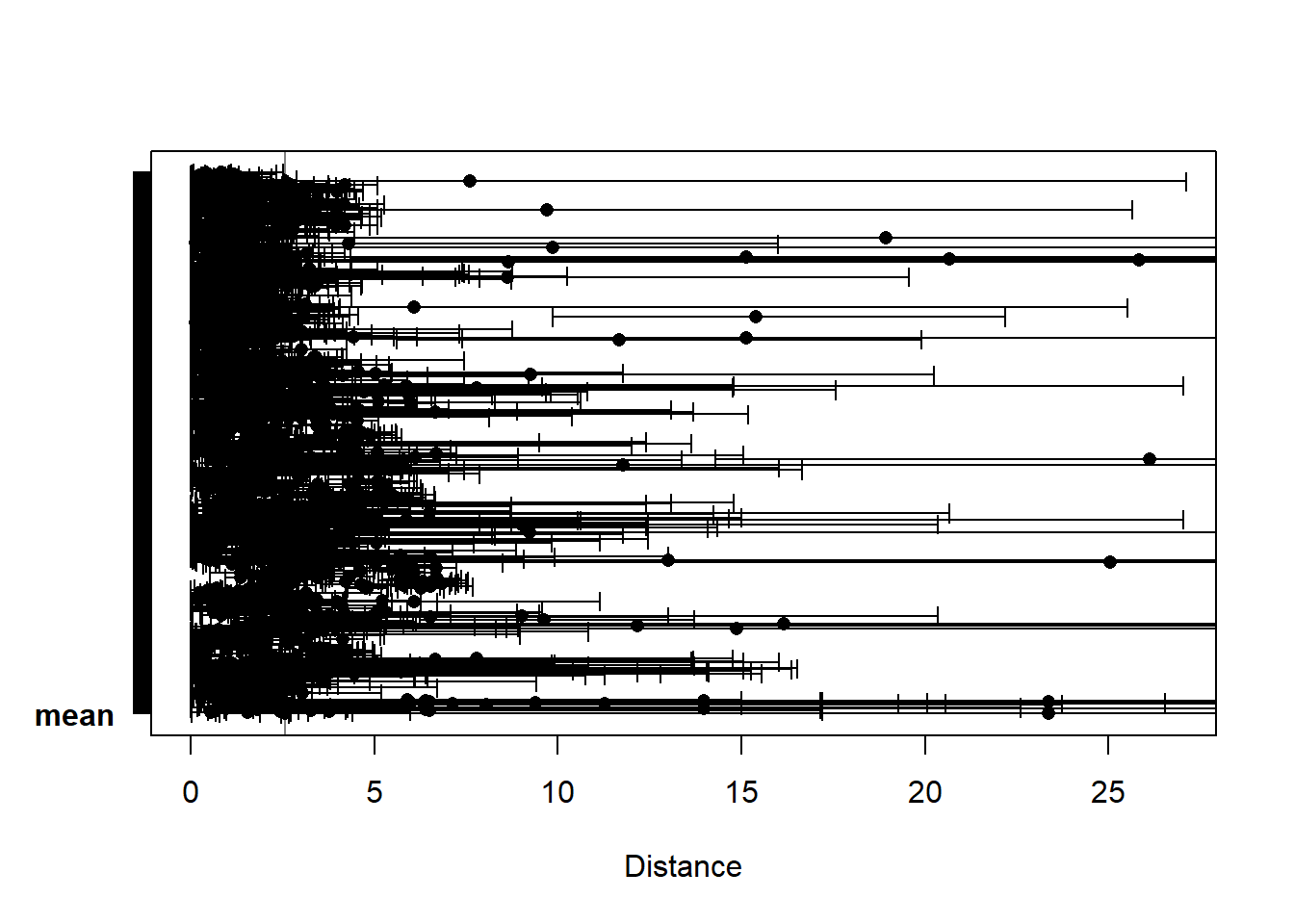

Supplement: Supplementary file 2 — Supplementary Material 2 [file 40462_2025_620_MOESM2_ESM.zip › Appendix_B_code/tracking_method_comparison_ANNOTATED_CODE_files/figure-html/unnamed-chunk-30-4.png]

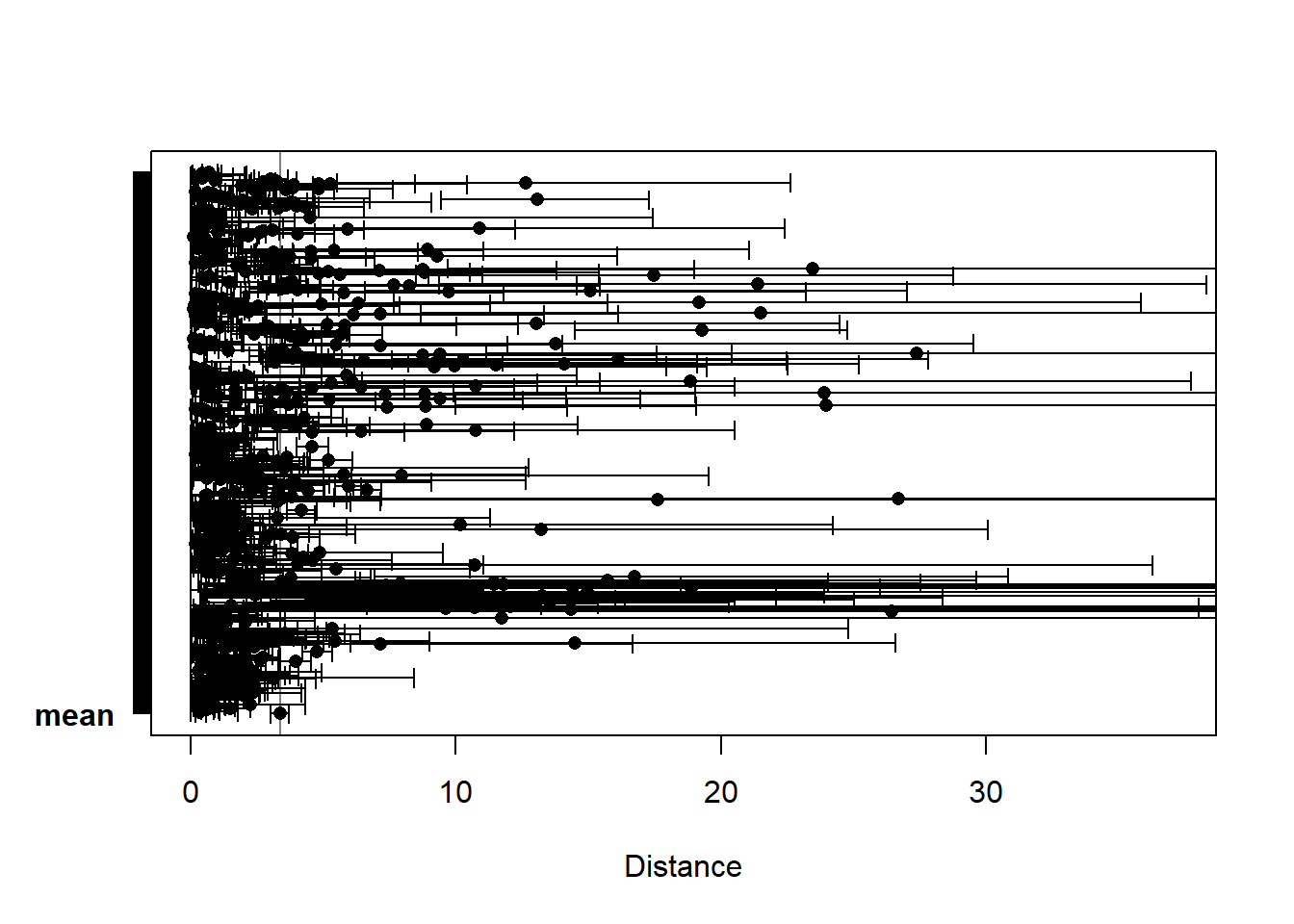

Supplement: Supplementary file 2 — Supplementary Material 2 [file 40462_2025_620_MOESM2_ESM.zip › Appendix_B_code/tracking_method_comparison_ANNOTATED_CODE_files/figure-html/unnamed-chunk-30-6.png]

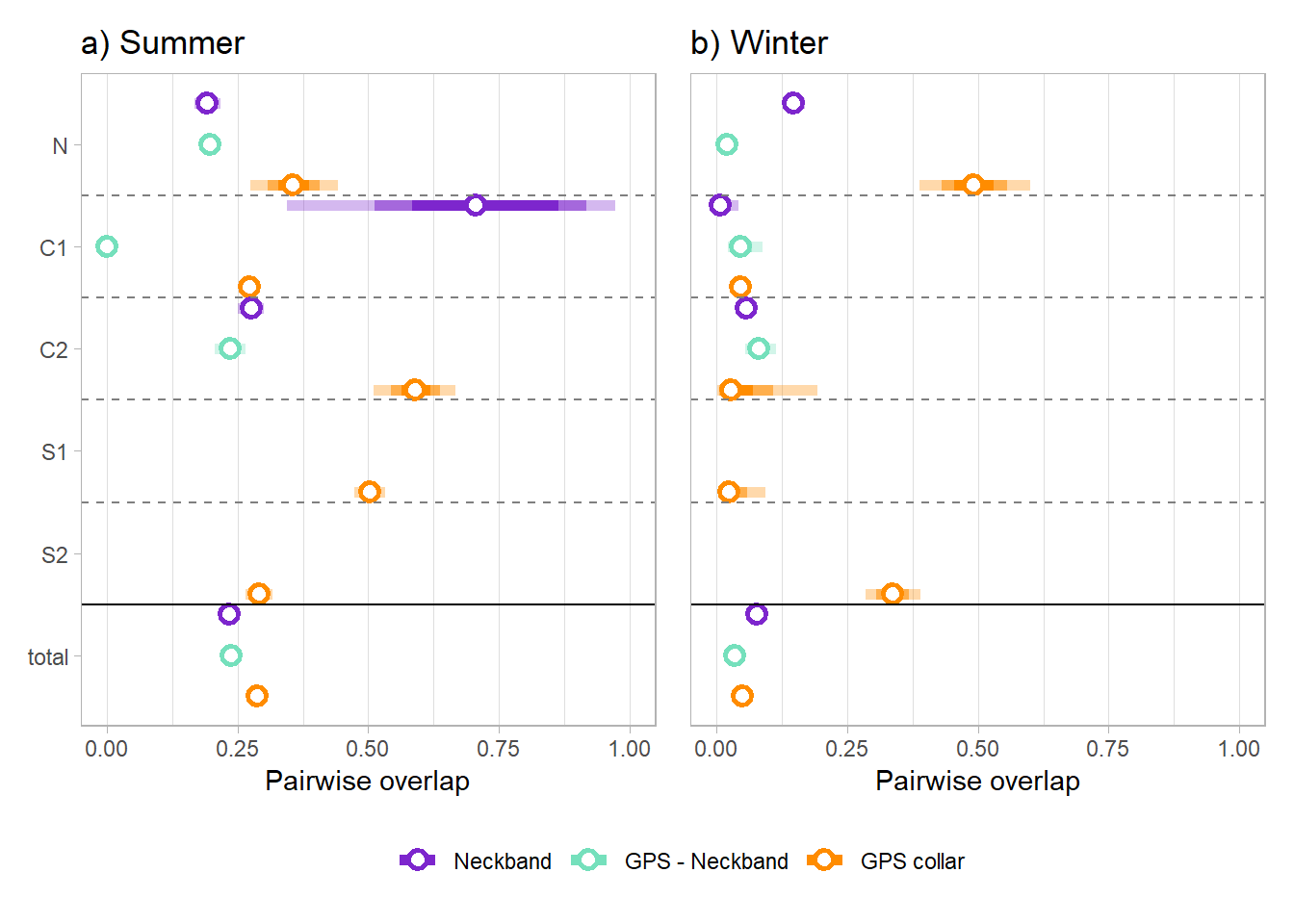

Supplement: Supplementary file 2 — Supplementary Material 2 [file 40462_2025_620_MOESM2_ESM.zip › Appendix_B_code/tracking_method_comparison_ANNOTATED_CODE_files/figure-html/unnamed-chunk-31-1.png]

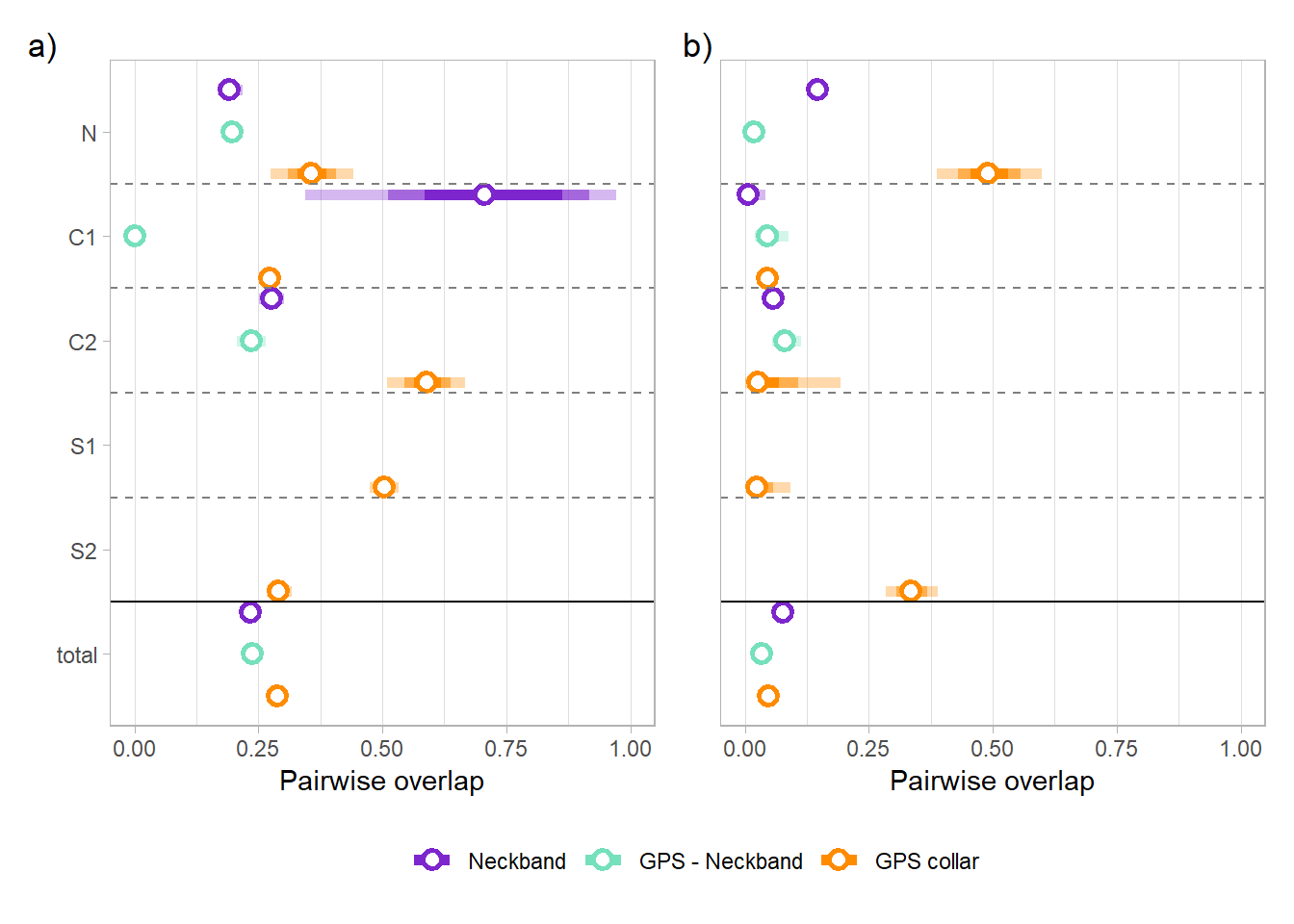

Supplement: Supplementary file 2 — Supplementary Material 2 [file 40462_2025_620_MOESM2_ESM.zip › Appendix_B_code/tracking_method_comparison_ANNOTATED_CODE_files/figure-html/unnamed-chunk-31-2.png]

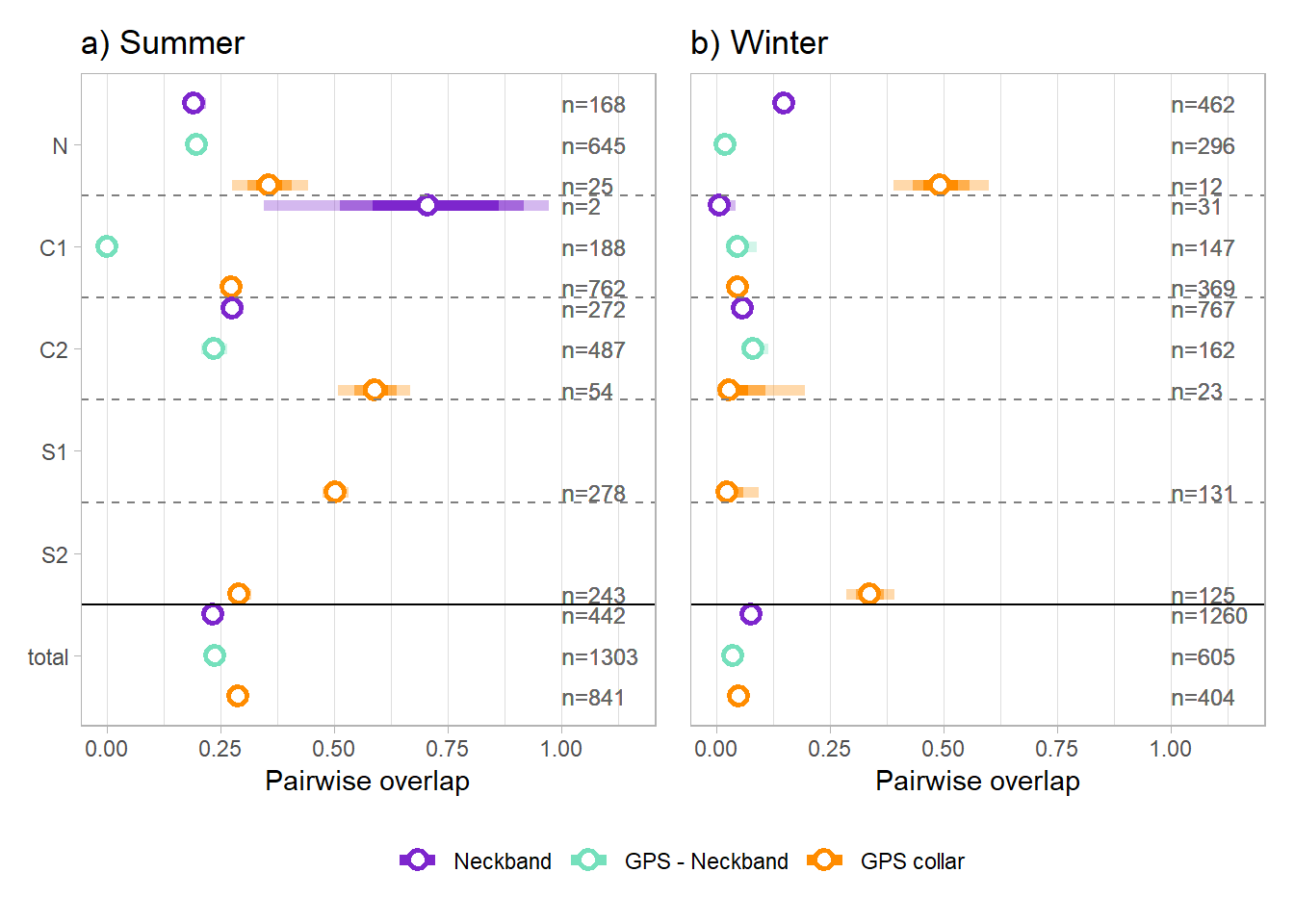

Supplement: Supplementary file 2 — Supplementary Material 2 [file 40462_2025_620_MOESM2_ESM.zip › Appendix_B_code/tracking_method_comparison_ANNOTATED_CODE_files/figure-html/unnamed-chunk-32-1.png]

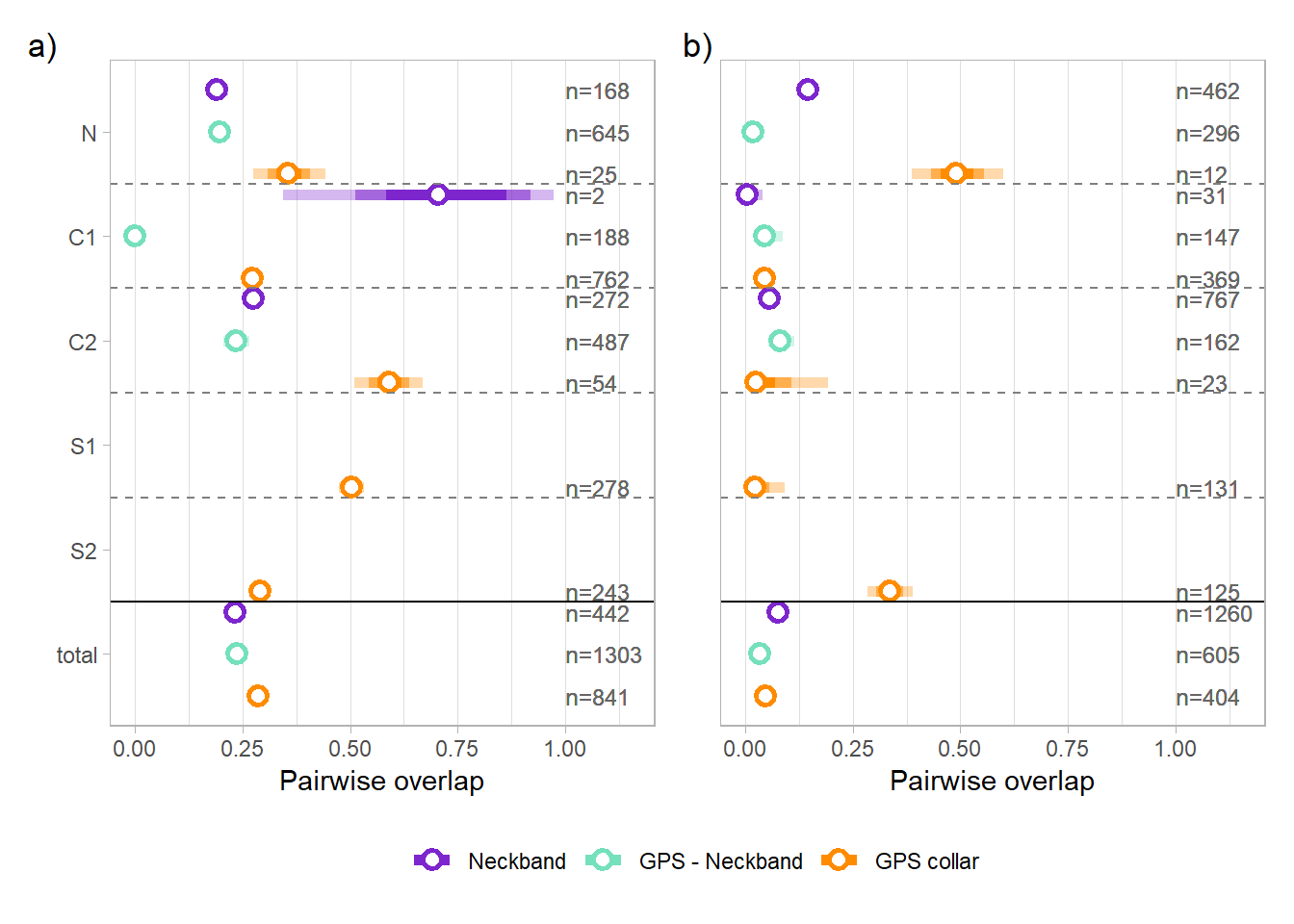

Supplement: Supplementary file 2 — Supplementary Material 2 [file 40462_2025_620_MOESM2_ESM.zip › Appendix_B_code/tracking_method_comparison_ANNOTATED_CODE_files/figure-html/unnamed-chunk-32-2.png]

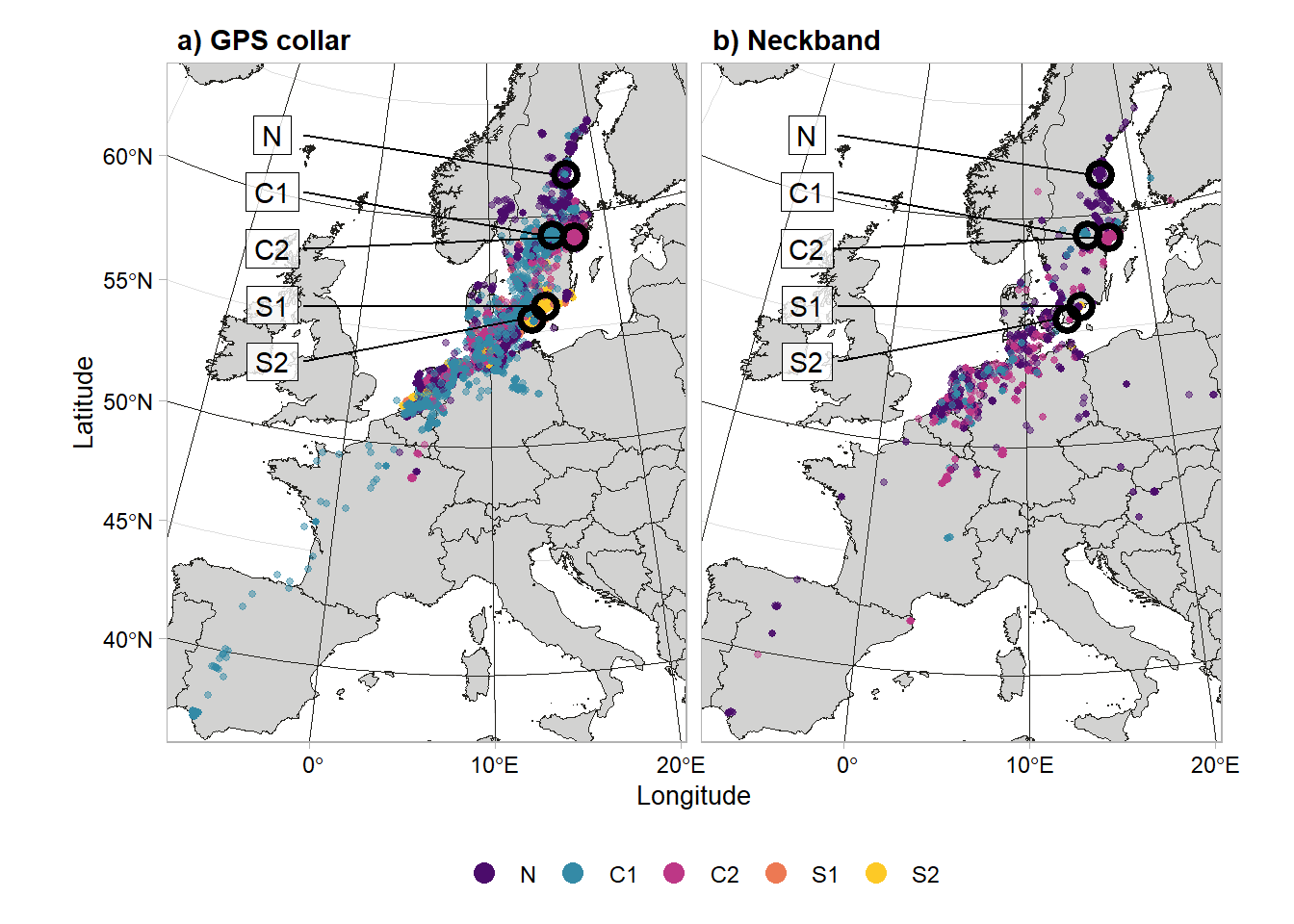

Supplement: Supplementary file 2 — Supplementary Material 2 [file 40462_2025_620_MOESM2_ESM.zip › Appendix_B_code/tracking_method_comparison_ANNOTATED_CODE_files/figure-html/unnamed-chunk-4-1.png]

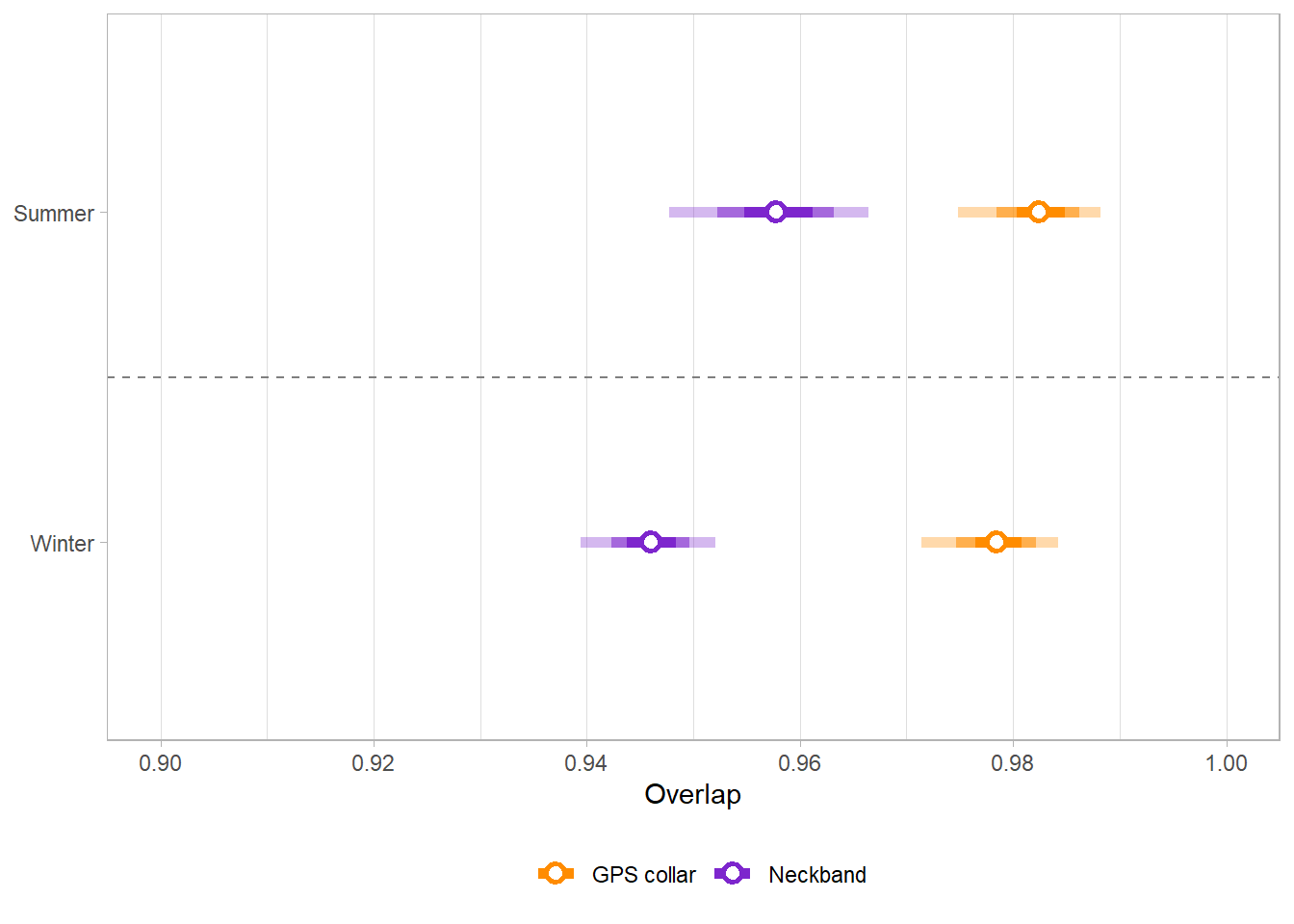

Supplement: Supplementary file 2 — Supplementary Material 2 [file 40462_2025_620_MOESM2_ESM.zip › Appendix_B_code/tracking_method_comparison_ANNOTATED_CODE_files/figure-html/unnamed-chunk-43-1.png]

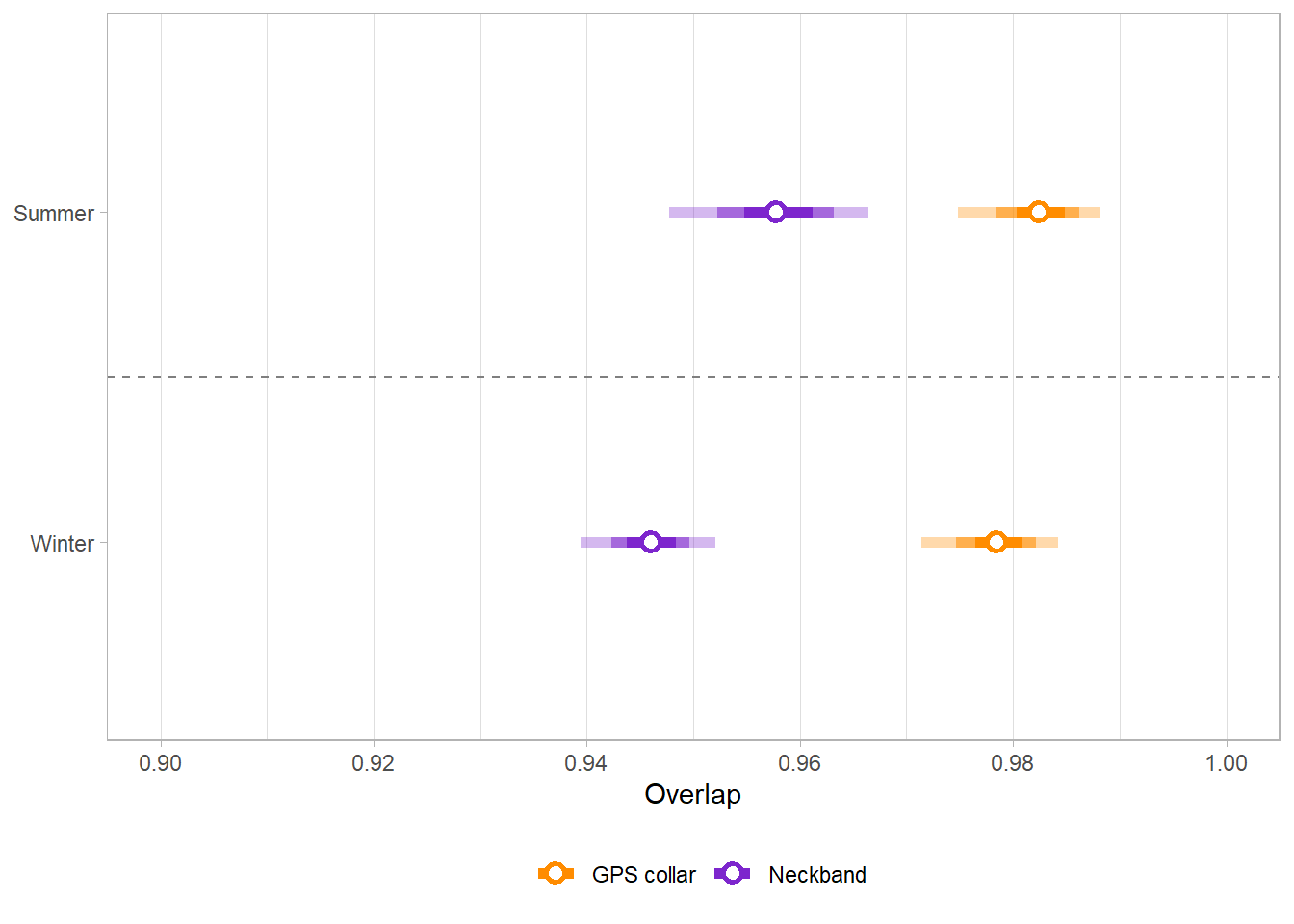

Supplement: Supplementary file 2 — Supplementary Material 2 [file 40462_2025_620_MOESM2_ESM.zip › Appendix_B_code/tracking_method_comparison_ANNOTATED_CODE_files/figure-html/unnamed-chunk-44-1.png]

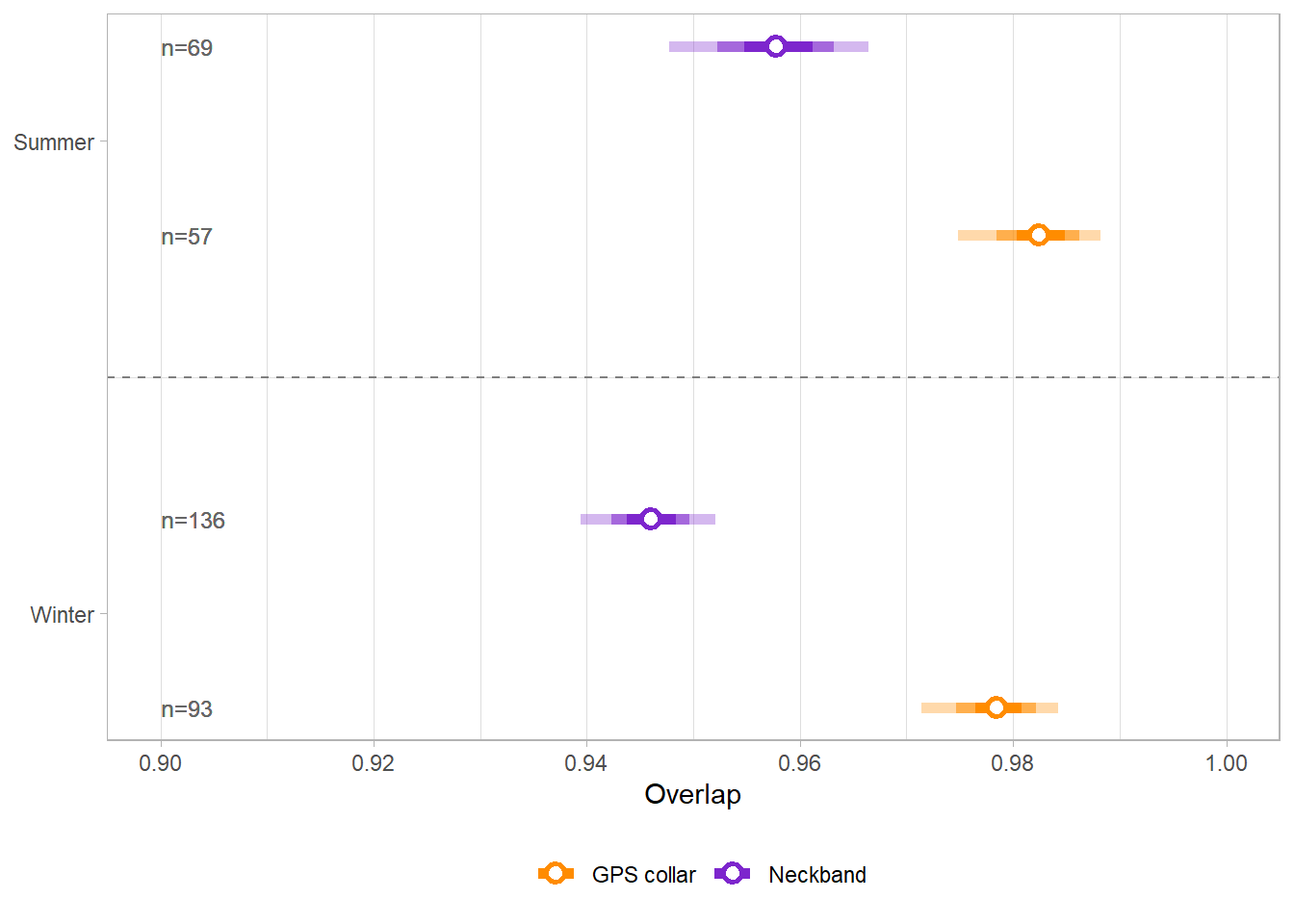

Supplement: Supplementary file 2 — Supplementary Material 2 [file 40462_2025_620_MOESM2_ESM.zip › Appendix_B_code/tracking_method_comparison_ANNOTATED_CODE_files/figure-html/unnamed-chunk-45-1.png]

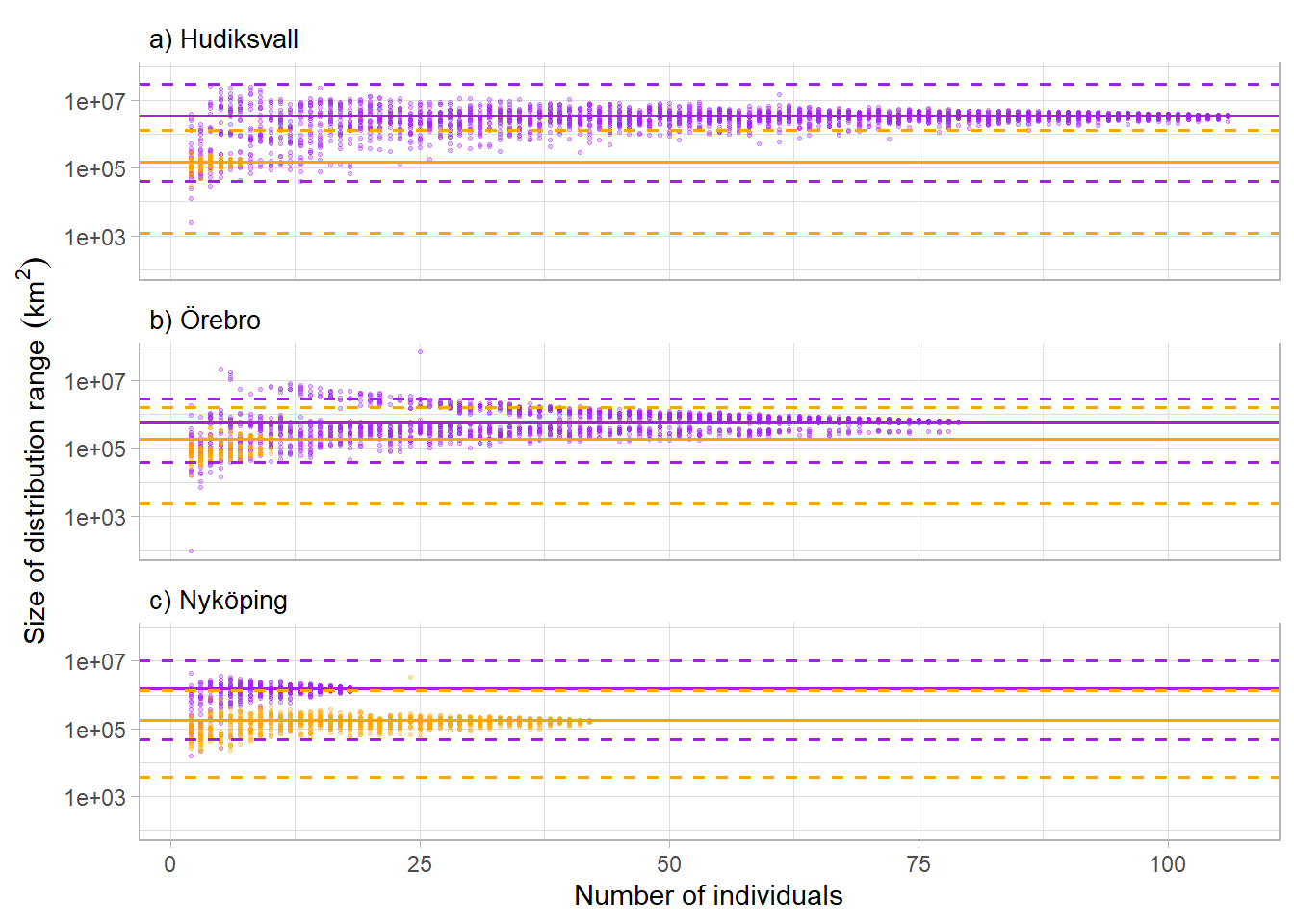

Supplement: Supplementary file 2 — Supplementary Material 2 [file 40462_2025_620_MOESM2_ESM.zip › Appendix_B_code/tracking_method_comparison_ANNOTATED_CODE_files/figure-html/unnamed-chunk-48-1.png]

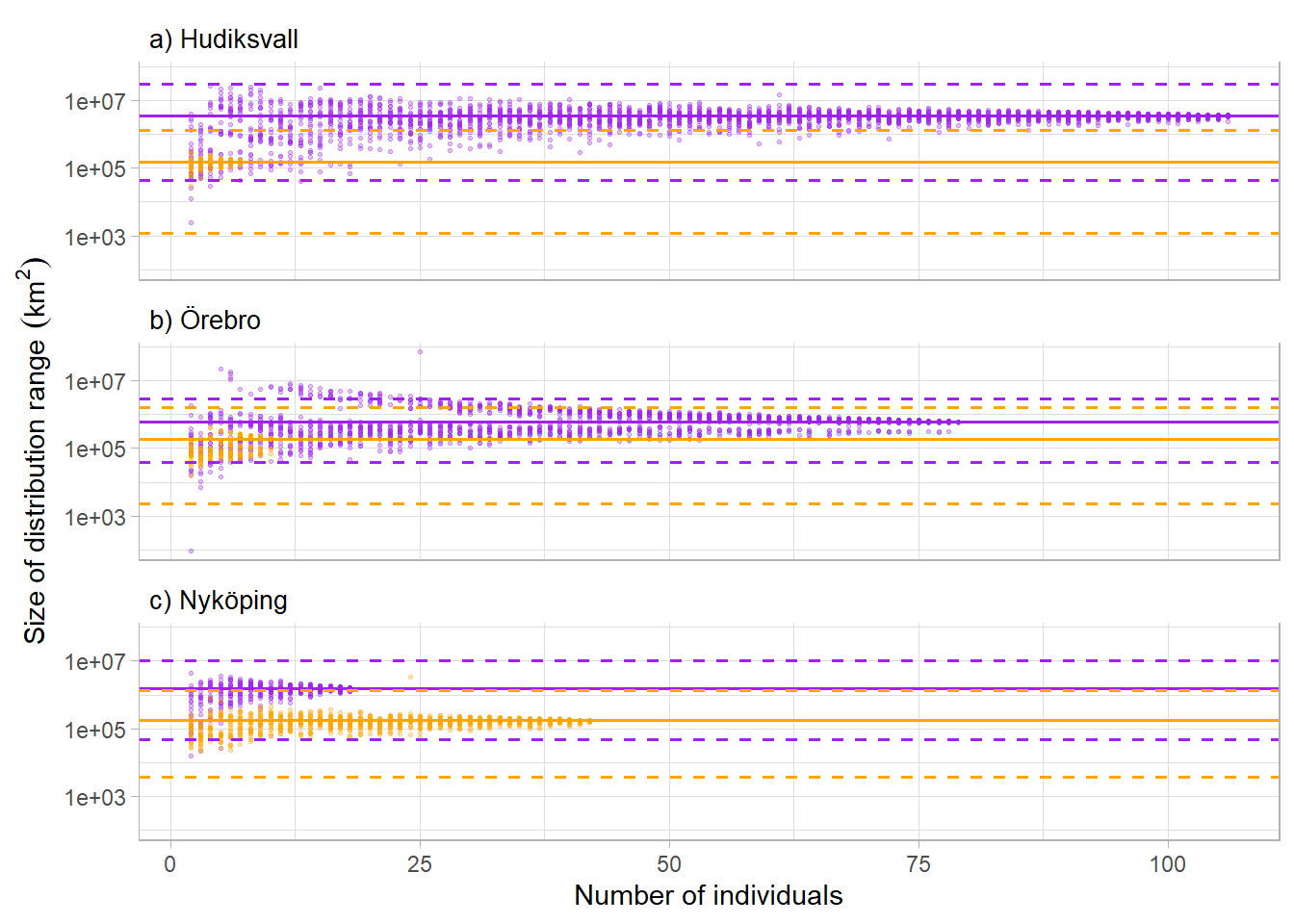

Supplement: Supplementary file 2 — Supplementary Material 2 [file 40462_2025_620_MOESM2_ESM.zip › Appendix_B_code/tracking_method_comparison_ANNOTATED_CODE_files/figure-html/unnamed-chunk-49-1.png]

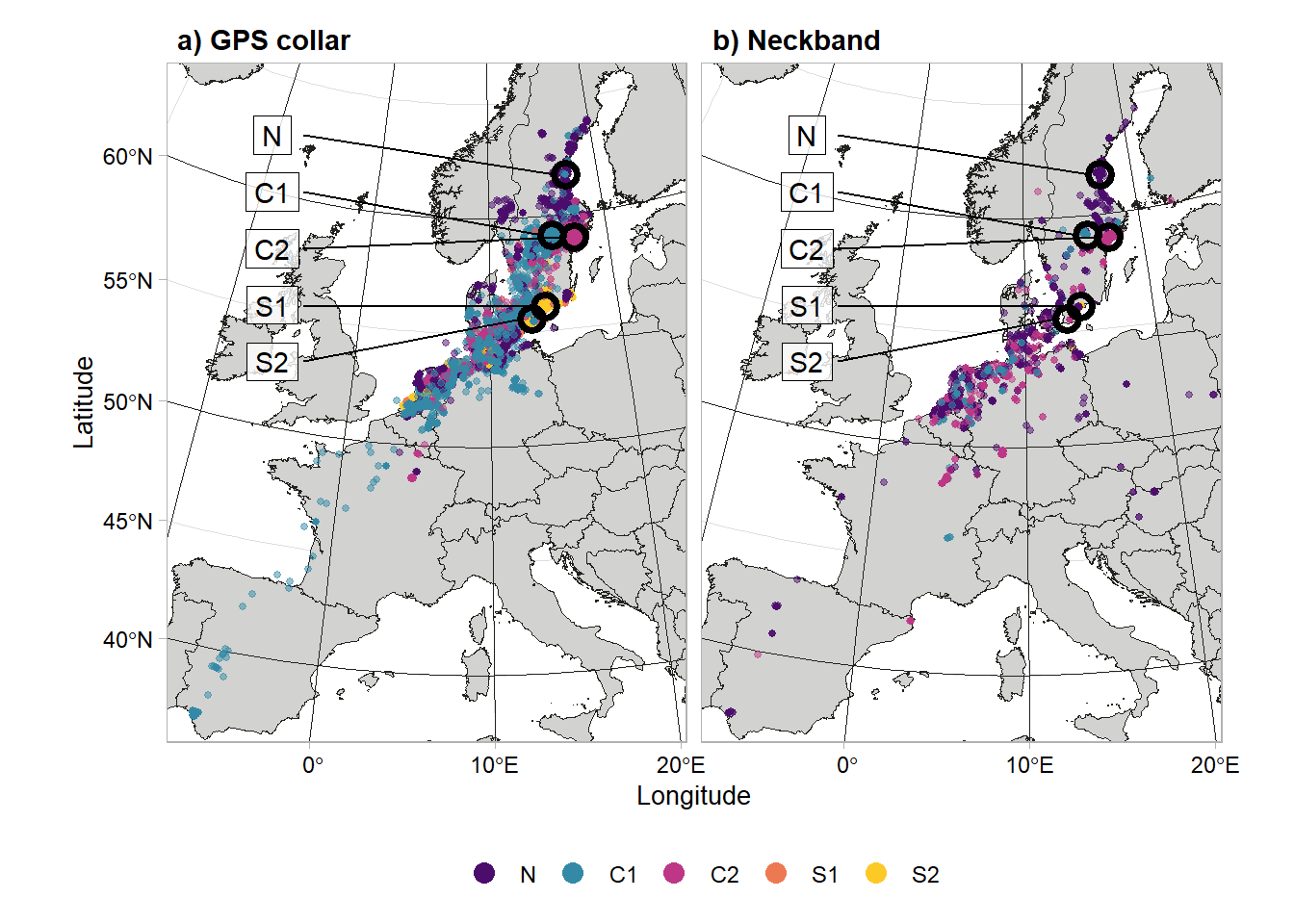

Supplement: Supplementary file 2 — Supplementary Material 2 [file 40462_2025_620_MOESM2_ESM.zip › Appendix_B_code/tracking_method_comparison_ANNOTATED_CODE_files/figure-html/unnamed-chunk-5-1.png]

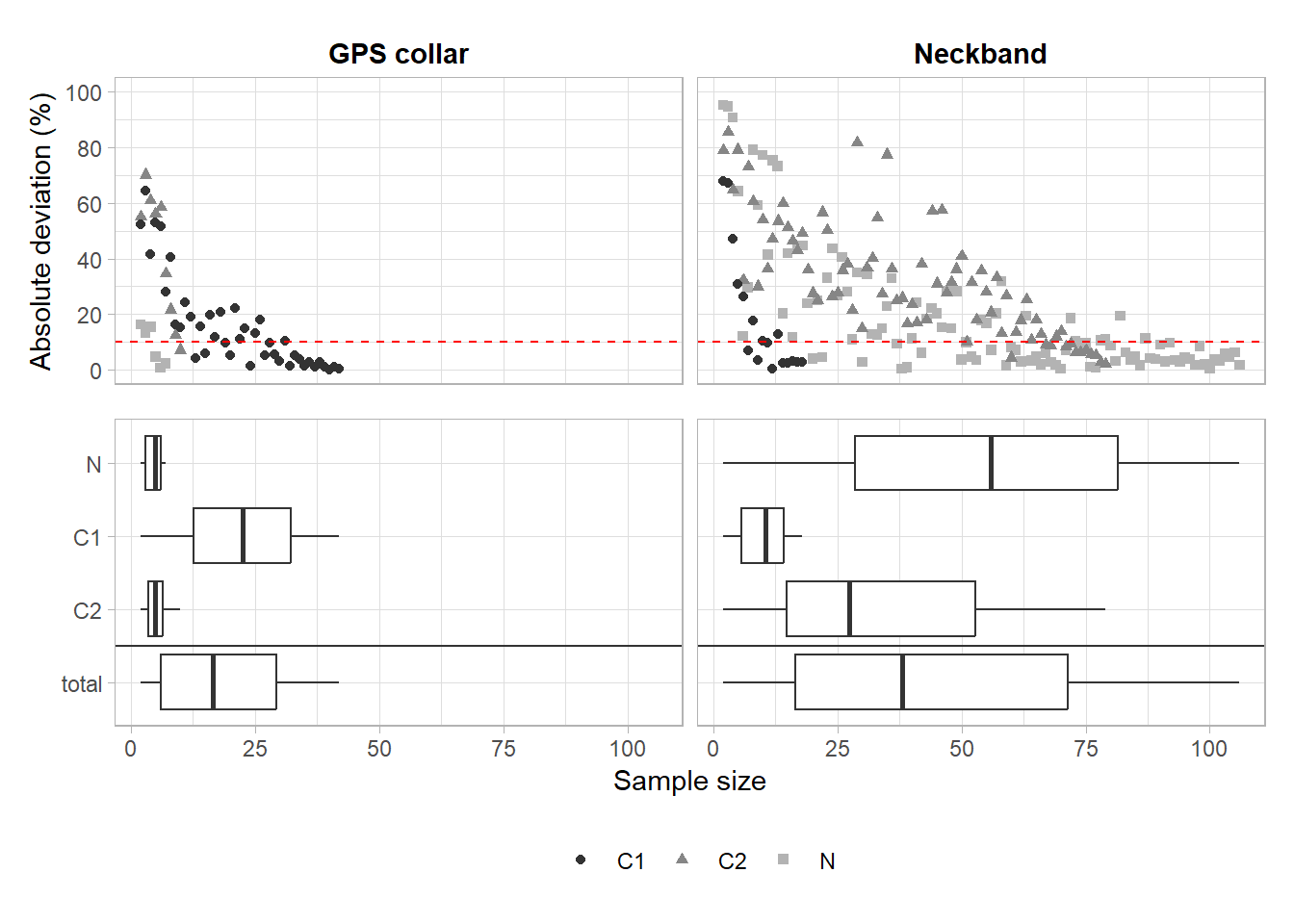

Supplement: Supplementary file 2 — Supplementary Material 2 [file 40462_2025_620_MOESM2_ESM.zip › Appendix_B_code/tracking_method_comparison_ANNOTATED_CODE_files/figure-html/unnamed-chunk-51-1.png]
